# Modular Synthetic Approach to Silicon-Rhodamine Homologues and Analogues via Bis-aryllanthanum Reagents

**DOI:** 10.1021/acs.orglett.1c00512

**Published:** 2021-03-15

**Authors:** Alexey N. Butkevich

**Affiliations:** Department of Optical Nanoscopy, Max Planck Institute for Medical Research, Jahnstrasse 29, 69120 Heidelberg, Germany

## Abstract

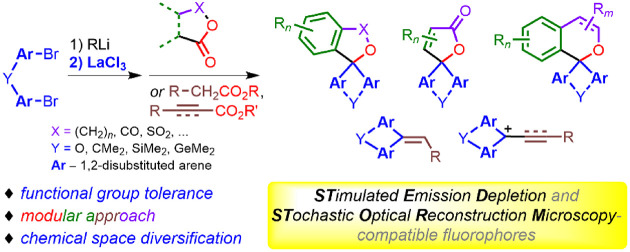

A modular synthetic approach toward diverse analogues of the far-red fluorophore silicon-rhodamine (SiR), based on a regioselective double nucleophilic addition of aryllanthanum reagents to esters, anhydrides, and lactones, is proposed. The reaction has improved functional group tolerance and represents a unified strategy toward cell-permeant, spontaneously blinking, and photoactivatable SiR fluorescent labels. In tandem with Pd-catalyzed hydroxy- or aminocarbonylation, it serves a streamlined synthetic pathway to a series of validated live-cell-compatible fluorescent dyes.

With the recent developments in molecular-size superresolution fluorescence microscopy techniques,^1^ the problem of design and development of suitable small-molecule fluorescent probes is becoming central to achieving practicality and fidelity in nanoscale fluorescence imaging. In particular, there is a deficiency in the range and diversity of photoactivatable or photoconvertible small-molecule fluorophores that also exhibit high emission brightness and photostability.^2^ Additional requirements of cell membrane permeability and live-cell compatibility are posed whenever superresolution imaging in live cells or tissues is required.^3^ Among the successful developments of the past decade, numerous triarylmethane fluorophores, in particular the rhodamine analogues with the oxygen bridge replaced with CMe_2_, SiMe_2_, GeMe_2_, and P(O)R groups to achieve bathochromic shifts of absorption and emission, have been reported.^4^ However, the latest disclosures^5^ show that the demand for chemical diversity of newly designed labels exceeds the scope of established synthetic chemistry of triarylmethane dyes, and the synthetic methods offering practicable yields are often lacking.

Recently,^6^ a reaction of bis-aryllithium or bis-arylmagnesium reagents has been proposed as an alternative synthetic strategy toward O-, Si-, and P-rhodamines and Si-fluoresceins (Scheme 1). While this method did provide access to an expanded range of triarylmethane fluorophores, in our hands for more demanding examples we found it operationally complex, as it required slow additions and temperature optimization across the interval between −78 °C and rt.^6b−6e^ We have concluded that transmetalation with a more Lewis acidic metal salt should mitigate the undesired reactivity of ArLi or ArMgX species stemming from single-electron transfer pathways or their highly basic nature. Indeed, it is long known^7^ that alkyl- and aryllanthanide reagents of type RMX_2_ (M = La, Ce) retain high nucleophilicity toward carbonyl groups and offer good yields with base-sensitive substrates such as CH-acids and Michael acceptors. Replacing the MgBr_2_ additive with the commercially available LaCl_3_·2LiCl complex,^7c^ we were able to quickly extend this chemistry to a number of sensitive substrates. In this report, we highlight the scope of this transformation and demonstrate its application to the synthesis of established live-cell-compatible fluorescent labels.

**Scheme 1:**
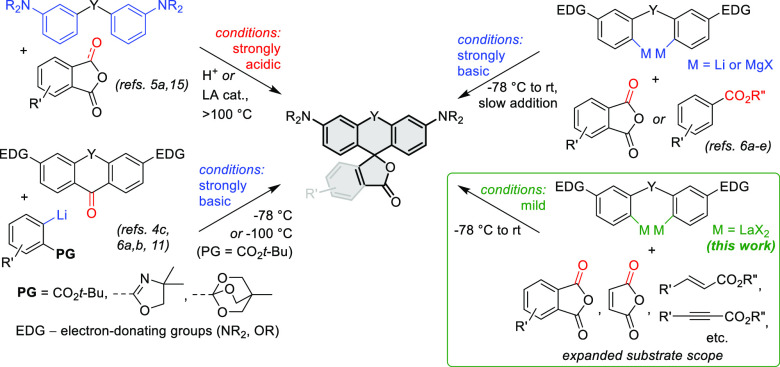
Synthetic Approaches to 5*H*-Spiro[furan-2,9′-xanthenes] (Y = O) and Their Analogues (Y = CMe_2_, SiMe_2_, etc.)

The reaction between the bis-aryllanthanum reagents derived from **1a**–**d** and cyclic anhydrides **2a**–**j** resulted in formation of the expected spirocyclic products with 40–70% isolated yields (Scheme 2a). In the case of 3-substituted phthalic anhydrides (**2f**,**i**,**j**), double nucleophilic addition proceeded regioselectively at the least substituted carbonyl group. This method allowed the preparation of previously inaccessible Si-rhodamine analogues **3a**,**b** lacking the pendant *o*-phenylene ring and containing an electrophilic cyclic unsaturated ester functionality, poorly compatible with basic RLi or RMgX nucleophiles. Reactive NO_2_ and F substituents in the aryl ring of the electrophilic partner were also tolerated. The new procedure provided markedly improved yields in the reactions with 3-bromophthalic (**3j**; 67% vs 5%) and glutaric anhydrides (thus, the photoactivatable fluorophore PA-SiR^5b^ was isolated in 71% vs reported 5% yield). Under identical conditions, the addition of bis-aryllanthanum reagent was 1,2-selective with α,β-unsaturated esters, producing 9-alkenyl- and 9-alkynyl-substituted Si-xanthenes **3n** and **3o**.^8^

**Scheme 2:**
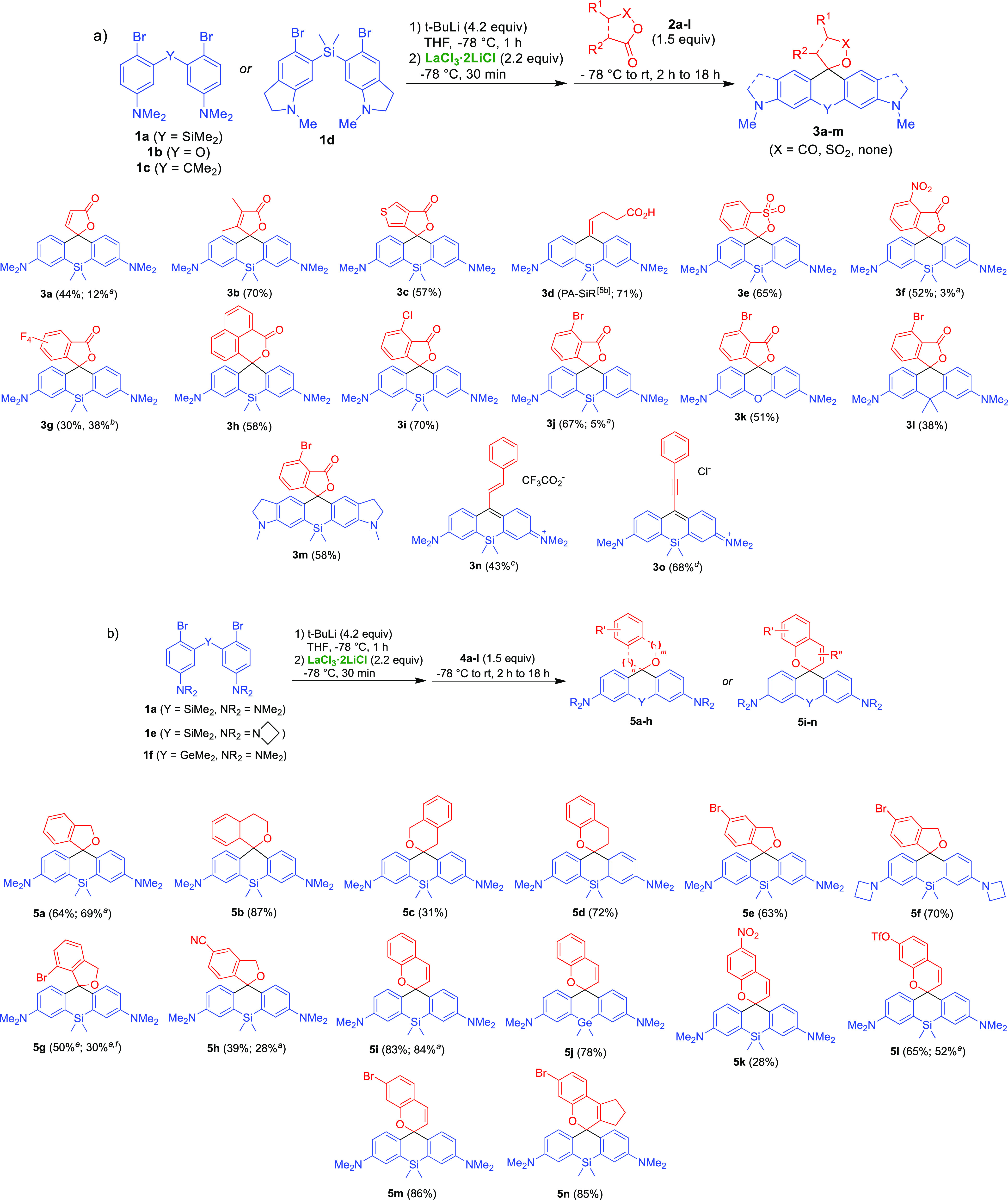
Reaction of Aryllanthanum Reagents Generated from **1a**–**f** with (a) Anhydrides **2a**–**j** and Esters **2g′,k,l**, and (b) Lactones **4a**–**l** Reaction yield with bis-aryllithium reagent without addition of LaCl_3_. Reaction with dimethyl tetrafluorophthalate (**2g′**) instead of tetrafluorophthalic anhydride (**2g**). Reaction with methyl cinnamate (**2k**). Reaction with ethyl phenylpropiolate (**2l**). Reaction run on 2 mmol scale. Dehalogenated product (**5a**) formed instead of expected **5g**.

The reaction of aryllanthanum reagents with phthalide and coumarin provided the desired spirocyclic products **5a**,**i** in comparable yields to those obtained from nontransmetalated aryllithium reagents (Scheme 2b). However, the aryllanthanum nucleophiles better tolerated the presence of a base-sensitive aryl triflate substituent (**5l**) and, to a lesser extent, aromatic NO_2_ (**5k**) and CN (**5h**) groups. The reaction of 7-bromophthalide with the aryllanthanum reagent allows synthetic access to the little explored 7′-HMSiR fluorophore core of **5g**, providing a new entry to its derivatives recently developed into fluorogenic far-red-fluorescent labels for Cu-free click chemistry.^9^ Treatment of this substrate with the basic bis-aryllithium reagent resulted in formation of only the corresponding proto-dehalogenated product **5a**.

The expanded functional group tolerance of the proposed aryllanthanum addition allows the introduction of a one-step approach to a variety of halogen-substituted triarylmethane fluorophores (**3i**–**m**, **5e**–**g**) and their novel halogen- and triflate-substituted spirochromene analogues (**5l**–**n**). The easy access to the former prompted us to explore their potential as substrates in carbonylative cross-coupling reactions (Scheme 3). The previously reported syntheses of regioisomerically pure 4′-, 5′-, or 6′-carboxyrhodamine dyes relied on the extensive use of protecting groups.^6a,10^ We envisaged that, given the regioselectivity of bis-aryllanthanum addition to 3-substituted phthalic anhydrides, recently proposed 4′-carboxycarbo-, silico-, and oxygen-bridged rhodamine fluorophores, demonstrating outstanding imaging performance in stimulated emission depletion (STED) microscopy in living cells,^11^ can be accessed from the intermediates **3j**–**m** via a carbonylative hydroxylation or amination. To demonstrate the versatility of the proposed method, we have prepared the fluorescent dyes 4-TMR, 4-610CP, 4-SiR,^11^ and its novel analogue 4-SiR700 by carbonylative hydroxylation with CO gas safely generated in a small amount *ex situ* in a two-chamber reactor^12^ (Scheme 3a). Similarly, the established spontaneously blinking fluorophore HMSiR,^13^ employed in stochastic optical reconstruction microscopy (STORM) in live and fixed cells, along with its azetidine analogue,^14^ have been prepared from the intermediates **5e,f**. By changing the catalyst and base, the same method could also be extended to a Pd-catalyzed carbonylative amination, allowing the installation of the carboxyl group and the amide coupling to be performed in one step on a regioisomerically pure aryl bromide **3j**. Hydrolysis of the crude *tert*-butyl ester, followed by the separation of the expected product **7a** from the minor bis-carboxylic acid impurity **7b** and the amide coupling with de-*N*-Boc-cabazitaxel (H_2_N-CTX), demonstrated an efficient technical shortcut in the synthesis of a far-red fluorescent live-cell tubulin probe 4-SiR-CTX^11^ (**8**).

**Scheme 3:**
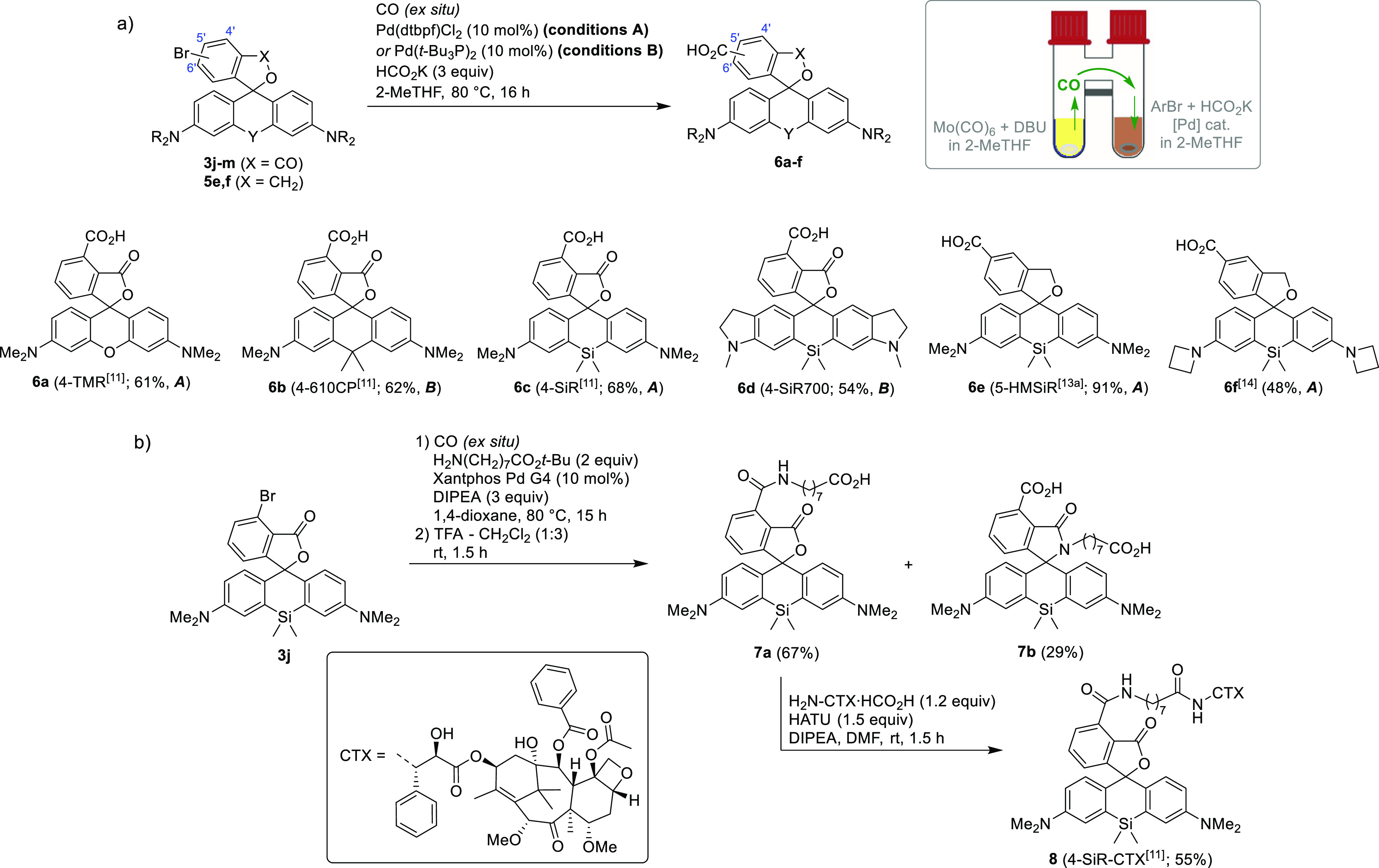
Preparation of (a) Live-Cell-Compatible Fluorescent Dyes **6a**–**f** by Pd-Catalyzed Carbonylative Hydroxylation of **3j**–**m** and **5e,f** and (b) Live-Cell Far-Red-Fluorescent Tubulin Probe 4-SiR-CTX^11^ (**8**) by Pd-Catalyzed Carbonylative Amination of **3j**

The selected properties of Si-rhodamine analogues (**3c**,**f**,**j-m** and **5a**,**b**,**d-n**), relevant for the development of fluorescent probes, are compiled in Table 1. The lactones **3a**,**b**,**h** and the spiroether **5c** exist exclusively in the colorless spirolactone forms (**I**, **III**) at pH > 1. Typical of the carbo- and Si-rhodamines is their propensity to close into the colorless form **I** under strongly acidic conditions (pH < 2), while the rhodamine dyes (e.g., **3k**) and thiophene analogues of Si-rhodamine^15^ such as **3c** remain zwitterionic (**II**) across the entire pH 1–10 range. However, compound **3m** undergoes attack by the nucleophilic solvent (water) and is only present in its colored and NIR-fluorescent form **II** between pH 1 and 2.

**Table 1:**
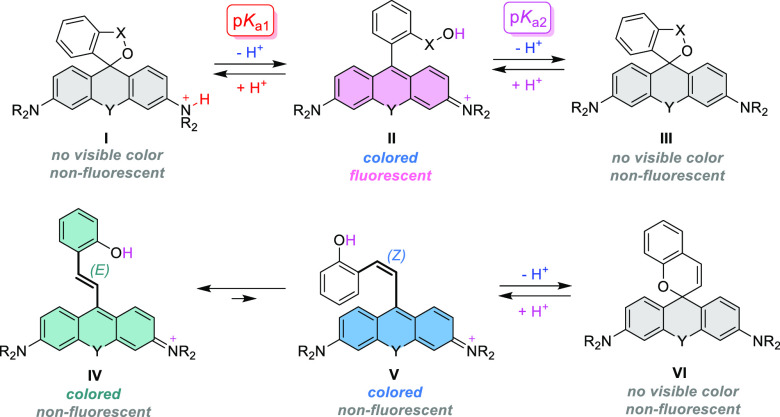
Properties of the Selected Fluorophores **3** and **5**^a^

compd	λ_max_(abs/fluor), nm	p*K*_a1_, p*K*_a2_^b^	*D*_0.5_^c^, *K*_L-Z_
**3c**	646/674	open	8, 5.0
**3f**	658/682	1.2, −	38, 0.36
**3j**	656/676	1.3, 2.3	48, 0.0041
**3k**	558/584	open	20, 20
**3l**	620/646	1.6, −	42, 0.17
**3m**	708/738	<1,^d^ –	39, 0.016
**5a**	650/674	2.0, 4.2	41, 0
**5b**	652/676	1.4, 5.1	36, 0
**5d**	650/647	2.6, 4.0	–
**5e**	658/678	1.9, 4.4	42, 0
**5f**	658/678	1.2, 2.9	41, 0
**5g**	664/688	1.7, 4.7	41, 0
**5h**	658/680	1.8, 4.5	47, 0
**5i**	656/–	2.1, 4.0	–
**5j**	642/–	2.2, 4.0	–
**5k**	658/–	1.7, 3.5	–
**5m**	658/–	1.7, 3.6	–
**5n**	650/674	1.3, 2.2	–

a See Figures S1 and S2 in the Supporting Information for the corresponding spectra.

b Apparent p*K*_a_ values in 20% (v/v) DMSO–100 mM Na phosphate buffer.

c With 1% (v/v) DMSO.

d **3m** forms a colorless water adduct at pH > 1.5.

On the contrary, spiroether analogues and homologues **5a**–**h** of the HMSiR dye^5c,13^ demonstrate the second distinct transition (**II** ↔ **III**, corresponding to p*K*_a2_) within the biologically relevant pH 4–10 range due to higher nucleophilicity of the reversibly formed alkoxide as compared to the carboxylate group. Similar behavior of the spirochromenes **5i**–**m** is further complicated with the reversible *E*/*Z*-isomerization (**IV** ↔ **V**) of the xanthylium form of these dyes observed upon the protonation of colorless **VI** (see Figure S3 in the Supporting Information). Because of free rotation of the styrene fragment, no far-red fluorescence emission characteristic of Si-pyronins is observed in solutions containing the protonated forms **IV** and **V** of the compounds **5i**–**m**. Rigidizing the double bond in the (*Z*)-configuration (**V**) within the cyclopentene context of **5n** results in recovery of Si-pyronin fluorescence upon its protonation.

As described previously,^4c,6a,15^ the position of the zwitterion–spirolactone (**II** ↔ **III**) equilibrium of the rhodamine dyes strongly depends on the nature of the solvent and increases with the increasing content of water in aprotic solvents such as 1,4-dioxane. We have measured these changes in absorbance and fluorescence (see Figure S2 the Supporting Information) according to the two proposed metrics: *D*_0.5_ (dielectric constant of a dioxane–water mixture at which the normalized extinction ε/ε_max_ of the dye is equal to one-half of the maximal value observed across the entire dioxane–water gradient,^15^ range 2 to 78) and *K*_L-Z_ (the spirolactone–zwitterion equilibrium constant in 1:1 dioxane–water,^16^ range 0 to +∞). We conclude that only the *D*_0.5_ metric is appropriate for evaluating the response of the position of this equilibrium to solvent polarity for both spirolactones **3** and spiroethers **5**, since the range of the *K*_L-Z_ metric is compressed between 0 and 1 for the compounds existing predominantly in the colorless closed forms (**III**, **VI**), resulting in zero measured values for all spiroethers. According to the data based on *D*_0.5_ (Table 1), the response of the spiroether fluorophores **5b**,**e-h** to the changes in solvent polarity is similar to that of HMSiR (**5a**) and carbo- and Si-rhodamines,^15^ while the spiroethers derived from dihydrocoumarin or coumarin do not undergo ring opening (**VI** → **IV**, **V**) under neutral conditions.

In summary, we have proposed a unified synthetic approach toward diverse analogues of cell-permeant, spontaneously blinking, and photoactivatable versions of the silicon-rhodamine fluorophore, and we investigated their behavior in aqueous solutions at different pH values and with varying contents of organic cosolvent (dioxane). We are currently applying these insights into the behavior of spirocyclic fluorophores **3** and **5** to the design and development of switchable fluorescent probes for biologically relevant superresolution microscopy.

## Supporting Information Available

Synthetic procedures and characterizations of the compounds, UV–vis spectra (Figures S1 and S2), and copies of the NMR spectra (PDF)
