# Supplementary material for: Modular Synthetic Approach to Silicon-Rhodamine Homologues and Analogues via Bis-aryllanthanum Reagents
Source: Org Lett. 2021 Mar 15;23(7):2604–9. doi: 10.1021/acs.orglett.1c00512 (PMC8041385; doi:10.1021/acs.orglett.1c00512)
Supplement: Supplementary file 1 — ol1c00512_si_001.pdf [file ol1c00512_si_001.pdf]

Supplementary information

Modular Synthetic Approach to Silicon-Rhodamine Homologues and Analogues via Bis-aryllanthanum Reagents

Alexey N. Butkevich[a*]

[a] Department of Optical Nanoscopy, Max Planck Institute for Medical Research, Jahnstrasse 29, 69120 Heidelberg, Germany

*Corresponding author's e-mail: alexey.butkevich@mr.mpg.de

Table of Contents

Supplementary Figures 4

Figure S1. Absorption and fluorescence emission spectra of selected compounds **3** and **5** in 100 mM phosphate buffer at different pH values (pH 1-9, +20 v/v% DMSO) 4

Figure S2. Absorption and fluorescence emission spectra of selected compounds **3** and **5** in dioxane-water mixtures (0…100% water content, +1 v/v% DMSO) 10

Figure S3. Protonation and 2*H*-chromene/2-(*E/Z*)-hydroxystyrene isomerization of the compound **5i** in 5% (v/v) TFA-*d* in methanol-d_4 (^{1}H NMR, 25 °C). 14

Supplementary Methods 15

General experimental information and synthesis....................15

Preparation of the starting materials....................18

S1 18

1c 18

S2 19

1d 20

4k 20

S3 21

4l 21

General synthetic procedure for compounds **3** and **5** 22

3a 22

3b 23

3c 23

3d 24

3e 24

3f 25

3g 25

3h 26

3i 26

3j 27

3k 27

3l 28

3m 28

3n 29
3o 30
5a 30
5b 31
5c 31
5d 32
5e 32
5f 33
5g 33
5h 34
5i 34
5j 35
5k 35
5l 36
5m 36
5n 37
General synthetic procedure for the preparation of live-cell dyes **6a-f** by carbonylative hydroxylation 37
4-TMR-CO_2H (**6a**) 38
4-610CP-CO_2H (**6b**) 38
4-SiR-CO_2H (**6c**) 39
4-SiR700-CO_2H (**6d**) 39
5-HMSiR-CO_2H (**6e**) 40
6f 40
Synthesis of live-cell fluorescent tubulin probe 4-SiR-CTX (**8**) by carbonylative amination 41
7a and **7b** 41
4-SiR-CTX (**8**) 42
Supplementary references 44
NMR spectra 46

Supplementary Figures

Figure S1. Absorption and fluorescence emission spectra of selected compounds **3** and **5** in 100 mM phosphate buffer at different pH values (pH 1-9, +20 v/v% DMSO).

3c (absorption)

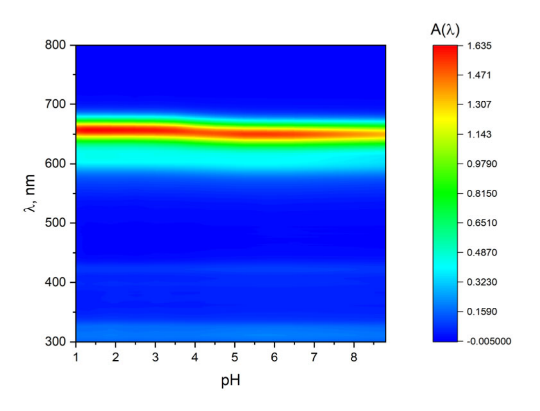

3c (emission)

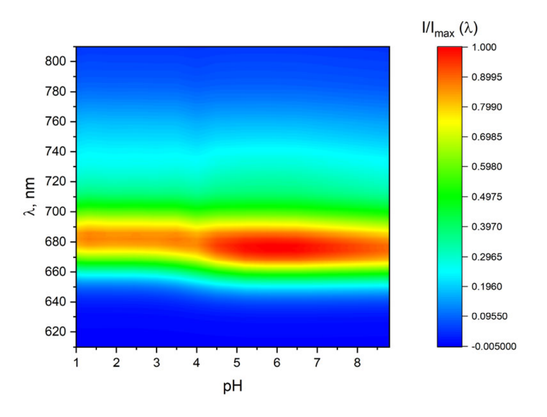

3f (absorption)

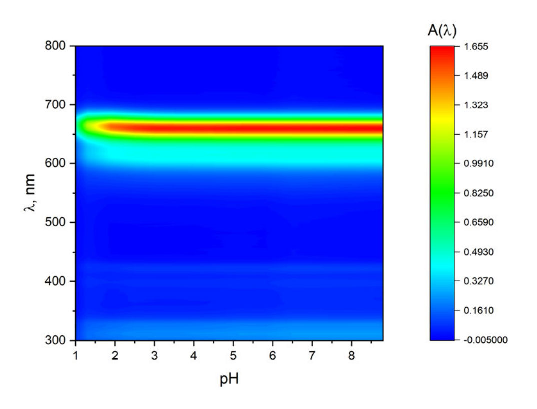

3f (emission)

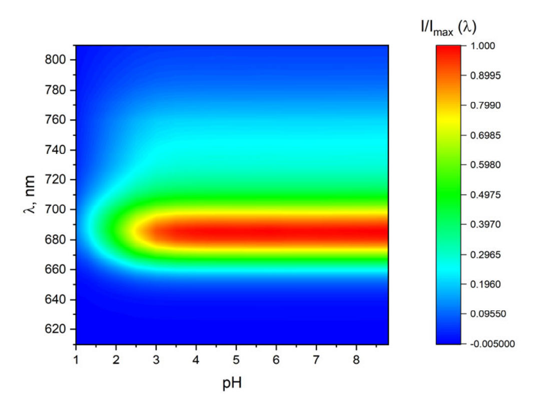

3j (absorption)

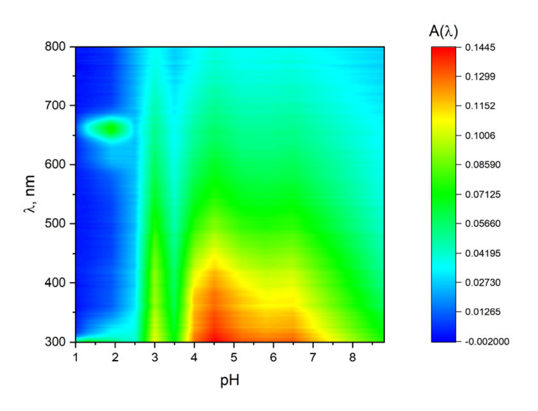

3j (emission)

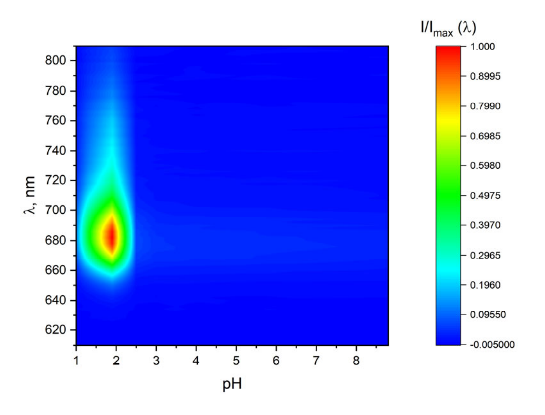

(continued)

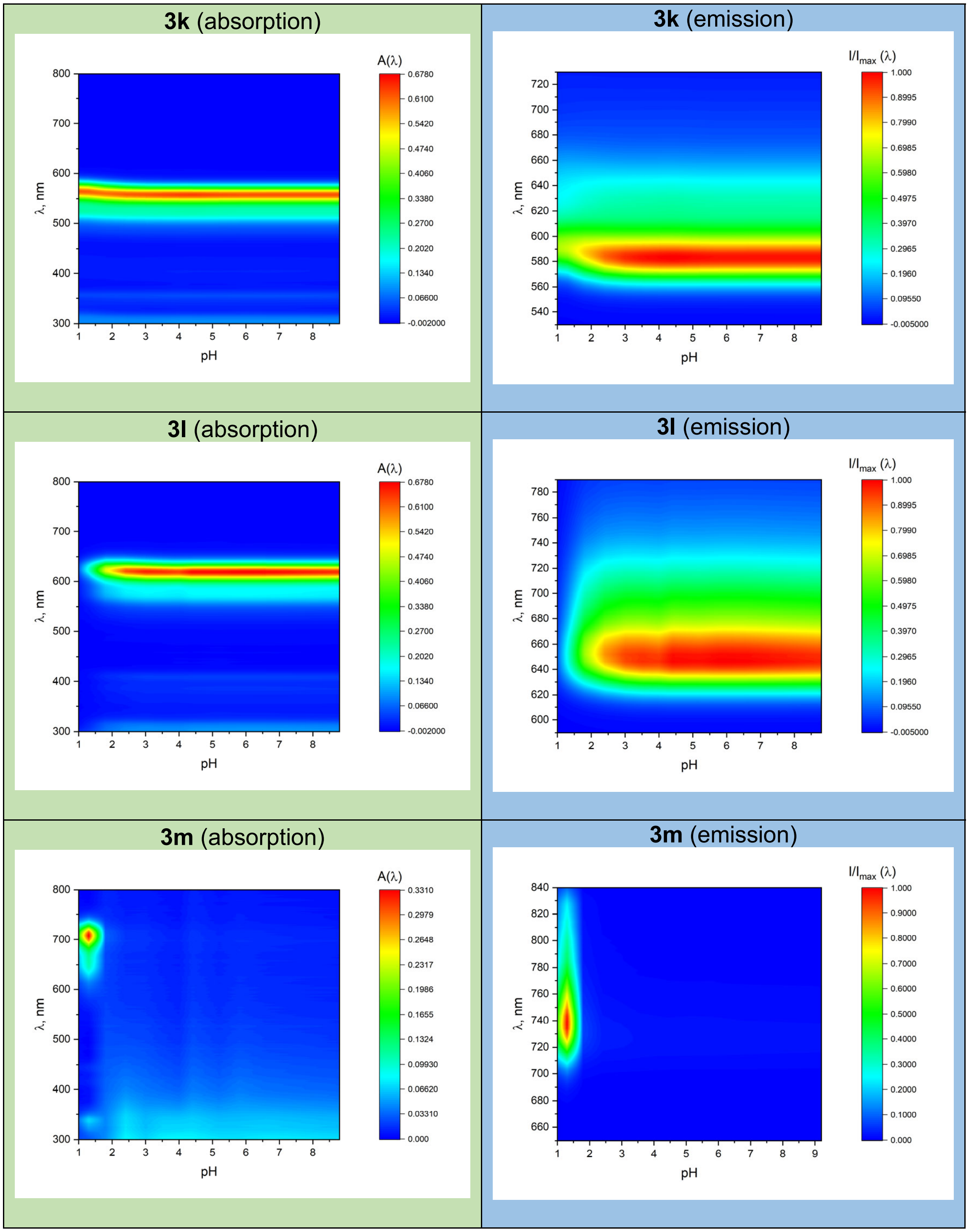
3k (absorption)
3k (emission)
3l (absorption)
3l (emission)
3m (absorption)
3m (emission)
A(λ)
I/Imax (λ)
λ, nm
pH

(continued)

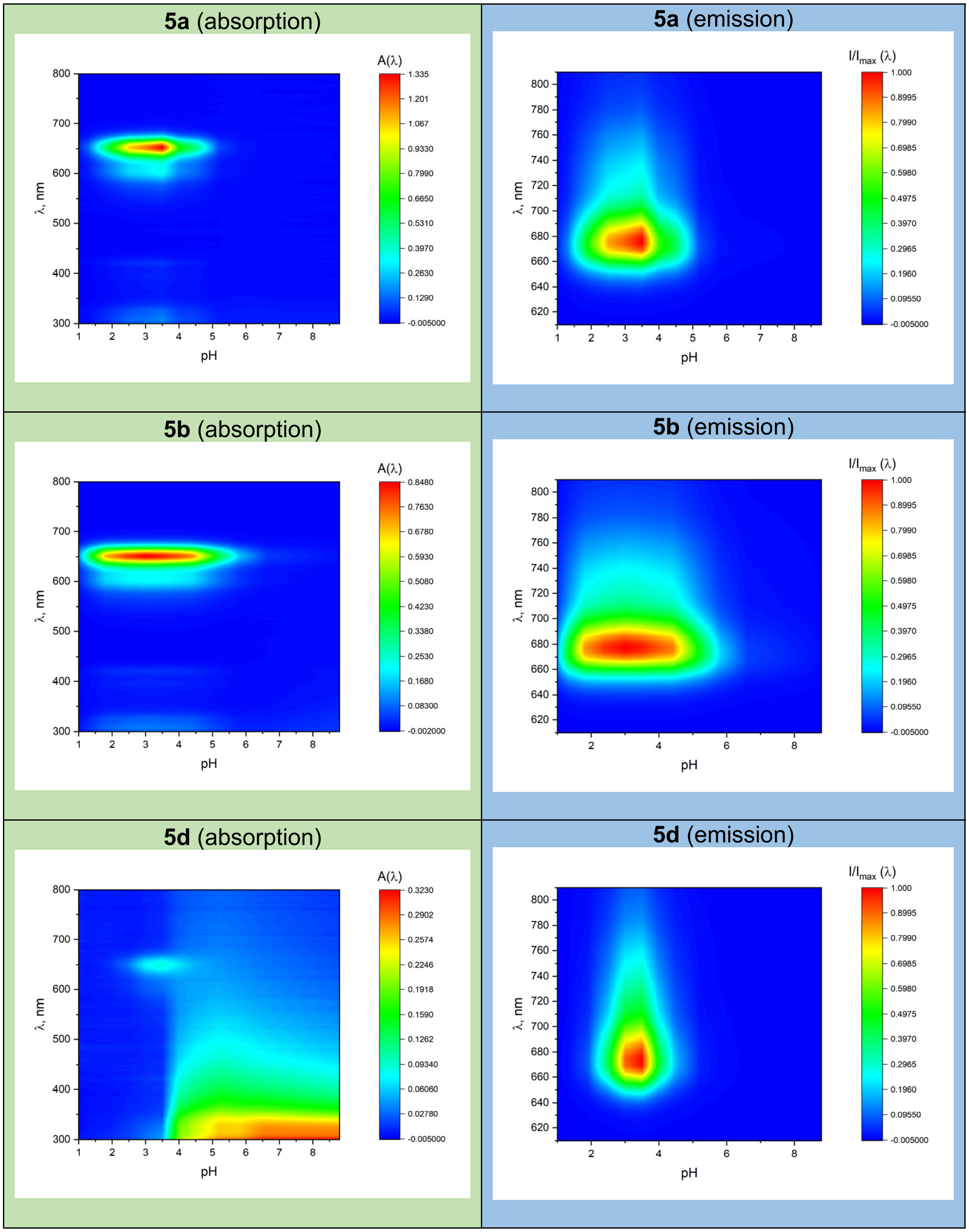
5a (absorption)
A(λ)
λ, nm
pH
5a (emission)
I/Imax (λ)
λ, nm
pH
5b (absorption)
A(λ)
λ, nm
pH
5b (emission)
I/Imax (λ)
λ, nm
pH
5d (absorption)
A(λ)
λ, nm
pH
5d (emission)
I/Imax (λ)
λ, nm
pH

(continued)

5e (absorption)

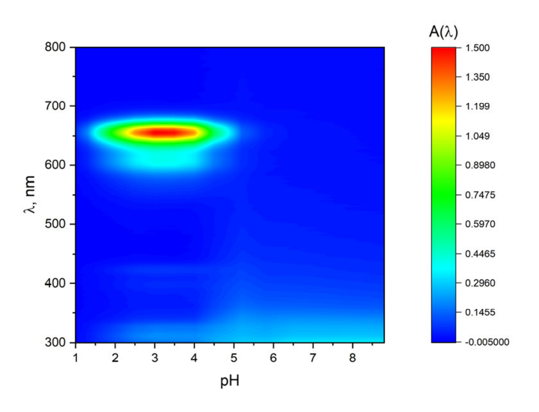

5e (emission)

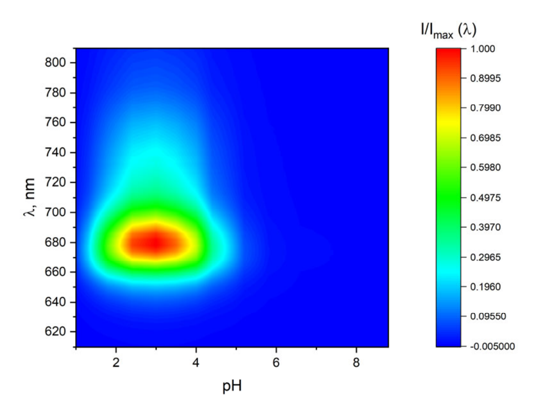

5f (absorption)

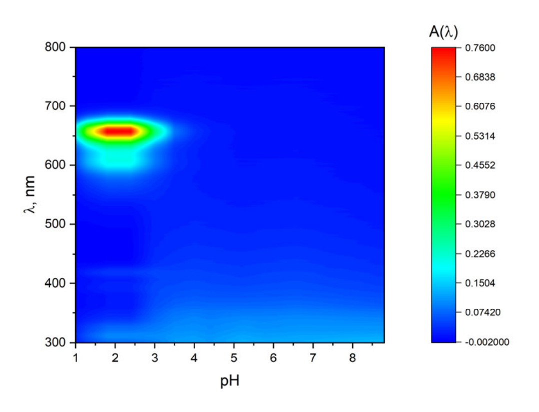

5f (emission)

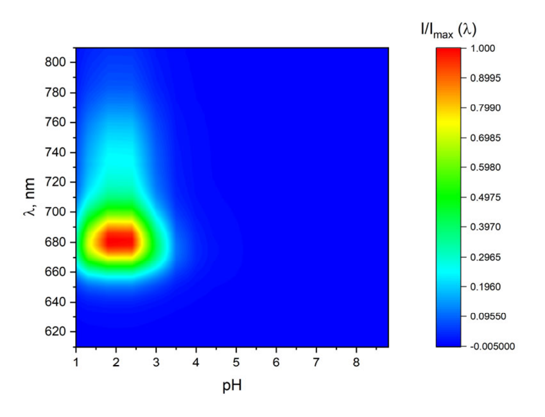

5g (absorption)

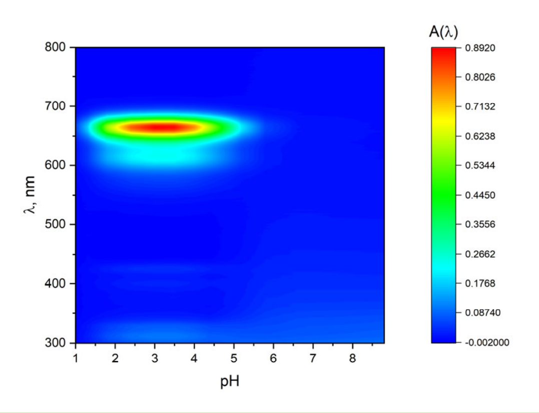

5g (emission)

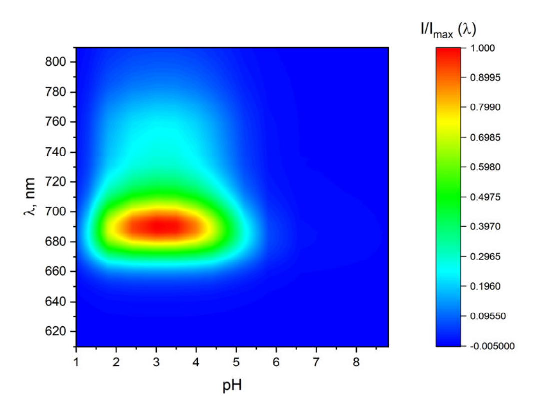

(continued)

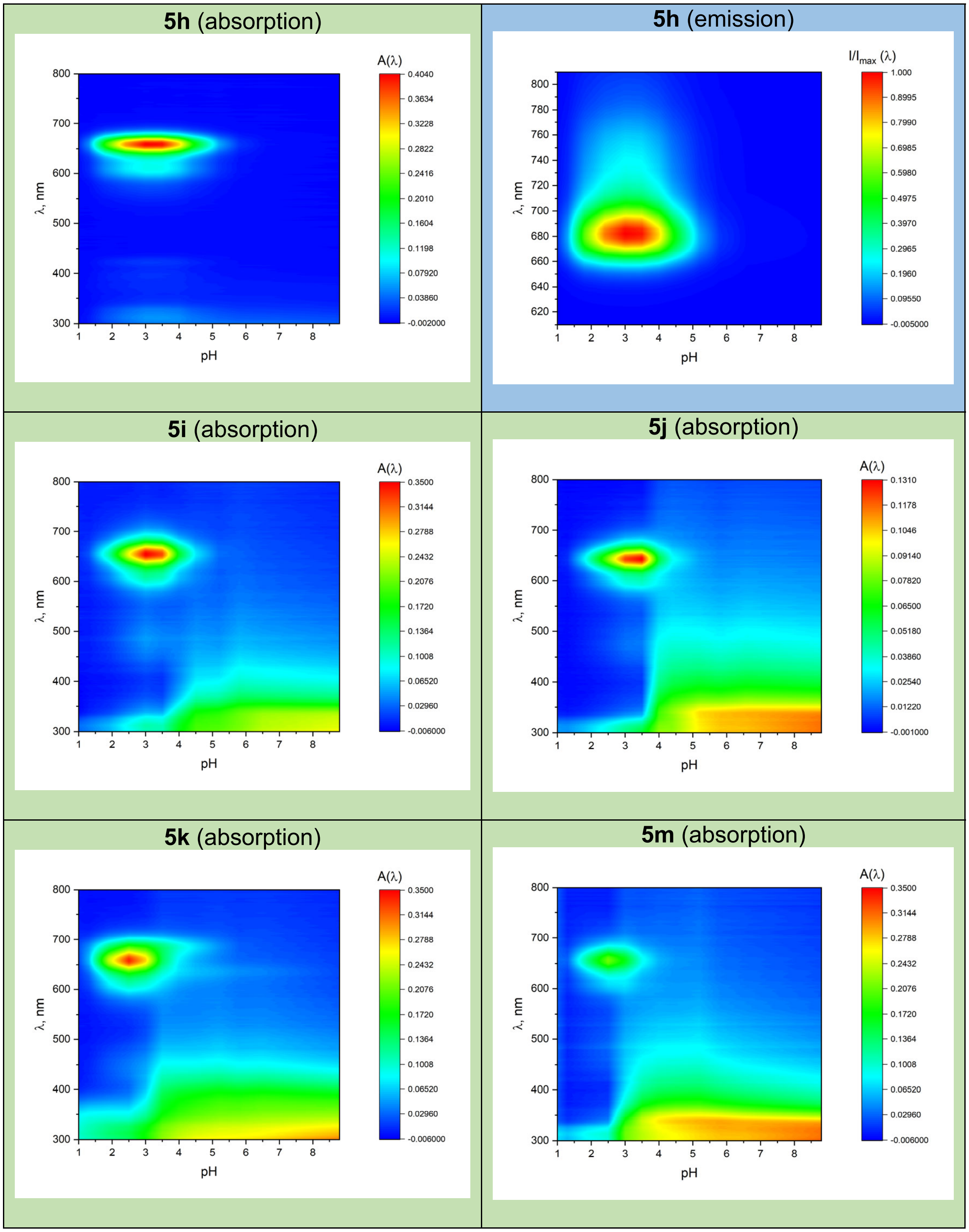
5h (absorption)
A(λ)
0.4040
0.3634
0.3228
0.2822
0.2416
0.2010
0.1604
0.1198
0.07920
0.03860
-0.002000
λ, nm
pH
5h (emission)
I/Imax (λ)
1.000
0.8995
0.7990
0.6985
0.5980
0.4975
0.3970
0.2965
0.1960
0.09550
-0.005000
λ, nm
pH
5i (absorption)
A(λ)
0.3500
0.3144
0.2788
0.2432
0.2076
0.1720
0.1364
0.1008
0.06520
0.02960
-0.006000
λ, nm
pH
5j (absorption)
A(λ)
0.1310
0.1178
0.1046
0.09140
0.07820
0.06500
0.05180
0.03860
0.02540
0.01220
-0.001000
λ, nm
pH
5k (absorption)
A(λ)
0.3500
0.3144
0.2788
0.2432
0.2076
0.1720
0.1364
0.1008
0.06520
0.02960
-0.006000
λ, nm
pH
5m (absorption)
A(λ)
0.3500
0.3144
0.2788
0.2432
0.2076
0.1720
0.1364
0.1008
0.06520
0.02960
-0.006000
λ, nm
pH

(continued)

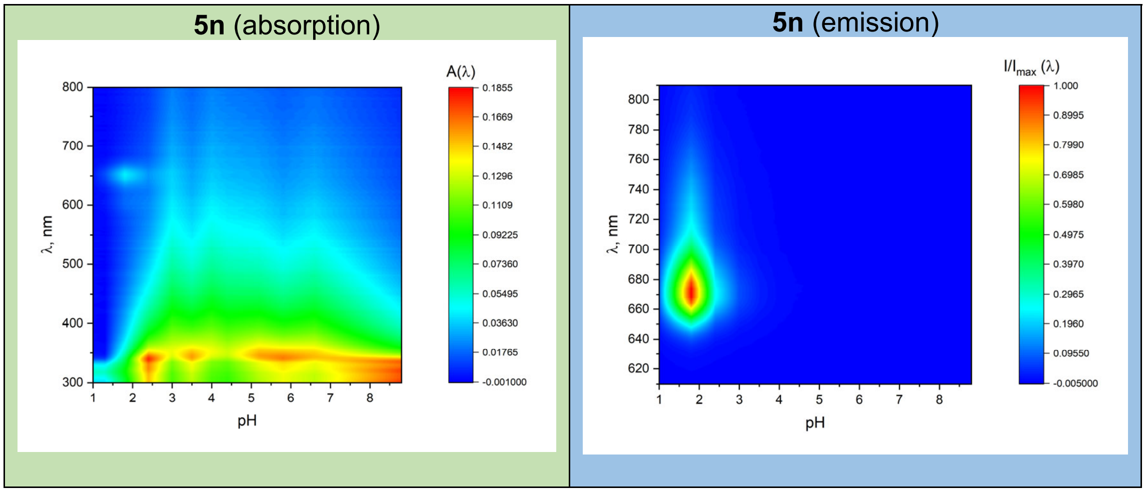
5n (absorption)
A(λ)
λ, nm
pH
0.1855
0.1669
0.1482
0.1296
0.1109
0.09225
0.07360
0.05495
0.03630
0.01765
-0.001000
5n (emission)
I/Imax (λ)
λ, nm
pH
1.000
0.8995
0.7990
0.6985
0.5980
0.4975
0.3970
0.2965
0.1960
0.09550
-0.005000

Figure S2. Absorption and fluorescence emission spectra of selected compounds **3** and **5** in dioxane-water mixtures (0…100% water content, +1 v/v% DMSO).

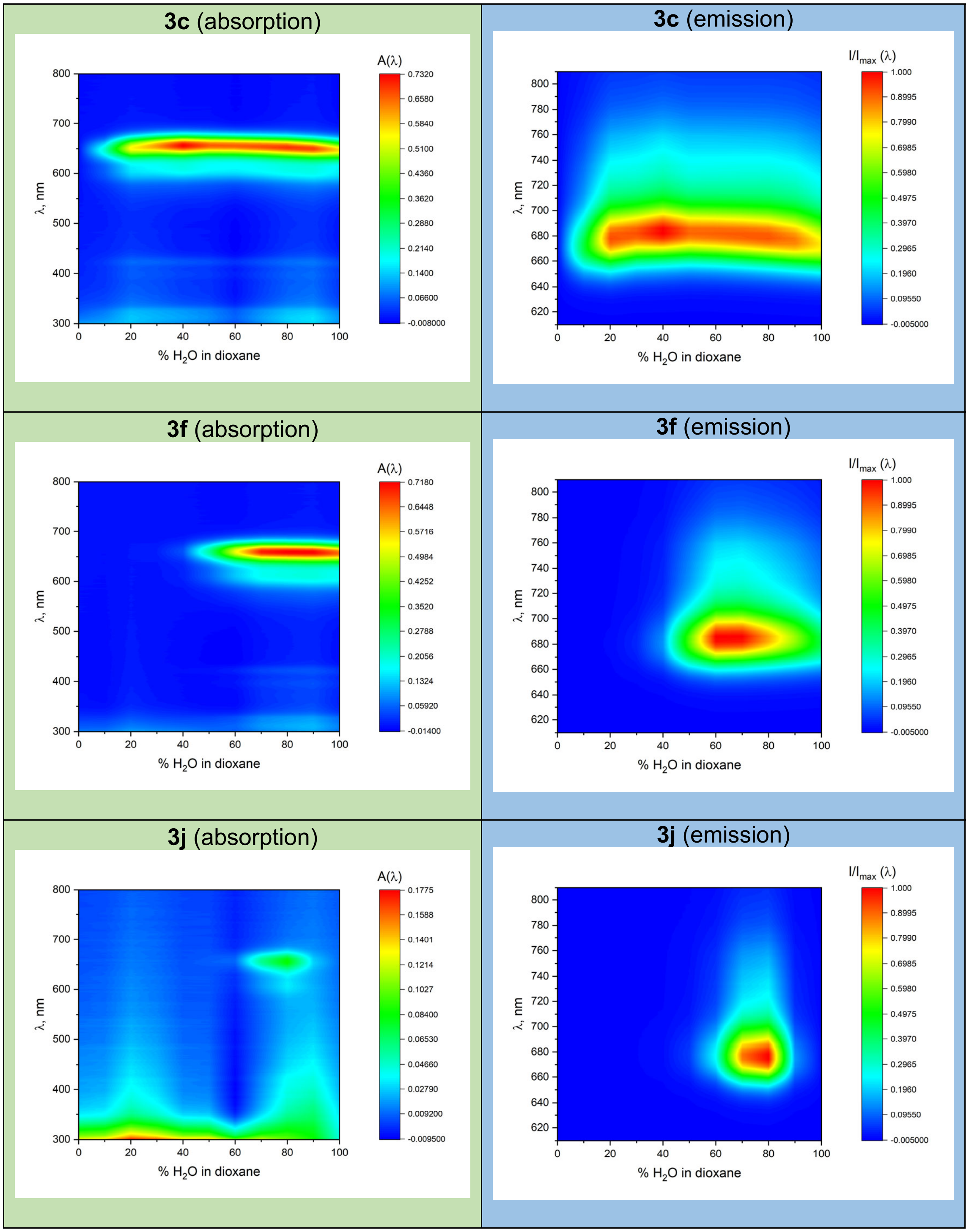

(continued)

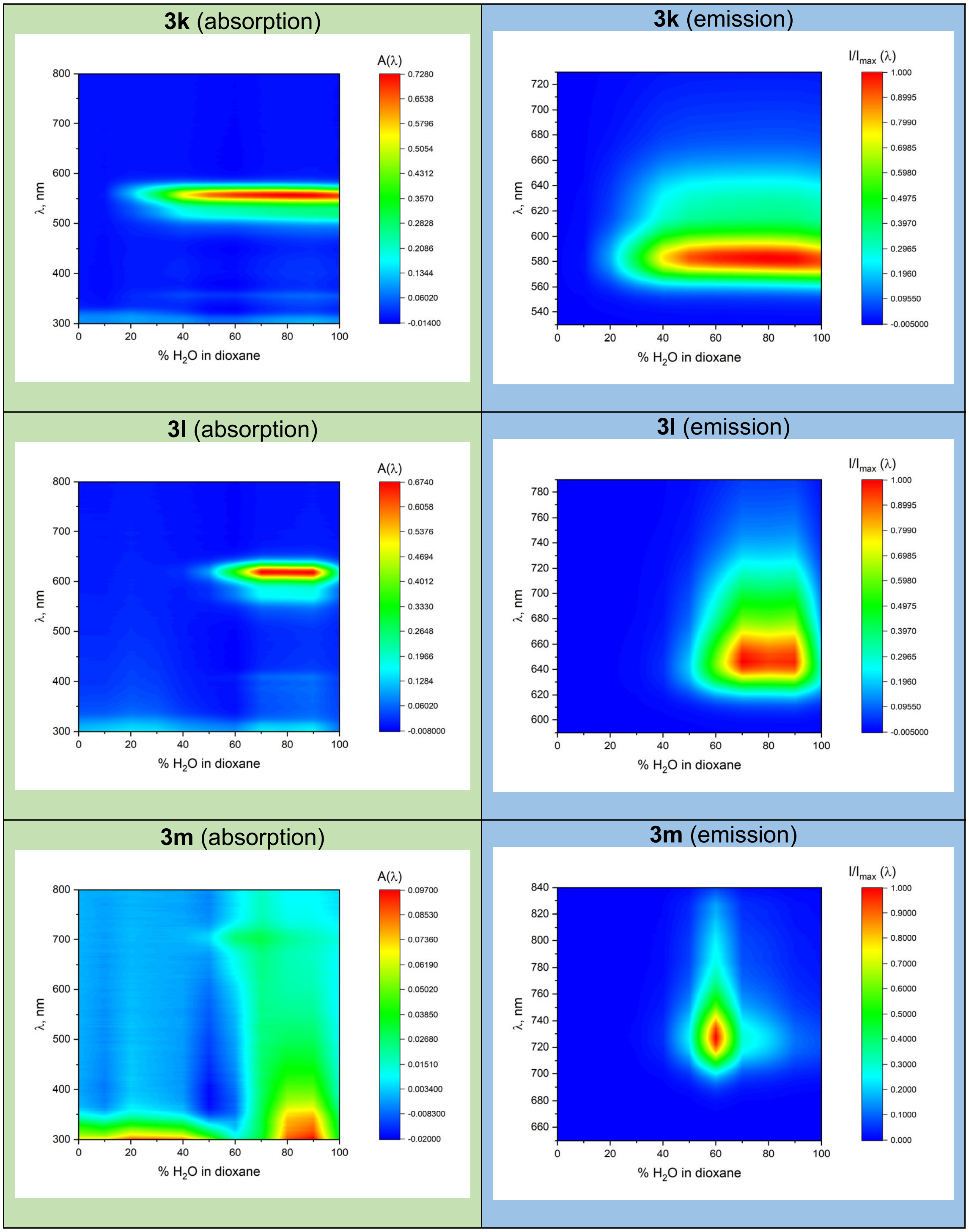

3k (absorption)
3k (emission)
3l (absorption)
3l (emission)
3m (absorption)
3m (emission)
A(λ)
I/I_{max} (λ)
λ, nm
% H_2O in dioxane
0.7280
0.6538
0.5796
0.5054
0.4312
0.3570
0.2828
0.2086
0.1344
0.06020
-0.01400
1.000
0.8995
0.7990
0.6985
0.5980
0.4975
0.3970
0.2965
0.1960
0.09550
-0.005000
0.6740
0.6058
0.5376
0.4694
0.4012
0.3330
0.2648
0.1966
0.1284
0.06020
-0.008000
0.09700
0.08530
0.07360
0.06190
0.05020
0.03850
0.02680
0.01510
0.003400
-0.008300
-0.02000
0.9000
0.8000
0.7000
0.6000
0.5000
0.4000
0.3000
0.2000
0.1000
0.000

(continued)

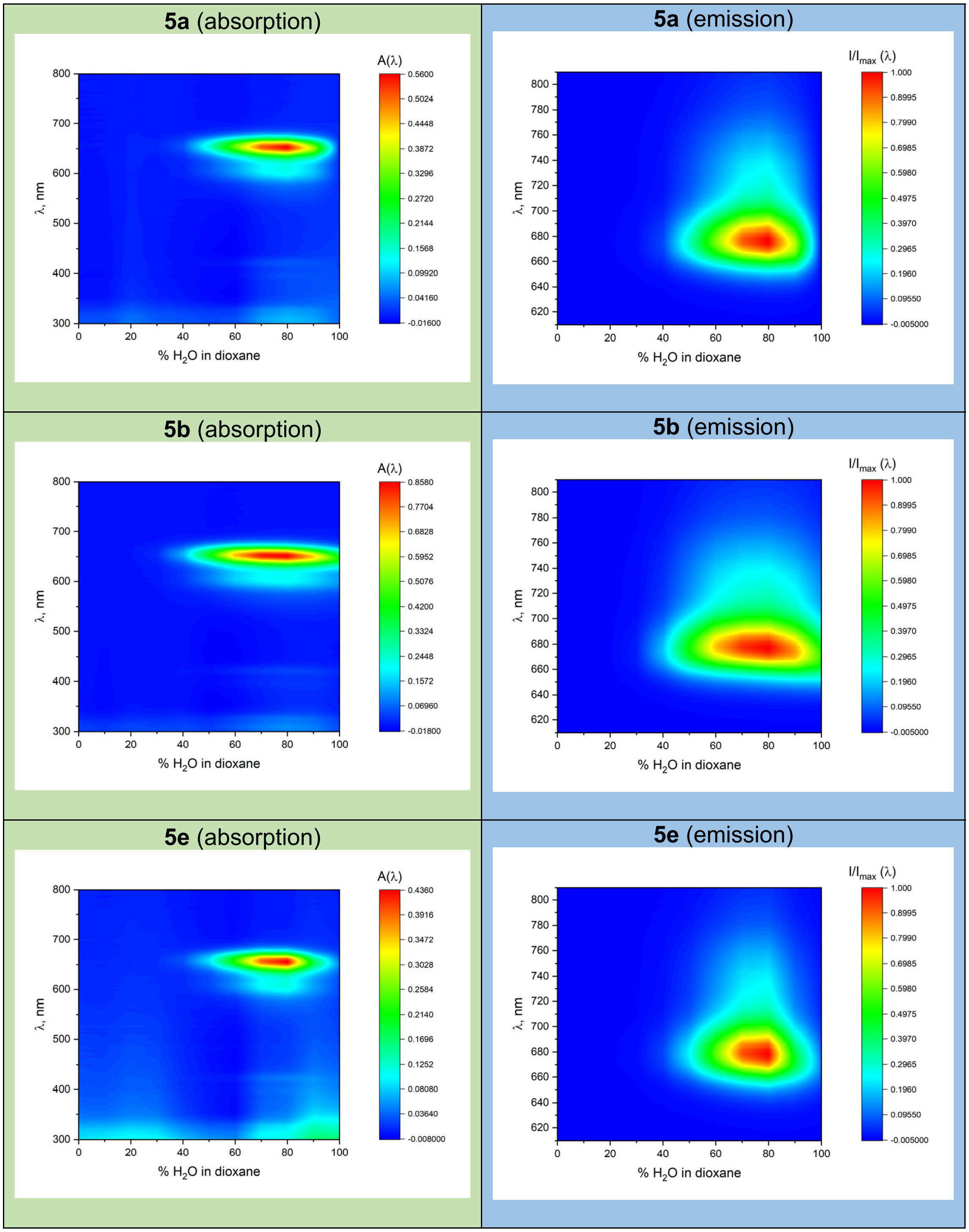

5a (absorption)
A(λ)
0.5600
0.5024
0.4448
0.3872
0.3296
0.2720
0.2144
0.1568
0.09920
0.04160
-0.01600
λ, nm
% H2O in dioxane
5a (emission)
I/Imax (λ)
1.000
0.8995
0.7990
0.6985
0.5980
0.4975
0.3970
0.2965
0.1960
0.09550
-0.005000
λ, nm
% H2O in dioxane
5b (absorption)
A(λ)
0.8580
0.7704
0.6828
0.5952
0.5076
0.4200
0.3324
0.2448
0.1572
0.06960
-0.01800
λ, nm
% H2O in dioxane
5b (emission)
I/Imax (λ)
1.000
0.8995
0.7990
0.6985
0.5980
0.4975
0.3970
0.2965
0.1960
0.09550
-0.005000
λ, nm
% H2O in dioxane
5e (absorption)
A(λ)
0.4360
0.3916
0.3472
0.3028
0.2584
0.2140
0.1696
0.1252
0.08080
0.03640
-0.008000
λ, nm
% H2O in dioxane
5e (emission)
I/Imax (λ)
1.000
0.8995
0.7990
0.6985
0.5980
0.4975
0.3970
0.2965
0.1960
0.09550
-0.005000
λ, nm
% H2O in dioxane

(continued)

5f (absorption)

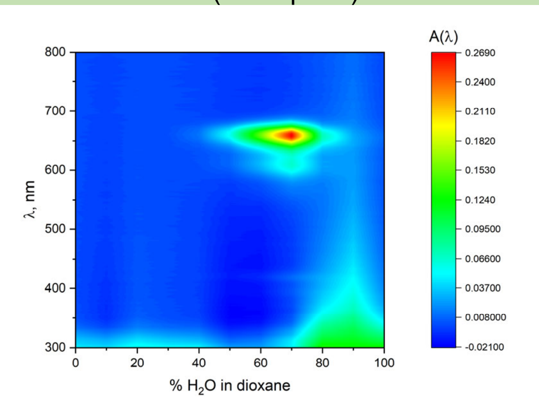

5f (emission)

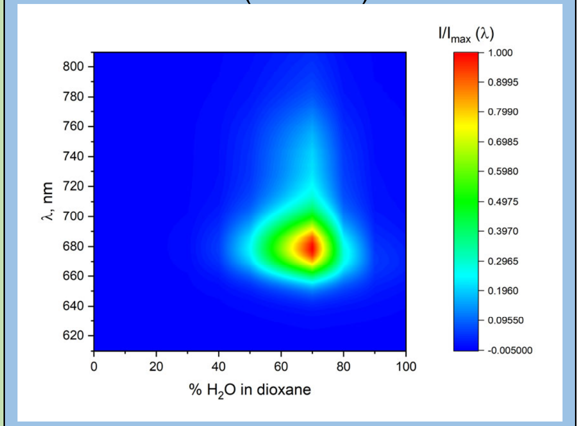

5g (absorption)

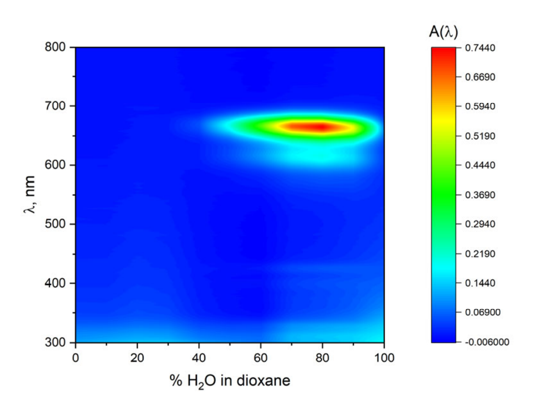

5g (emission)

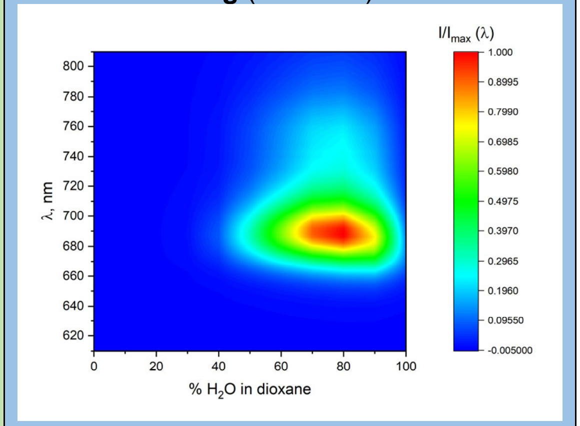

5h (absorption)

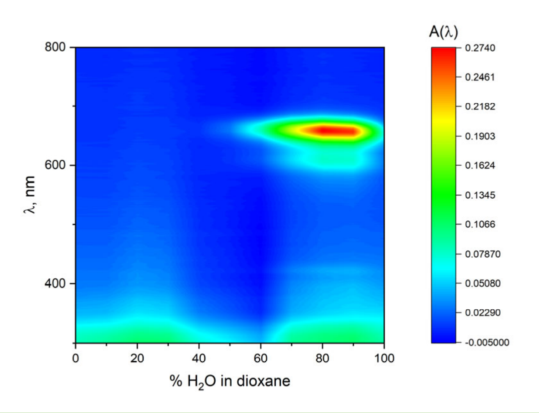

5h (emission)

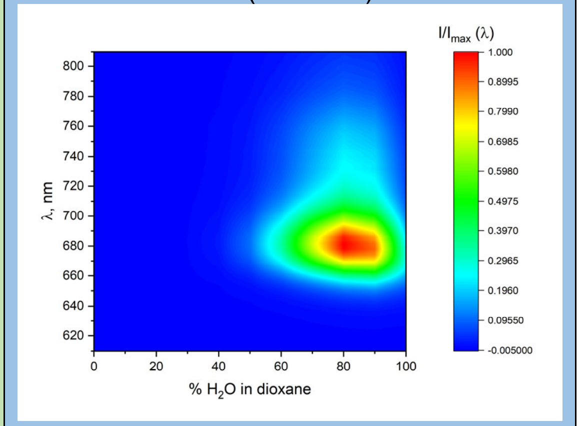

Figure S3. Protonation and 2*H*-chromene/2-(*E*/*Z*)-hydroxystyrene isomerization of the compound **5i** in 5% (v/v) TFA-*d* in methanol-d_4 (^{1}H NMR, 25 °C).

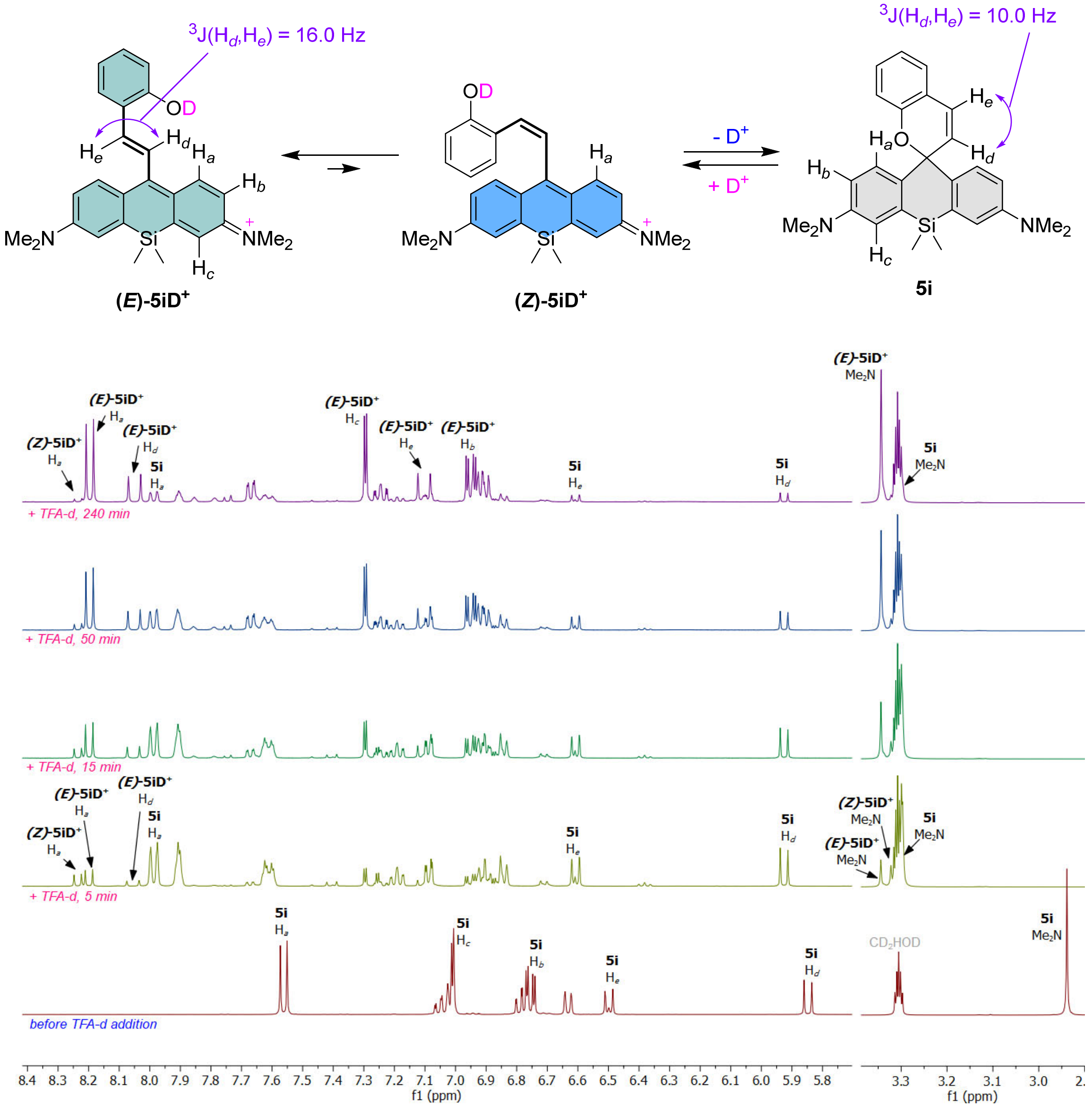

Supplementary Methods

General experimental information and synthesis

Thin layer chromatography

Analytical TLC (normal phase) was performed on Merck Millipore ready-to-use aluminum sheets coated with silica gel 60 (F_{254}) (Cat. No. 1.05554.0001). Analytical TLC on reversed phase (RP-C_{18}) was performed on Merck Millipore ready-to-use aluminum sheets coated with RP-18 60 (F_{254}s) (Cat. No. 1.05560.0001). Compounds were detected by exposing TLC plates to UV-light (254 or 366 nm) or by heating with vanillin stain (6 g vanillin and 1.5 mL conc. H_2SO_4 in 100 mL ethanol), 1 N NaOH or 1 N HCl as indicated.

Preparative flash column chromatography

Automated separations on normal phase were performed with an Isolera Spektra One system (Biotage AG, Sweden) using commercially available cartridges of suitable size (RediSep Rf series from Teledyne ISCO, Puriflash Silica HP 30µm series from Interchim) and solvent gradient indicated. Automated separations on reversed phase (C18, Amino) were performed on the cartridge flow path of a Reveleris Prep system (Büchi Labortechnik AG, Switzerland) using the type of cartridge and solvent gradient as indicated.

High-Performance Liquid Chromatography (HPLC) and Mass Spectrometry (MS)

Analytical liquid chromatography-mass spectrometry was performed on an LC-MS system (Shimadzu): 2x LC-20AD HPLC pumps with DGU-20A3R solvent degassing unit, SIL-20ACHT autosampler, CTO-20AC column oven, SPD-M30A diode array detector and CBM-20A communication bus module, integrated with CAMAG TLC-MS interface 2, FCV-20AH_2 diverter valve and LCMS-2020 spectrometer with electrospray ionization (ESI, 100 – 1500 m/z). Analytical column: Hypersil GOLD 50×2.1 mm 1.9µm, standard conditions: sample volume 1-2 µL, solvent flow rate 0.5 mL/min, column temperature 30 °C. General method: isocratic 90:10 A:B over 2 min, then gradient 90:10 to 1:99 A:B over 5 min, then isocratic 1:99 A:B over 2 min; solvent A – water + 0.1% (v/v) HCO_2H, solvent B – acetonitrile + 0.1% (v/v) HCO_2H.

High resolution mass spectra (HRMS) were obtained on a maXis II ETD (Bruker) with electrospray ionization (ESI) at the Mass Spectrometry Core facility of the Max-Planck Institute for Medical Research (Heidelberg, Germany).

Preparative high-performance liquid chromatography was performed on a Büchi Reveleris Prep system using the suitable preparative columns and conditions as indicated for individual preparations. Method scouting was performed on a HPLC system (Shimadzu): 2x LC-20AD HPLC pumps with DGU-20A3R solvent degassing unit, CTO-20AC column oven equipped with a manual injector with a 20 µL sample loop, SPD-M20A diode array detector, RF-20A fluorescence detector and CBM-20A communication bus module; or on a Dionex Ultimate 3000 UPLC system: LPG-3400SD pump, WPS-3000SL autosampler, TCC-3000SD column compartment with 2× 7-port 6-position valves and DAD-3000RS diode array detector. The test runs were performed on analytical columns with matching phases (HPLC: Interchim 250×4.6 mm 10 µm C18HQ, Interchim 250×4.6 mm 5 µm PhC4, solvent flow rate 1.2 mL/min; UPLC: Interchim C18HQ or PhC4 75×2.1 mm 2.2 µm, ThermoFisher Hypersil GOLD 100×2.1 mm 1.9 µm, solvent flow rate 0.5 mL/min).

Optical spectroscopy

Absorption and fluorescence emission spectra were recorded in triplicate with a CLARIOstar Plus microplate reader (BMG LABTECH GmbH, Germany) in 96-well microplates (200 µL/well): non-binding polystyrene F-bottom, µClear (Greiner Bio-One GmbH, Ref. 655906) for aqueous solutions, polypropylene F-bottom (Greiner Bio-One GmbH, Ref. 655201) for dioxane-water mixtures. The spectra were recorded at 25 °C in air-saturated solvents and are background corrected.

pK_a values (apparent) were determined from the absorbance data measured at the longest wavelength absorption maxima $A(\lambda_{max})$ within the visible range (400…700 nm) in the presence of 20% (v/v) DMSO to ensure sample solubility and fitted to the equation:

$$A(\lambda_{max}) = \frac{1}{1 + 10^{n(pK_a - pH)}}$$

for cases with one K_a, or

$$A(\lambda_{max}) = \frac{1}{1 + 10^{n_1(pK_{a1} - pH)}} + \frac{1}{1 + 10^{n_2(pK_{a2} - pH)}} - 1$$

for cases with two K_a's (K_{a1} and K_{a2}), using the Origin 2020b software.

$D_{0.5}$ values [1] were determined from the absorbance data measured at the longest wavelength absorption maxima $A(\lambda_{max})$ within the visible range (400…700 nm) in dioxane-water mixtures containing from 0% to 100% water in 10% steps and fitted to the following equation:

$$A(\lambda_{max}) = \frac{1}{1 + 10^{n(D_{0.5}-D)}}$$

where D is the dielectric constant of the corresponding dioxane-water mixture [2].

$K_{L\text{-}Z}$ values [3] were calculated according to the equation:

$$K_{L-Z} = \frac{\varepsilon_{dw}/\varepsilon_{max}}{1 - \varepsilon_{dw}/\varepsilon_{max}} = \frac{A_{dw}/A_{max}}{1 - A_{dw}/A_{max}}$$

where A_{dw} and A_{max} – absorptions of the solutions with equal concentration of a dye in dioxane-water (1:1) + 0.01% Et_3N (for A_{dw}) or in 0.1% TFA-EtOH (for A_{max}), measured at the longest wavelength absorption maxima λ_{max}. The corresponding absorption spectra were recorded with a Varian Cary 4000 UV-Vis spectrophotometer (Agilent).

NMR spectra

NMR spectra were recorded at 25 °C with a Bruker Ascend 400 spectrometer at 400.15 MHz (^{1}H), 376.52 MHz (^{19}F) and 100.62 MHz (^{13}C) and are reported in ppm. All ^{1}H spectra are referenced to tetramethylsilane as an internal standard (δ = 0.00 ppm). ^{13}C spectra are referenced to tetramethylsilane (δ = 0 ppm) using the signals of the solvent: $CDCl_3$ (77.16 ppm), CD_3OD (49.00 ppm), DMSO-d_6 (39.52 ppm) or C-2,6 (150.35 ppm) of pyridine-d_5. Multiplicities of the signals are described as follows: s = singlet, d = doublet, t = triplet, q = quartet, m = multiplet or overlap of non-equivalent resonances; br = broad signal. Coupling constants $^nJ_{X\text{-}Y}$ are given in Hz, where n is the number of bonds between the coupled nuclei X and Y ($J_{H\text{-}H}$ are always listed as J without indices).

Preparation of the starting materials

S1

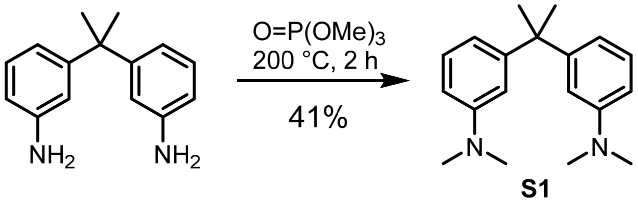

The procedure from [4] was followed. A solution of 2,2-bis(3-aminophenyl)propane (252 mg, 1.12 mmol; prepared according to the literature procedure: see Example 6 in [5]) in trimethyl phosphate (275 µL, 2.35 mmol, 2.1 eq) in a 10 mL round-bottom flask (equipped with an air condenser and a drying tube) was heated up under argon at 200 °C (bath temperature) for 2 h. The reaction mixture was then allowed to cool below 100 °C, and 1 N NaOH (15 mL) was added. The resulting emulsion was stirred at rt overnight. The mixture was extracted with CH_2Cl_2 (3 × 20 mL), the combined extracts were washed with brine, dried over Na_2SO_4, the filtrate was evaporated on Celite and the product was isolated by flash column chromatography (12 g 30 µm Interchim Puriflash SiHP, gradient 5% to 40% EtOAc – hexane) to give 128 mg (41%) of the product as viscous colorless oil.

^{1}H NMR (400 MHz, $CDCl_3$): δ 7.13 (app.t, J = 7.9 Hz, 2H), 6.68 (dd, J = 2.5, 1.8 Hz, 2H), 6.62 (ddd, J = 7.7, 1.8, 0.9 Hz, 2H), 6.57 (ddd, J = 8.2, 2.5, 0.9 Hz, 2H), 2.89 (s, 12H), 1.67 (s, 6H).
^{13}C NMR (101 MHz, $CDCl_3$): δ 151.7, 150.4, 128.5, 116.1, 111.8, 110.1, 43.3, 40.9, 30.9.
HRMS (ESI) m/z: $[M+H]^+$ Calcd for $C_{19}H_{27}N_2$ 283.2169; Found 283.2165.

1c

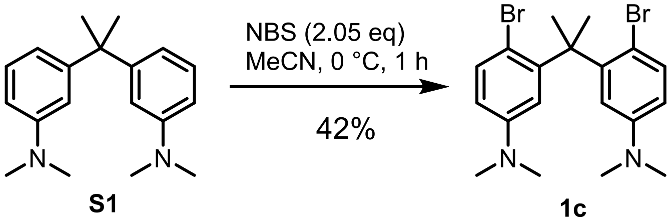

N-Bromosuccinimide (709 mg, 3.98 mmol, 2.05 eq) was added portionwise over 10 min to a solution of **S1** (548 mg, 1.94 mmol) in acetonitrile (15 mL), cooled in an ice-water bath. The reaction mixture was stirred at 0 °C for 1 h, diluted with aq. Na_2SO_3 solution (30 mL), extracted with CH_2Cl_2 (3 × 30 mL); the combined organic layers were washed with brine and dried over Na_2SO_4. The filtrate was evaporated on silica and the product was isolated by flash column chromatography (80 g Teledyne Isco RediSep Rf, gradient 20% to 100% CH_2Cl_2 – hexane, followed by 20% EtOAc – CH_2Cl_2). The combined fractions containing the product as major component were evaporated and recrystallized from EtOAc – hexane; the impure fractions were

pooled, and the purification was repeated. The combined yield was 356 mg (42%) of **1c** as white solid.

^{1}H NMR (400 MHz, $CDCl_3$): δ 7.26 (d, J = 8.8 Hz, 2H), 7.01 (d, J = 3.0 Hz, 2H), 6.46 (dd, J = 8.8, 3.0 Hz, 2H), 2.95 (s, 12H), 1.82 (s, 6H).
^{13}C NMR (101 MHz, $CDCl_3$): δ 150.0, 147.2, 135.1, 113.8, 112.3, 109.6, 46.4, 41.0, 29.4.
HRMS (ESI) m/z: $[M+2H]^{2+}$ Calcd for $C_{19}H_{26}Br_2N_2$ 221.0216; Found 221.0213.

S2

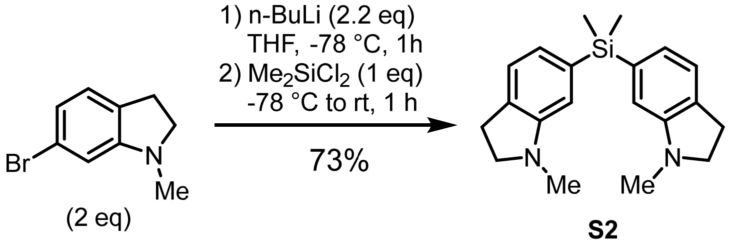

S2

In a 50 mL round bottom flask, *n*-BuLi (2 mL of 2.5 M solution in hexane, 5.2 mmol, 2.2 eq) was added dropwise to a solution of 6-bromo-1-methylindoline (1 g, 4.7 mmol, 2 eq; prepared according to literature procedure [6]) in anhydrous THF (20 mL), cooled to -78 °C (dry ice-acetone bath). After 1 h, dichlorodimethylsilane (0.29 mL, 2.4 mmol) was added dropwise to the resulting white suspension, which turned into a clear solution by the end of addition. The reaction mixture was allowed to warm up and stirred at rt for 1 h. It was then quenched by addition of water, poured into brine (50 mL) and extracted with EtOAc (3 × 30 mL). The combined extracts were dried over Na_2SO_4, and the product was isolated by flash column chromatography (40 g Teledyne Isco RediSep Rf, gradient 0% to 50% EtOAc:hexane) to give 560 mg (73%) of the product as yellowish oil.

^{1}H NMR (400 MHz, $CDCl_3$): δ 7.08 (d, *J* = 7.1 Hz, 2H), 6.86 (d, *J* = 7.1 Hz, 2H), 6.65 (s, 2H), 3.27 (t, *J* = 8.1 Hz, 3H), 2.93 (t, *J* = 8.1 Hz, 4H), 2.74 (s, 6H), 0.50 (s, 6H).
^{13}C NMR (101 MHz, $CDCl_3$): δ 152.8, 137.4, 131.8, 124.5, 124.0, 112.5, 56.1, 36.5, 28.9, -1.7.
HRMS (ESI) m/z: $[M+2H]^{2+}$ Calcd for $C_{20}H_{28}N_2Si$ 162.1005; Found 162.1005.

1d

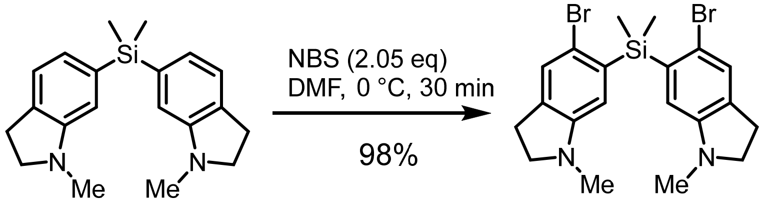

S2 **1d**

N-Bromosuccinimide (628 mg, 3.53 mmol, 2.05 eq) was added portionwise over 5 min to a solution of **S2** (555 mg, 1.72 mmol) in DMF (6 mL), cooled in an ice-water bath. The resulting suspension was stirred at 0 °C for 30 min, diluted with sat. aq. $NaHCO_3$ solution (50 mL), extracted with CH_2Cl_2 (3 × 25 mL); the combined organic layers were washed with water (2 × 50 mL), brine and dried over Na_2SO_4. The product was isolated by flash column chromatography (25 g 30 µm Interchim Puriflash SiHP, gradient 0% to 50% CH_2Cl_2 – hexane with 10% EtOAc constant additive) to give 805 mg (98%) of **1d** as white solid.

^{1}H NMR (400 MHz, $CDCl_3$): δ 7.20 (s, 2H), 6.57 (s, 2H), 3.30 (t, *J* = 8.2 Hz, 4H), 2.93 (t, *J* = 8.2 Hz, 4H), 2.70 (s, 6H), 0.71 (s, 6H).
^{13}C NMR (101 MHz, $CDCl_3$): δ 152.0, 137.1, 134.2, 128.8, 118.0, 115.3, 56.2, 36.3, 28.5, -0.6.
HRMS (ESI) m/z: $[M+H]^+$ Calcd for $C_{20}H_{25}Br_2N_2Si$ 481.0129; Found 481.0120.

4k

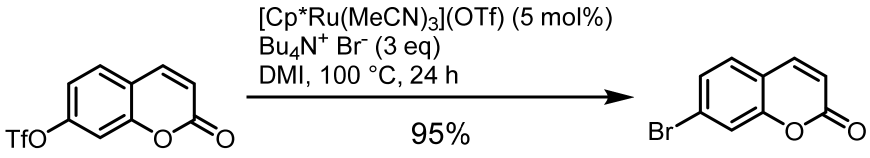

4j **4k**

In a 25 mL round bottom flask, a solution of 2-oxo-2*H*-chromen-7-yl trifluoromethanesulfonate (441 mg, 1.5 mmol, **4j**; prepared according to the literature procedure: compound **S21** in [7]), tetrabutylammonium bromide (1.45 g, 4.5 mmol, 3 eq) and tris(acetonitrile)pentamethylcyclopentadienylruthenium(II) trifluoromethanesulfonate (38 mg, 0.075 mmol, 5 mol%; CAS # 113860-02-9, Strem Chemicals) in anhydrous 1,3-dimethyl-2-imidazolidinone (DMI, 5 mL) was degassed and stirred under argon at 100 °C (bath temperature) for 24 h. Upon cooling, the mixture was diluted with EtOAc-hexane (1:1), poured into water (100 mL) and extracted with EtOAc-hexane (1:1, 3 × 30 mL). The combined extracts were washed with water (100 mL), brine and dried over Na_2SO_4. The filtrate was evaporated on Celite, and the product was isolated by flash column chromatography (25 g 30 µm Interchim Puriflash SiHP, gradient 10% to 70% EtOAc:hexane) to give 319 mg (95%) of **4k** as white solid. Known

compound: compound **2o** in [8] (mislabeled in the original publication as “7-bromo-2*H*-chromene”).

^{1}H NMR (400 MHz, $CDCl_3$): δ 7.66 (d, *J* = 9.6 Hz, 1H), 7.52 (d, *J* = 1.8 Hz, 1H), 7.42 (dd, *J* = 8.3, 1.8 Hz, 1H), 7.35 (d, *J* = 8.3 Hz, 1H), 6.44 (d, *J* = 9.6 Hz, 1H).

S3

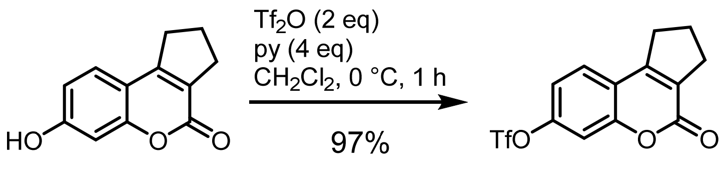

S3

To a stirred solution of 7-hydroxy-2,3-dihydro-1*H*-cyclopenta[*c*]chromen-4-one (190 mg, 0.94 mmol; prepared according to the literature procedure: compound **1** in [9]) and pyridine (300 µL, 3.76 mmol, 4 eq) in dry CH_2Cl_2 (9 mL), cooled in ice-water bath, a solution of trifluoromethanesulfonic anhydride (1.9 mL of 1 M in CH_2Cl_2, 1.9 mmol, 2 eq) was added quickly dropwise. The reaction mixture was stirred for 1 h at 0 °C, then poured into water (50 mL), extracted with CH_2Cl_2 (3 × 25 mL); the combined extracts were washed with water, brine and dried over Na_2SO_4. The filtrate was evaporated on Celite, and the product was isolated by flash column chromatography (12 g 30 µm Interchim Puriflash SiHP, gradient 10% to 80% EtOAc:hexane) to give 304 mg (97%) of **S3** as white solid.

^{1}H NMR (400 MHz, $CDCl_3$): δ 7.53 (d, *J* = 8.6 Hz, 1H), 7.31 (d, *J* = 2.4 Hz, 1H), 7.22 (dd, *J* = 8.6, 2.4 Hz, 1H), 3.15 – 3.06 (m, 2H), 3.01 – 2.91 (m, 2H), 2.31 – 2.20 (m, 2H).
^{19}F NMR (376 MHz, $CDCl_3$): δ -72.57.
^{13}C NMR (101 MHz, $CDCl_3$): δ 159.1, 154.9, 154.7, 150.2, 129.4, 126.4, 123.9 (q, $^{1}J_{C-F}$ = 320.7 Hz), 119.0, 117.5, 110.5, 32.3, 30.9, 22.5.
HRMS (ESI) m/z: [M+H]+ Calcd for $C_{13}H_{10}F_3O_5S$ 335.0196; Found 335.0195.

4l

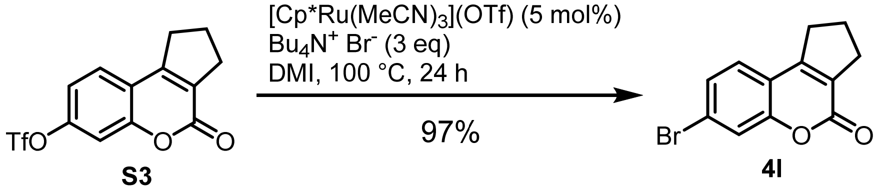

S3 **4l**

Prepared similarly to compound **4k** from **S3** (282 mg, 0.84 mmol), tetrabutylammonium bromide (816 mg, 2.53 mmol, 3 eq) and tris(acetonitrile)pentamethylcyclopentadienylruthenium(II)

trifluoromethanesulfonate (21.5 mg, 0.042 mmol, 5 mol%) in anhydrous 1,3-dimethyl-2-imidazolidinone (3 mL). The product was isolated by flash column chromatography (12 g 30 µm Interchim Puriflash SiHP, gradient 5% to 70% EtOAc:hexane). Yield 216 mg (97%), white solid.

^{1}H NMR (400 MHz, $CDCl_3$): δ 7.53 (d, *J* = 1.8 Hz, 1H), 7.41 (dd, *J* = 8.3, 1.8 Hz, 1H), 7.30 (d, *J* = 8.3 Hz, 1H), 3.12 – 3.04 (m, 2H), 2.96 – 2.86 (m, 2H), 2.29 – 2.18 (m, 2H).
^{13}C NMR (101 MHz, $CDCl_3$): δ 159.5, 155.6, 154.4, 128.5, 127.6, 125.9, 124.6, 120.1, 117.9, 32.1, 30.8, 22.5.
HRMS (ESI) m/z: [M+H]+ Calcd for $C_{12}H_{10}BrO_2$ 264.9859; Found 264.9858.

General synthetic procedure for compounds 3 and 5

In an oven dried 50 mL round bottom flask, a solution of *t*-BuLi (1.1 mL of 1.7 M in pentane, 1.84 mmol, 4.2 eq) was added dropwise to a solution of dibromide **1a-f** (0.439 mmol) in anhydrous THF (10 mL), cooled down to -78 °C in a dry ice-acetone bath. After 1 h, a solution of $LaCl_3 \cdot 2LiCl$ (1.6 mL of 0.6 M in THF, 0.966 mmol, 2.2 eq) was added dropwise, and the reaction mixture was stirred at -78 °C for further 30 min. A solution of anhydride (**2a-j**), ester (**2g', 2k,l**) or lactone (**4a-n**) (0.66 mmol, 1.5 eq) in THF (5 mL) was then added dropwise, the mixture was allowed to warm up to rt and left stirring overnight. Sat. aq. $NaHCO_3$ (30 mL) was added, and the mixture was extracted with EtOAc (3 × 30 mL). The combined extracts were dried over Na_2SO_4, the filtrate was evaporated on Celite and the product was isolated by flash column chromatography, the combined fractions were evaporated and the residue was freeze-dried from dioxane or aqueous dioxane. For photophysical characterization purposes, a sample (20-50 mg) of the material was additionally purified by RP-HPLC.

3a

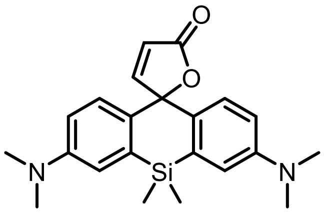

3a

Yield 73 mg (44%) from **1a** (0.44 mmol; prepared according to the procedure in [10]) and maleic anhydride (**2a**), off-white solid. The product was isolated by flash column chromatography (25 g 30 µm Interchim Puriflash SiHP, gradient 20% to 70% EtOAc:hexane).

^{1}H NMR (400 MHz, $CDCl_3$): δ 7.48 (d, J = 5.4 Hz, 1H), 7.36 (d, J = 8.9 Hz, 2H), 6.94 (d, J = 2.9 Hz, 2H), 6.73 (dd, J = 8.9, 2.9 Hz, 2H), 5.91 (d, J = 5.4 Hz, 1H), 2.98 (s, 12H), 0.60 (s, 3H), 0.50 (s, 3H).
^{13}C NMR (101 MHz, $CDCl_3$): δ 174.7, 163.8, 149.7, 135.2, 130.9, 125.6, 116.9, 115.7, 114.1, 91.3, 40.5, 0.1, -1.4.
HRMS (ESI) m/z: [M+H]+ Calcd for $C_{22}H_{27}N_2O_2Si$ 379.1836; Found 379.1833.

3b

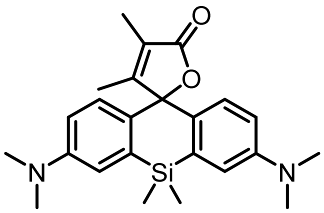

3b

Yield 114 mg (70%) from **1a** (0.4 mmol) and 2,3-dimethylmaleic anhydride (**2b**), off-white solid. The product was isolated by flash column chromatography (25 g 30 µm Interchim Puriflash SiHP, gradient 20% to 70% EtOAc:hexane).

^{1}H NMR (400 MHz, $CDCl_3$): δ 7.11 (d, J = 8.9 Hz, 2H), 6.86 (d, J = 2.8 Hz, 2H), 6.75 (dd, J = 8.9, 2.8 Hz, 2H), 2.99 (s, 12H), 1.90 (q, J = 1.1 Hz, 3H), 1.55 (q, J = 1.1 Hz, 3H), 0.50 (s, 3H), 0.49 (s, 3H).
^{13}C NMR (101 MHz, $CDCl_3$): δ 176.6, 167.2, 149.2, 135.1, 129.6, 127.3, 120.0, 115.8, 114.5, 91.0, 40.4, 12.2, 8.9, -0.1, -0.2.
HRMS (ESI) m/z: [M+H]+ Calcd for $C_{24}H_{31}N_2O_2Si$ 407.2149; Found 407.2146.

3c

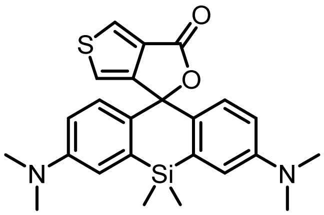

3c

Yield 108 mg (57%) from **1a** (0.44 mmol) and 2,3-thiophenedicarboxylic anhydride (**2c**), green solid. The product was isolated by flash column chromatography (25 g 30 µm Interchim Puriflash SiHP, gradient 0% to 100% A:B, A = CH_2Cl_2:EtOH:H_2O 60:35:5, B = CH_2Cl_2).

^{1}H NMR (400 MHz, $CDCl_3$ + 1% TFA-*d*): δ 11.23 (s, 1H, OH), 8.39 (d, *J* = 3.3 Hz, 1H), 7.21 (d, *J* = 3.3 Hz, 1H), 7.13 – 7.04 (m, 4H), 6.58 (dd, *J* = 9.6, 2.7 Hz, 2H), 3.29 (s, 12H), 0.57 (s, 3H), 0.45 (s, 3H).

^{13}C NMR (101 MHz, $CDCl_3$ + 1% TFA-*d*) δ 165.9, 164.7, 154.2, 148.3, 141.3, 139.4, 136.3, 133.7, 128.7, 126.4, 120.4, 114.0, 40.8, -0.7, -1.8.

HRMS (ESI) m/z: [M+H]+ Calcd for $C_{24}H_{27}N_2O_2SSi$ 435.1557; Found 435.1557.

3d

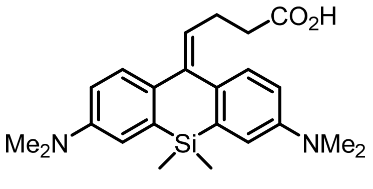

3d

Yield 123 mg (71%) from **1a** (0.44 mmol) and glutaric anhydride (**2d**), light blue solid. The product was isolated by flash column chromatography (25 g 30 µm Interchim Puriflash NH2, gradient 0% to 50% A:B, A – water + 0.1% (v/v) HCO_2H, B – acetonitrile + 0.1% (v/v) HCO_2H) and repurified by preparative HPLC (ThermoFisher Hypersil GOLD 250×21.2 mm 5 µm, solvent flow rate 15 mL/min, gradient 40% to 80% A:B, A – acetonitrile, B – 50 mM Et_3NH^+ HCO_3^- in water). Known compound (compound **1** in [11]).

^{1}H NMR (400 MHz, CD_3OD): δ 7.34 (d, *J* = 8.6 Hz, 1H), 7.29 (d, *J* = 8.5 Hz, 1H), 7.00 (d, *J* = 2.8 Hz, 1H), 6.93 (d, *J* = 2.7 Hz, 1H), 6.80 (dd, *J* = 3.8, 2.8 Hz, 1H), 6.78 (dd, *J* = 3.8, 2.8 Hz, 1H), 5.72 (t, *J* = 7.3 Hz, 1H), 3.12 (q, *J* = 7.3 Hz, 4H), 2.94 (s, 5H), 2.91 (s, 6H), 2.70 – 2.62 (m, 2H), 2.33 (dd, *J* = 8.5, 6.7 Hz, 2H), 1.26 (t, *J* = 7.3 Hz, 6H), 0.52 (s, 1H), 0.38 (s, 6H).

3e

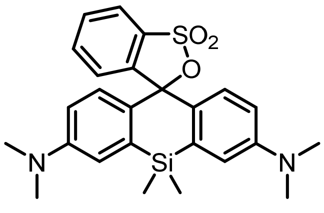

3e

Yield 135 mg (65%) from **1a** (0.44 mmol) and 2-sulfobenzoic anhydride (**2e**), blue solid. The product was isolated by flash column chromatography (25 g 30 µm Interchim Puriflash SiHP, gradient 0% to 100% A:B, A = 25% EtOH in CH_2Cl_2, B = CH_2Cl_2). Known compound (“Berkeley Red” in [12]).

^{1}H NMR (400 MHz, $CDCl_3$ + 2% TFA-*d*): δ 9.10 (br.s, 2H, TFA), 8.27 (d, *J* = 7.6 Hz, 1H), 7.62 (td, *J* = 7.6, 1.4 Hz, 1H), 7.55 (t, *J* = 7.6 Hz, 1H), 7.20 – 7.10 (m, 3H), 7.04 (d, *J* = 2.7 Hz, 2H), 6.57 (dd, *J* = 9.6, 2.7 Hz, 2H), 3.25 (s, 12H), 0.54 (s, 3H), 0.52 (s, 3H).
^{13}C NMR (101 MHz, $CDCl_3$ + 2% TFA-*d*): δ 170.9, 158.2 (q, $^2J_{C\text{-}F}$ = 40.8 Hz, TFA), 154.0, 148.5, 143.2, 142.7, 136.1, 130.1, 129.7, 129.4, 129.0, 128.9, 120.0, 114.9 (q, $^1J_{C\text{-}F}$ = 286.0 Hz, TFA), 113.8, 40.7, -1.2, -1.3.

3f

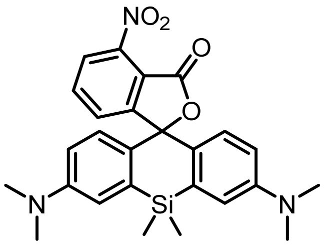

3f

Yield 109 mg (52%) from **1a** (0.44 mmol) and 3-nitrophthalic anhydride (**2f**), yellow solid. The product was isolated by flash column chromatography (12 g 30 µm Interchim Puriflash SiHP, gradient 10% to 80% EtOAc:hexane).

^{1}H NMR (400 MHz, $CDCl_3$): δ 7.84 (dd, *J* = 7.8, 0.8 Hz, 1H), 7.70 (t, *J* = 7.8 Hz, 1H), 7.42 (dd, *J* = 7.8, 0.9 Hz, 1H), 6.94 (d, *J* = 2.8 Hz, 2H), 6.81 (d, *J* = 8.9 Hz, 2H), 6.60 (dd, *J* = 9.0, 2.9 Hz, 2H), 2.98 (s, 12H), 0.64 (s, 3H), 0.58 (s, 3H).
^{13}C NMR (101 MHz, $CDCl_3$): δ 165.0, 158.4, 149.5, 147.0, 136.4, 134.7, 130.2, 128.4, 128.3, 123.5, 118.4, 116.5, 113.8, 90.7, 40.3, 0.3, -0.8.
HRMS (ESI) m/z: [M+H]+ Calcd for $C_{26}H_{28}N_3O_4Si$ 474.1844; Found 474.1841.

3g

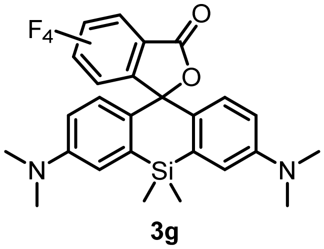

3g

Yield 66 mg (30%) from **1a** (0.44 mmol) and tetrafluorophthalic anhydride (**2g**) or 84 mg (38%) from **1a** (0.44 mmol) and dimethyl tetrafluorophthalate (**2g'** [13]), yellow-green solid. The product was isolated by flash column chromatography (12 g 30 µm Interchim Puriflash SiHP, gradient 0% to 50% EtOAc:hexane with 20% CH_2Cl_2 constant additive). Known compound (compound **37** in [14]).

^{1}H NMR (400 MHz, $CDCl_3$): δ 6.94 (d, J = 2.9 Hz, 2H), 6.79 (d, J = 8.9 Hz, 2H), 6.61 (dd, J = 8.9, 2.9 Hz, 2H), 2.99 (s, 12H), 0.59 (s, 3H), 0.56 (s, 3H).
^{19}F NMR (376 MHz, $CDCl_3$): δ -138.59 – -138.97 (m, 2F), -143.40 – -143.68 (m, 1F), -151.41 – -151.71 (m, 1F).

3h

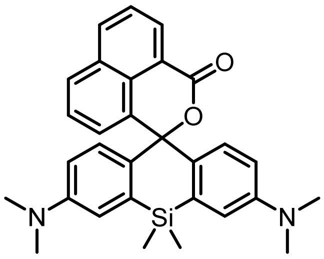

3h

Yield 122 mg (58%) from **1a** (0.44 mmol) and naphthalic anhydride (**2h**), yellowish solid. The product was isolated by flash column chromatography (12 g 30 µm Interchim Puriflash SiHP pretreated with 2% Et_3N-hexane, gradient 10% to 80% EtOAc:hexane).

^{1}H NMR (400 MHz, $CDCl_3$): δ 8.55 (dd, J = 7.2, 1.2 Hz, 1H), 8.18 (dd, J = 8.4, 1.3 Hz, 1H), 7.83 (dd, J = 8.3, 1.1 Hz, 1H), 7.70 (dd, J = 8.3, 7.2 Hz, 1H), 7.44 (dd, J = 8.2, 7.3 Hz, 1H), 7.10 (dd, J = 7.3, 1.1 Hz, 1H), 6.90 (d, J = 2.9 Hz, 2H), 6.80 (d, J = 8.9 Hz, 2H), 6.52 (dd, J = 9.0, 2.9 Hz, 2H), 2.94 (s, 12H), 0.64 (s, 3H), 0.62 (s, 3H).
^{13}C NMR (101 MHz, $CDCl_3$): δ 163.4, 148.9, 137.7, 137.3, 134.8, 133.6, 132.2, 130.7, 129.4, 127.9, 127.8, 126.7, 126.3, 126.2, 120.7, 115.4, 114.2, 91.1, 40.48, 0.3, -0.8.
HRMS (ESI) m/z: [M+H]+ Calcd for $C_{30}H_{31}N_2O_2Si$ 479.2149; Found 479.2143.

3i

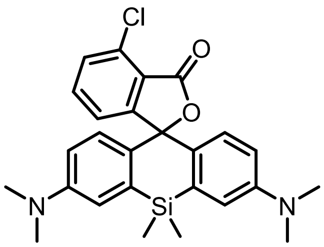

3i

Yield 143 mg (70%) from **1a** (0.44 mmol) and 3-chlorophthalic anhydride (**2i**), greenish solid. The product was isolated by flash column chromatography (12 g 30 µm Interchim Puriflash SiHP, gradient 50% to 100% CH_2Cl_2:hexane followed by 0% to 20% EtOAc:CH_2Cl_2).

^{1}H NMR (400 MHz, $CDCl_3$): δ 7.51 – 7.42 (m, 2H), 7.10 (dd, J = 7.4, 1.1 Hz, 1H), 6.94 (d, J = 2.9 Hz, 2H), 6.82 (d, J = 8.9 Hz, 2H), 6.58 (dd, J = 9.0, 2.9 Hz, 2H), 2.96 (s, 13H), 0.63 (s, 3H), 0.58 (s, 3H).
^{13}C NMR (101 MHz, $CDCl_3$): δ 167.9, 157.9, 149.4, 136.5, 134.8, 133.0, 131.4, 130.0, 128.3, 123.0, 122.9, 116.5, 113.7, 90.0, 40.4, 0.4, -1.0.
HRMS (ESI) m/z: [M+H]+ Calcd for $C_{26}H_{28}ClN_2O_2Si$ 463.1603; Found 463.1603.

3j

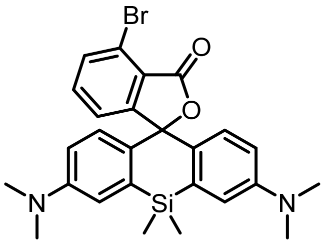

3j

Yield 219 mg (67%) from **1a** (0.64 mmol) and 3-bromophthalic anhydride (**2j**), greenish solid. The product was isolated by flash column chromatography (25 g 30 µm Interchim Puriflash SiHP, gradient 0% to 40% EtOAc:hexane with 20% CH_2Cl_2 constant additive).

^{1}H NMR (400 MHz, $CDCl_3$): δ 7.64 (dd, J = 7.8, 0.8 Hz, 1H), 7.39 (t, J = 7.7 Hz, 1H), 7.14 (dd, J = 7.7, 0.8 Hz, 1H), 6.95 (br.s, 2H), 6.82 (d, J = 8.9 Hz, 2H), 6.59 (br.dd, J = 9.1, 2.9 Hz, 2H), 2.96 (s, 12H), 0.63 (s, 3H), 0.58 (s, 3H).
^{13}C NMR (101 MHz, $CDCl_3$): δ 168.3, 158.0, 149.4, 136.4, 134.9, 133.4, 131.4, 128.3, 124.4, 123.5, 120.6, 116.5, 113.8, 89.6, 40.4, 0.4, -0.9.
HRMS (ESI) m/z: [M+H]+ Calcd for $C_{26}H_{28}BrN_2O_2Si$ 507.1098; Found 507.1096.

3k

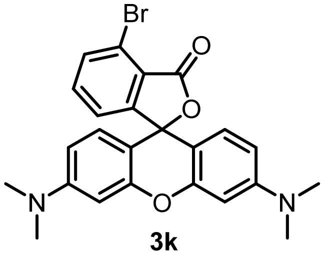

3k

Yield 119 mg (51%) from **1b** (0.5 mmol; prepared according to the procedure reported in [15]) and 3-bromophthalic anhydride (**2j**), bright pink solid. The product was isolated by flash column chromatography (25 g 30 µm Interchim Puriflash SiHP, gradient 0% to 100% A:B, A = CH_2Cl_2:EtOH:25% aq. NH_3 70:30:2, B = CH_2Cl_2).

^{1}H NMR (400 MHz, DMSO-d_6 + 1% TFA-d): δ 8.01 (dd, J = 8.1, 1.0 Hz, 1H), 7.66 (t, J = 7.9 Hz, 1H), 7.50 (dd, J = 7.7, 1.0 Hz, 1H), 7.08 (br.s, 4H), 6.85 (br.s, 2H), 3.24 (s, 12H).
^{13}C NMR (101 MHz, DMSO-d_6 + 1% TFA-d): δ 166.9, 156.7, 156.5, 134.4, 131.7, 131.1, 128.5, 119.1, 114.4, 112.5, 96.4, 40.6.
HRMS (ESI) m/z: [M+H]+ Calcd for $C_{24}H_{22}BrN_2O_3$ 465.0808; Found 465.0807.

3l

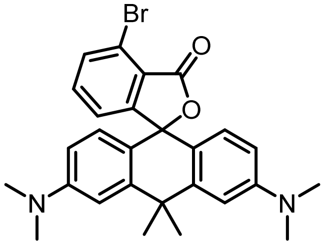

3l

Yield 67 mg (38%) from **1c** (0.36 mmol) and 3-bromophthalic anhydride (**2j**), light turquoise solid. The product was isolated by flash column chromatography (12 g 30 μm Interchim Puriflash SiHP, gradient 20% to 80% EtOAc:hexane).

^{1}H NMR (400 MHz, $CDCl_3$): δ 7.67 (d, J = 7.7 Hz, 1H), 7.37 (t, J = 7.7 Hz, 1H), 6.97 (d, J = 7.7 Hz, 1H), 6.87 (d, J = 2.6 Hz, 2H), 6.62 (d, J = 8.8 Hz, 2H), 6.53 (dd, J = 8.8, 2.6 Hz, 2H), 2.98 (s, 12H), 1.86 (s, 3H), 1.76 (s, 3H).
^{13}C NMR (101 MHz, $CDCl_3$): δ 168.2, 158.5, 150.8, 146.8, 135.4, 133.5, 129.0, 125.1, 123.2, 120.0, 119.1, 111.8, 109.3, 86.2, 40.5, 38.6, 35.7, 33.0.
HRMS (ESI) m/z: [M+H]+ Calcd for $C_{27}H_{28}BrN_2O_2$ 491.1329; Found 491.1328.

3m

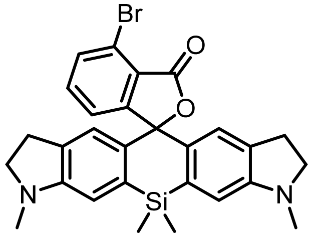

3m

Yield 155 mg (58%) from **1d** (0.5 mmol) and 3-bromophthalic anhydride (**2j**), yellowish solid. The product was isolated by flash column chromatography (12 g 30 μm Interchim Puriflash SiHP, gradient 10% to 60% EtOAc:hexane with 10% CH_2Cl_2 constant additive).

^{1}H NMR (400 MHz, $CDCl_3$): δ 7.58 (dd, J = 7.7, 0.8 Hz, 1H), 7.32 (t, J = 7.7 Hz, 1H), 7.01 (dd, J = 7.7, 0.8 Hz, 1H), 6.71 (s, 2H), 6.61 (s, 2H), 3.33 – 3.23 (m, 4H), 2.91 – 2.74 (m, 4H), 2.80 (s, 6H), 0.58 (s, 3H), 0.54 (s, 3H).
^{13}C NMR (101 MHz, $CDCl_3$): δ 169.0, 159.7, 152.5, 135.3, 133.5, 133.1, 132.8, 123.4, 123.3, 122.8, 120.5, 110.0, 89.8, 55.6, 35.7, 28.7, 0.1, 0.0.
HRMS (ESI) m/z: [M+H]+ Calcd for $C_{28}H_{28}BrN_2O_2Si$ 531.1098; Found 531.1094.

3n

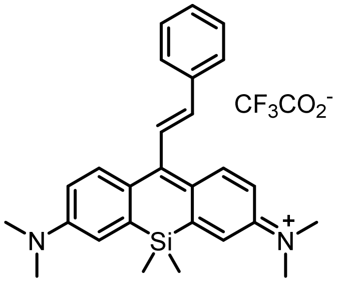

3n

Yield 90 mg (43%) from **1a** (0.4 mmol) and methyl cinnamate (**2k**), dark green solid (trifluoroacetate salt). The reaction mixture was cooled in ice-water bath and quenched with 2 N HCl (1.5 mL) before workup. The product was isolated by flash column chromatography (12 g 30 µm Interchim Puriflash SiHP, gradient 0% to 20% MeOH:CH_2Cl_2) and repurified by preparative HPLC (Interchim Uptisphere Strategy C18-HQ 250×30 mm 10 µm, solvent flow rate 40 mL/min, gradient 50% to 90% A:B, A – acetonitrile + 0.1% (v/v) TFA, B – water + 0.1% (v/v) TFA).

^{1}H NMR (400 MHz, DMSO-d_6): δ 8.07 (d, J = 9.6 Hz, 2H), 8.01 (d, J = 16.2 Hz, 1H), 7.83 – 7.77 (m, 2H), 7.53 – 7.38 (m, 3H), 7.39 (d, J = 2.8 Hz, 2H), 6.97 (dd, J = 9.6, 2.8 Hz, 2H), 6.82 (d, J = 16.2 Hz, 1H), 3.32 (s, 12H), 0.55 (s, 6H).
^{13}C NMR (101 MHz, DMSO-d_6): δ 166.4, 153.7, 146.6, 141.0, 139.7, 135.7, 129.5, 128.9, 127.7, 127.2, 126.6, 121.1, 114.0, 40.4, -1.2.
HRMS (ESI) m/z: [M]+ Calcd for $C_{27}H_{31}N_2Si$ 411.2251; Found 411.2252.

3o

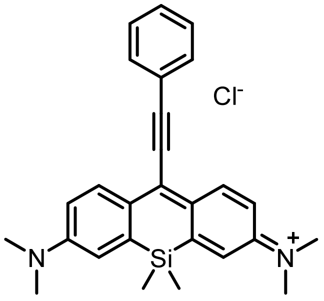

3o

Yield 121 mg (68%) from **1a** (0.4 mmol) and ethyl phenylpropiolate (**2l**), dark green solid (chloride salt). The reaction mixture was cooled in ice-water bath and quenched with 2 N HCl (1.5 mL) before workup. The product was isolated by flash column chromatography (12 g 30 µm Interchim Puriflash SiHP, gradient 0% to 20% MeOH:CH_2Cl_2) and repurified by preparative HPLC (Interchim Uptisphere Strategy C18-HQ 250×30 mm 10 µm, solvent flow rate 40 mL/min, gradient 50% to 90% A:B, A – acetonitrile + 0.1% (v/v) TFA, B – water + 0.1% (v/v) TFA). Known compound (compound **1b** in [16]).

^{1}H NMR (400 MHz, CD_3OD): δ 8.39 (d, J = 9.5 Hz, 2H), 7.71 – 7.66 (m, 2H), 7.60 – 7.55 (m, 1H), 7.54 – 7.48 (m, 2H), 7.17 (d, J = 2.9 Hz, 2H), 6.96 (dd, J = 9.5, 2.9 Hz, 2H), 3.33 (s, 12H), 0.56 (s, 6H).
^{13}C NMR (101 MHz, CD_3OD): δ 155.6, 148.8, 147.3, 141.6, 133.7, 132.3, 130.1, 129.7, 122.6, 121.5, 115.8, 113.2, 90.5, 41.0, -1.0.

5a

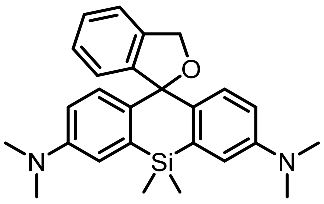

5a

Yield 116 mg (64%) from **1a** (0.44 mmol) and phthalide (**4a**), light green solid. The product was isolated by flash column chromatography (12 g 30 µm Interchim Puriflash SiHP, gradient 5% to 50% A:B, A – 2% (v/v) acetic acid in EtOAc, B – 2% (v/v) acetic acid in hexane). Known compound (compound **4** in [17]).

^{1}H NMR (400 MHz, $CDCl_3$): δ 7.32 – 7.28 (m, 2H), 7.28 – 7.21 (m, 1H), 7.06 (dt, J = 7.3, 1.0 Hz, 1H), 6.97 (dd, J = 5.9, 2.9 Hz, 4H), 6.61 (dd, J = 8.9, 2.9 Hz, 2H), 5.23 (s, 2H), 2.94 (s, 12H), 0.62 (s, 3H), 0.55 (s, 3H).

5b

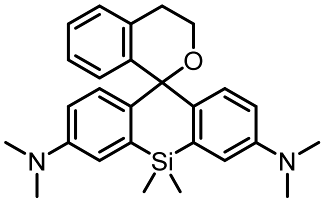

5b

Yield 164 mg (87%) from **1a** (0.44 mmol) and 1-isochromanone (**4b**), light tan solid. The product was isolated by flash column chromatography (12 g 30 μm Interchim Puriflash SiHP, gradient 20% to 80% A:B, A – 2% (v/v) Et_3N in EtOAc, B – hexane).

^{1}H NMR (400 MHz, $CDCl_3$): δ 7.31 – 7.22 (m, 2H), 7.18 (ddd, J = 7.8, 6.9, 1.8 Hz, 1H), 7.06 (d, J = 2.9 Hz, 2H), 7.04 (dd, J = 7.8, 1.3 Hz, 1H), 6.74 (d, J = 8.8 Hz, 2H), 6.50 (dd, J = 8.8, 2.9 Hz, 2H), 3.60 (t, J = 5.5 Hz, 2H), 2.94 (s, 12H), 2.85 (t, J = 5.5 Hz, 2H), 0.62 (s, 3H), 0.50 (s, 3H).

^{13}C NMR (101 MHz, $CDCl_3$): δ 148.8, 139.4, 138.9, 138.3, 136.4, 131.1, 130.6, 128.9, 126.5, 124.9, 117.7, 112.1, 82.3, 59.2, 40.7, 29.6, 1.2, -2.7.

HRMS (ESI) m/z: [M+H]+ Calcd for $C_{27}H_{33}N_2OSi$ 429.2357; Found 429.2355.

5c

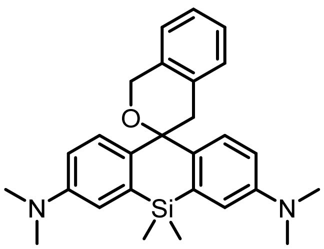

5c

Yield 58 mg (31%) from **1a** (0.44 mmol) and 3-isochromanone (**4c**), light tan solid. The product was isolated by flash column chromatography (25 g 30 μm Interchim Puriflash SiHP, gradient 10% to 80% EtOAc:hexane).

^{1}H NMR (400 MHz, $CDCl_3$): δ 7.34 (dd, J = 7.7, 1.3 Hz, 1H), 7.23 – 7.16 (m, 3H), 7.09 (td, J = 7.5, 1.3 Hz, 1H), 7.05 (d, J = 2.8 Hz, 2H), 6.80 (dd, J = 7.6, 1.3 Hz, 1H), 6.48 (dd, J = 8.6, 2.9 Hz, 2H), 4.35 (s, 2H), 3.82 (s, 2H), 2.89 (s, 12H), 0.63 (s, 3H), 0.51 (s, 3H).

^{13}C NMR (101 MHz, $CDCl_3$): δ 148.9, 139.4, 138.9, 134.8, 134.2, 128.5, 126.5, 125.7, 125.6, 124.0, 119.3, 112.0, 76.7, 63.5, 40.9, 34.7, 0.2, -1.4.

HRMS (ESI) m/z: [M+H]+ Calcd for $C_{27}H_{33}N_2OSi$ 429.2357; Found 429.2360.

5d

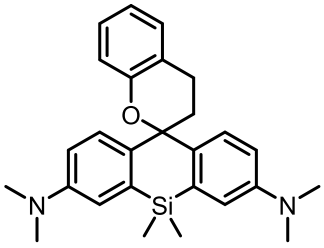

5d

Yield 136 mg (72%) from **1a** (0.44 mmol) and dihydrocoumarin (**4d**), white solid. The product was isolated by flash column chromatography (25 g 30 µm Interchim Puriflash SiHP, gradient 30% to 100% CH_2Cl_2:hexane).

^{1}H NMR (400 MHz, $CDCl_3$): δ 7.50 (d, J = 8.8 Hz, 2H), 7.28 – 7.22 (td, J = 7.3, 1.6 Hz, 1H), 7.18 (dd, J = 8.2, 1.4 Hz, 1H), 7.04 (dd, J = 7.5, 1.6 Hz, 1H), 6.97 (d, J = 2.9 Hz, 2H), 6.88 (td, J = 7.3, 1.3 Hz, 1H), 6.72 (dd, J = 8.8, 2.9 Hz, 2H), 2.95 (s, 12H), 2.41 (t, J = 6.1 Hz, 2H), 2.09 – 1.99 (m, 2H), 0.55 (s, 3H), 0.51 (s, 3H).

^{13}C NMR (101 MHz, $CDCl_3$): δ 156.1, 148.7, 140.6, 133.4, 129.2, 127.8, 126.0, 123.1, 119.6, 117.4, 116.3, 113.9, 81.9, 41.3, 40.8, 23.0, 1.1, -1.4.

HRMS (ESI) m/z: [M+H]+ Calcd for $C_{27}H_{33}N_2OSi$ 429.2357; Found 429.2358.

5e

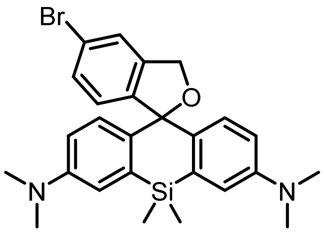

5e

Yield 136 mg (63%) from **1a** (0.44 mmol) and 5-bromophthalide (**4e**), greenish solid. The product was isolated by flash column chromatography (25 g 30 µm Interchim Puriflash SiHP, gradient 0% to 50% A:B, A – 1% acetic acid in EtOAc, B – 1% acetic acid in CH_2Cl_2).

^{1}H NMR (400 MHz, $CDCl_3$): δ 7.44 (q, J = 0.7 Hz, 1H), 7.40 – 7.34 (m, 1H), 6.98 – 6.91 (m, 6H), 6.61 (dd, J = 8.9, 2.9 Hz, 3H), 5.17 (s, 2H), 2.94 (s, 16H), 0.61 (s, 4H), 0.53 (s, 3H).

^{13}C NMR (101 MHz, $CDCl_3$): δ 148.9, 145.5, 142.4, 137.7, 135.8, 130.6, 128.6, 126.3, 124.4, 121.1, 116.9, 113.8, 92.5, 71.7, 40.6, 0.7, -1.2.

HRMS (ESI) m/z: [M+H]+ Calcd for $C_{26}H_{30}BrN_2OSi$ 493.1305; Found 493.1306.

5f

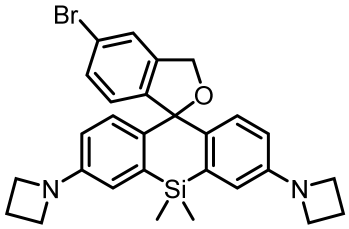

5f

Yield 113 mg (70%) from **1e** (0.31 mmol; known compound: compound **31a** in [14]) and 5-bromophthalide (**4e**), light tan solid. The product was isolated by flash column chromatography (12 g 30 μm Interchim Puriflash SiHP, gradient 5% to 60% A:B, A – 2% Et_3N in EtOAc, B – 2% Et_3N in hexane).

^1H NMR (400 MHz, $CDCl_3$): δ 7.45 – 7.42 (m, 1H), 7.39 – 7.34 (m, 1H), 6.93 (d, J = 8.1 Hz, 1H), 6.91 (d, J = 8.6 Hz, 2H), 6.66 (d, J = 2.6 Hz, 2H), 6.31 (dd, J = 8.6, 2.7 Hz, 2H), 5.15 (s, 2H), 3.87 (t, J = 7.2 Hz, 8H), 2.41 – 2.29 (m, 4H), 0.57 (s, 3H), 0.51 (s, 3H).
^{13}C NMR (101 MHz, $CDCl_3$): δ 150.5, 145.3, 142.4, 138.5, 135.7, 130.5, 128.3, 126.4, 124.5, 121.2, 115.7, 112.5, 92.7, 71.7, 52.5, 17.1, 0.7, -1.3.
HRMS (ESI) m/z: [M+H]+ Calcd for $C_{28}H_{30}BrN_2OSi$ 517.1305; Found 517.1302.

5g

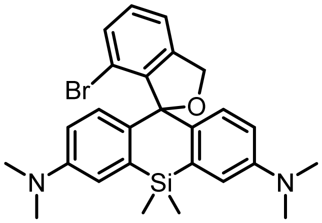

5g

Yield 496 mg (50%) from **1a** (2.0 mmol) and 7-bromophthalide (**4f**), greenish solid. The product was isolated by flash column chromatography (40 g 25 μm Silicycle SiliaSep Premium, gradient 5% to 80% EtOAc:hexane).

^1H NMR (400 MHz, $CDCl_3$): δ 7.50 – 7.46 (m, 1H), 7.32 – 7.24 (m, 2H), 6.97 (d, J = 2.9 Hz, 2H), 6.71 (d, J = 8.8 Hz, 2H), 6.60 (dd, J = 8.8, 2.9 Hz, 2H), 5.09 (s, 2H), 2.96 (s, 12H), 0.58 (s, 3H), 0.51 (s, 3H).
^{13}C NMR (101 MHz, $CDCl_3$): δ 148.8, 144.8, 144.2, 136.3, 135.4, 132.1, 129.6, 129.0, 120.3, 120.0, 116.6, 113.8, 93.9, 71.3, 40.5, 1.1, -2.4.
HRMS (ESI) m/z: [M+H]+ Calcd for $C_{26}H_{30}BrN_2OSi$ 493.1305; Found 493.1299.

5h

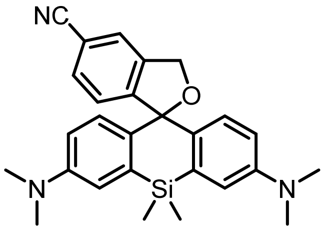

5h

Yield 75 mg (39%) from **1a** (0.44 mmol) and 5-cyanophthalide (**4g**), greenish solid. The product was isolated by flash column chromatography (25 g 30 μm Interchim Puriflash SiHP, gradient 5% to 50% EtOAc:hexane).

^{1}H NMR (400 MHz, $CDCl_3$): δ 8.36 (d, J = 8.6 Hz, 1H), 7.75 – 7.70 (m, 2H), 6.90 (d, J = 2.9 Hz, 2H), 6.76 (d, J = 8.9 Hz, 2H), 6.59 (dd, J = 8.9, 2.9 Hz, 2H), 3.94 (s, 2H), 2.96 (s, 12H), 0.59 (s, 3H), 0.49 (s, 3H).
^{13}C NMR (101 MHz, $CDCl_3$): δ 152.2, 148.9, 139.6, 137.5, 134.6, 132.7, 130.7, 130.3, 127.1, 119.3, 116.0, 114.4, 111.1, 78.4, 62.1, 40.4, 0.9, -1.8.
HRMS (ESI) m/z: [M+H]+ Calcd for $C_{27}H_{30}N_3OSi$ 440.2153; Found 440.2149.

5i

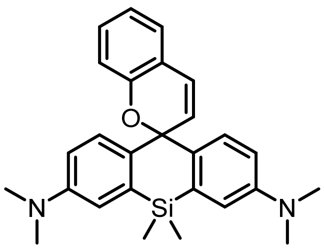

5i

Yield 156 mg (83%) from **1a** (0.44 mmol) and coumarin (**4h**), white solid. The product was isolated by flash column chromatography (25 g 30 μm Interchim Puriflash SiHP, gradient 2% to 50% EtOAc:hexane).

^{1}H NMR (400 MHz, $CDCl_3$): δ 7.65 (d, J = 8.8 Hz, 2H), 7.08 (ddd, J = 8.0, 7.4, 1.7 Hz, 1H), 7.00 (dd, J = 7.4, 1.7 Hz, 1H), 6.95 (d, J = 2.9 Hz, 2H), 6.79 (td, J = 7.4, 1.2 Hz, 1H), 6.77 – 6.71 (m, 3H), 6.42 (dd, J = 10.0, 0.8 Hz, 1H), 5.81 (d, J = 10.0 Hz, 1H), 2.96 (s, 12H), 0.60 (s, 3H), 0.51 (s, 3H).
^{13}C NMR (101 MHz, $CDCl_3$): δ 153.5, 149.0, 138.4, 134.3, 130.0, 129.3, 128.7, 126.6, 120.2, 119.6, 119.5, 116.7, 115.6, 114.0, 82.3, 40.6, -0.3, -0.6.
HRMS (ESI) m/z: [M+H]+ Calcd for $C_{27}H_{31}N_2OSi$ 427.2200; Found 427.2196.

5j

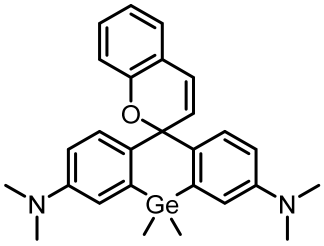

5j

Yield 141 mg (78%) from **1f** (0.38 mmol; known compound: compound **16a** in [18]) and coumarin (**4h**), white solid. The product was isolated by flash column chromatography (25 g 30 μm Interchim Puriflash SiHP, gradient 2% to 50% EtOAc:hexane).

^{1}H NMR (400 MHz, $CDCl_3$): δ 7.62 (d, J = 8.8 Hz, 2H), 7.08 – 7.02 (m, 1H), 6.97 (dd, J = 7.6, 1.7 Hz, 1H), 6.93 (d, J = 2.8 Hz, 2H), 6.81 – 6.72 (m, 2H), 6.66 (dd, J = 8.8, 2.8 Hz, 2H), 6.49 (dd, J = 10.1, 0.7 Hz, 1H), 6.02 (d, J = 10.0 Hz, 1H), 2.94 (s, 12H), 0.77 (s, 3H), 0.66 (s, 3H).
^{13}C NMR (101 MHz, $CDCl_3$): δ 153.5, 149.1, 138.0, 137.5, 129.3, 129.2, 127.9, 126.6, 120.8, 120.3, 120.1, 117.3, 115.8, 112.9, 82.1, 40.7, -0.1, -1.5.
HRMS (ESI) m/z: [M+H]+ Calcd for $C_{27}H_{31}GeN_2O$ 469.1673; Found 469.1672.

5k

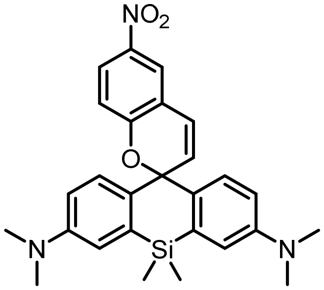

5k

Yield 58 mg (28%) from **1a** (0.44 mmol) and 6-nitrocoumarin (**4i**), yellow solid. The product was isolated by flash column chromatography (12 g 30 μm Interchim Puriflash SiHP, gradient 2% to 30% EtOAc:hexane with 20% CH_2Cl_2 constant additive).

^{1}H NMR (400 MHz, $CDCl_3$): δ 7.98 – 7.93 (m, 2H), 7.53 (d, J = 8.8 Hz, 2H), 6.96 (d, J = 2.9 Hz, 2H), 6.76 – 6.68 (m, 3H), 6.60 (dd, J = 10.2, 0.7 Hz, 1H), 6.04 (d, J = 10.2 Hz, 1H), 2.98 (s, 12H), 0.60 (s, 3H), 0.54 (s, 3H).
^{13}C NMR (101 MHz, $CDCl_3$): δ 158.7, 149.3, 141.0, 136.0, 135.2, 131.4, 129.2, 125.5, 122.5, 119.5, 119.1, 116.7, 116.1, 113.6, 84.5, 40.5, 0.2, -1.3.
HRMS (ESI) m/z: [M+H]+ Calcd for $C_{27}H_{30}N_3O_3Si$ 472.2051; Found 472.2048.

5l

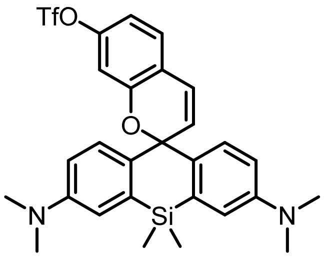

5l

Yield 163 mg (65%) from **1a** (0.44 mmol) and 2-oxo-2*H*-chromen-7-yl trifluoromethanesulfonate (**4j**; prepared according to the literature procedure: compound **S21** in [7]), bluish solid. The product was isolated by flash column chromatography (25 g 30 µm Interchim Puriflash SiHP, gradient 5% to 50% EtOAc:hexane).

^{1}H NMR (400 MHz, $CDCl_3$): δ 7.56 (d, J = 8.9 Hz, 2H), 7.04 (d, J = 8.3 Hz, 1H), 6.96 (d, J = 2.9 Hz, 2H), 6.74 (dd, J = 8.8, 2.9 Hz, 2H), 6.70 (dd, J = 8.3, 2.4 Hz, 1H), 6.60 (dd, J = 2.4, 0.7 Hz, 1H), 6.50 (dd, J = 10.2, 0.7 Hz, 1H), 5.93 (d, J = 10.2 Hz, 1H), 2.98 (s, 12H), 0.59 (s, 3H), 0.52 (s, 3H).

^{19}F NMR (376 MHz, $CDCl_3$): δ -72.96.

^{13}C NMR (101 MHz, $CDCl_3$): δ 154.4, 149.6, 149.2, 136.9, 135.0, 130.8, 129.0, 127.4, 119.9, 118.82 (q, $^1J_{C\text{-}F}$ = 320.8 Hz), 118.77, 116.8, 113.8, 112.7, 109.1, 83.2, 40.6, -0.0, -1.0.

HRMS (ESI) m/z: [M+H]+ Calcd for $C_{28}H_{30}F_3N_2O_4SSi$ 575.1642; Found 575.1637.

5m

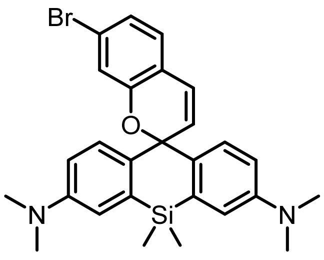

5m

Yield 190 mg (86%) from **1a** (0.44 mmol) and 7-bromocoumarin (**4k**), white solid. The product was isolated by flash column chromatography (25 g 30 µm Interchim Puriflash SiHP, gradient 1% to 30% EtOAc:hexane).

^{1}H NMR (400 MHz, $CDCl_3$): δ 7.58 (d, J = 8.9 Hz, 2H), 6.95 (d, J = 2.9 Hz, 2H), 6.91 (dd, J = 7.9, 1.9 Hz, 1H), 6.87 (dd, J = 1.9, 0.8 Hz, 1H), 6.85 (d, J = 7.9 Hz, 1H), 6.73 (dd, J = 8.9, 2.9 Hz, 2H), 6.44 (dd, J = 10.1, 0.8 Hz, 1H), 5.88 (d, J = 10.1 Hz, 1H), 2.97 (s, 12H), 0.60 (s, 3H), 0.51 (s, 3H).

^{13}C NMR (101 MHz, $CDCl_3$): δ 154.1, 149.2, 137.5, 134.8, 130.1, 128.8, 127.6, 123.2, 122.0, 119.3, 118.9, 118.8, 116.8, 113.8, 82.9, 40.6, -0.1, -0.9.
HRMS (ESI) m/z: [M+H]+ Calcd for $C_{27}H_{30}BrN_2OSi$ 505.1305; Found 505.1304.

5n

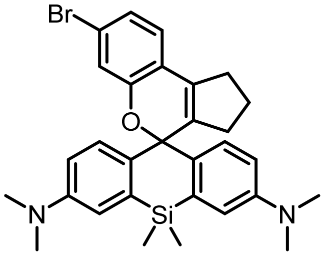

5n

Yield 204 mg (85%) from **1a** (0.44 mmol) and 7-bromo-2,3-dihydrocyclopenta[*c*]chromen-4(1*H*)-one (**4l**), white solid. The product was isolated by flash column chromatography (25 g 30 µm Interchim Puriflash SiHP, gradient 5% to 50% A:B, A – 1% Et_3N in EtOAc, B – 1% Et_3N in hexane).

^{1}H NMR (400 MHz, $CDCl_3$): δ 7.43 (d, J = 8.8 Hz, 2H), 6.95 (dd, J = 8.0, 1.9 Hz, 1H), 6.89 (d, J = 8.0 Hz, 1H), 6.86 – 6.83 (m, 3H), 6.75 (dd, J = 8.9, 2.9 Hz, 2H), 2.97 (s, 12H), 2.78 – 2.70 (m, 2H), 2.11 – 2.02 (m, 2H), 1.93 – 1.82 (m, 2H), 0.51 (s, 3H), 0.45 (s, 3H).
^{13}C NMR (101 MHz, $CDCl_3$): δ 153.8, 149.0, 140.5, 136.2, 133.8, 130.2, 127.7, 124.3, 122.6, 121.0, 118.9, 118.2, 115.2, 114.6, 86.6, 40.5, 34.1, 31.0, 22.1, -0.0, -0.6.
HRMS (ESI) m/z: [M+H]+ Calcd for $C_{30}H_{34}BrN_2OSi$ 545.1618; Found 545.1614.

General synthetic procedure for the preparation of live-cell dyes 6a-f by carbonylative hydroxylation

The modification of a procedure reported in [19] was employed. A dried small two-chamber Skrydstrup reactor (total inner volume 20 mL, available from Sigma-Aldrich as "COware gas reactor", Cat. No. STW1-1EA) was equipped with stirring bars in both chambers. In the chamber A, $Mo(CO)_6$ (106 mg, 0.4 mmol) was placed, and the chamber B was loaded with the corresponding aryl bromide (0.1 mmol), sodium formate (25 mg, 0.3 mmol, 3 eq) and a suitable Pd catalyst: <u>conditions A</u> – $Pd(dtbpf)Cl_2$ (6.6 mg, 0.01 mmol, 10 mol%), <u>conditions B</u> – Pd(*t*-$Bu_3P)_2$ (5.1 mg, 0.01 mmol, 10 mol%). The reactor was flushed with argon, and anhydrous 2-methyltetrahydrofuran (2-MeTHF) was added into both chambers (0.7 mL each). The reactor was sealed, and after briefly degassing the reaction mixture, 1,8-diazabicyclo[5.4.0]undec-7-ene (DBU, 180 µL) was injected into the chamber A. The reactor was immersed into an oil bath

preheated to 80 °C and the contents were stirred overnight (16 h). The reaction mixture from the chamber B was diluted with acetonitrile and filtered through a plug of Celite, washing with acetonitrile and acetonitrile-water. The filtrate was evaporated to dryness, the residue was dissolved in minimal volume of DMSO-acetonitrile, microfiltered through a 0.2 µm PTFE membrane filter, and the product was isolated by preparative HPLC as indicated below for individual compounds.

4-TMR-CO_2H (6a)

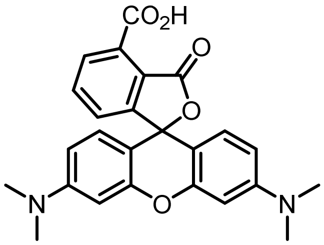

6a

Prepared from **3k** (30 mg, 0.065 mmol) according to conditions A. The product was isolated by preparative HPLC (Interchim Uptisphere Strategy PhC4 250×21.2 mm 5 µm, solvent flow rate 18 mL/min, gradient 20% to 60% A:B, A – acetonitrile + 0.1% (v/v) formic acid, B – water + 0.1% (v/v) formic acid). Yield 17 mg (61%), red solid. Known compound (compound **18** in [20]).

^{1}H NMR (400 MHz, DMSO-d_6): δ 7.84 (dd, J = 7.5, 1.1 Hz, 1H), 7.79 (t, J = 7.5 Hz, 1H), 7.31 (dd, J = 7.5, 1.1 Hz, 1H), 6.61 – 6.50 (m, 6H), 2.96 (s, 12H).

4-610CP-CO_2H (6b)

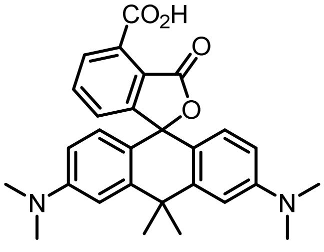

6b

Prepared from **3l** (40 mg, 0.082 mmol) according to conditions B. The product was isolated by preparative HPLC (Interchim Uptisphere Strategy PhC4 250×21.2 mm 5 µm, solvent flow rate 18 mL/min, gradient 25% to 70% A:B, A – acetonitrile + 0.1% (v/v) formic acid, B – water + 0.1% (v/v) formic acid). Yield 23 mg (62%), light blue solid. Known compound (compound **20** in [20]).

^{1}H NMR (400 MHz, CD_3OD): δ 8.17 (dd, J = 7.7, 1.2 Hz, 1H), 7.76 (t, J = 7.7 Hz, 1H), 7.52 (dd, J = 7.7, 1.2 Hz, 1H), 7.23 (d, J = 2.5 Hz, 2H), 7.08 (d, J = 9.4 Hz, 2H), 6.84 (dd, J = 9.4, 2.5 Hz, 2H), 3.34 (s, 12H), 1.86 (s, 3H), 1.72 (s, 3H).

4-SiR-CO$_2$H (6c)

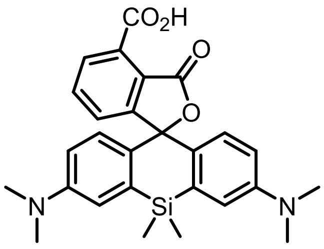

6c

Prepared from **3j** (30 mg, 0.059 mmol) according to conditions A. The product was isolated by preparative HPLC (Interchim Uptisphere Strategy PhC4 250×21.2 mm 5 µm, solvent flow rate 18 mL/min, gradient 25% to 70% A:B, A – acetonitrile + 0.1% (v/v) TFA, B – water + 0.1% (v/v) TFA). Yield 19 mg (61%), turquoise solid. Known compound (compound **21** in [20]).

^{1}H NMR (400 MHz, pyridine-d_5): δ 8.17 (dd, J = 7.5, 0.9 Hz, 1H), 7.78 (app.t, J = 7.6 Hz, 1H), 7.54 (dd, J = 7.8, 0.9 Hz, 1H), 7.18 (d, J = 2.9 Hz, 2H), 7.04 (d, J = 8.9 Hz, 2H), 6.53 (dd, J = 8.9, 2.9 Hz, 2H), 2.84 (s, 12H), 0.73 (s, 3H), 0.64 (s, 3H).

4-SiR700-CO$_2$H (6d)

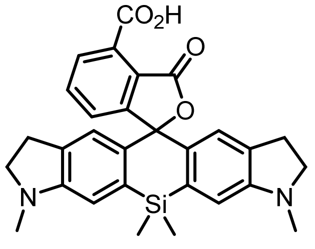

6d

Prepared from **3m** (40 mg, 0.075 mmol) according to conditions B. The product was isolated by preparative HPLC (Interchim Uptisphere Strategy PhC4 250×21.2 mm 5 µm, solvent flow rate 18 mL/min, gradient 30% to 70% A:B, A – acetonitrile + 0.1% (v/v) formic acid, B – water + 0.1% (v/v) formic acid). Yield 20 mg (54%), yellow-green solid.

^{1}H NMR (400 MHz, $CDCl_3$): δ 8.53 (dd, J = 7.7, 1.0 Hz, 1H), 7.82 (t, J = 7.7 Hz, 1H), 7.46 (dd, J = 7.7, 1.0 Hz, 1H), 6.65 (s, 2H), 6.46 (s, 1H), 3.35 – 3.28 (m, 4H), 2.88 – 2.74 (m, 10H), 0.61 (s, 3H), 0.56 (s, 3H).
^{13}C NMR (101 MHz, $CDCl_3$): δ 174.6, 164.4, 157.1, 153.1, 135.7, 135.4, 134.5, 132.8, 130.7, 130.2, 128.9, 124.0, 123.6, 110.3, 96.5, 55.4, 35.5, 28.6, 0.3, -1.0.
HRMS (ESI) m/z: [M+H]+ Calcd for $C_{29}H_{29}N_2O_4Si$ 497.1891; Found 497.1887.

5-HMSiR-CO_2H (6e)

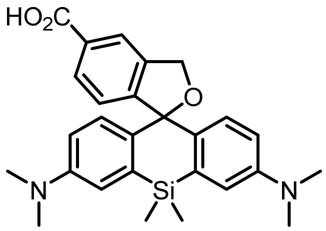

6e

Prepared from **5e** (50 mg, 0.1 mmol) according to conditions A. The product was isolated by preparative HPLC (Interchim Uptisphere Strategy PhC4 250×21.2 mm 5 µm, solvent flow rate 18 mL/min, gradient 20% to 70% A:B, A – acetonitrile + 0.1% (v/v) TFA, B – water + 0.1% (v/v) TFA). Yield 52 mg (91%) as trifluoroacetate salt, blue solid. Known compound (compound **7** in in [21]).

^{1}H NMR (400 MHz, pyridine-*d*$_5$): δ 8.46 (d, J = 1.5 Hz, 1H), 8.37 (dd, J = 7.9, 1.5 Hz, 1H), 7.45 (d, J = 8.9 Hz, 2H), 7.34 (d, J = 7.9 Hz, 1H), 7.21 (d, J = 2.9 Hz, 2H), 6.79 (dd, J = 8.9, 2.9 Hz, 2H), 6.4 (br.s, CO_2H + H_2O), 5.62 (s, 2H), 2.84 (s, 12H), 0.77 (s, 3H), 0.67 (s, 3H).

6f

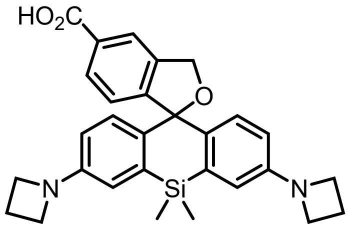

6f

Prepared from **5f** (40 mg, 0.077 mmol) according to conditions A. The product was isolated by preparative HPLC (Interchim Uptisphere Strategy PhC4 250×21.2 mm 5 µm, solvent flow rate 18 mL/min, gradient 30% to 70% A:B, A – acetonitrile + 0.1% (v/v) formic acid, B – water + 0.1% (v/v) formic acid). Yield 18 mg (48%), light blue solid. Known compound (compound **AB640B** in [22]).

^{1}H NMR (400 MHz, $CDCl_3$): δ 8.05 (d, J = 1.1 Hz, 1H), 7.99 (dd, J = 8.0, 1.1 Hz, 1H), 7.11 (d, J = 8.0 Hz, 1H), 6.94 (d, J = 8.6 Hz, 2H), 6.67 (d, J = 2.6 Hz, 2H), 6.33 (dd, J = 8.6, 2.6 Hz, 2H), 5.31 (s, 2H), 3.88 (app.t, J = 7.2 Hz, 8H), 2.35 (app.p, J = 7.2 Hz, 4H), 0.60 (s, 3H), 0.52 (s, 3H).
^{13}C NMR (101 MHz, $CDCl_3$): δ 171.1, 152.6, 150.6, 140.1, 138.5, 135.3, 129.9, 128.7, 128.4, 124.7, 123.6, 115.7, 112.8, 92.8, 72.3, 52.6, 17.1, 0.6, -1.0.
HRMS (ESI) m/z: [M+H]+ Calcd for $C_{29}H_{31}N_2O_3Si$ 483.2098; Found 483.2094.

Synthesis of live-cell fluorescent tubulin probe 4-SiR-CTX (8) by carbonylative amination

7a and 7b

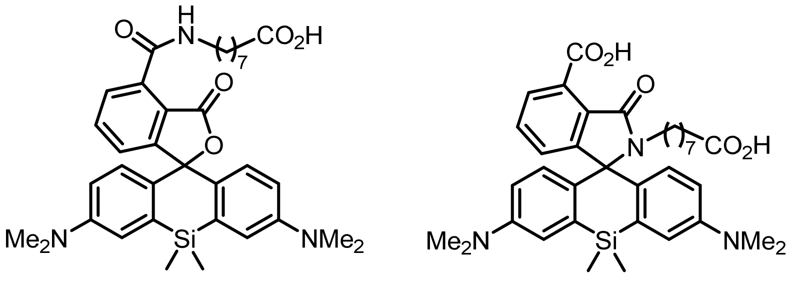

7a **7b**

A dried small two-chamber Skrydstrup reactor (total inner volume 20 mL, available from Sigma-Aldrich as "COware gas reactor", Cat. No. STW1-1EA) was equipped with stirring bars in both chambers. In the chamber A, $Mo(CO)_6$ (106 mg, 0.4 mmol) was placed, and the chamber B was loaded with **3j** (51 mg, 0.1 mmol), *tert*-butyl 8-aminooctanoate (43 mg, 0.2 mmol, 2 eq; known compound: compound **SI-10** in [10]) and Xantphos Pd G4 catalyst (9.6 mg, 0.01 mmol, 10 mol%). The reactor was flushed with argon, and anhydrous 1,4-dioxane was added into both chambers (0.7 mL each). The reactor was sealed, and after briefly degassing the reaction mixture, 1,8-diazabicyclo[5.4.0]undec-7-ene (DBU, 180 µL) was injected into the chamber A and *N*-ethyldiisopropylamine (DIPEA, 52 µL, 0.3 mmol, 3 eq) was injected into the chamber B. The reactor was immersed into an oil bath preheated to 80 °C and the contents were stirred overnight (15 h). The reaction mixture from the chamber B was diluted with acetonitrile and filtered through a plug of Celite, washing with acetonitrile, EtOAc and CH_2Cl_2. The filtrate was concentrated and transferred into a 10 mL round bottom flask, where it was evaporated to dryness and redissolved in CH_2Cl_2 (1.5 mL). Trifluoroacetic acid (TFA, 0.5 mL) was added, and the reaction mixture was stirred at rt for 1.5 h. Afterwards, the solution was diluted with toluene (4 mL), evaporated to dryness, the residue was dissolved in minimal volume of DMSO-acetonitrile, microfiltered through a 0.2 µm PTFE membrane filter, and the product were isolated by preparative HPLC (Interchim Uptisphere Strategy PhC4 250×21.2 mm 5 µm, solvent flow rate 18 mL/min, gradient 30% to 70% A:B, A – acetonitrile + 0.1% (v/v) TFA, B – water + 0.1% (v/v) TFA).

Compound **7a** (4-SiR-C_8-acid, major): yield 41 mg (67%).

^{1}H NMR (400 MHz, DMSO-*d*$_6$): δ 9.00 (t, J = 5.5 Hz, 1H), 7.81 – 7.72 (m, 2H), 7.26 (dd, J = 6.2, 2.5 Hz, 1H), 7.06 (d, J = 2.7 Hz, 2H), 6.74 (d, J = 8.9 Hz, 2H), 6.69 (dd, J = 8.9, 2.7 Hz, 2H),

3.31 (q, J = 6.6 Hz, 2H), 2.94 (s, 12H), 2.20 (t, J = 7.4 Hz, 2H), 1.64 – 1.45 (m, 4H), 1.44 – 1.20 (m, 6H), 0.63 (s, 3H), 0.53 (s, 3H).
^{13}C NMR (101 MHz, DMSO-d_6): δ 174.6, 169.2, 164.7, 155.5, 149.0, 135.9, 129.0, 128.1, 121.4, 117.1, 116.8, 114.1, 90.2, 33.7, 28.9, 28.6, 26.5, 24.6, 0.0, -1.1.
HRMS (ESI) m/z: [M+H]+ Calcd for $C_{35}H_{44}N_3O_5Si$ 614.3045; Found 614.3041.

Compound **7b** (lactam byproduct, minor): yield 18 mg (29%).
^{1}H NMR (400 MHz, DMSO-d_6): δ 8.09 (dd, J = 7.7, 1.0 Hz, 1H), 7.63 (t, J = 7.7 Hz, 1H), 7.07 (dd, J = 7.7, 1.0 Hz, 1H), 6.98 (d, J = 2.9 Hz, 2H), 6.71 (dd, J = 9.1, 2.9 Hz, 2H), 6.54 (d, J = 9.1 Hz, 2H), 3.01 (t, J = 7.5 Hz, 2H), 2.92 (s, 12H), 2.08 (t, J = 7.4 Hz, 2H), 1.33 (p, J = 7.4 Hz, 2H), 1.14 – 0.89 (m, 8H), 0.59 (s, 3H), 0.53 (s, 3H).
^{13}C NMR (101 MHz, DMSO-d_6): δ 174.5, 169.5, 165.0, 156.7, 148.6, 135.2, 133.7, 131.6, 129.1, 128.5, 127.6, 127.3, 115.8, 115.4, 73.7, 41.5, 40.0, 33.6, 28.2, 28.0, 26.4, 26.2, 24.4, -0.0, -0.2.
HRMS (ESI) m/z: [M+H]+ Calcd for $C_{35}H_{44}N_3O_5Si$ 614.3045; Found 614.3040.

4-SiR-CTX (8)

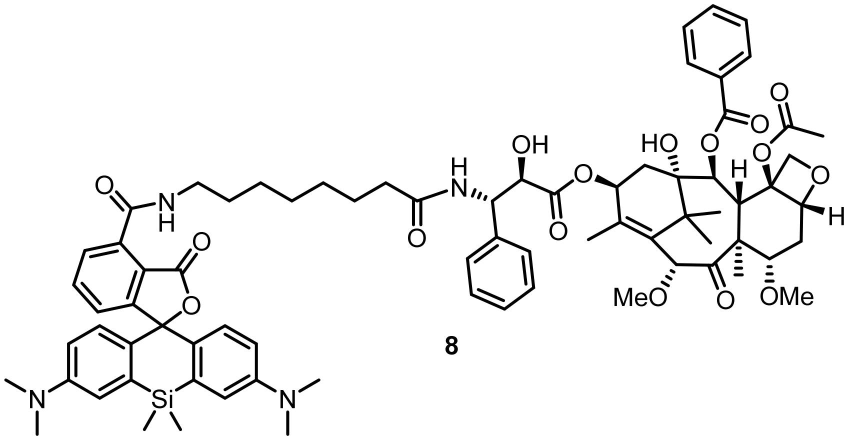

8

In a 4 mL vial, equipped with a stirring bar, 1-[bis(dimethylamino)methylene]-1*H*-1,2,3-triazolo[4,5-*b*]pyridinium 3-oxid hexafluorophosphate (HATU; 15 mg in 50 µL DMF, 39 µmol, 1.5 eq) was added to the mixture of **7a** (16 mg, 26 µmol), H_2N-CTX·HCO_2H (25 mg, 31 µmol, 1.2 eq; known compound: compound **3'-H$_2$NMe$_2$XT·HCO$_2$H** in [23]) and *N*-ethyldiisopropylamine (DIPEA; 100 µL) in DMF (200 µL). The reaction mixture was vigorously stirred at rt for 1.5 h, at which point LC-MS analysis of a sample showed complete conversion of **7a**. The solvents were removed *in vacuo* at rt, and the product was isolated by preparative HPLC (Interchim Uptisphere Strategy PhC4 250×21.2 mm 5 µm, solvent flow rate 18 mL/min, gradient 50% to 90% A:B, A – acetonitrile + 0.1% (v/v) formic acid, B – water + 0.1% (v/v) formic acid). Yield 19 mg (55%) of bluish solid. Known compound (compound **31** in [20]).

^{1}H NMR (400 MHz, DMSO-d_6): δ 9.02 (t, J = 5.5 Hz, 1H), 8.38 (d, J = 9.1 Hz, 1H), 8.01 – 7.95 (m, 2H), 7.77 – 7.74 (m, 2H), 7.70 – 7.64 (m, 1H), 7.62 – 7.56 (m, 2H), 7.41 – 7.28 (m, 4H), 7.27 – 7.18 (m, 2H), 7.00 (d, J = 2.8 Hz, 2H), 6.70 (dd, J = 9.0, 1.2 Hz, 2H), 6.64 (ddd, J = 9.0, 2.8, 1.6 Hz, 2H), 5.99 – 5.90 (m, 2H), 5.39 (d, J = 7.1 Hz, 1H), 5.29 (dd, J = 9.0, 5.7 Hz, 1H), 4.95 (dd, J = 10.0, 1.9 Hz, 1H), 4.71 (s, 1H), 4.65 (s, 1H), 4.43 (dd, J = 6.9, 6.0 Hz, 1H), 4.02 (s, 2H), 3.76 (dd, J = 10.6, 6.6 Hz, 1H), 3.63 (d, J = 7.1 Hz, 1H), 3.32 – 3.27 (m, 2H), 3.30 (s, 3H), 3.21 (s, 3H), 2.92 (s, 12H), 2.72 – 2.59 (m, 1H), 2.25 (s, 3H), 2.18 (t, J = 7.4 Hz, 2H), 1.97 (dd, J = 15.4, 9.2 Hz, 1H), 1.91 – 1.80 (m, 1H), 1.83 (s, 3H), 1.60 – 1.44 (m, 6H), 1.40 – 1.17 (m, 8H), 1.03 (s, 3H), 0.97 (s, 3H), 0.62 (s, 3H), 0.52 (s, 3H).

Supplementary references

[1] Butkevich, A. N.; Mitronova, G. Y.; Sidenstein, S. C.; Klocke, J. L.; Kamin, D.; Meineke, D. N. H.; D'Este, E.; Kraemer, P.-T.; Danzl, J. G.; Belov, V. N.; Hell, S. W. Fluorescent rhodamines and fluorogenic carbopyronines for super-resolution STED microscopy in living cells. *Angew. Chem. Int. Ed.* **2016**, *55*, 3290-3294.

[2] Kuila, D. P.; Lahiri, S. C. Comparison of the macroscopic molecular properties in understanding the structural aspects of mixed aquo-organic binary mixtures. *Z. Phys. Chem.* **2004**, *218(7)*, 803-828.

[3] Grimm, J. B.; Muthusamy, A. K.; Liang, Y.; Brown, T. A.; Lemon, W. C.; Patel, R.; Lu, R.; Macklin, J. J.; Keller, P. J.; Ji, N.; Lavis, L. D. A general method to fine-tune fluorophores for live-cell and in vivo imaging. *Nat. Methods* **2017**, *14*, 987-994.

[4] Sheppard, W. A. *m*-Trifluoromethyl-*N,N*-dimethylaniline. *Org. Synth.* **1969**, *49*, 111.

[5] Lukhtanov, E. A. Preparation of carborhodamine compounds for oligonucleotides-carborhodamine conjugates useful as nucleic acid probes. PCT Int. Appl. **2019**, WO 2019231617 A1.

[6] Koide, Y.; Urano, Y.; Hanaoka, K.; Piao, W.; Kusakabe, M.; Saito, N.; Terai, T.; Okabe, T.; Nagano, T. Development of NIR fluorescent dyes based on Si-rhodamine for in vivo imaging. *J. Am. Chem. Soc.* **2012**, *134(11)*, 5029-5031.

[7] Woll, M. G.; Qi, H.; Turpoff, A.; Zhang, N.; Zhang, X.; Chen, G.; Li, C.; Huang, S.; Yang, T.; Moon, Y.-C.; Lee, C.-S.; Choi, S.; Almstead, N. G.; Naryshkin, N. A.; Dakka, A.; Narasimhan, J.; Gabbeta, V.; Welch, E.; Zhao, X.; Risher, N.; Sheedy, J.; Weetall, M.; Karp, G. M. Discovery and optimization of small molecule splicing modifiers of Survival Motor Neuron 2 as a treatment for spinal muscular atrophy. *J. Med. Chem.* **2016**, *59(13)*, 6070-6085.

[8] Liu, X.-G.; Zhang, S.-S.; Jiang, C.-Y.; Wu, J.-Q.; Li, Q.; Wang, H. Cp*Co(III)-catalyzed annulations of 2-alkenylphenols with CO: mild access to coumarin derivatives. *Org. Lett.* **2015**, *17(21)*, 5404-5407.

[9] Garazd, M. M.; Garazd, Ya. L.; Shilin, S. V.; Panteleimonova, T. N.; Khilya, V. P. Modified coumarins. 4. Synthesis and biological properties of cyclopentane-annelated furocoumarins. *Chem. Nat. Compd.* **2002**, *38(3)*, 230-242.

[10] Butkevich, A. N.; Lukinavičius, G.; d'Este, E.; Hell, S. W. Cell-permeant large Stokes shift dyes for transfection-free multicolor nanoscopy. *J. Am. Chem. Soc.* **2017**, *139(36)*, 12378-12381.

[11] Frei, M. S.; Hoess, P.; Lampe, M.; Nijmeijer, B.; Kueblbeck, M.; Ellenberg, J.; Wadepohl, H.; Ries, J.; Pitsch, S.; Reymond, L.; Johnsson, K. Photoactivation of silicon rhodamines via a light-induced protonation. *Nat. Commun.* **2019**, *10*, 4580.

[12] Huang, Y.-L.; Walker, A. S.; Miller, E. W. A photostable silicon rhodamine platform for optical voltage sensing. *J. Am. Chem. Soc.* **2015**, *137(33)*, 10767-10776.

[13] Liang, Z.; Tang, Q.; Liu, J.; Li, J.; Yan, F.; Miao, Q. N-Type organic semiconductors based on π-deficient pentacenequinones: synthesis, electronic structures, molecular packing, and thin film transistors. *Chem. Mater.* **2010**, *22(23)*, 6438-6443.
[14] Grimm, J. B.; Brown, T. A.; Tkachuk, A. N.; Lavis, L. D. General synthetic method for Si-fluoresceins and Si-rhodamines. *ACS Cent. Sci.* **2017**, *3(9)*, 975-985.
[15] Fischer, C.; Sparr, C. Direct transformation of esters into heterocyclic fluorophores. *Angew. Chem. Int. Ed.* **2018**, *57*, 2436-2440.
[16] Pastierik, T.; Šebej, P.; Medalová, J.; Štacko, P.; Klán, P. Near-infrared fluorescent 9-phenylethynylpyronin analogues for bioimaging. *J. Org. Chem.* **2014**, *79*, 3374-3382.
[17] Halabi, E. A.; Pinotsi, D.; Rivera-Fuentes, P. Photoregulated fluxional fluorophores for live-cell super-resolution microscopy with no apparent photobleaching. *Nat. Commun.* **2019**, *10*, 1232.
[18] Butkevich, A. N.; Belov, V. N.; Kolmakov, K.; Sokolov, V. V.; Shojaei, H.; Sidenstein, S. C.; Kamin, D.; Matthias, J.; Vlijm, R.; Engelhardt, J.; Hell, S. W. Hydroxylated fluorescent dyes for live-cell labeling: synthesis, spectra and super-resolution STED microscopy. *Chem. Eur. J.* **2017**, *23*, 12114-12119.
[19] Korsager, S.; Taaning, R. H.; Skrydstrup, T. Effective palladium-catalyzed hydroxycarbonylation of aryl halides with substoichiometric carbon monoxide. *J. Am. Chem. Soc.* **2013**, *135*, 2891-2894.
[20] Bucevičius, J.; Kostiuk, G.; Gerasimaitė, R.; Gilat, T.; Lukinavičius, G. Enhancing the biocompatibility of rhodamine fluorescent probes by a neighbouring group effect. *Chem. Sci.* **2020**, *11*, 7313-7323.
[21] Uno, S.; Kamiya, M.; Yoshihara, T.; Sugawara, K.; Okabe, K.; Tarhan, M. C.; Fujita, H.; Funatsu, T.; Okada, Y.; Tobita, S.; Urano, Y. A spontaneously blinking fluorophore based on intramolecular spirocyclization for live-cell super-resolution imaging. *Nat. Chem.* **2014**, *6*, 681-689.
[22] Xu, Z.; Liu, X.; Qiao, Q.; Li, J. Preparation of rhodamine-based heteroaryl compounds as full-spectrum photo-switch molecules. Patent CN 111333612 A, **2020**.
[23] Butkevich, A. N.; Ta, H.; Ratz, M.; Stoldt, S.; Jakobs, S.; Belov, V. N.; Hell, S. W. Two-color 810 nm STED nanoscopy of living cells with endogenous SNAP-tagged fusion proteins. *ACS Chem. Biol.* **2018**, *13(2)*, 475-480.

NMR spectra

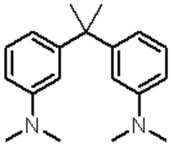

S1

^{1}H (400 MHz, $CDCl_3$)

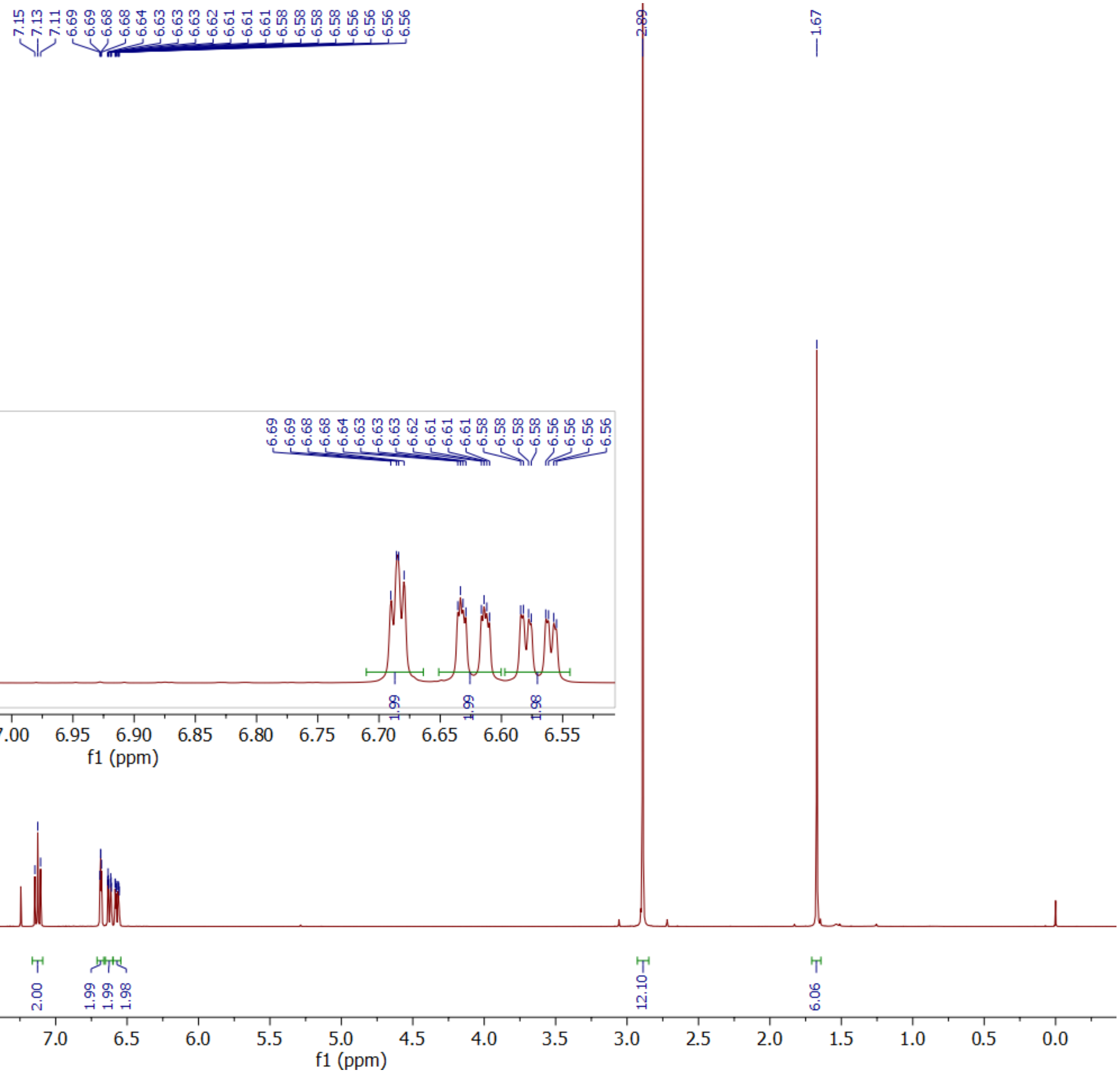

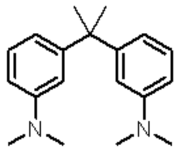

S1

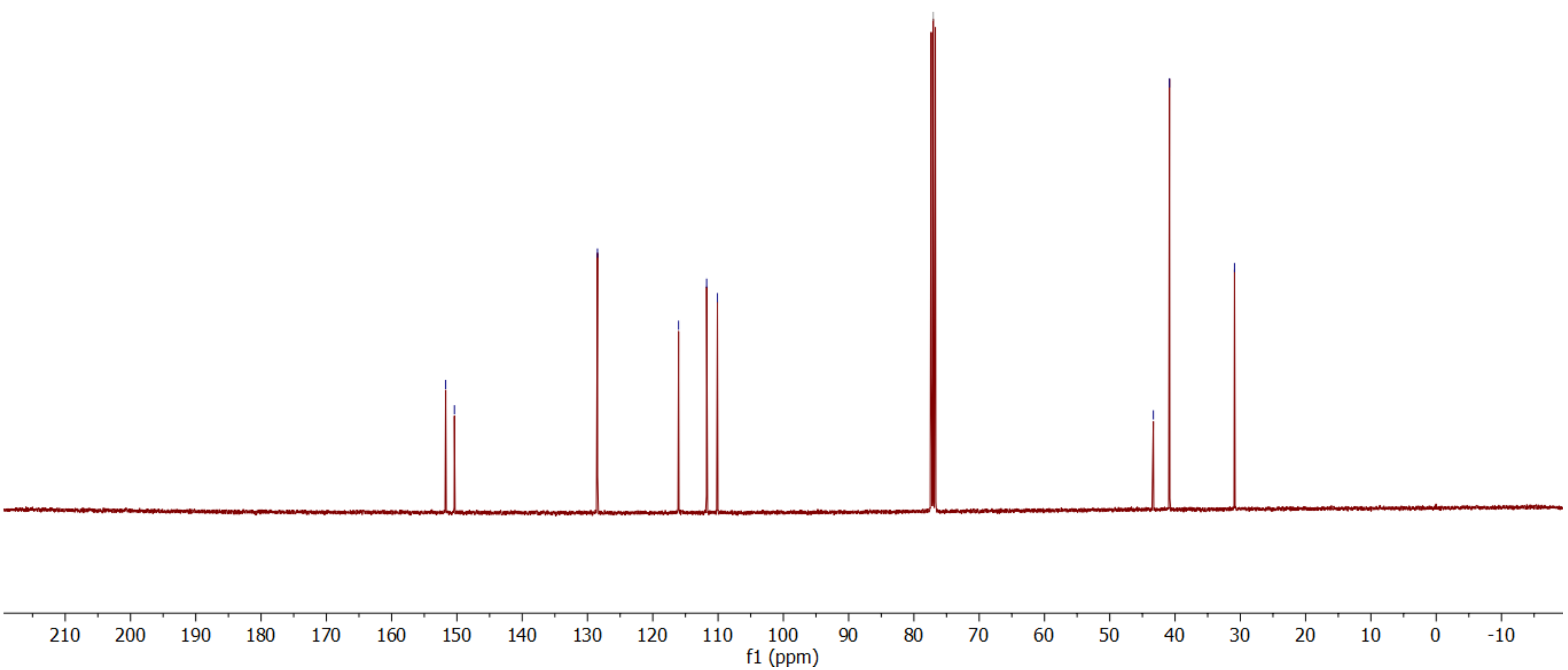

¹³C (101 MHz, CDCl₃)
151.72
150.38
128.47
116.05
111.75
110.10
77.04 CDCl3
43.32
40.86
30.88
210
200
190
180
170
160
150
140
130
120
110
100
90
80
70
60
50
40
30
20
10
0
-10
f1 (ppm)

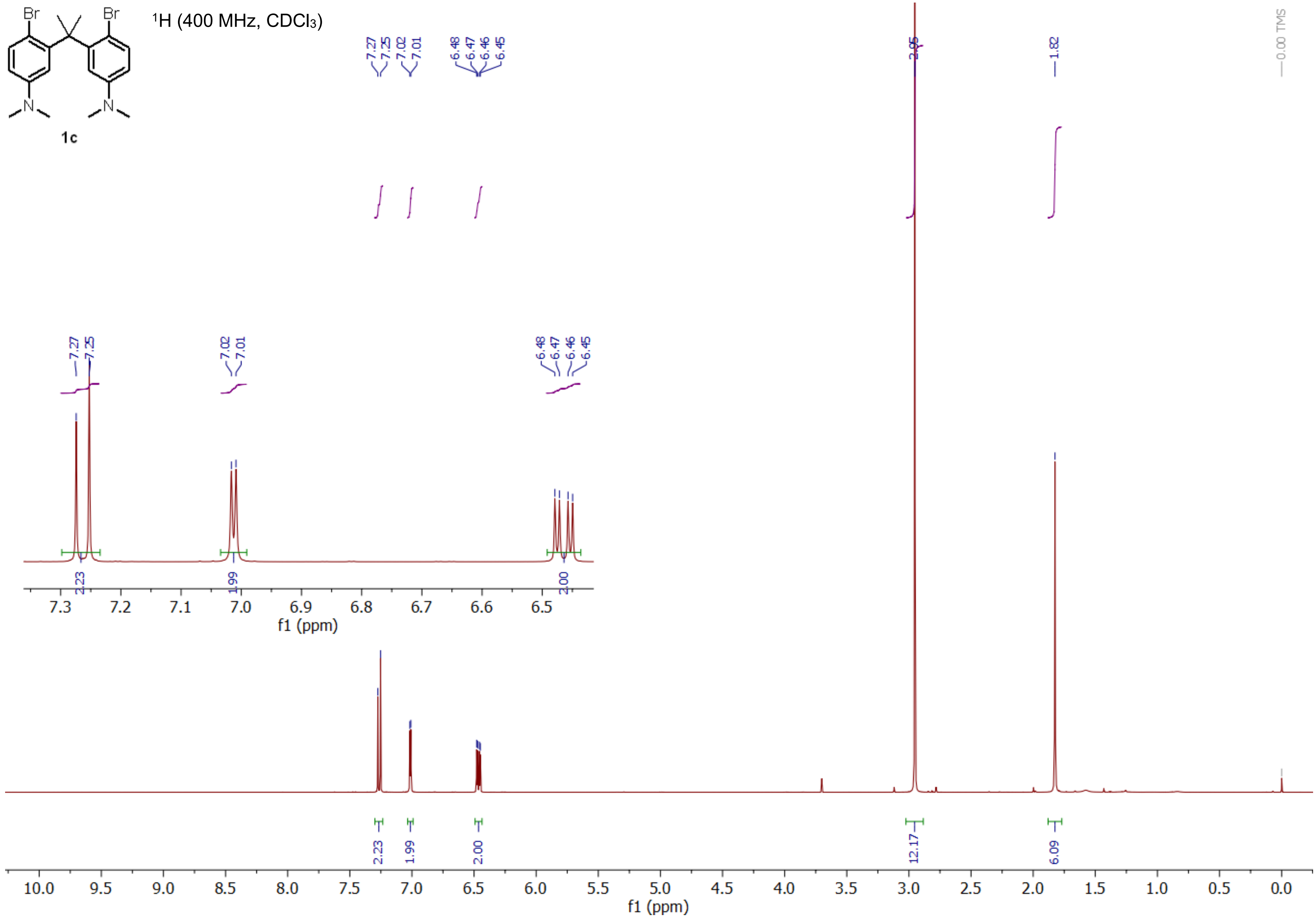
Br
Br
N
N
1c
¹H (400 MHz, CDCl₃)
7.27
7.25
7.02
7.01
6.48
6.47
6.46
6.45
2.95
1.82
0.00 TMS
2.23
1.99
2.00
12.17
6.09
f1 (ppm)

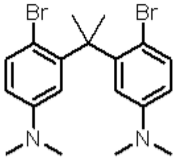
Br
Br
N
N

1c

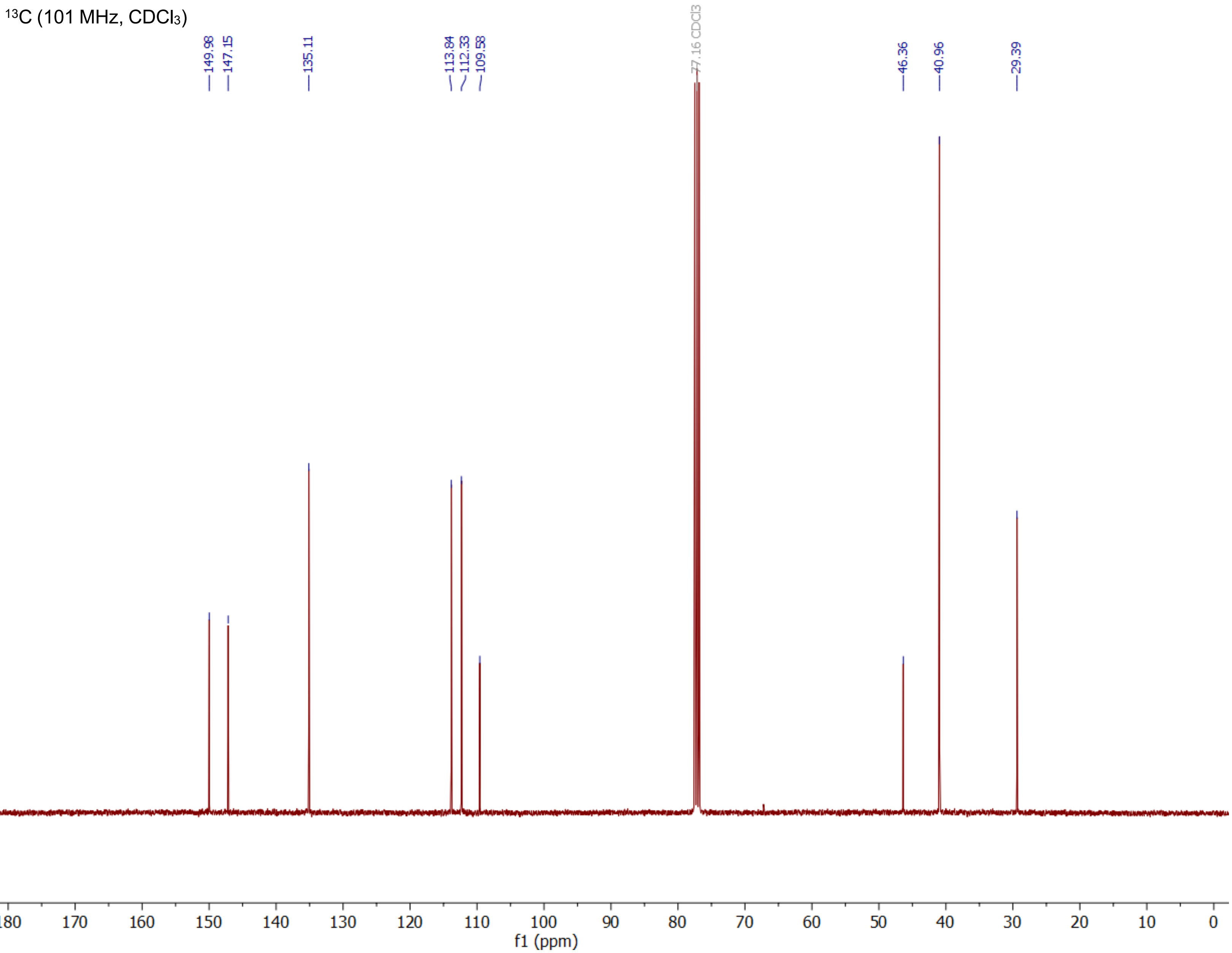
13C (101 MHz, CDCl3)
149.98
147.15
135.11
113.84
112.33
109.58
77.16 CDCl3
46.36
40.96
29.39
200
190
180
170
160
150
140
130
120
110
100
90
80
70
60
50
40
30
20
10
0
f1 (ppm)

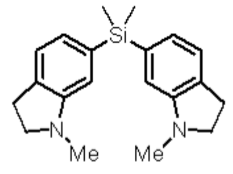

S2

^{1}H (400 MHz, CDCl$_3$)

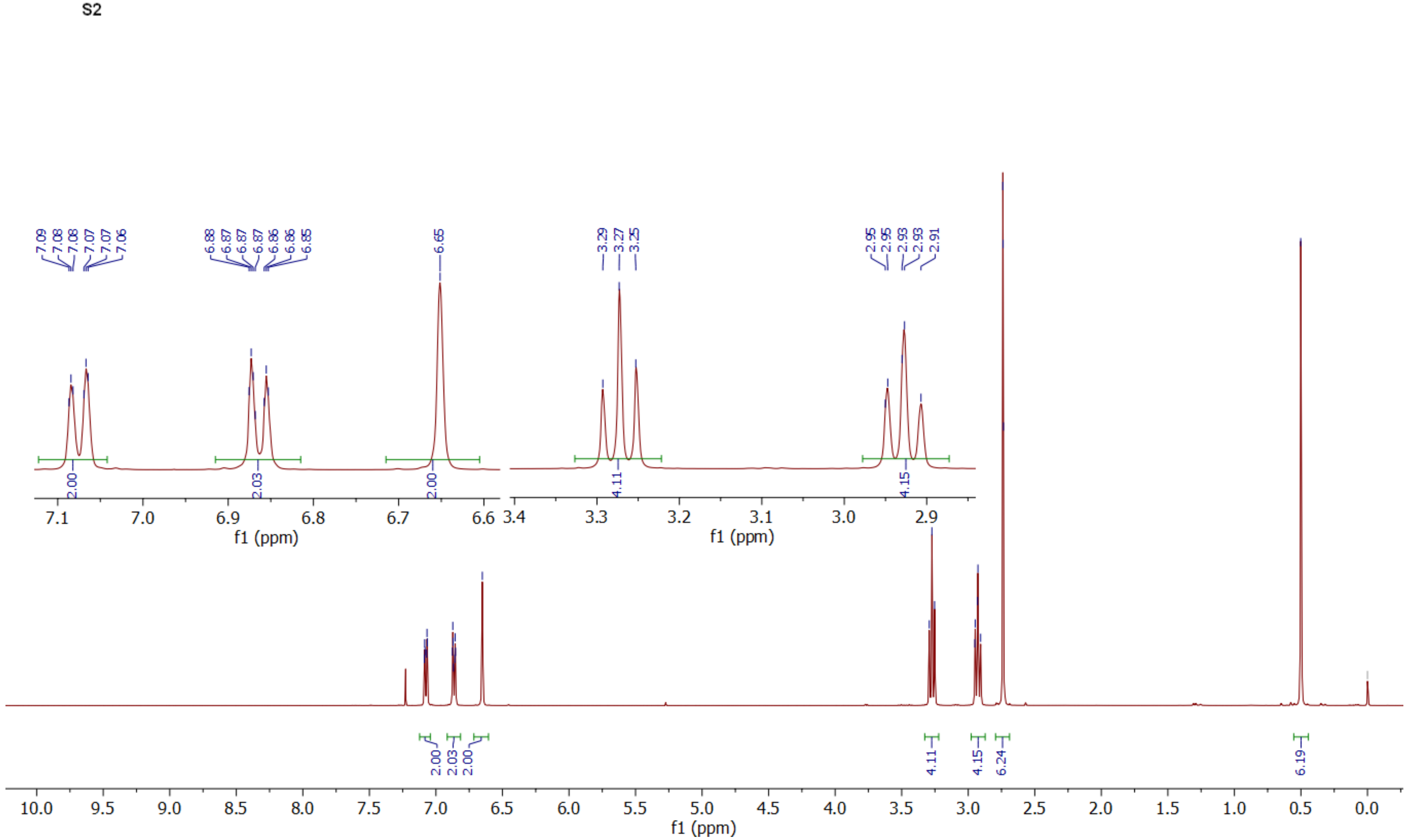

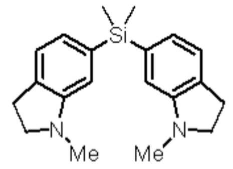

S2

^{13}C (101 MHz, $CDCl_3$)

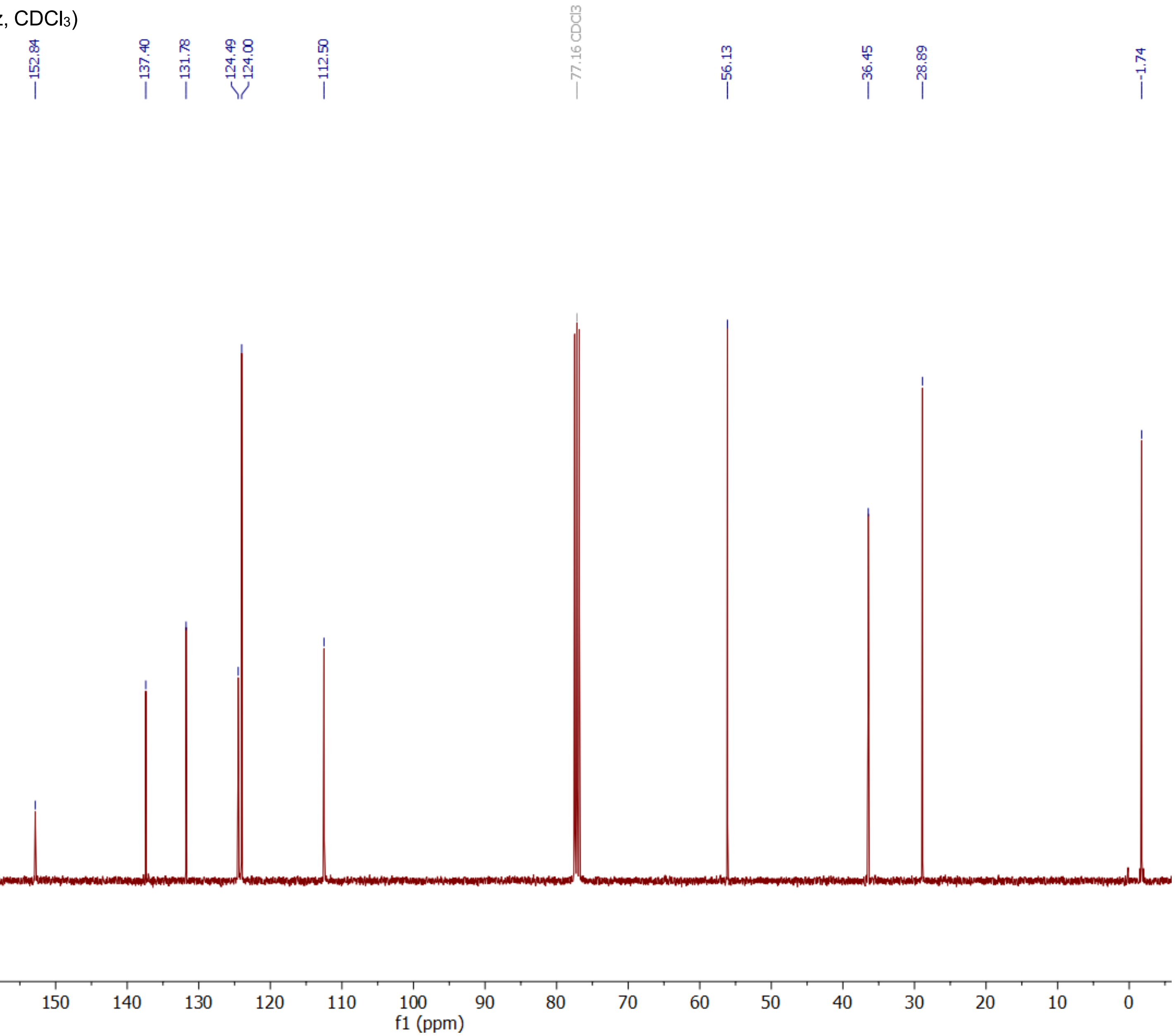

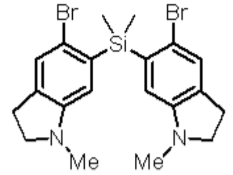

1d

^{1}H (400 MHz, $CDCl_3$)

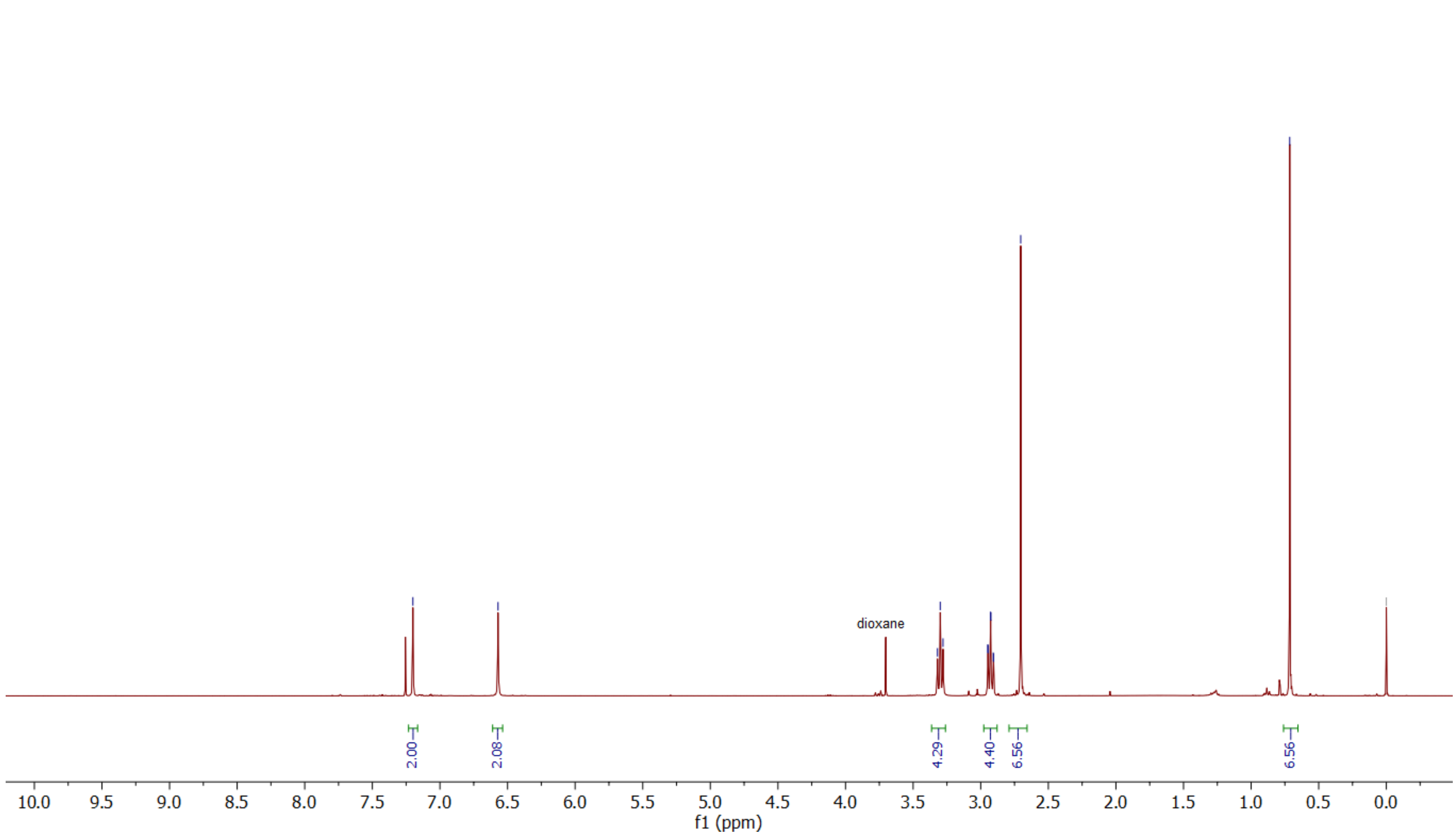

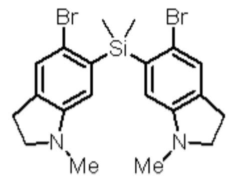

1d

^{13}C (101 MHz, $CDCl_3$)

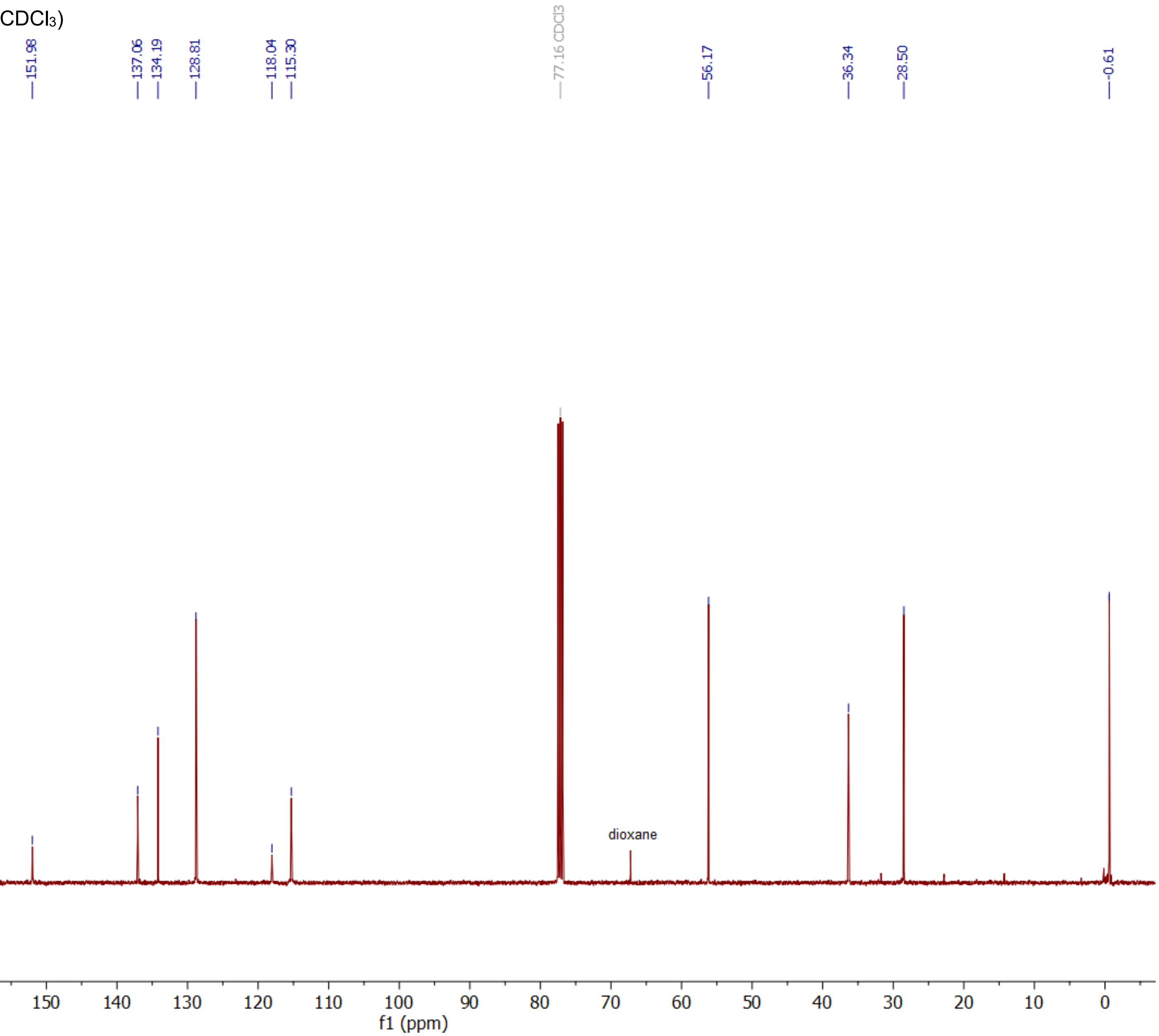

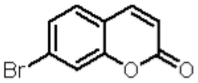

4k

^{1}H (400 MHz, $CDCl_3$)

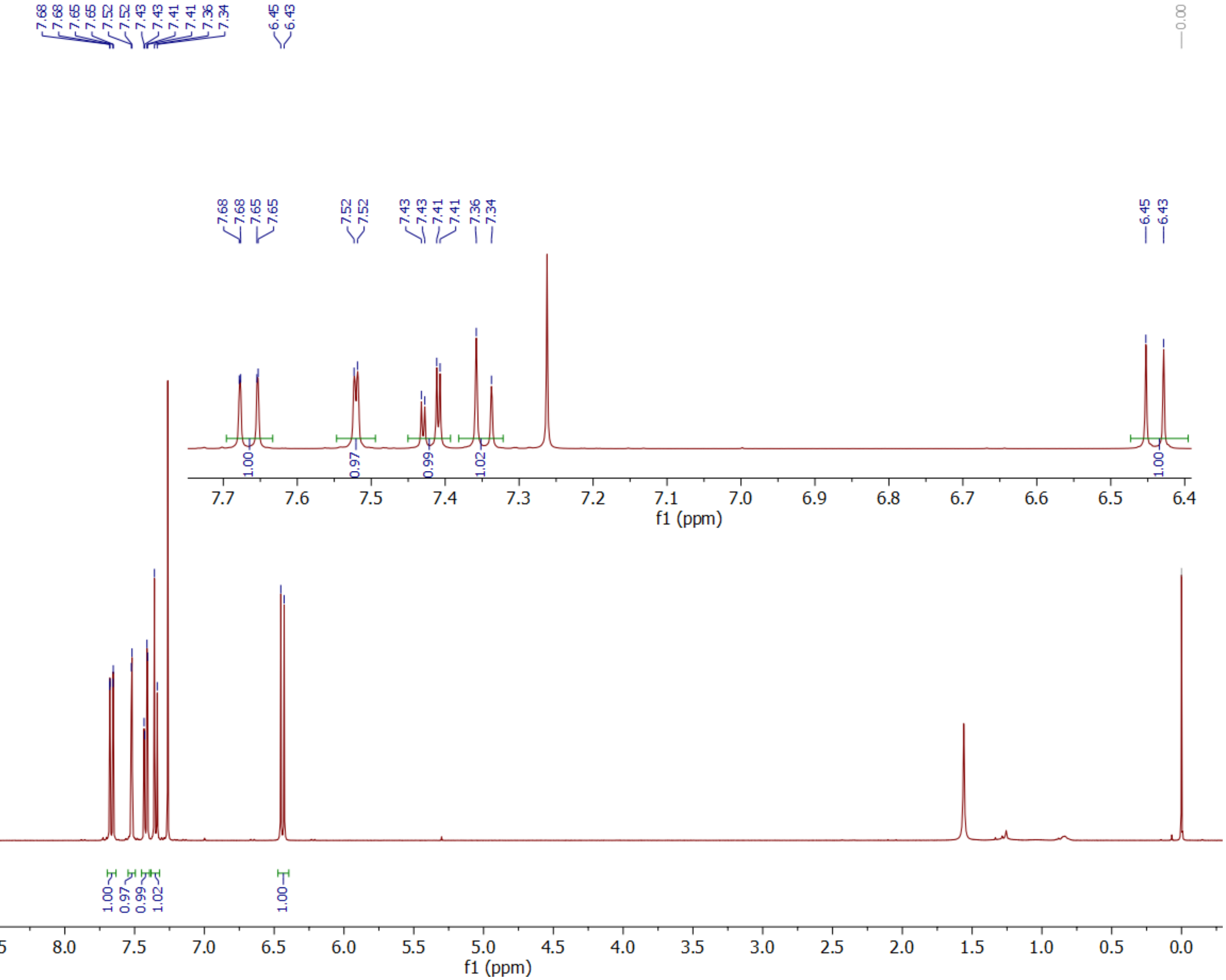

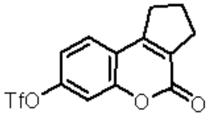

S3

^{1}H (400 MHz, $CDCl_3$)

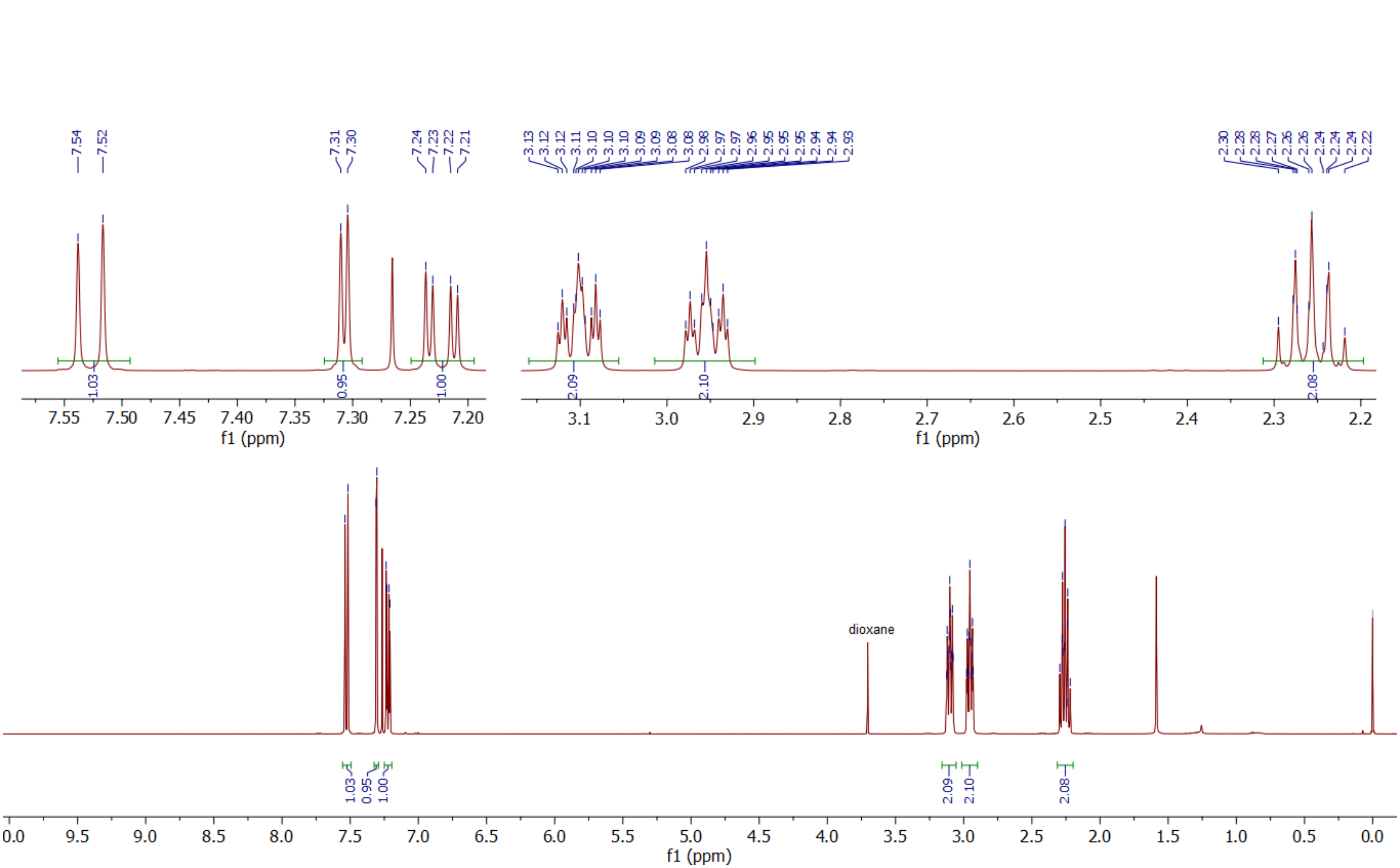

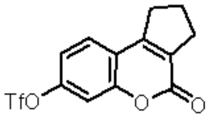

S3

^{19}F (376 MHz, $CDCl_3$)

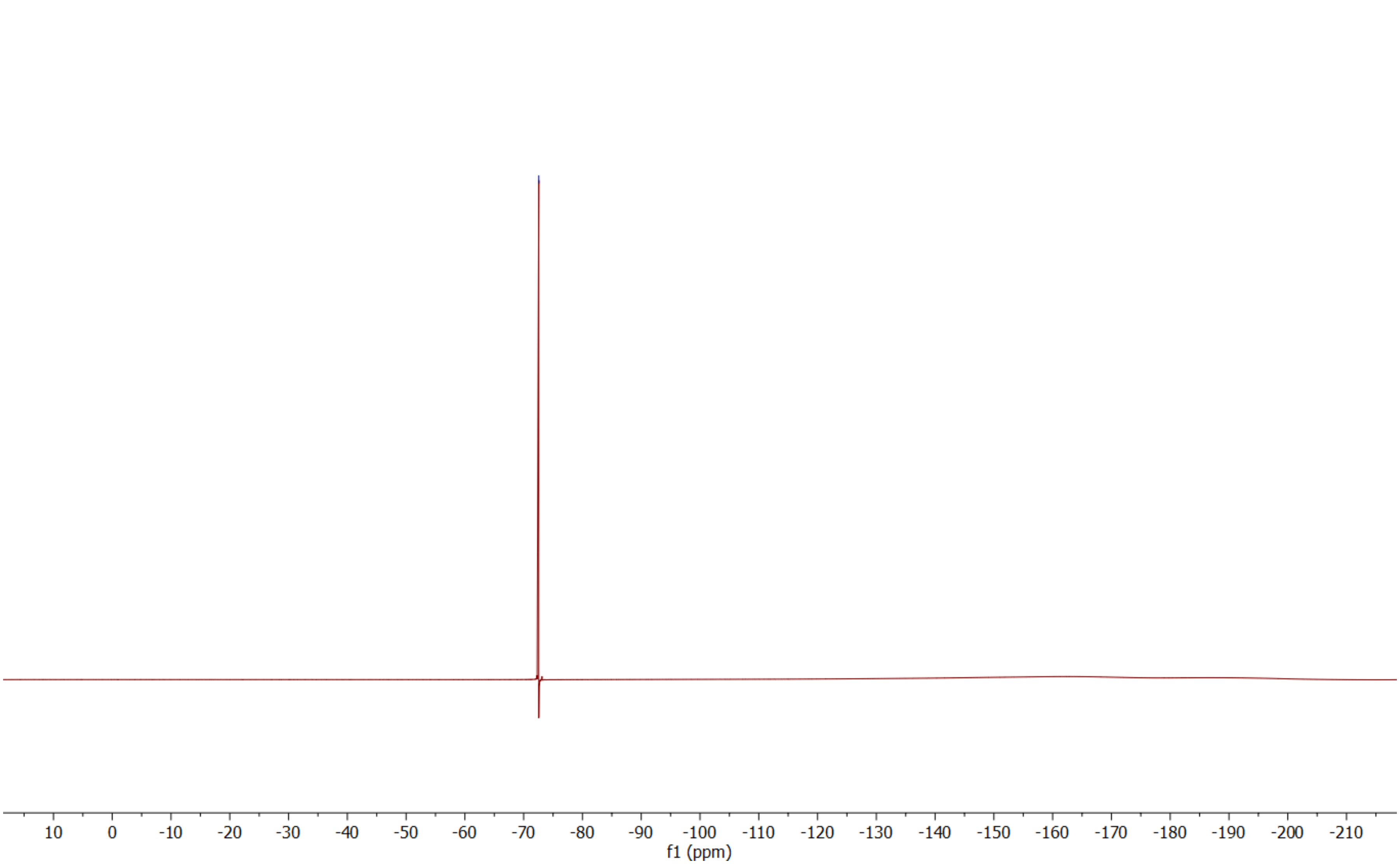

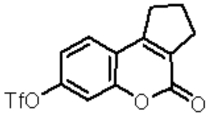

S3

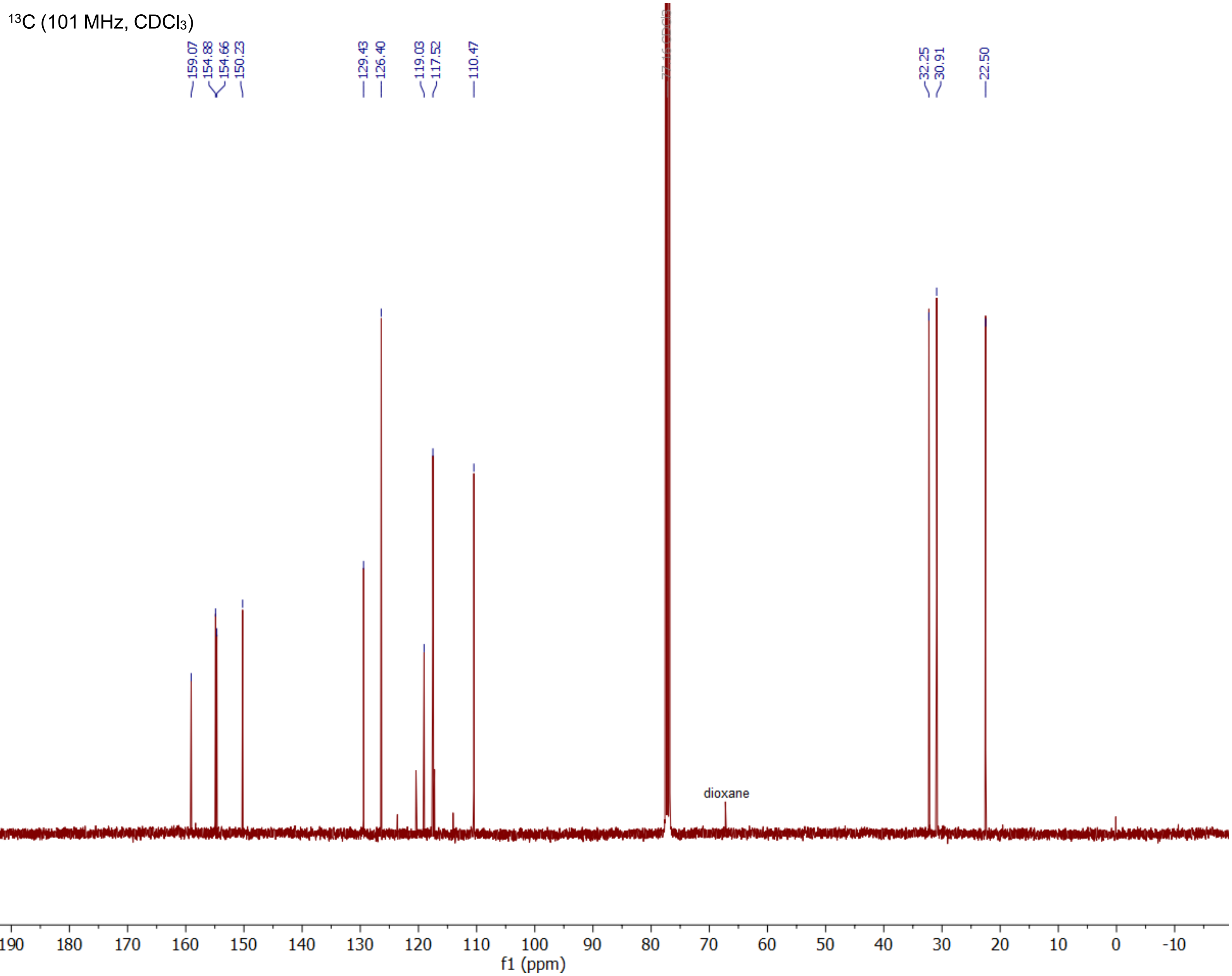
13C (101 MHz, CDCl3)
159.07
154.88
154.66
150.23
129.43
126.40
119.03
117.52
110.47
32.25
30.91
22.50
dioxane
210
200
190
180
170
160
150
140
130
120
110
100
90
80
70
60
50
40
30
20
10
0
-10
f1 (ppm)

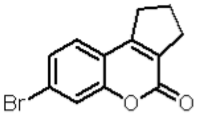

4l

^{1}H (400 MHz, $CDCl_3$)

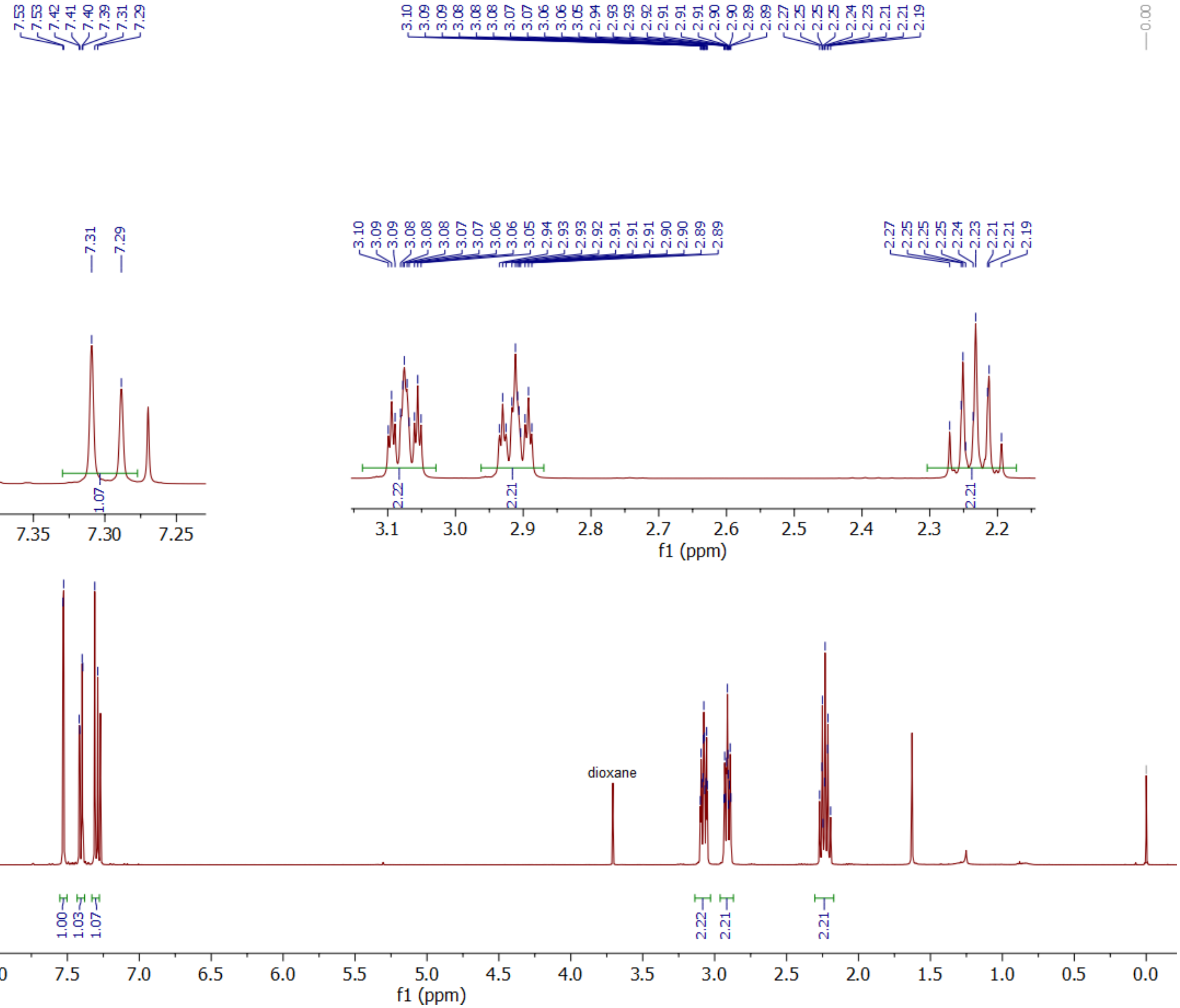

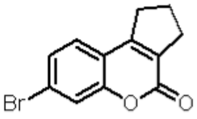

4l

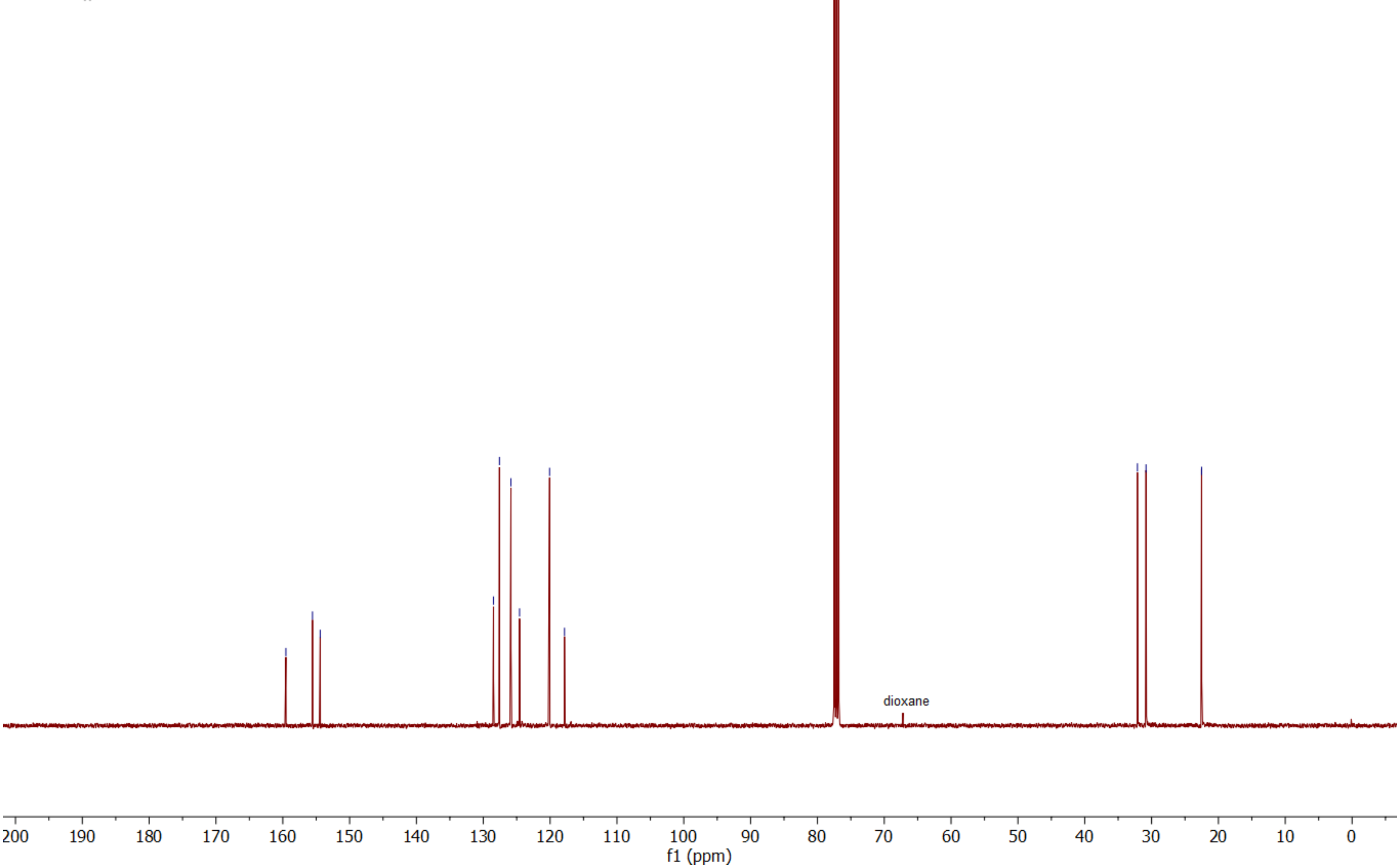

13C (101 MHz, CDCl3)
159.53
155.56
154.40
128.47
127.58
125.86
124.57
120.08
117.85
77.16 CDCl3
32.12
30.83
22.51
dioxane
200
190
180
170
160
150
140
130
120
110
100
90
80
70
60
50
40
30
20
10
0
f1 (ppm)

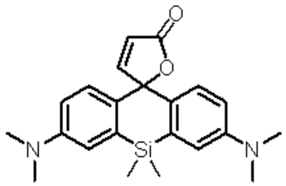

O
O
Si
N
N

3a

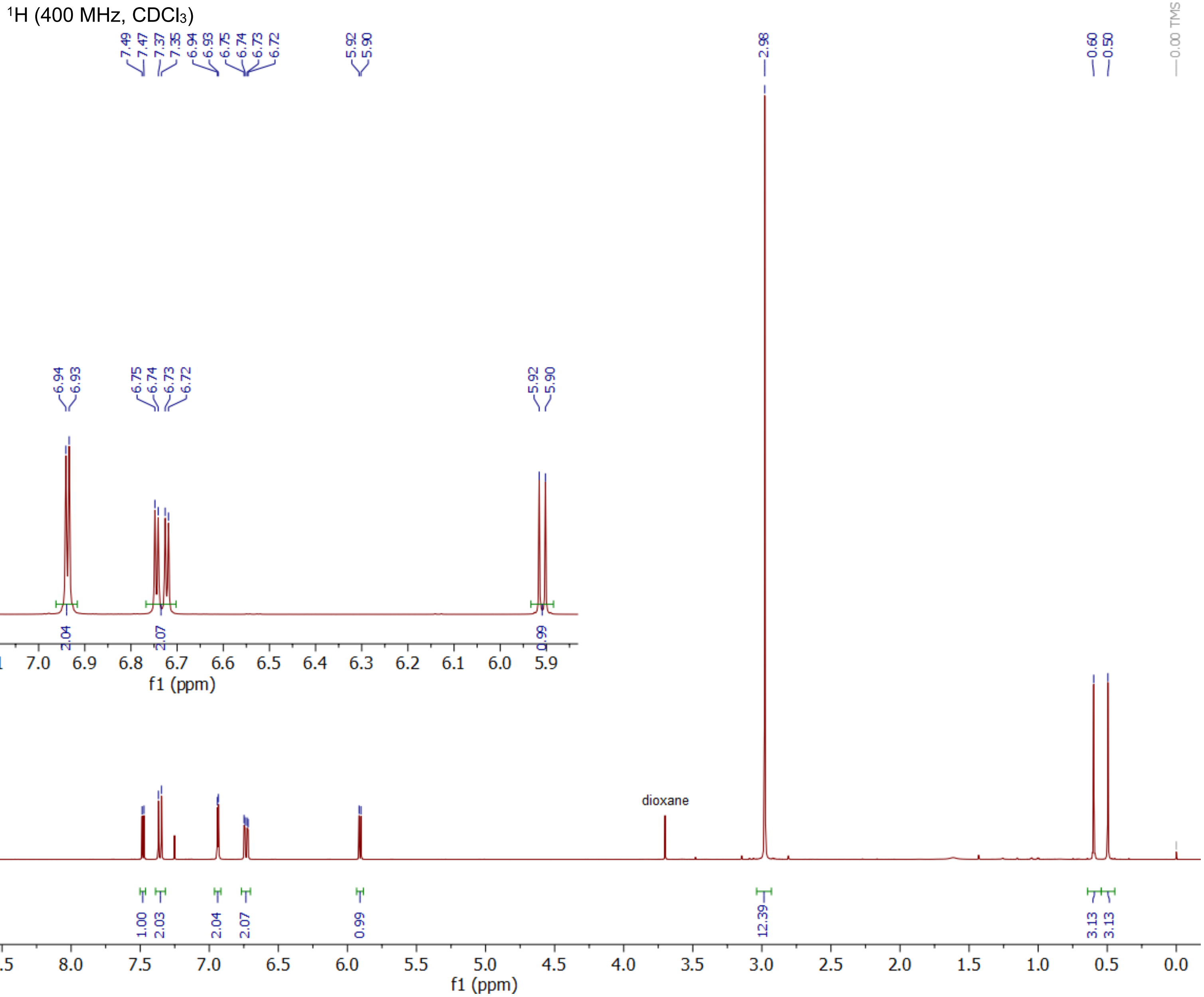

^{1}H (400 MHz, CDCl3)
7.49
7.47
7.37
7.35
6.94
6.93
6.75
6.74
6.73
6.72
5.92
5.90
2.98
0.60
0.50
0.00 TMS
dioxane
1.00
2.03
2.04
2.07
0.99
12.39
3.13
3.13
f1 (ppm)

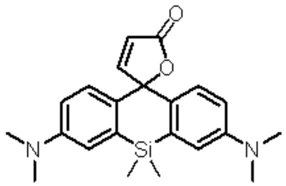

3a

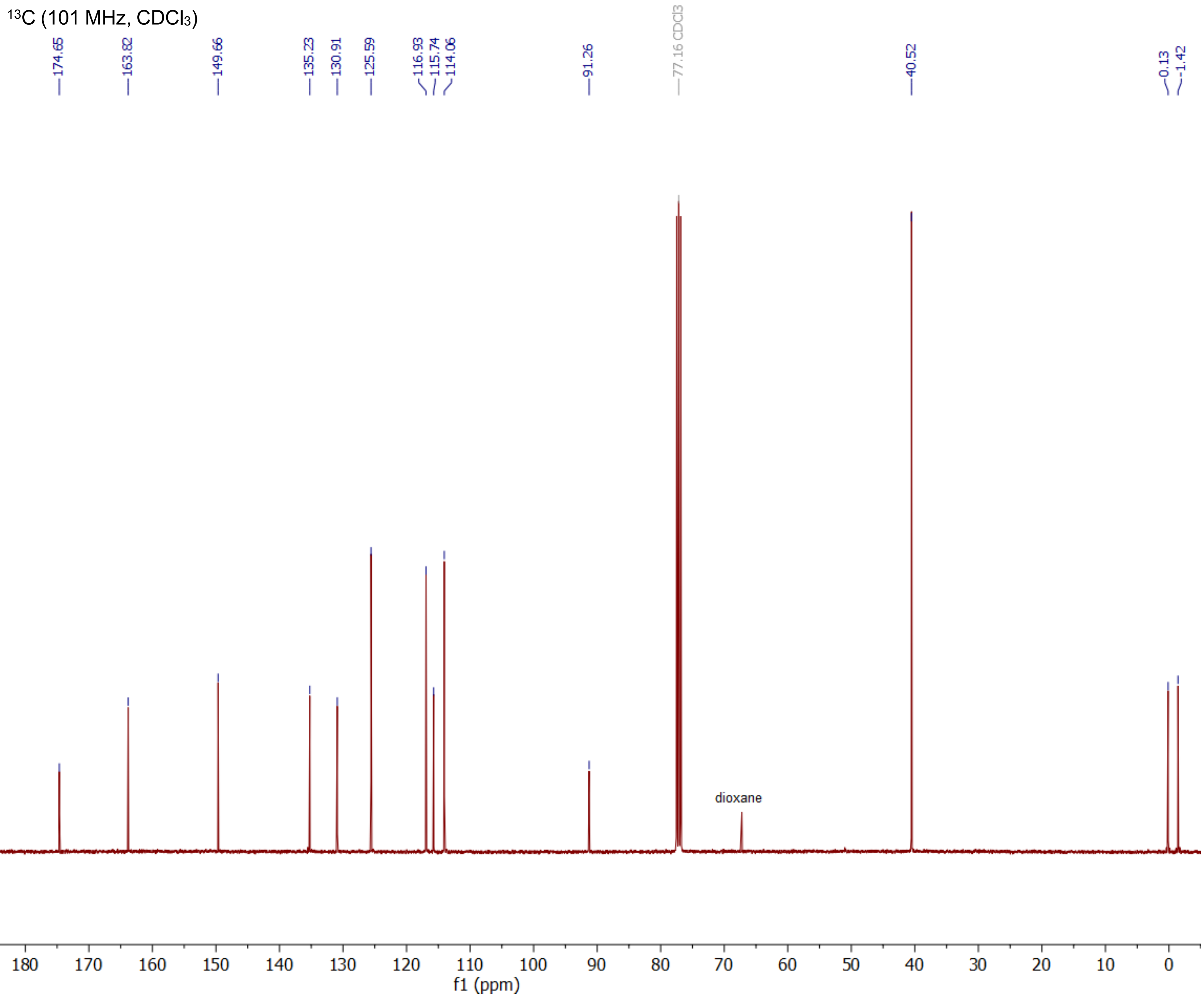

13C (101 MHz, CDCl3)
174.65
163.82
149.66
135.23
130.91
125.59
116.93
115.74
114.06
91.26
77.16 CDCl3
40.52
0.13
-1.42
dioxane
210
200
190
180
170
160
150
140
130
120
110
100
90
80
70
60
50
40
30
20
10
0
f1 (ppm)

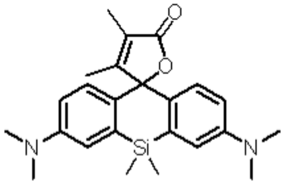

3b

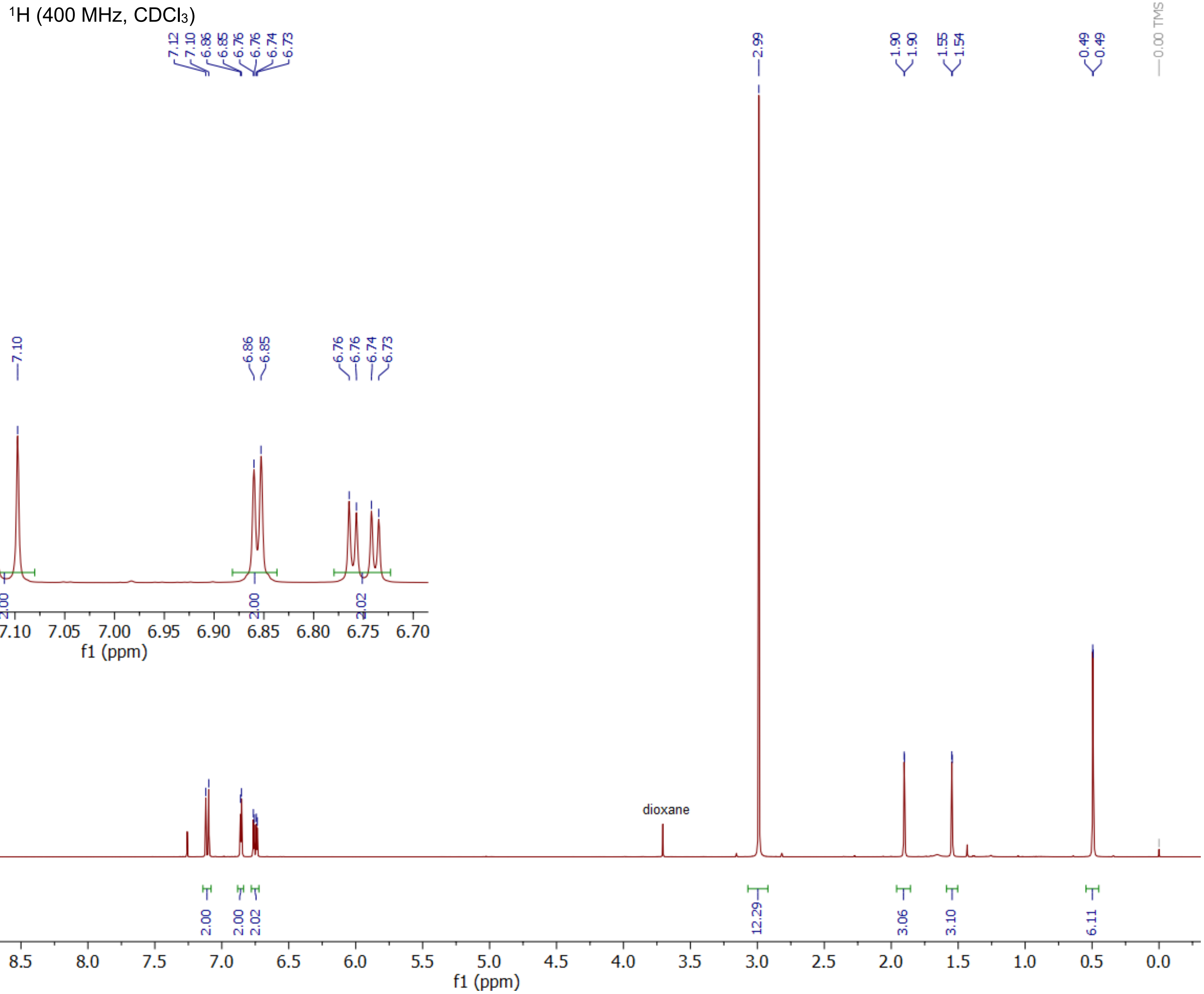

¹H (400 MHz, CDCl3)
7.12
7.10
6.86
6.85
6.76
6.76
6.74
6.73
2.99
1.90
1.90
1.55
1.54
0.49
0.49
0.00 TMS
dioxane
2.00
2.00
2.02
12.29
3.06
3.10
6.11
f1 (ppm)

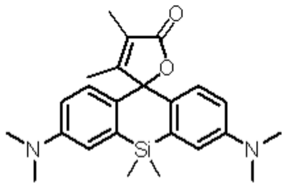

3b

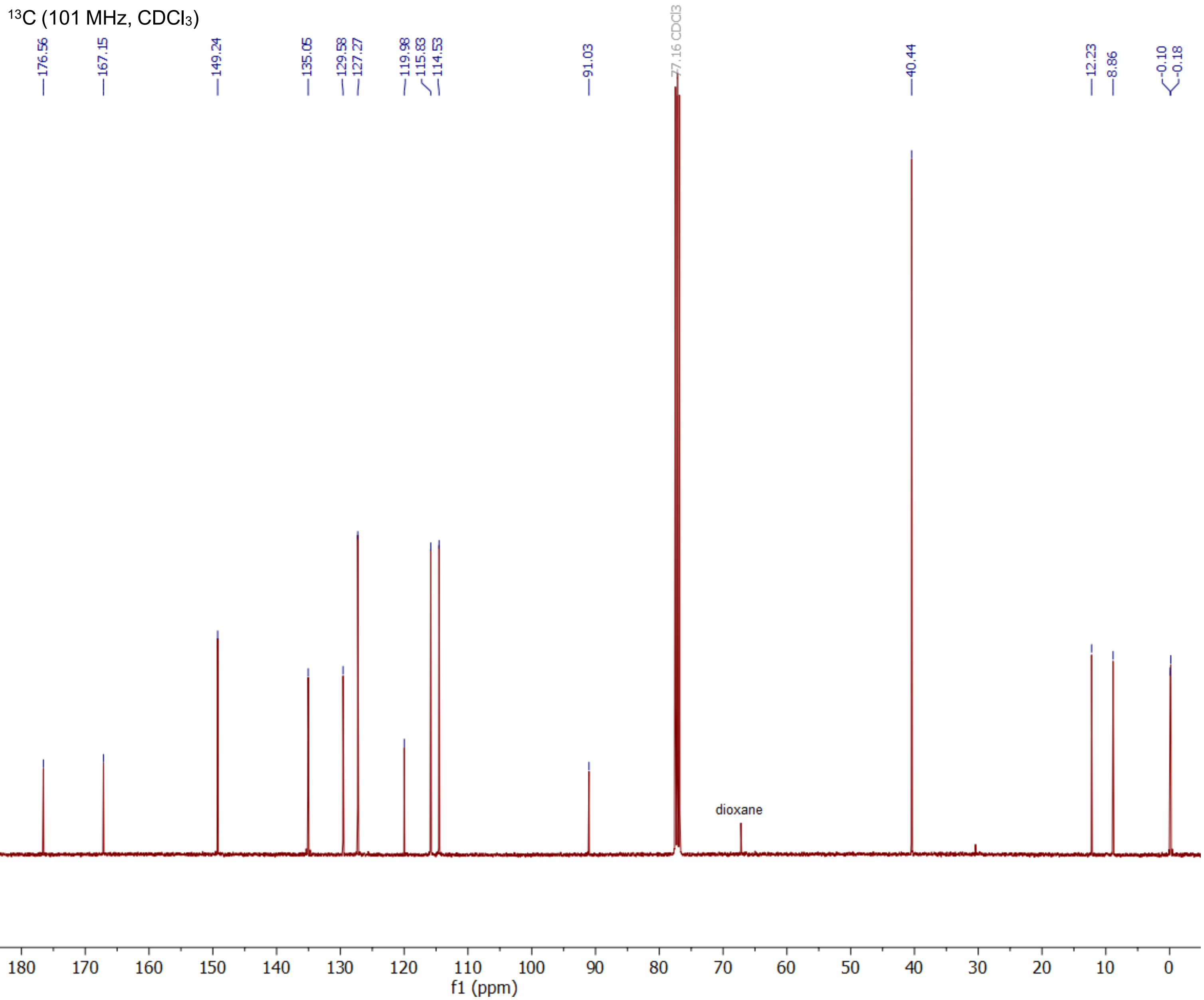

^{13}C (101 MHz, $CDCl_3$)
176.56
167.15
149.24
135.05
129.58
127.27
119.98
115.83
114.53
91.03
77.16 CDCl3
40.44
12.23
8.86
-0.10
-0.18
dioxane
210 200 190 180 170 160 150 140 130 120 110 100 90 80 70 60 50 40 30 20 10 0
f1 (ppm)

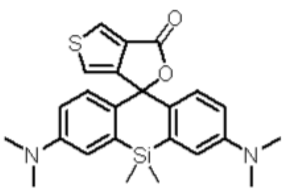

3c

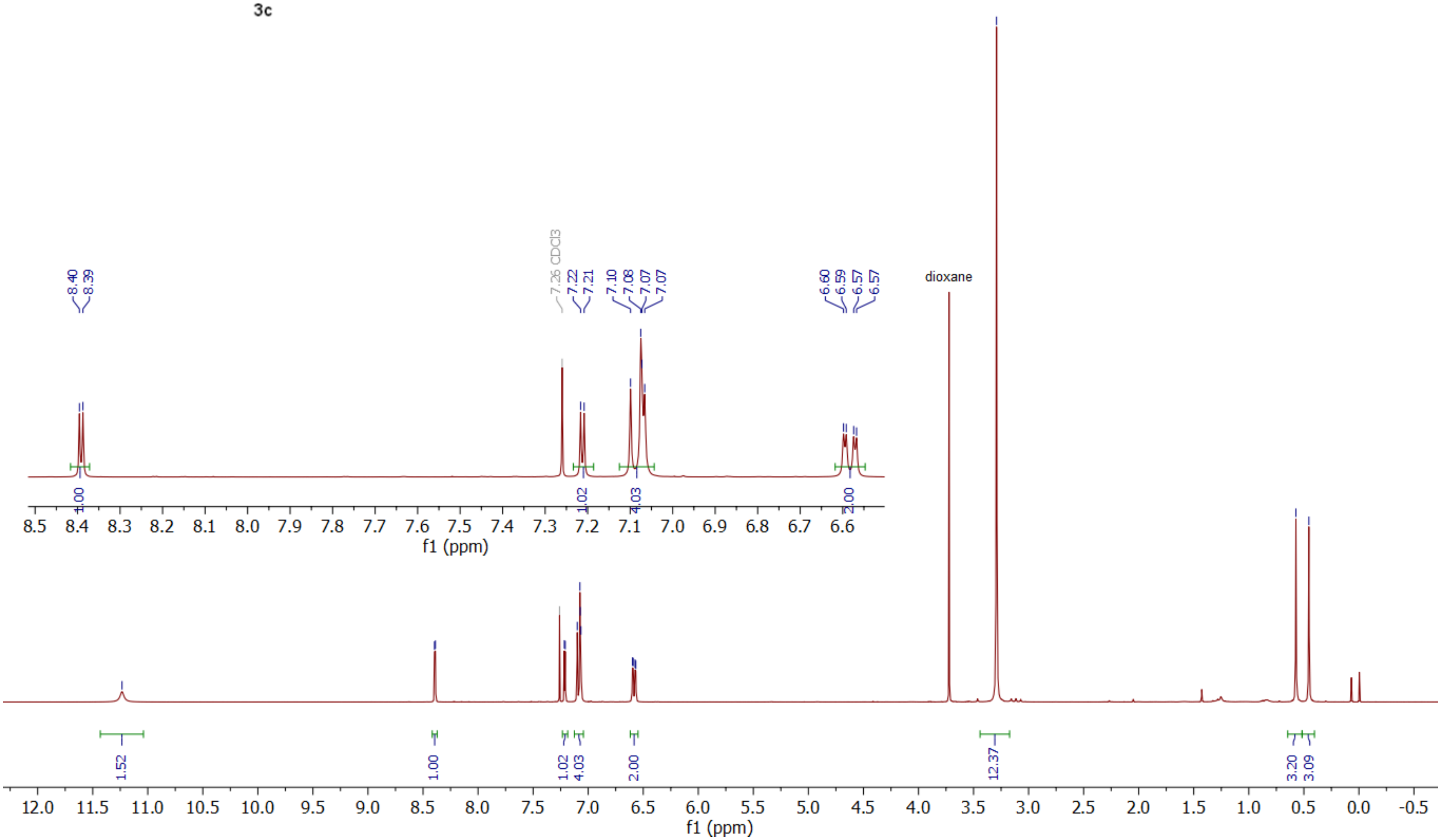

¹H (400 MHz, CDCl3 + 1% TFA-d)
11.23
8.40
8.39
7.26 CDCl3
7.22
7.21
7.10
7.08
7.07
7.07
6.60
6.59
6.57
6.57
3.29
0.57
0.45
dioxane
1.00
1.02
4.03
2.00
1.52
12.37
3.20
3.09
f1 (ppm)

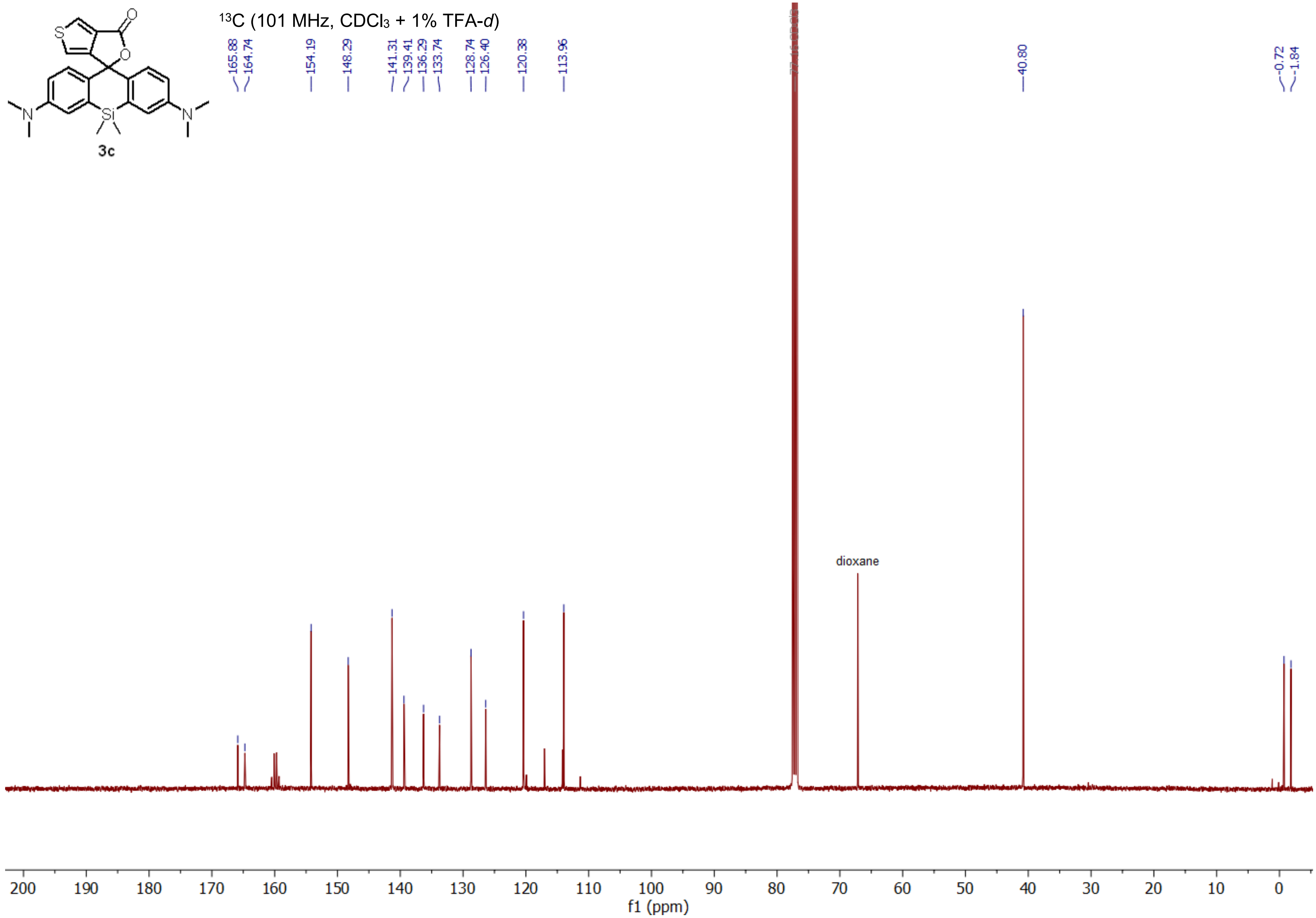
3c
13C (101 MHz, CDCl3 + 1% TFA-d)
165.88
164.74
154.19
148.29
141.31
139.41
136.29
133.74
128.74
126.40
120.38
113.96
40.80
-0.72
-1.84
dioxane
200 190 180 170 160 150 140 130 120 110 100 90 80 70 60 50 40 30 20 10 0
f1 (ppm)

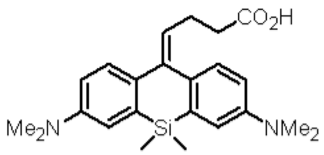

3d

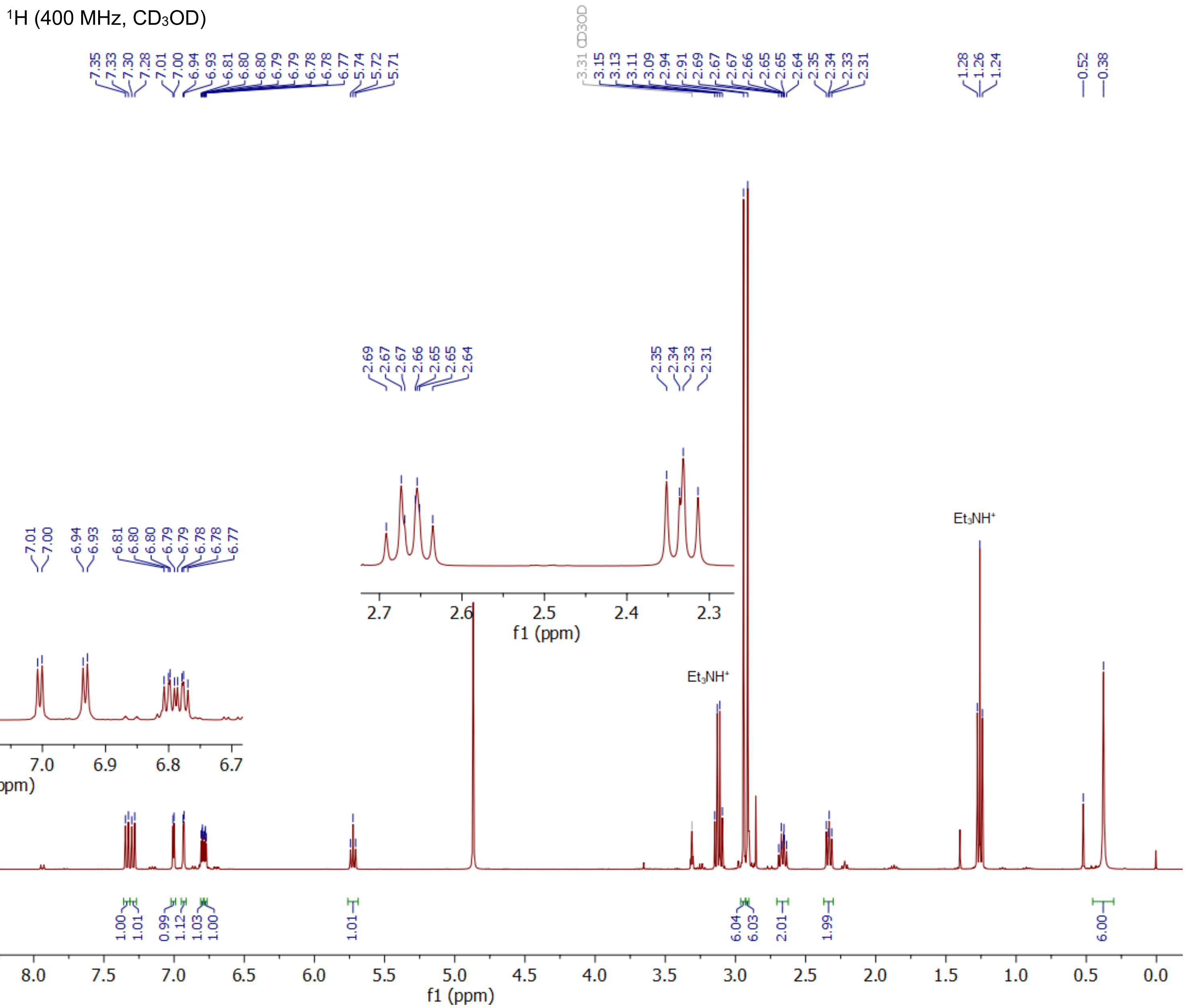

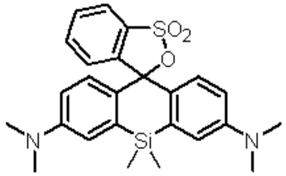

3e

^{1}H (400 MHz, CDCl₃ + 2% TFA-*d*)

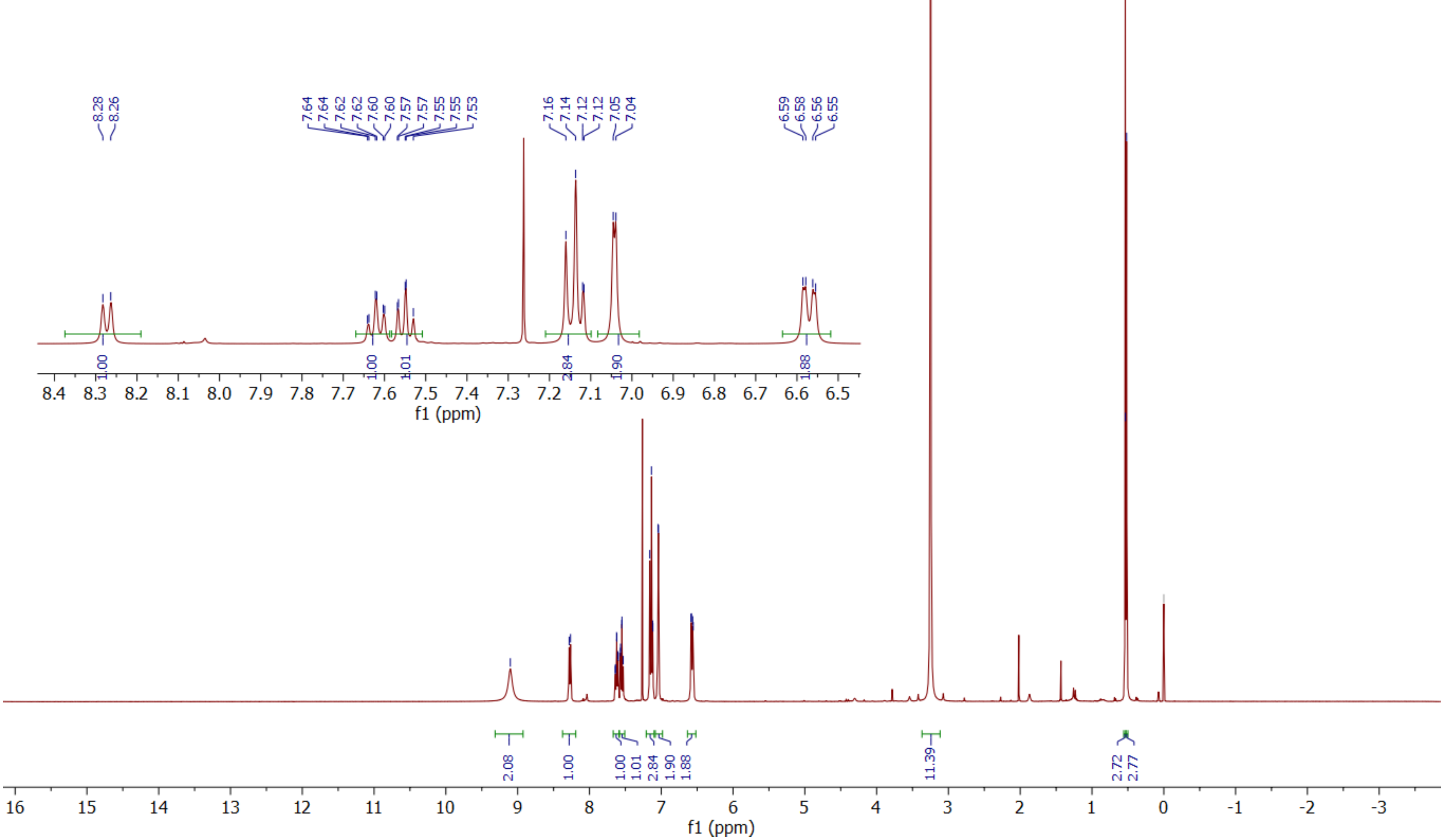

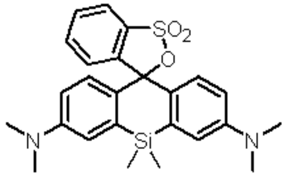

3e

^{13}C (101 MHz, $CDCl_3$ + 2% TFA-*d*)

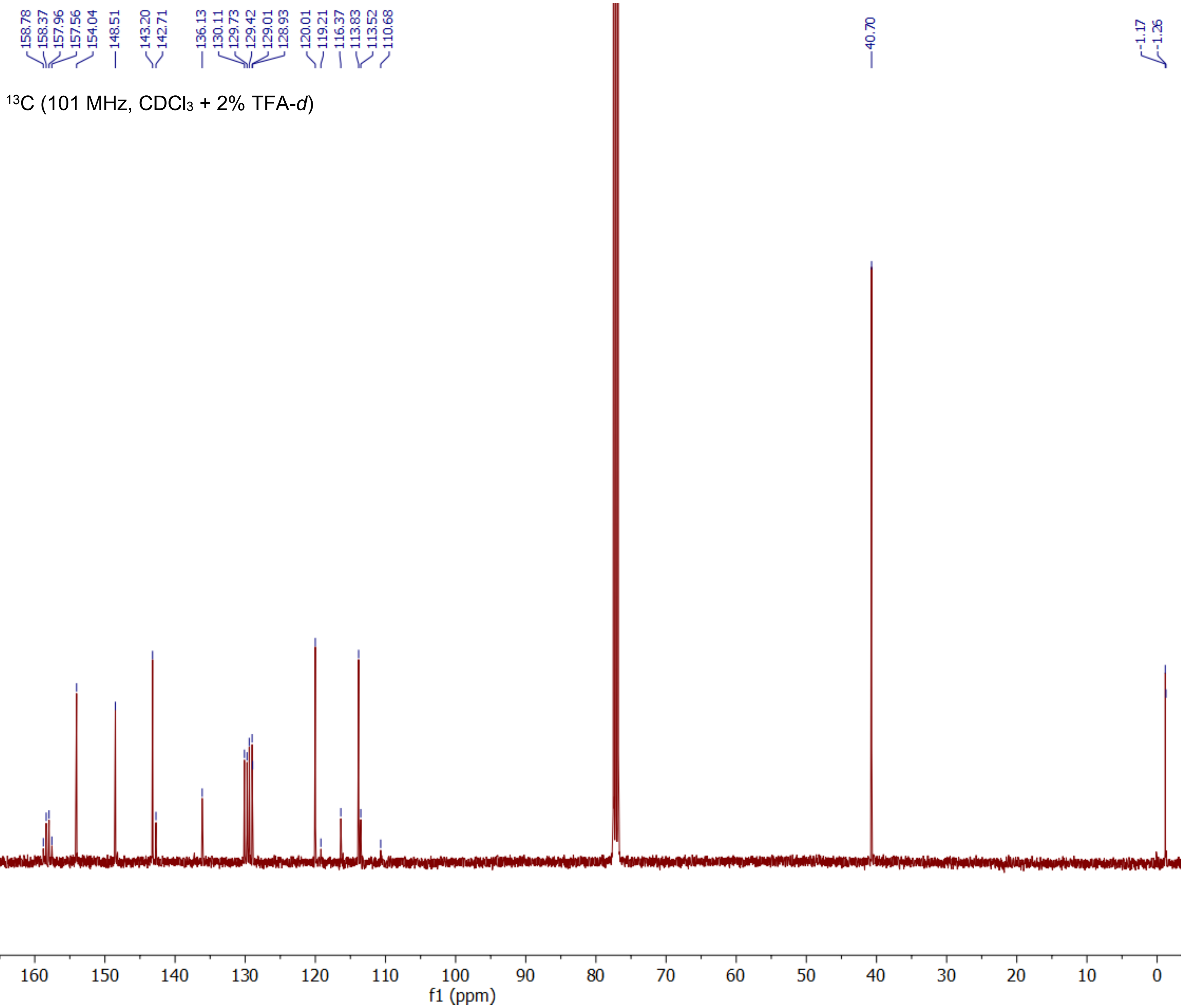

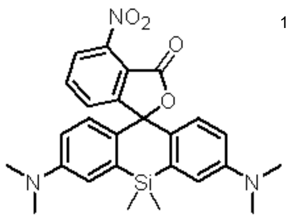

3f

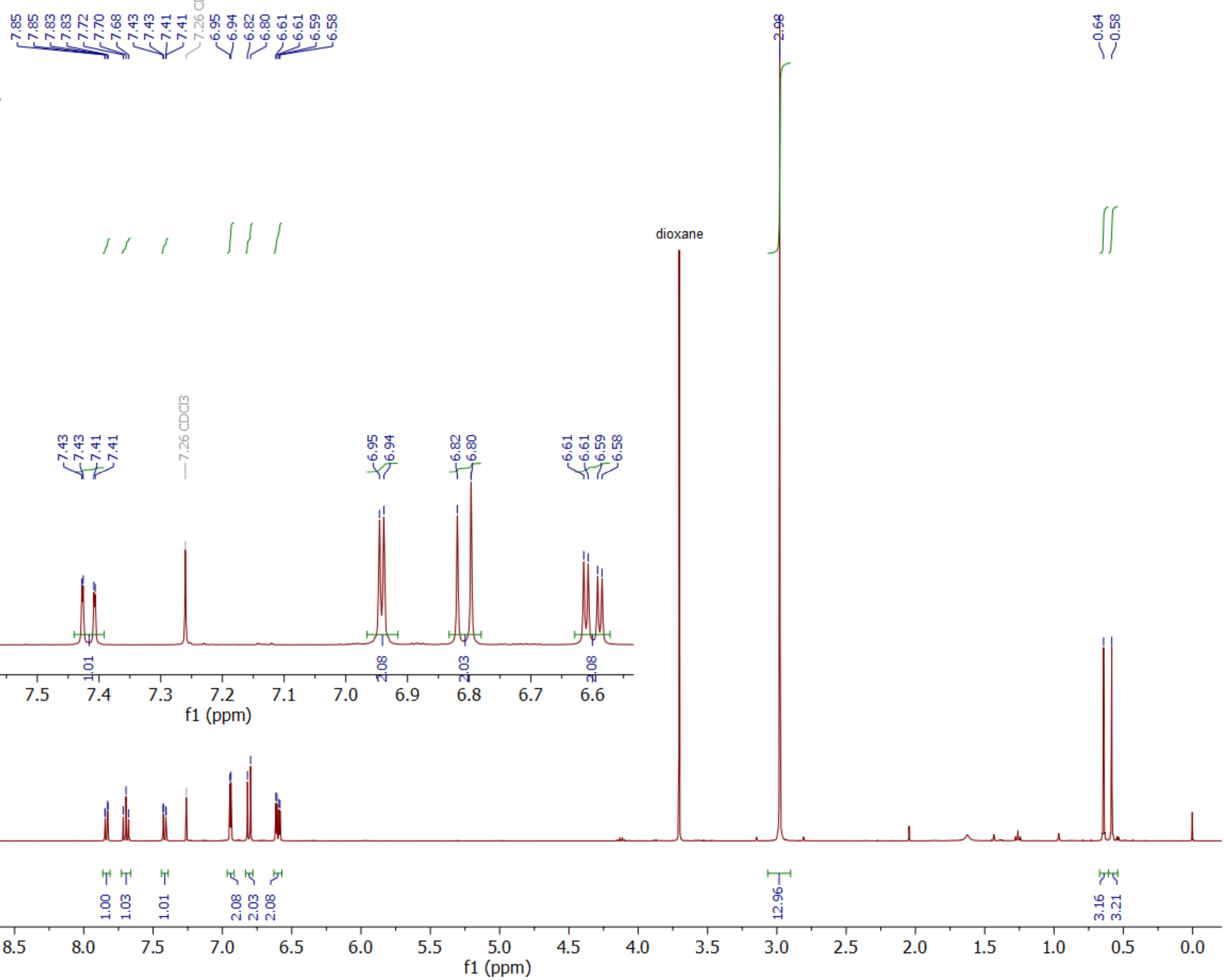

^1H (400 MHz, $CDCl_3$)
7.85 7.85 7.83 7.83 7.72 7.70 7.68 7.43 7.43 7.41 7.41 7.26 CDCl3 6.95 6.94 6.82 6.80 6.61 6.61 6.59 6.58
2.98
0.64 0.58
dioxane
1.00 1.03 1.01 2.08 2.03 2.08 12.96 3.16 3.21
f1 (ppm)

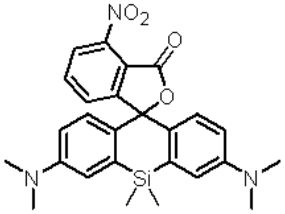

3f

^{13}C (101 MHz, $CDCl_3$)

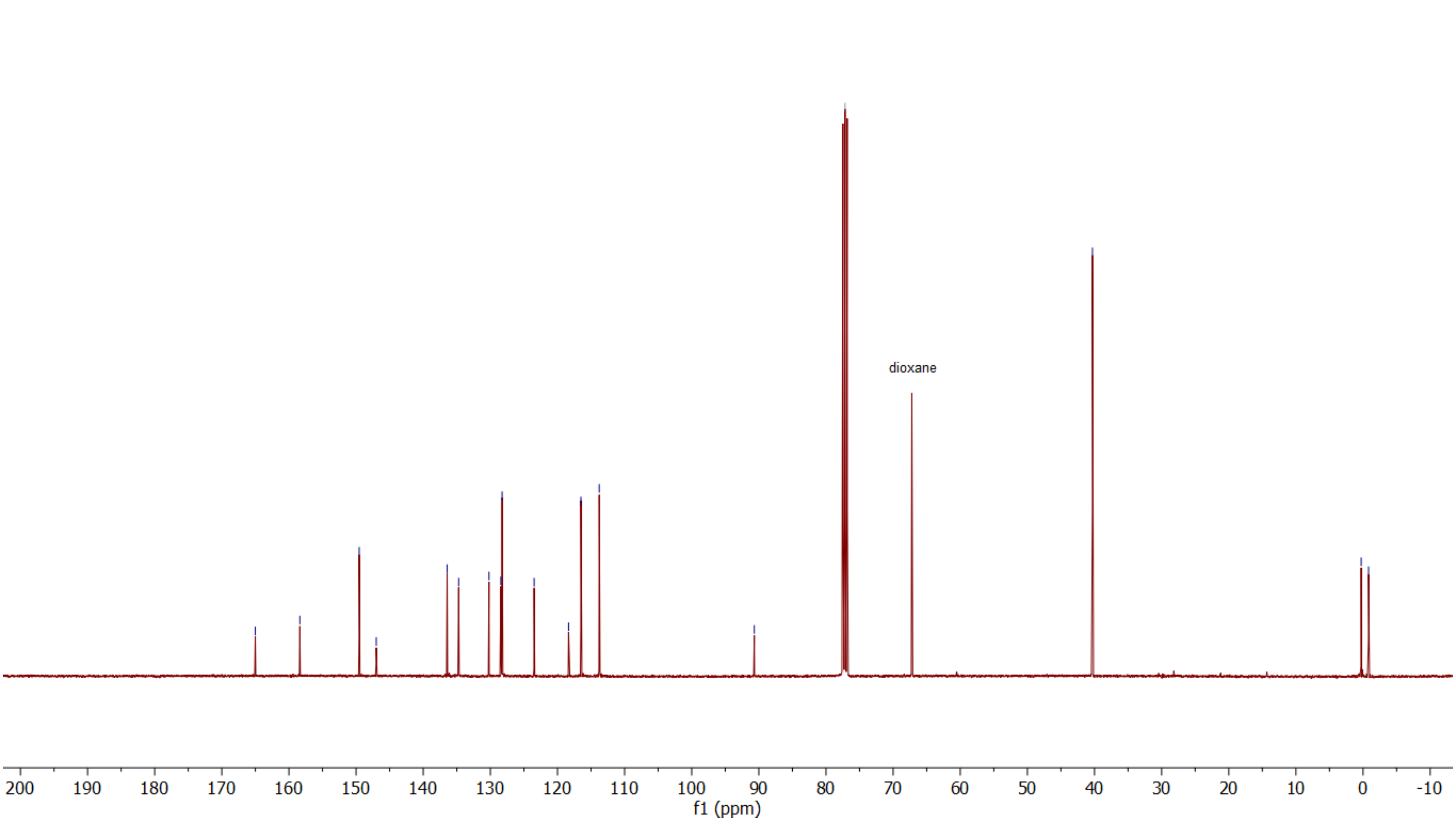

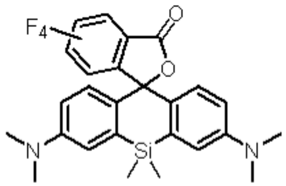

3g

^{1}H (400 MHz, $CDCl_3$)

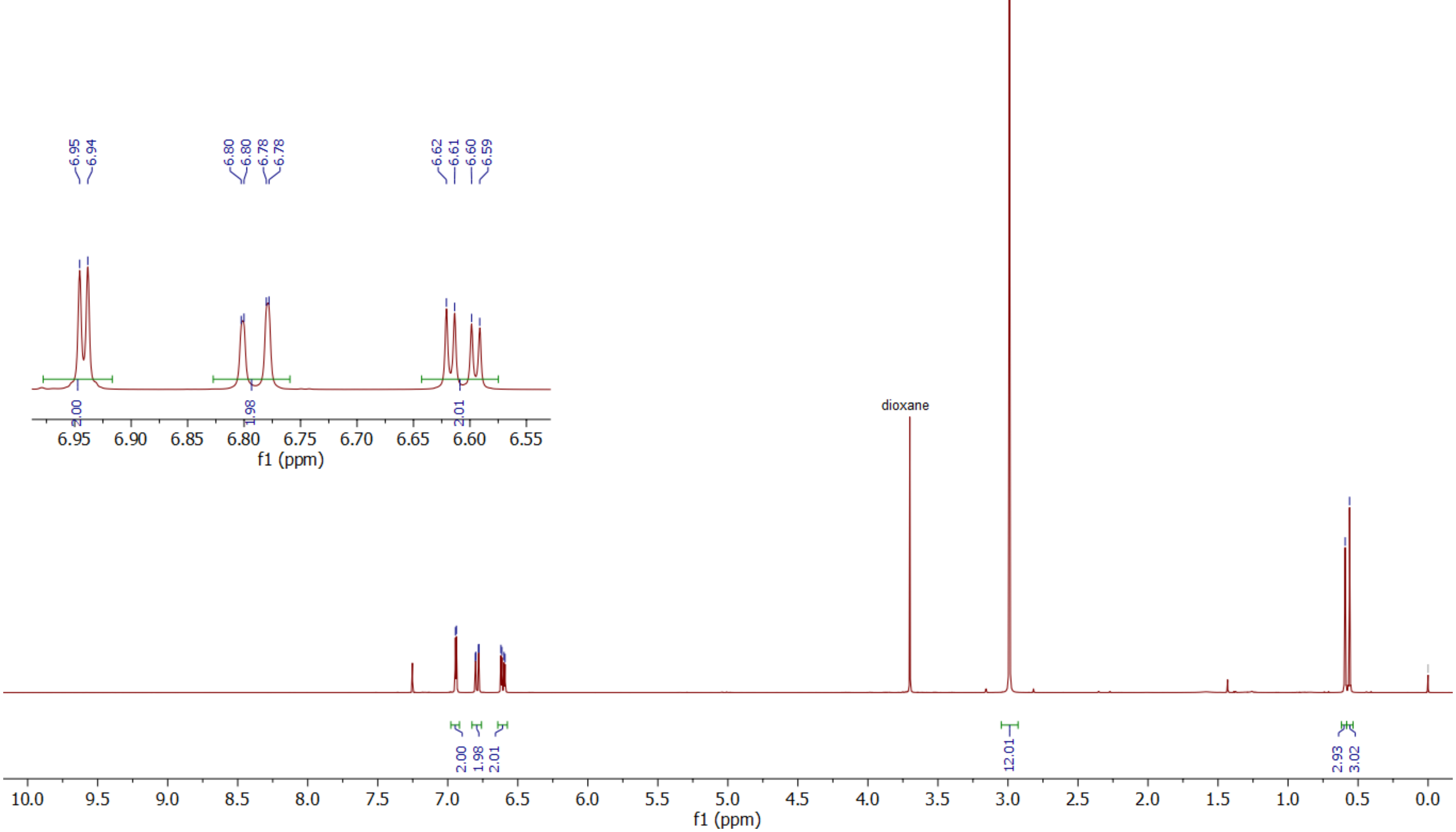

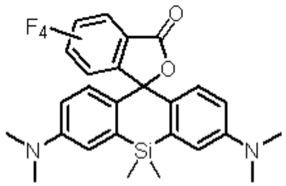
F4
O
O
N
Si
N

3g

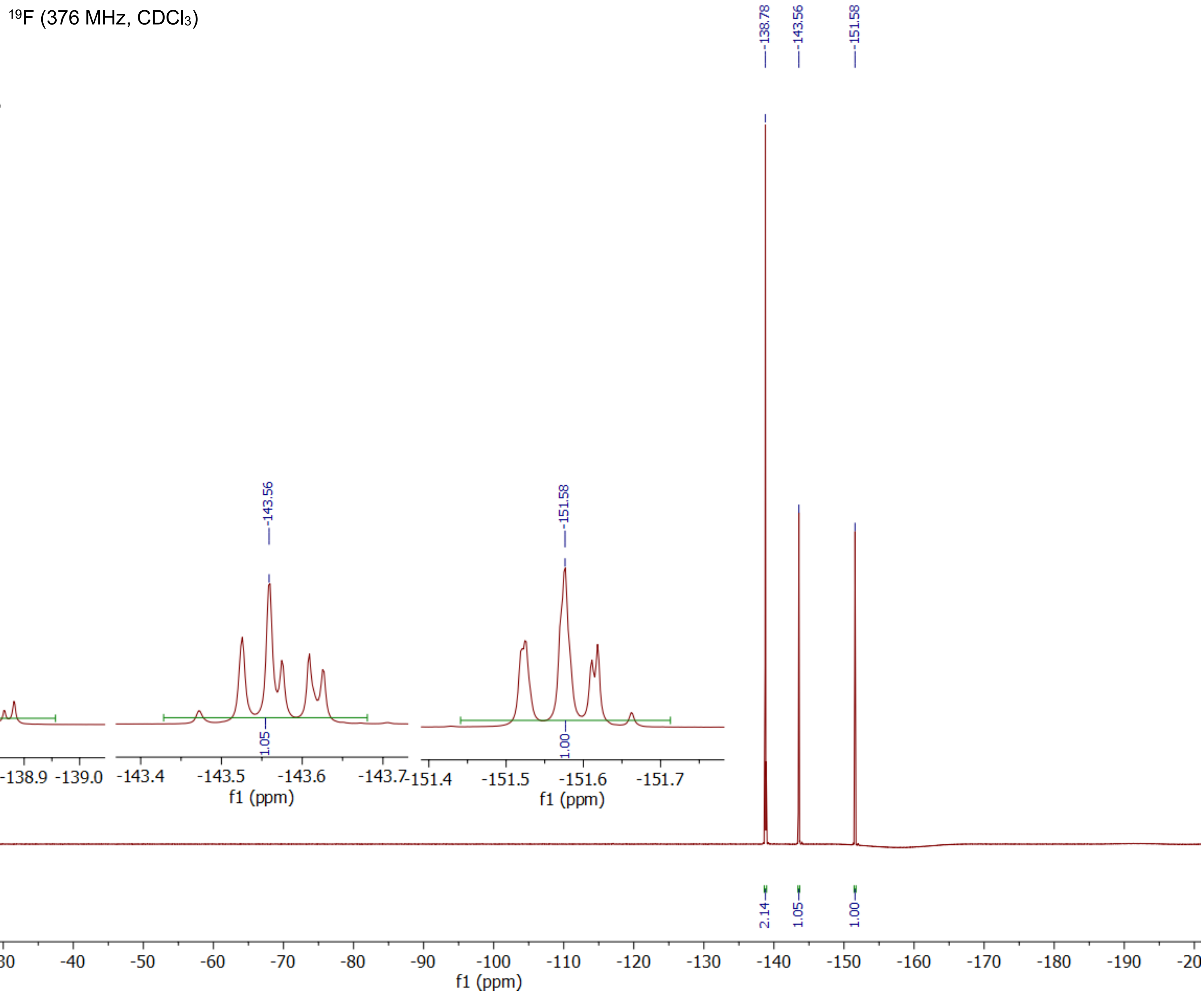
19F (376 MHz, CDCl3)
-138.78
-143.56
-151.58
2.14
1.05
1.00
f1 (ppm)

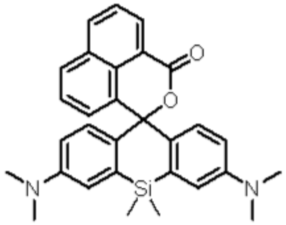

3h

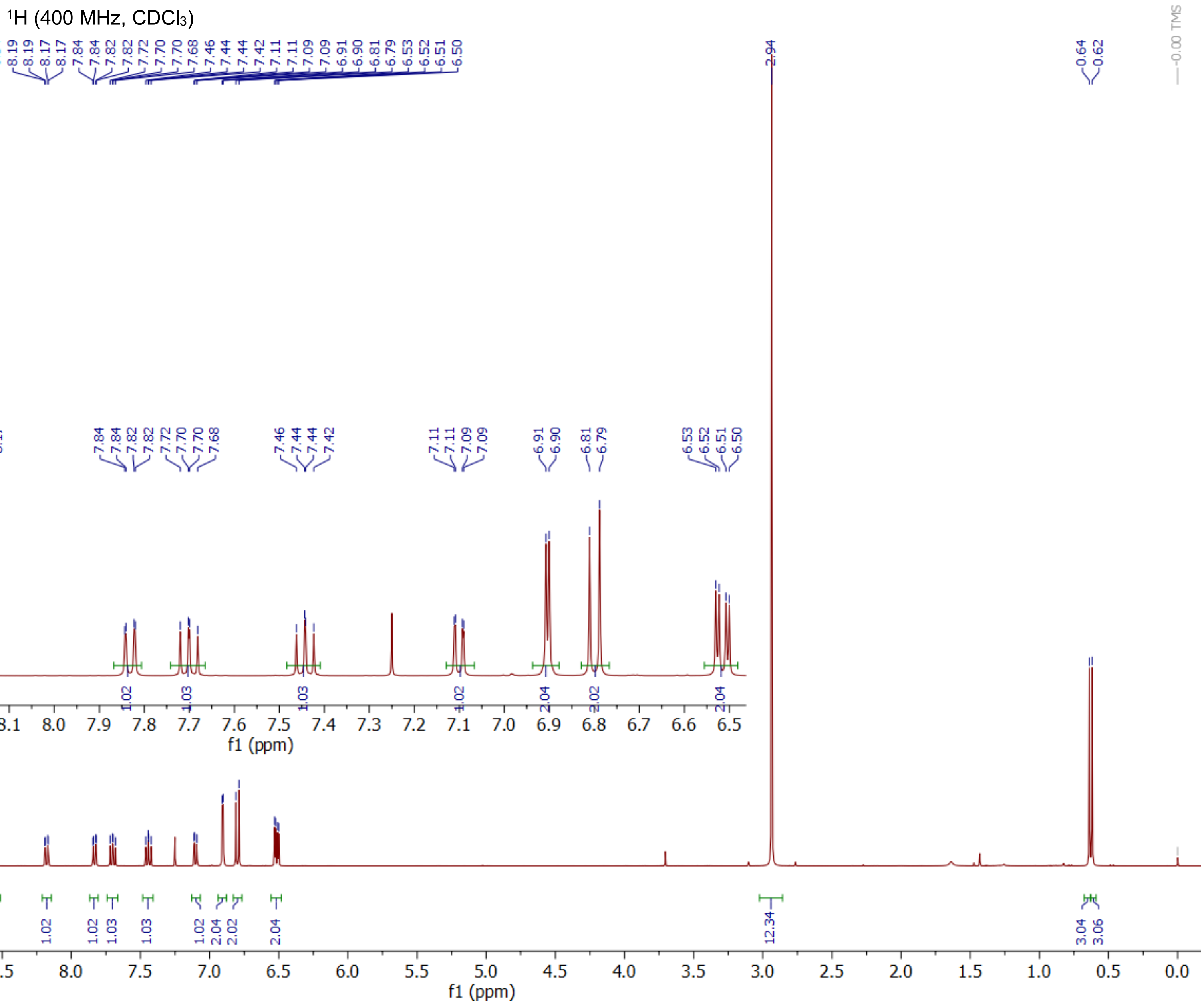

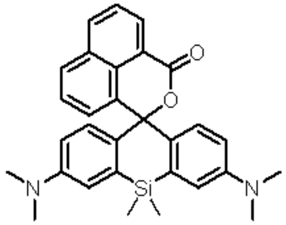

3h

^{13}C (101 MHz, $CDCl_3$)

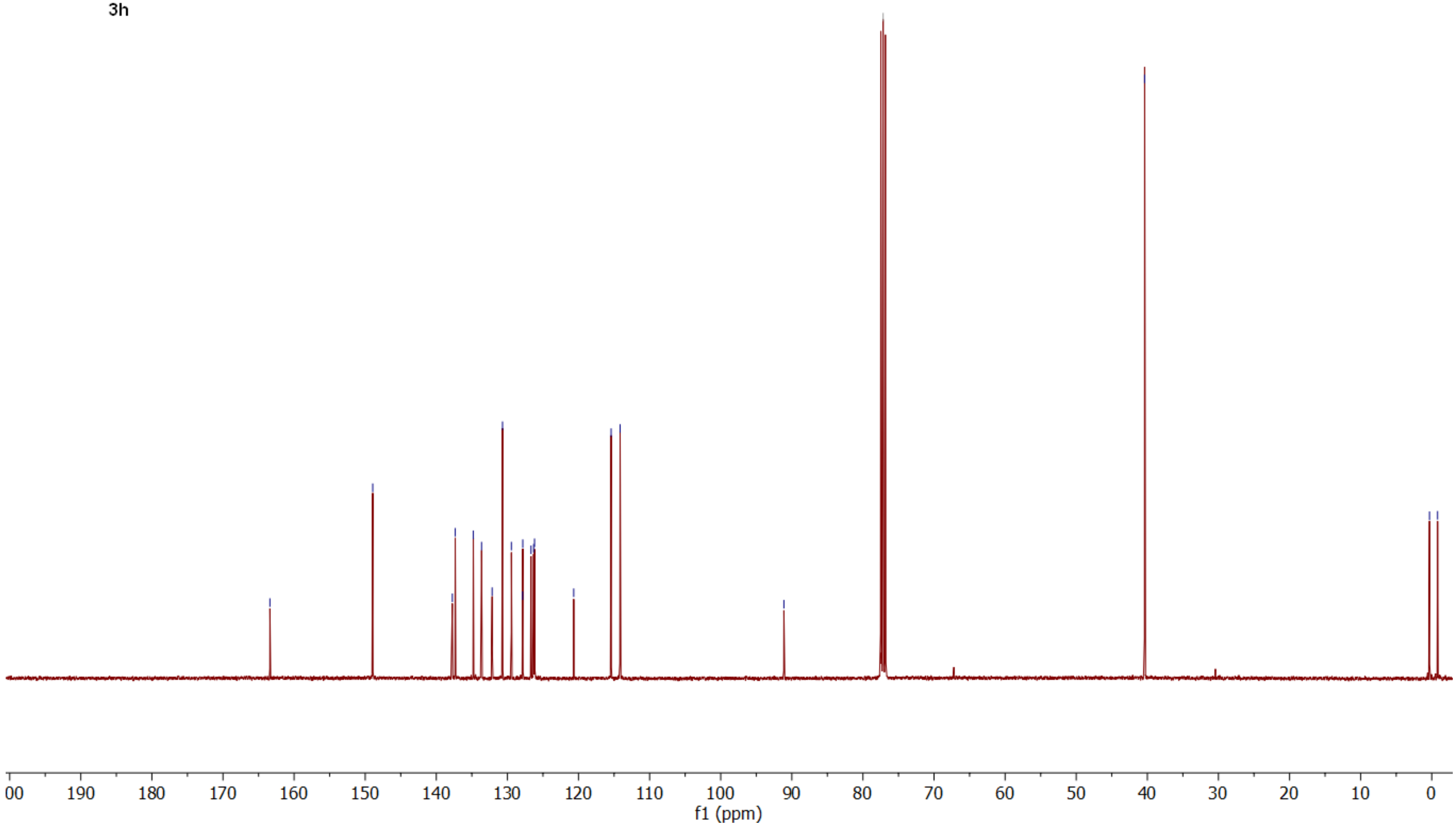

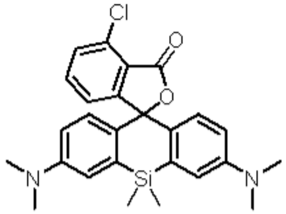

3i

^{1}H (400 MHz, $CDCl_3$)

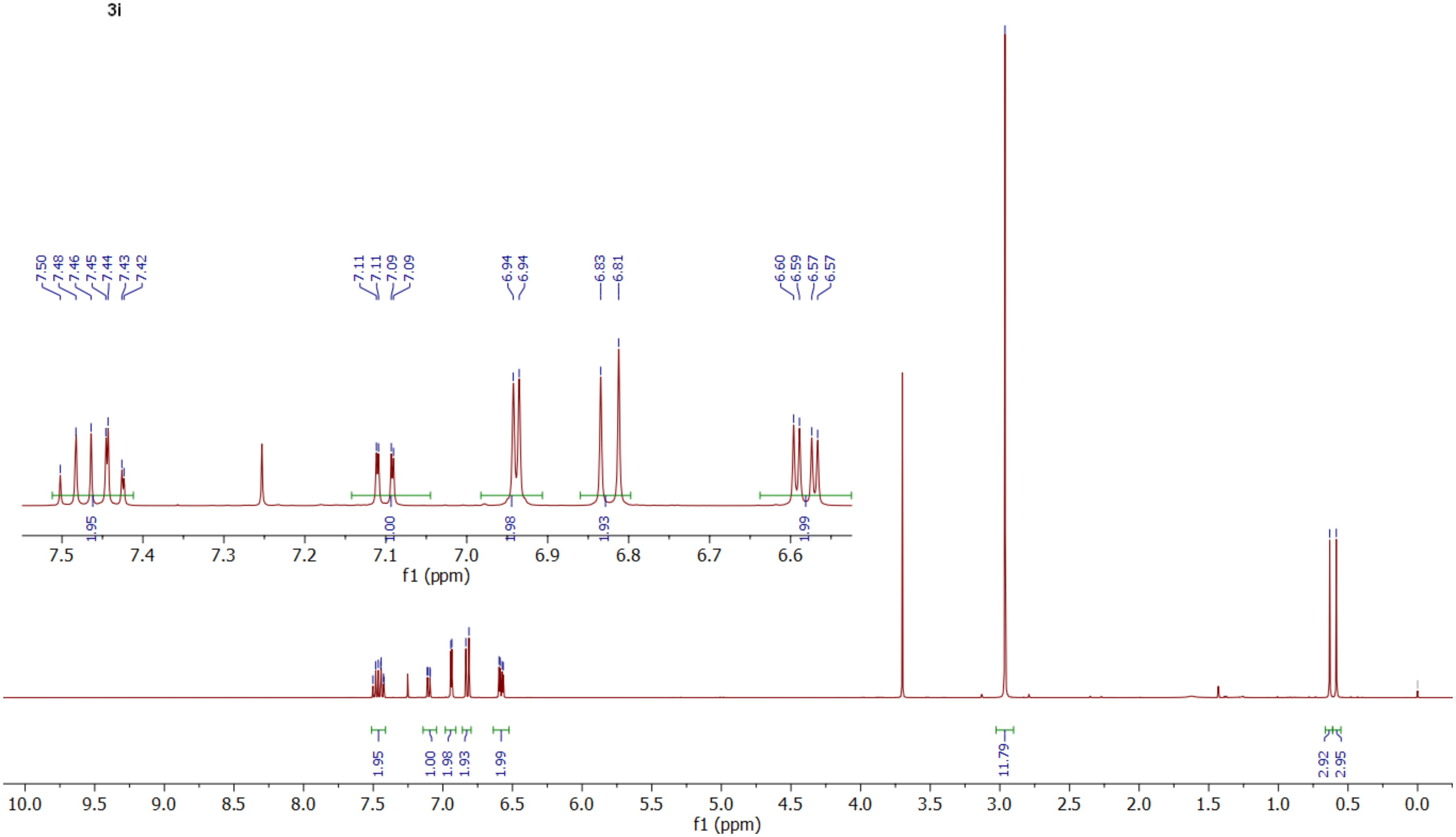

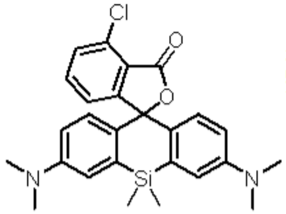

3i

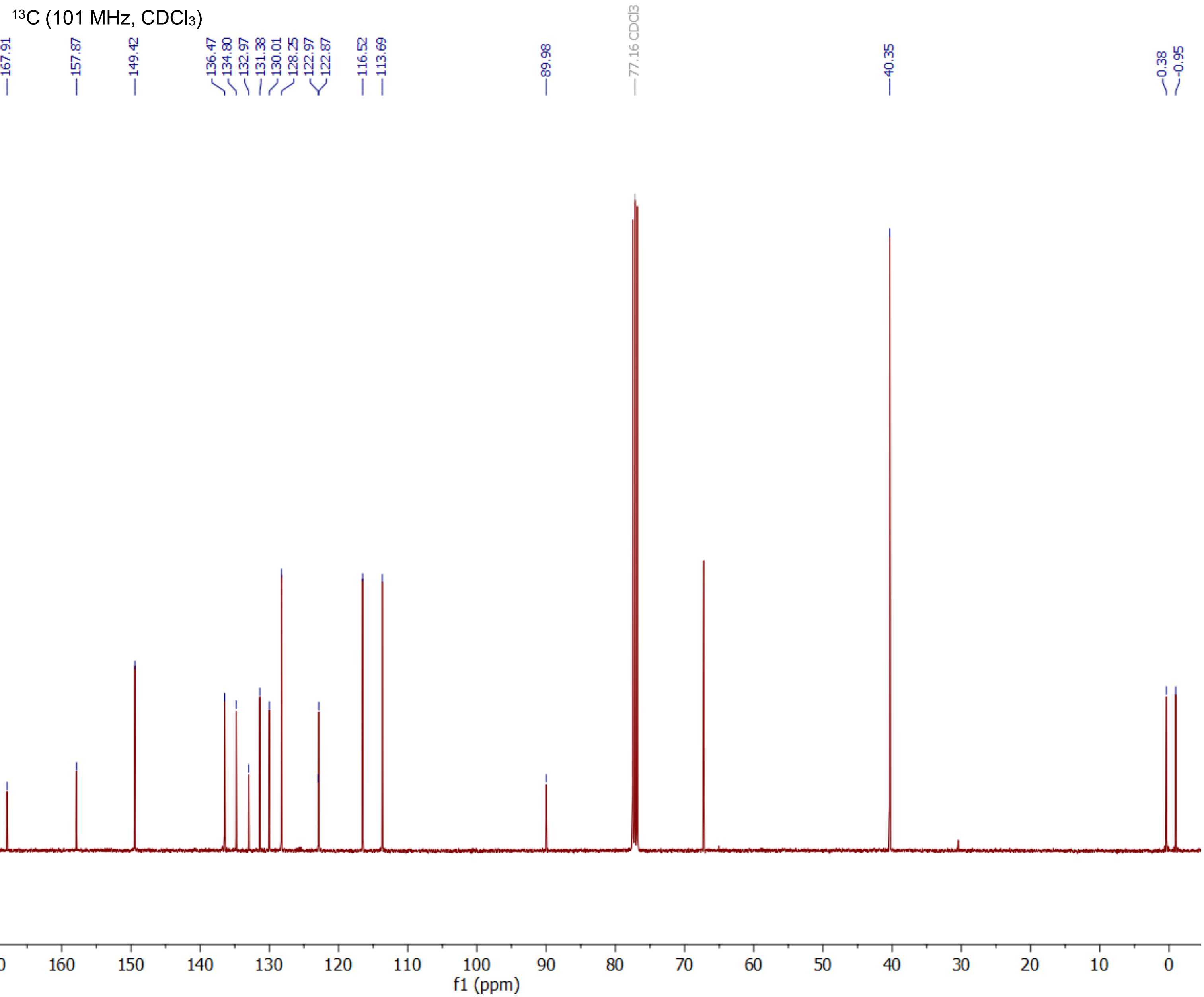

13C (101 MHz, CDCl3)
167.91
157.87
149.42
136.47
134.80
132.97
131.38
130.01
128.25
122.97
122.87
116.52
113.69
89.98
77.16 CDCl3
40.35
0.38
-0.95
f1 (ppm)

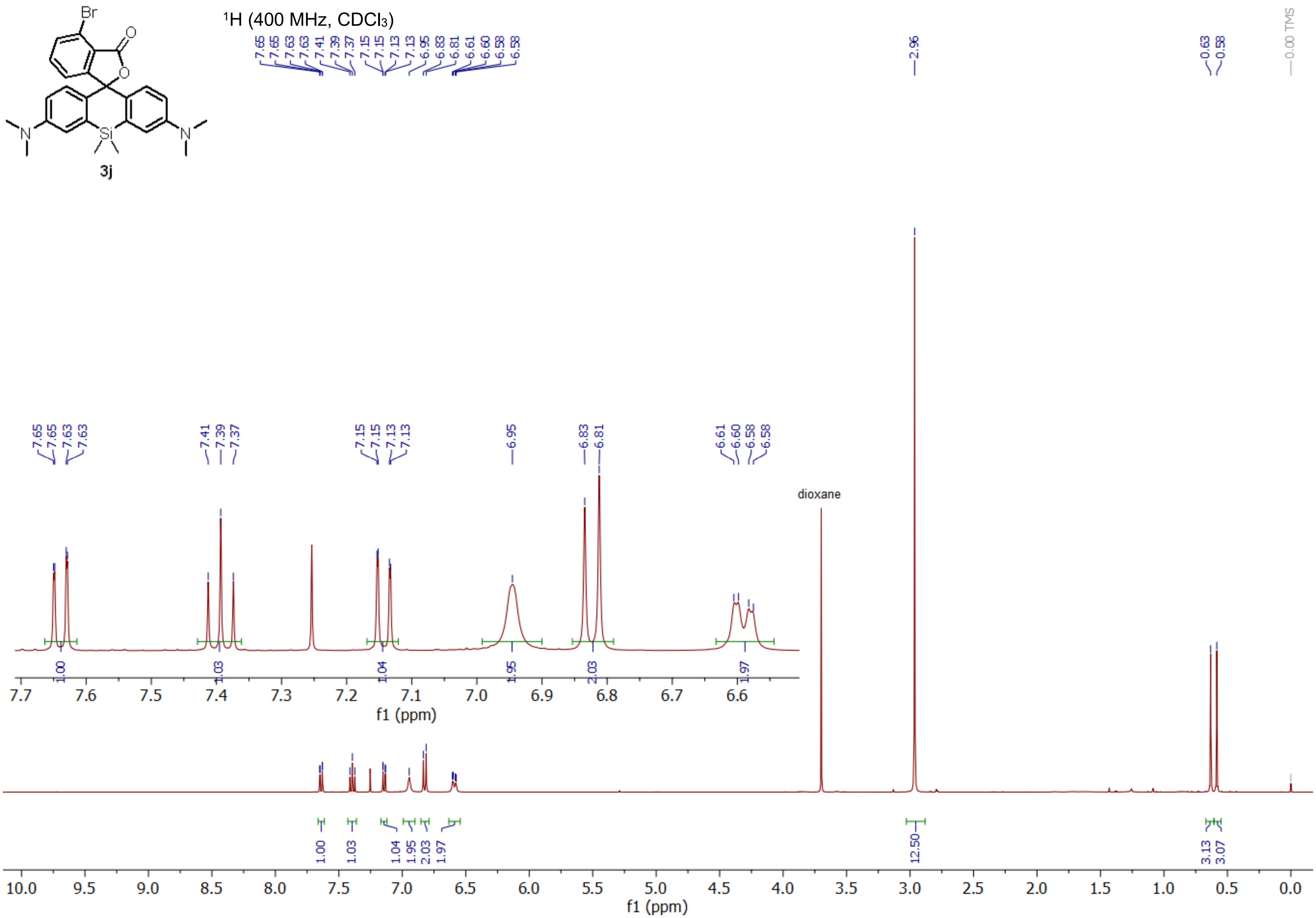

Br
O
O
N
Si
N
3j
¹H (400 MHz, CDCl3)
7.65 7.65 7.63 7.63 7.41 7.39 7.37 7.15 7.15 7.13 7.13 6.95 6.83 6.81 6.61 6.60 6.58 6.58
2.96
0.63 0.58
0.00 TMS
dioxane
1.00 1.03 1.04 1.95 2.03 1.97
f1 (ppm)
1.00 1.03 1.04 1.95 2.03 1.97 12.50 3.13 3.07
10.0 9.5 9.0 8.5 8.0 7.5 7.0 6.5 6.0 5.5 5.0 4.5 4.0 3.5 3.0 2.5 2.0 1.5 1.0 0.5 0.0
f1 (ppm)

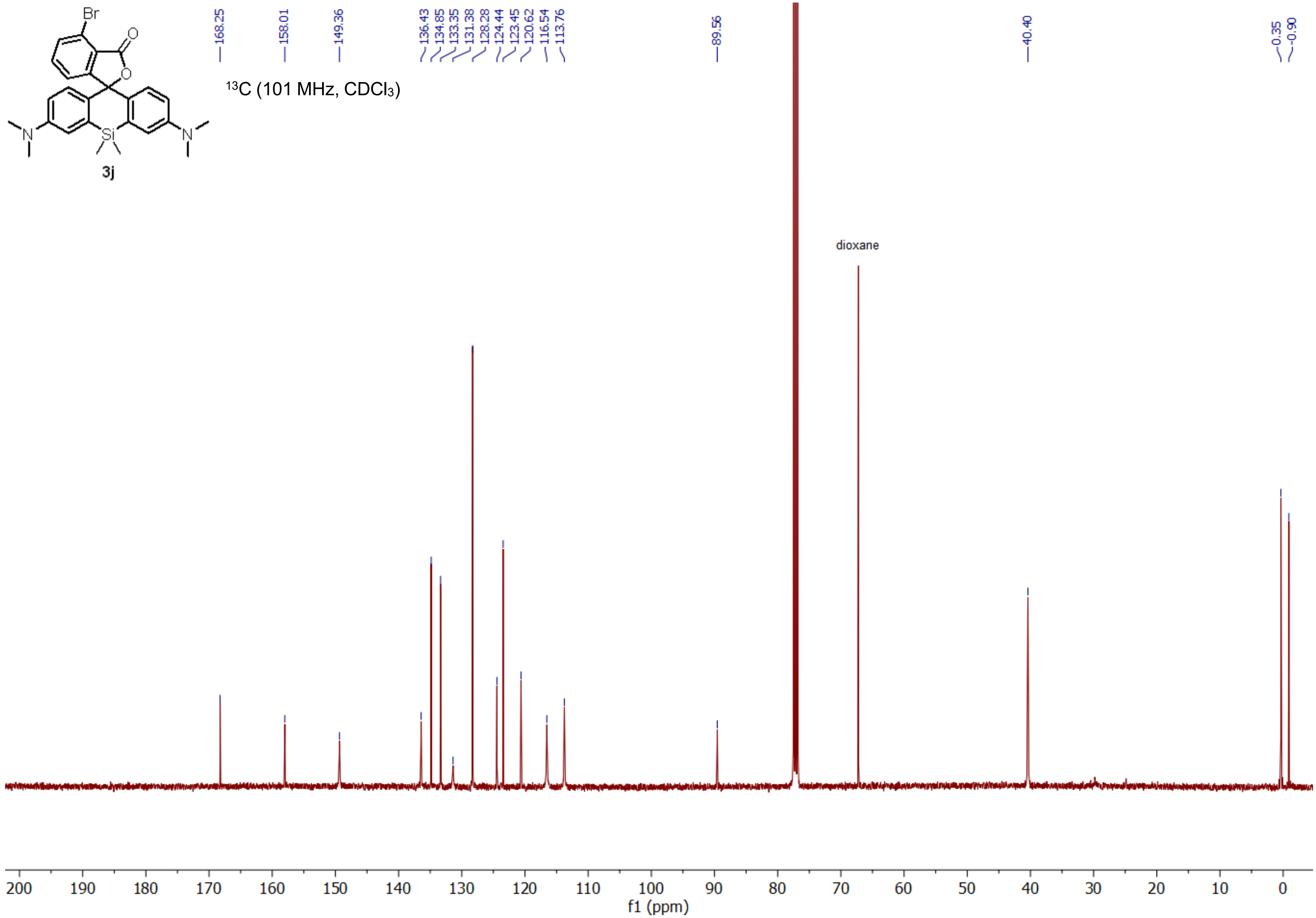

Br
O
O
N
Si
N
3j
13C (101 MHz, CDCl3)
168.25
158.01
149.36
136.43
134.85
133.35
131.38
128.28
124.44
123.45
120.62
116.54
113.76
89.56
40.40
0.35
-0.90
dioxane
200
190
180
170
160
150
140
130
120
110
100
90
80
70
60
50
40
30
20
10
0
f1 (ppm)

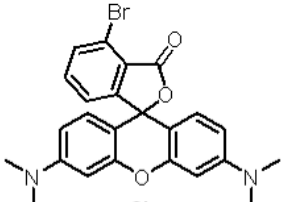

3k

^{1}H (400 MHz, DMSO-d_6 + 1% TFA-d)

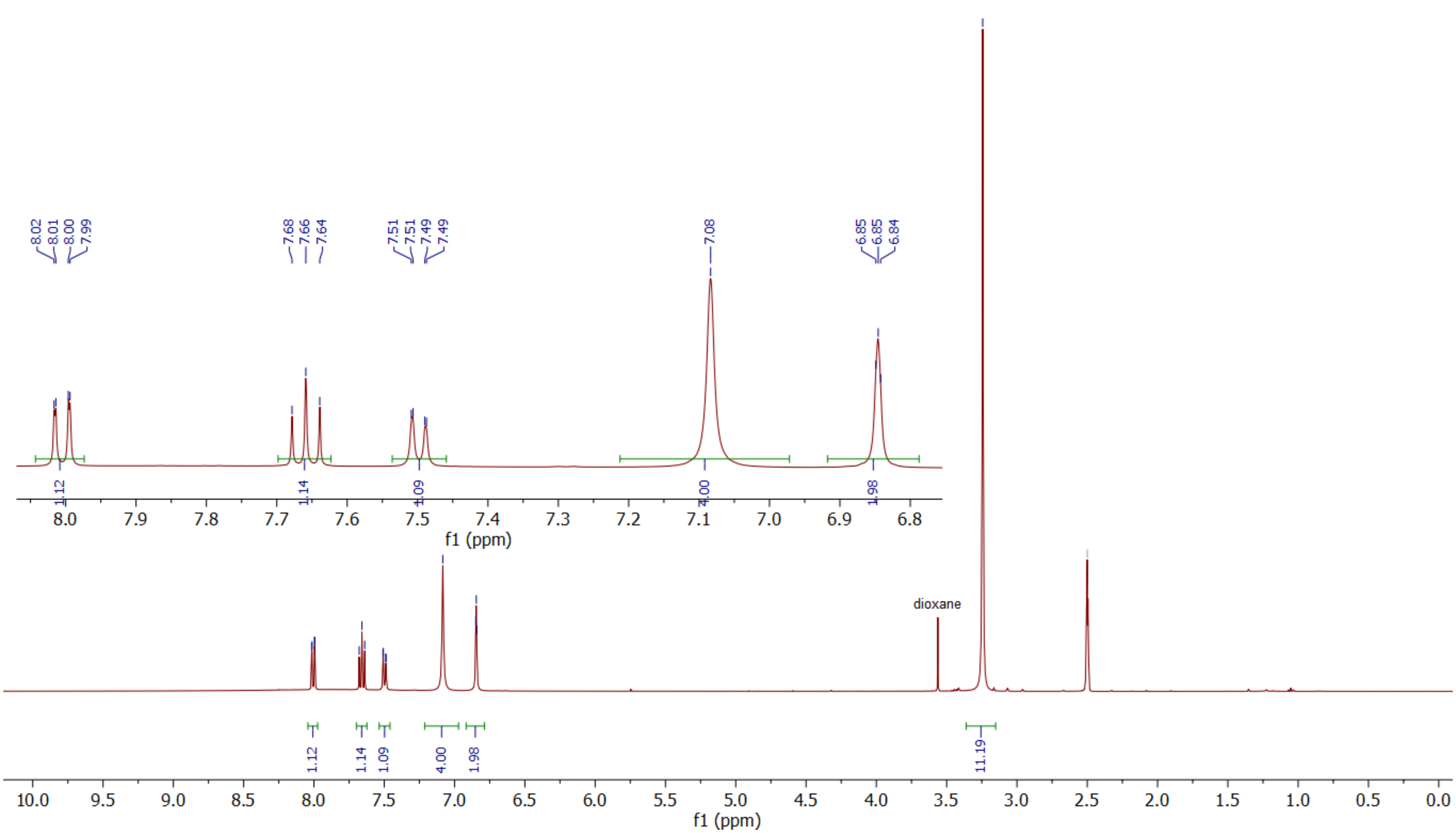

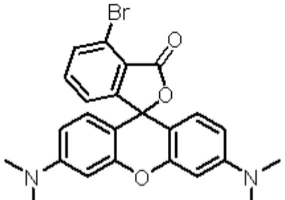

3k

^{13}C (101 MHz, DMSO-d_6 + 1% TFA-d)

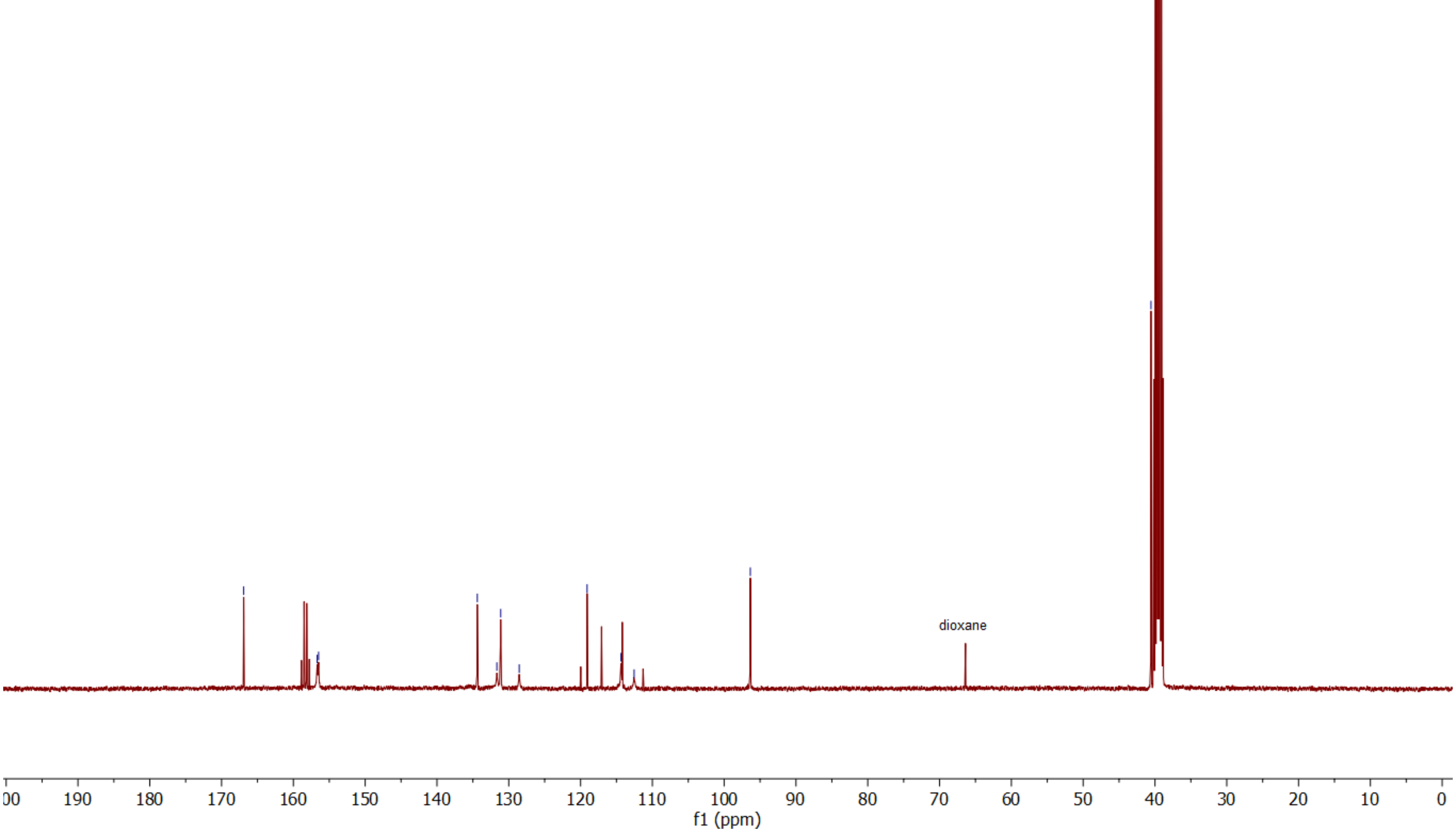

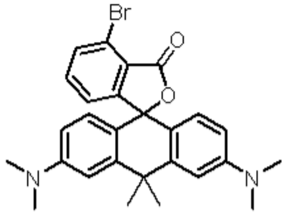
Br
O
O
N
N

3l

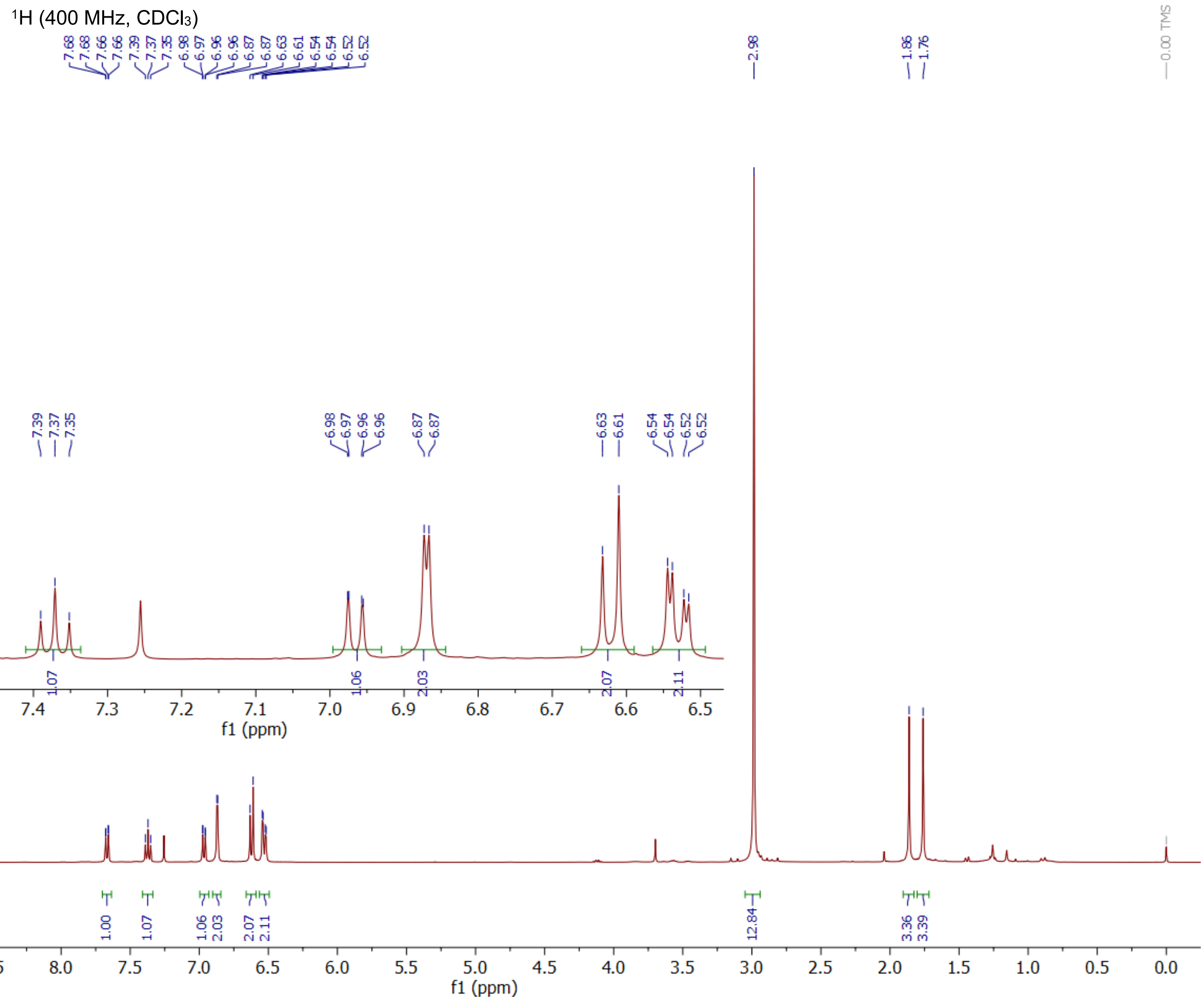
1H (400 MHz, CDCl3)
7.68 7.68 7.66 7.66 7.39 7.37 7.35 6.98 6.97 6.96 6.96 6.87 6.87 6.63 6.61 6.54 6.54 6.52 6.52
2.98
1.86 1.76
0.00 TMS
1.00 1.07 1.06 2.03 2.07 2.11
12.84 3.36 3.39
f1 (ppm)

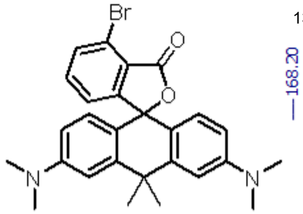

3l

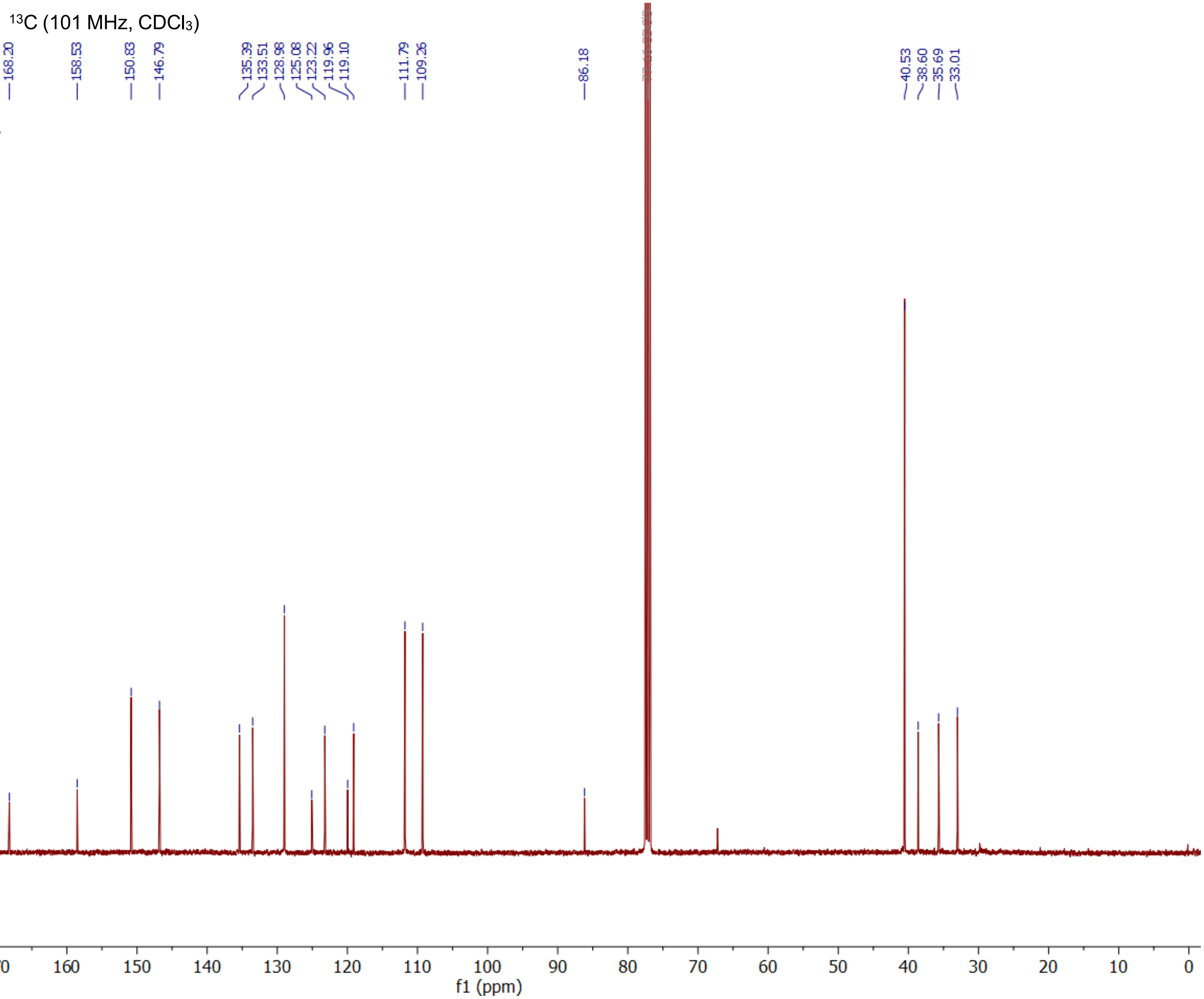

^{13}C (101 MHz, $CDCl_3$)
168.20
158.53
150.83
146.79
135.39
133.51
128.98
125.08
123.22
119.96
119.10
111.79
109.26
86.18
40.53
38.60
35.69
33.01
f1 (ppm)

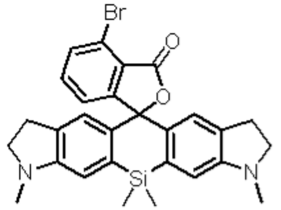

3m

^{1}H (400 MHz, $CDCl_3$)

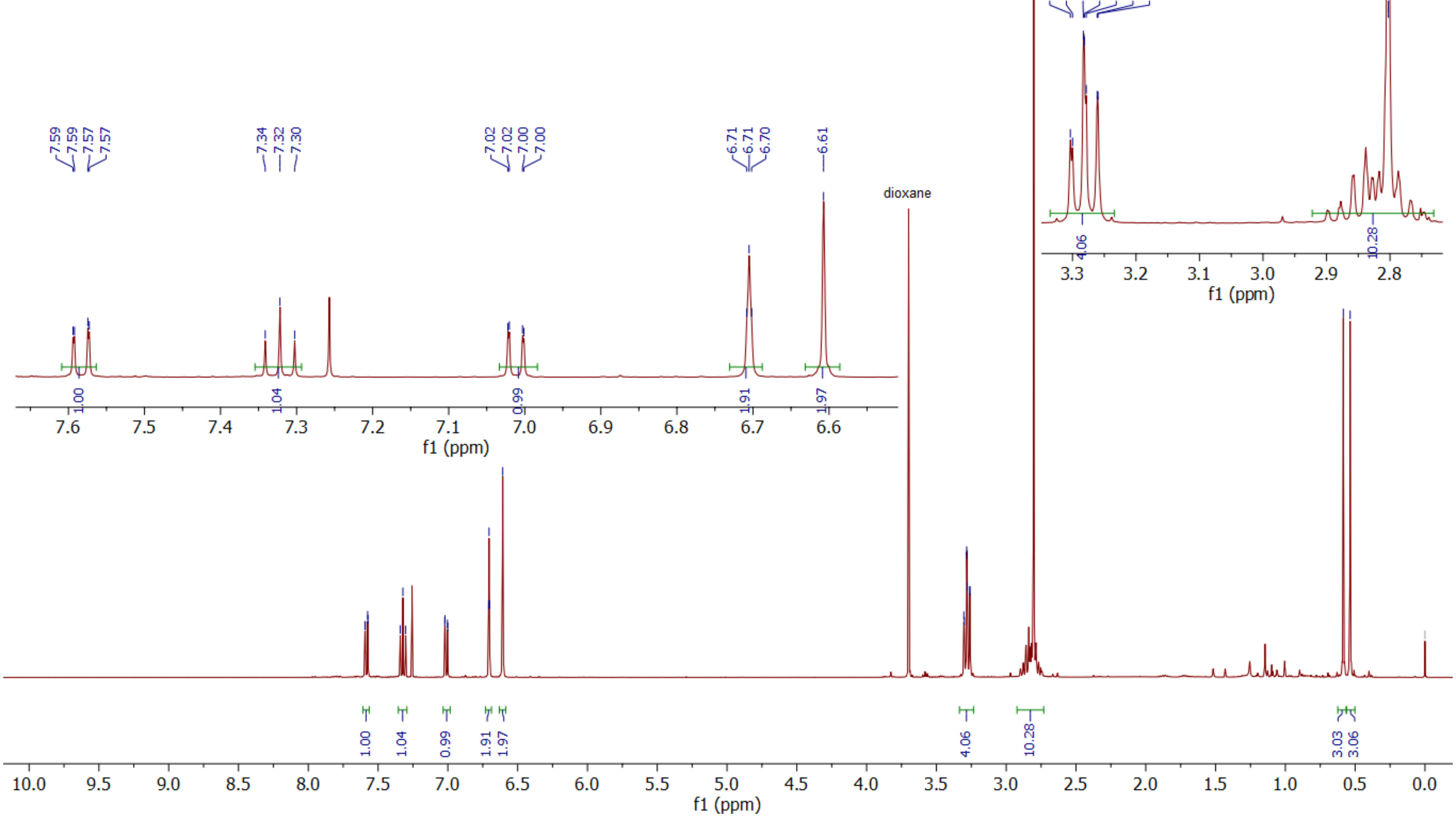

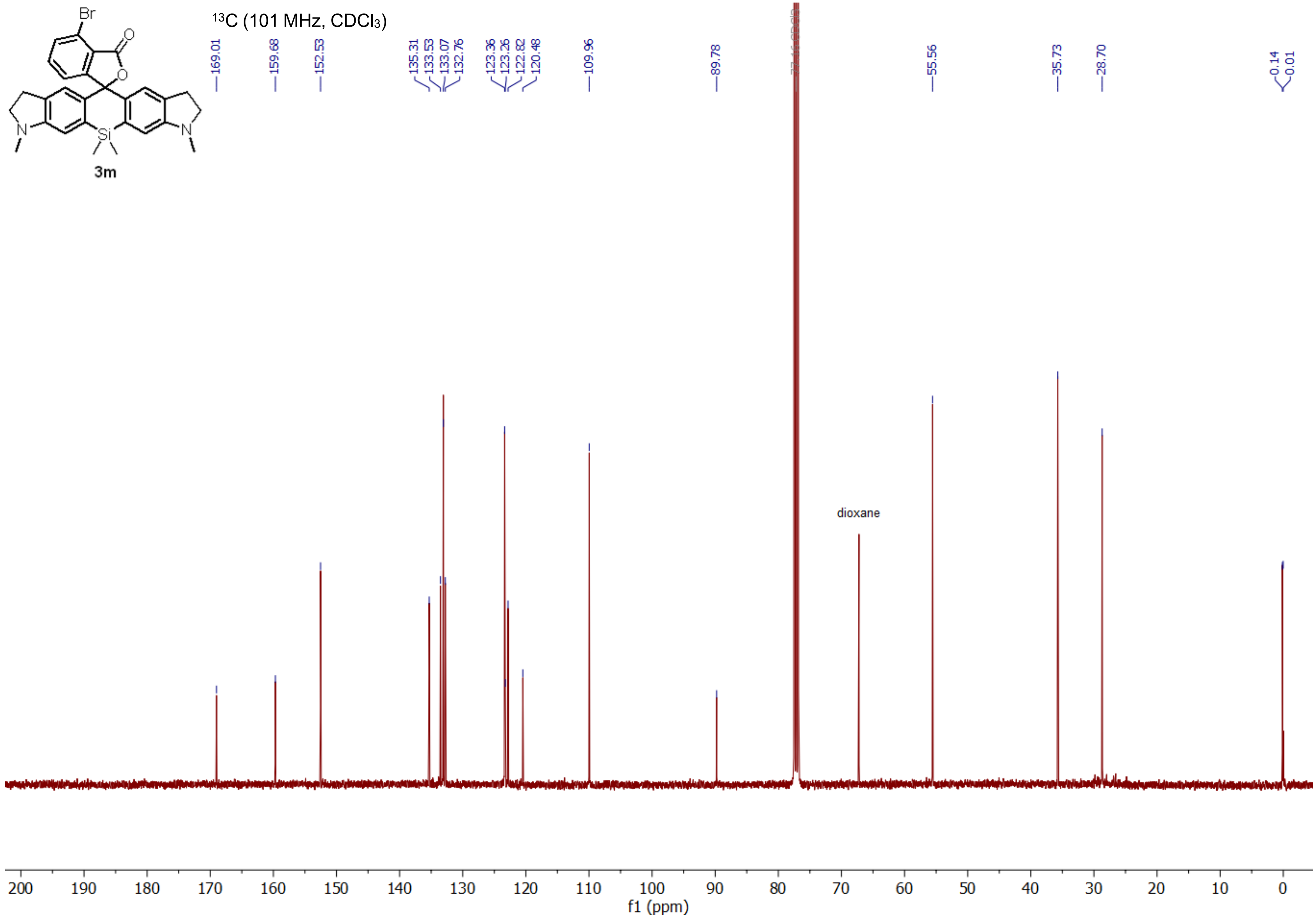
Br
O
O
N
Si
N
3m
13C (101 MHz, CDCl3)
169.01
159.68
152.53
135.31
133.53
133.07
132.76
123.36
123.26
122.82
120.48
109.96
89.78
[illegible]
55.56
35.73
28.70
0.14
0.01
dioxane
200 190 180 170 160 150 140 130 120 110 100 90 80 70 60 50 40 30 20 10 0
f1 (ppm)

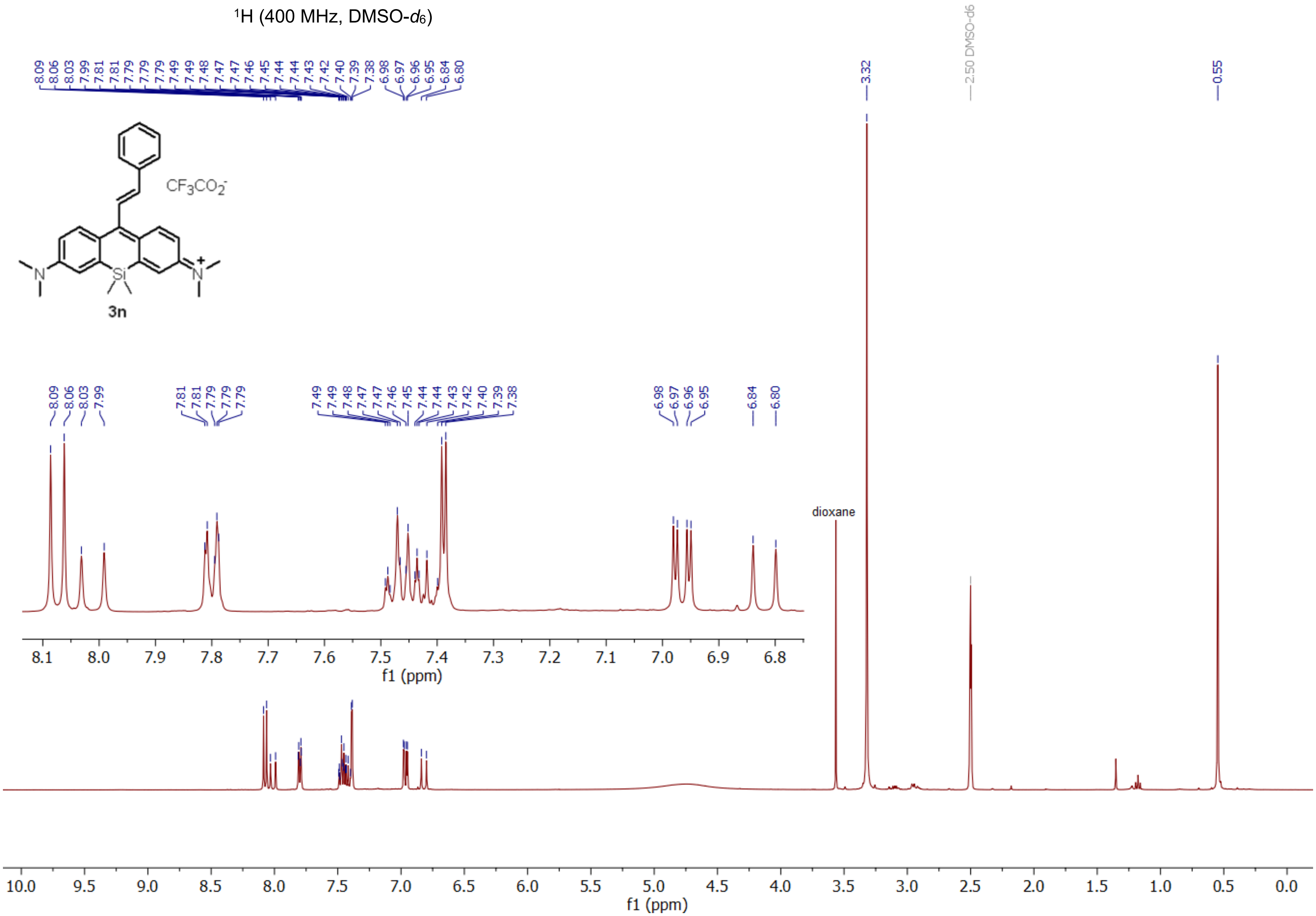

1H (400 MHz, DMSO-d6)
8.09 8.06 8.03 7.99 7.81 7.81 7.79 7.79 7.79 7.49 7.49 7.48 7.47 7.47 7.46 7.45 7.44 7.44 7.43 7.42 7.40 7.39 7.38 6.98 6.97 6.96 6.95 6.84 6.80
3.32
2.50 DMSO-d6
0.55
CF3CO2-
Si
N
N+
3n
dioxane
f1 (ppm)
f1 (ppm)

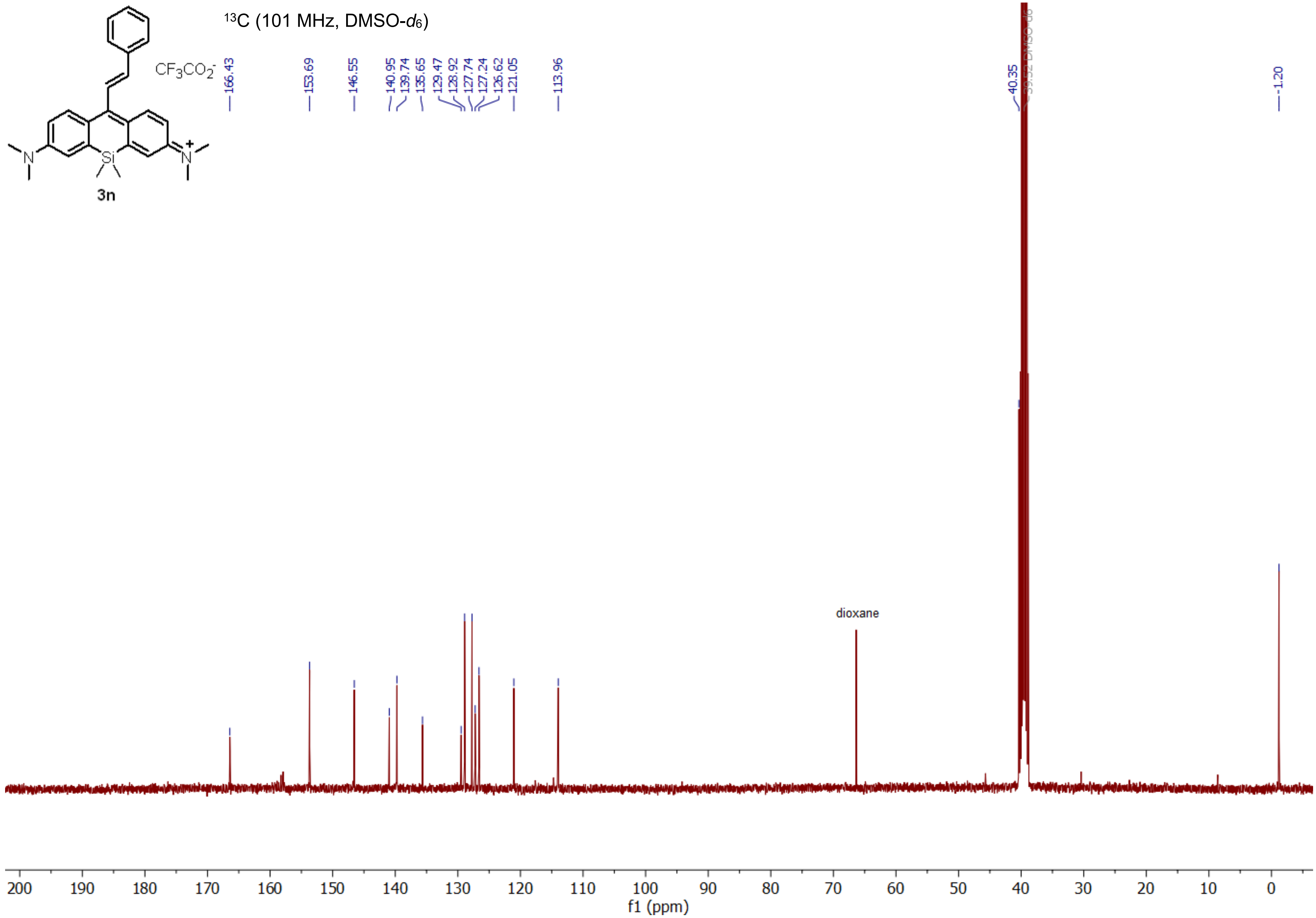
CF3CO2-
3n
13C (101 MHz, DMSO-d6)
166.43
153.69
146.55
140.95
139.74
135.65
129.47
128.92
127.74
127.24
126.62
121.05
113.96
40.35
39.52 DMSO-d6
-1.20
dioxane
200
190
180
170
160
150
140
130
120
110
100
90
80
70
60
50
40
30
20
10
0
f1 (ppm)

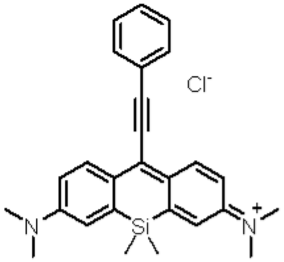

3o

^{1}H (400 MHz, CD_3OD)

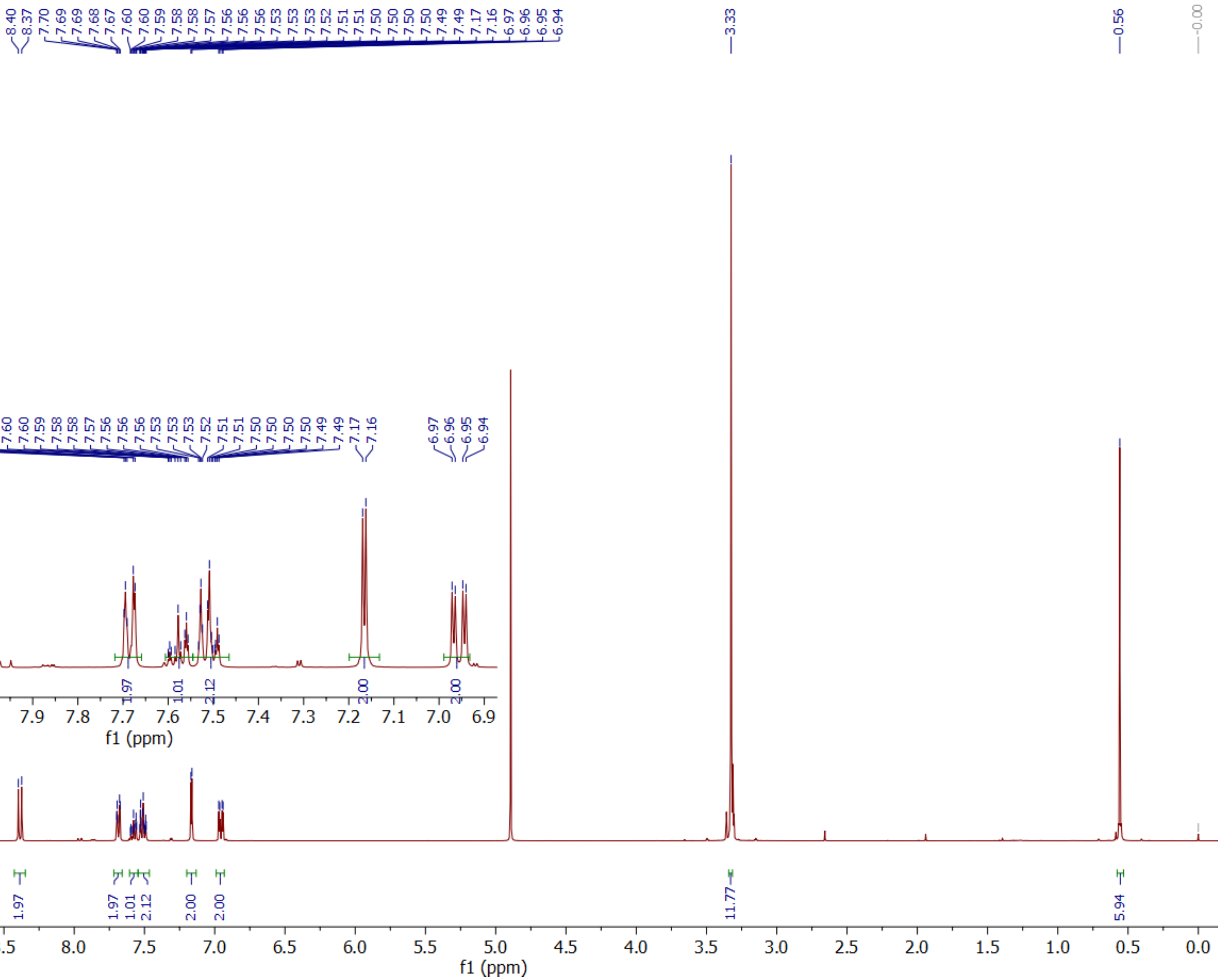

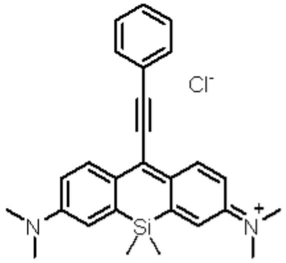

3o

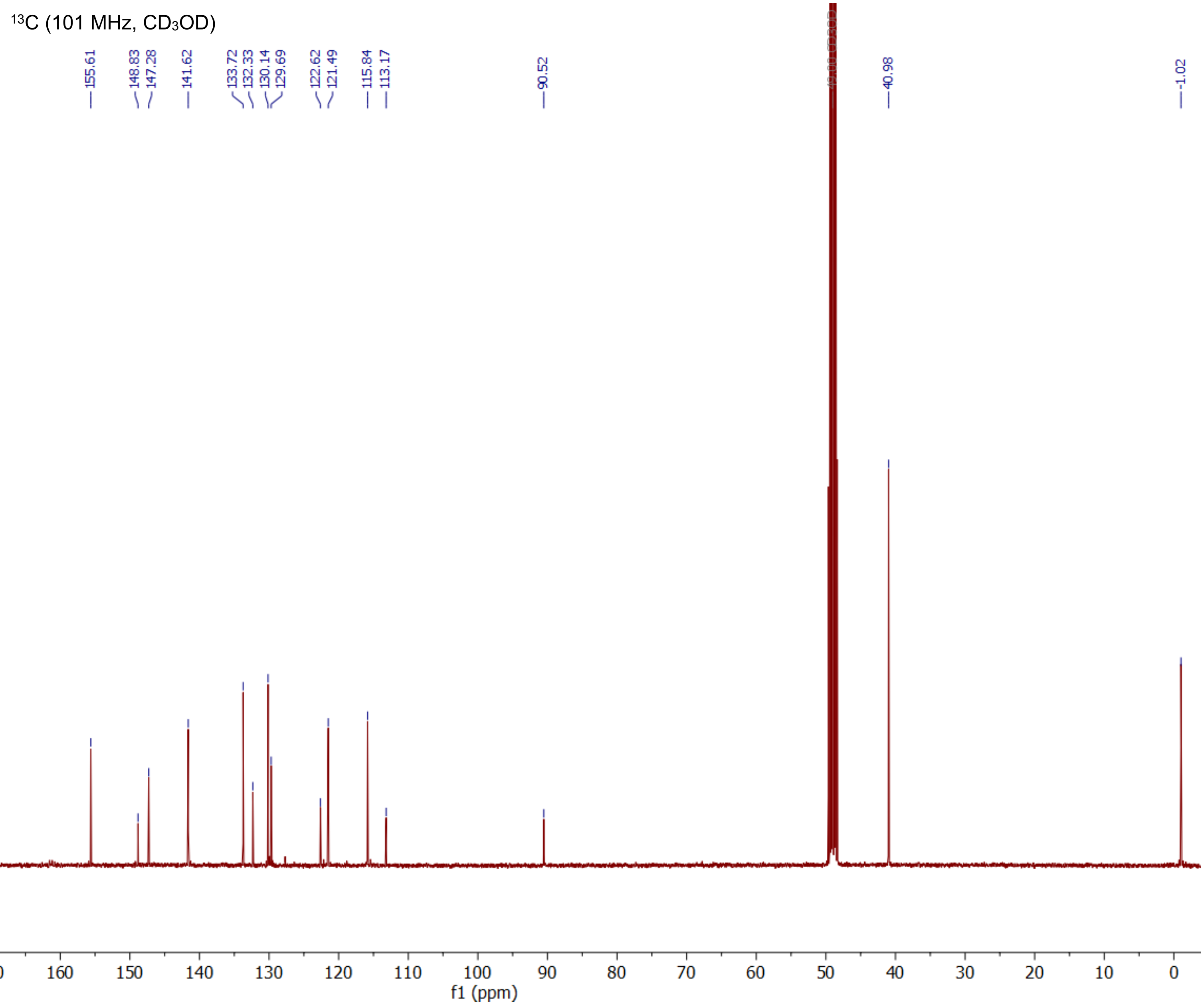

13C (101 MHz, CD3OD)
155.61
148.83
147.28
141.62
133.72
132.33
130.14
129.69
122.62
121.49
115.84
113.17
90.52
40.98
-1.02
200
190
180
170
160
150
140
130
120
110
100
90
80
70
60
50
40
30
20
10
0
f1 (ppm)

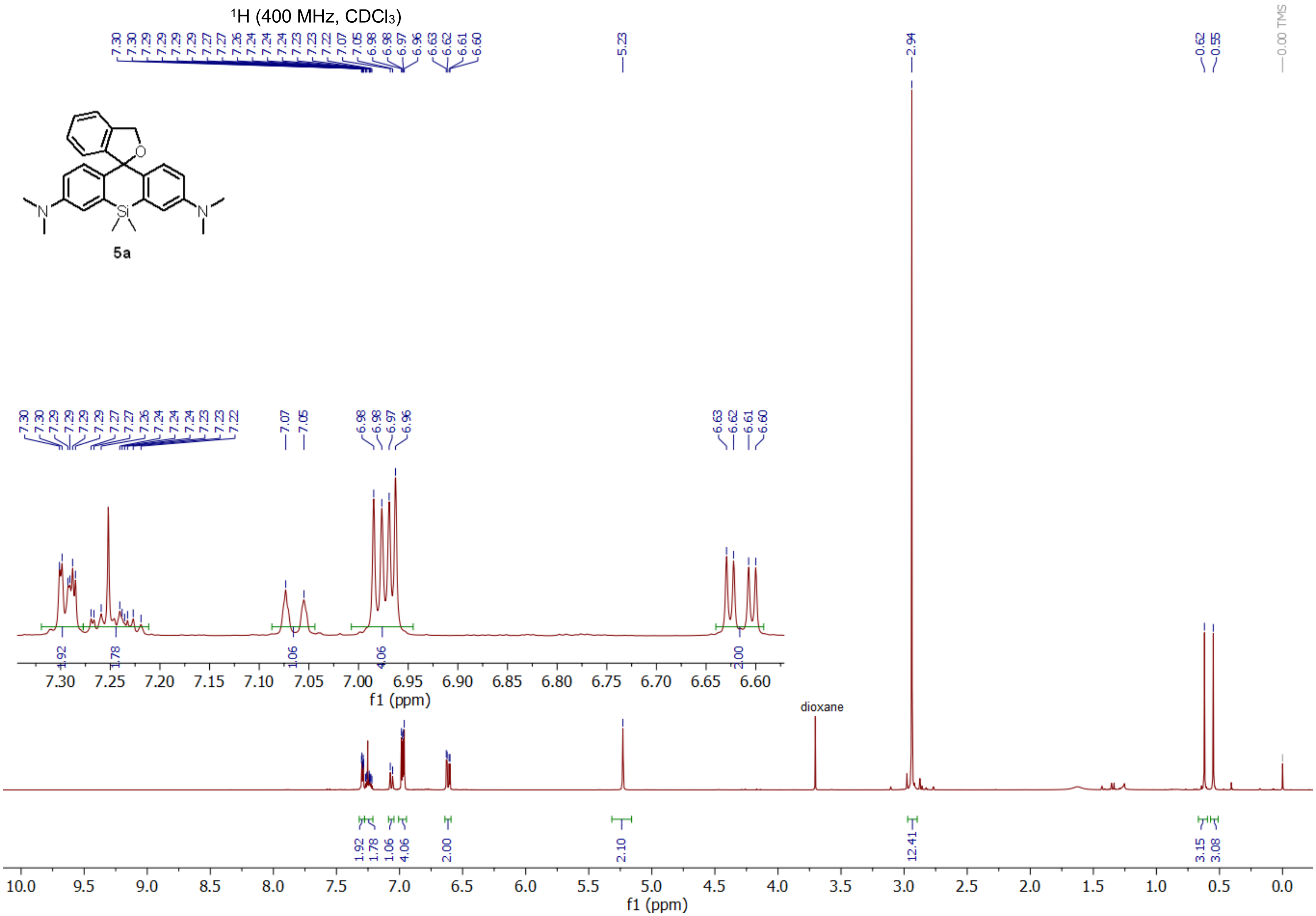

1H (400 MHz, CDCl3)
7.30
7.30
7.29
7.29
7.29
7.29
7.27
7.27
7.26
7.24
7.24
7.24
7.23
7.23
7.22
7.07
7.05
6.98
6.98
6.97
6.96
6.63
6.62
6.61
6.60
5.23
2.94
0.62
0.55
0.00 TMS
5a
1.92
1.78
1.06
4.06
2.00
7.30
7.25
7.20
7.15
7.10
7.05
7.00
6.95
6.90
6.85
6.80
6.75
6.70
6.65
6.60
f1 (ppm)
dioxane
2.10
12.41
3.15
3.08
10.0
9.5
9.0
8.5
8.0
7.5
7.0
6.5
6.0
5.5
5.0
4.5
4.0
3.5
3.0
2.5
2.0
1.5
1.0
0.5
0.0
f1 (ppm)

^1H (400 MHz, $CDCl_3$)

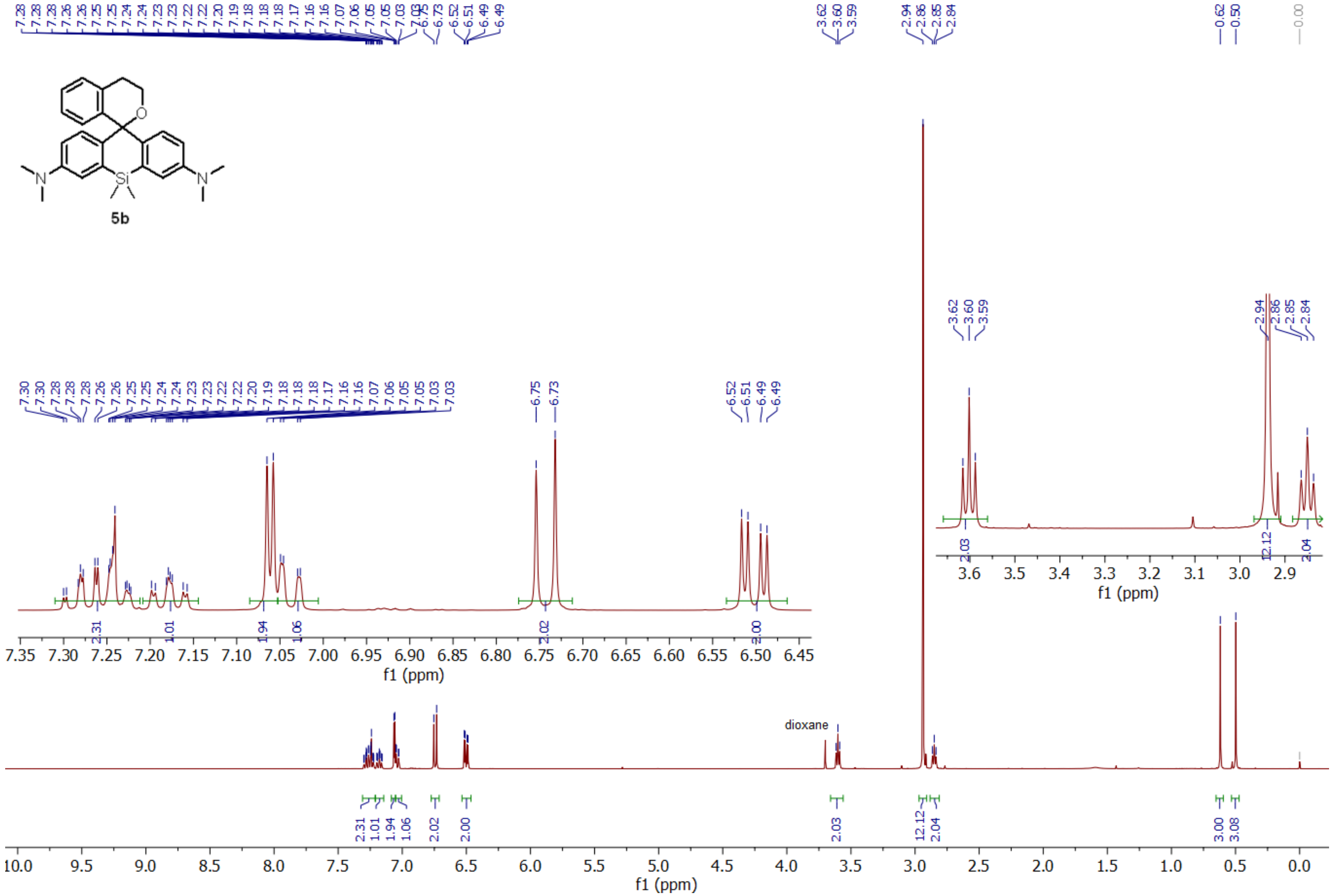

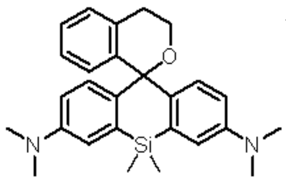

5b

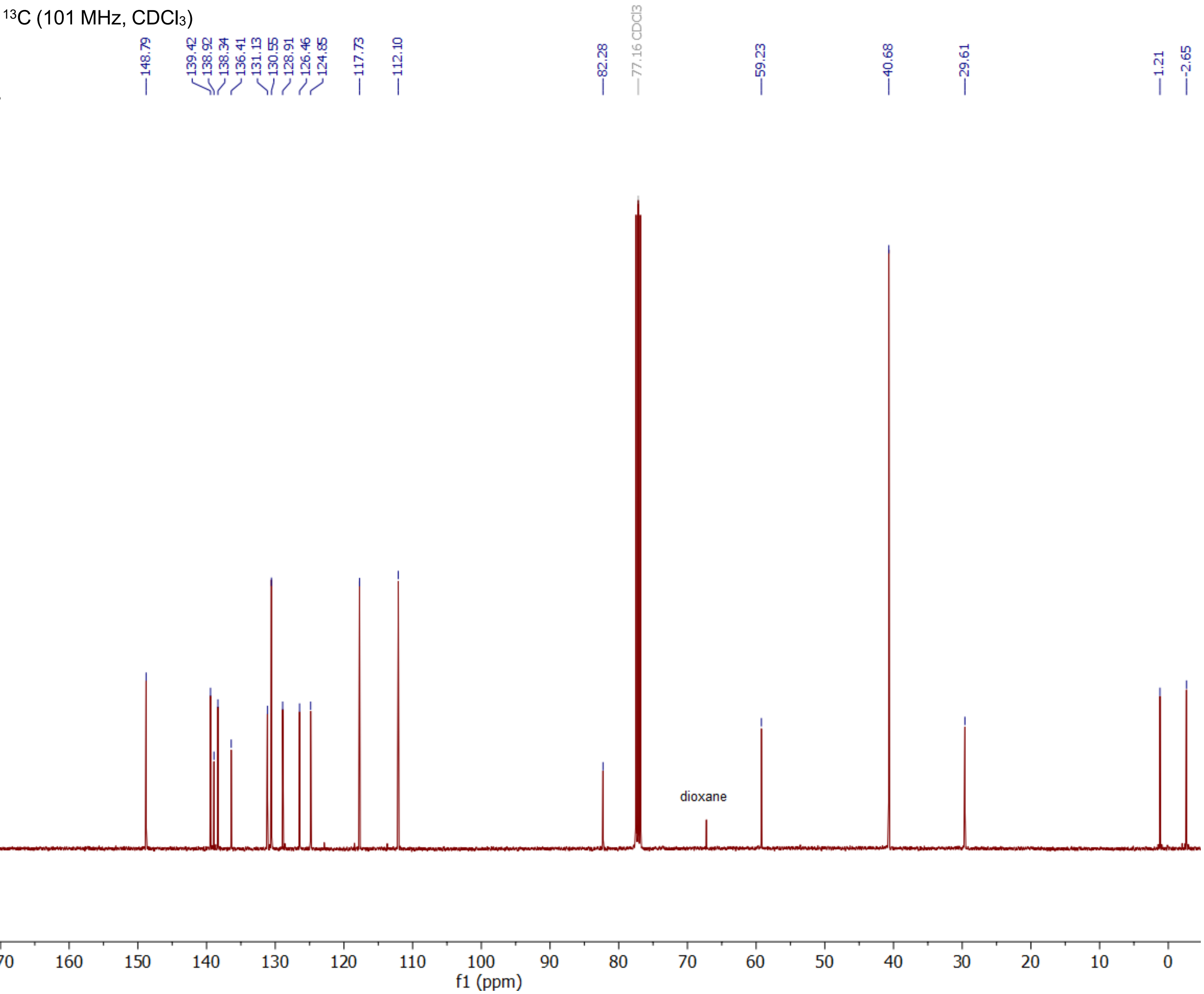

^{13}C (101 MHz, $CDCl_3$)
148.79
139.42
138.92
138.34
136.41
131.13
130.55
128.91
126.46
124.85
117.73
112.10
82.28
77.16 CDCl3
59.23
40.68
29.61
1.21
-2.65
dioxane
200 190 180 170 160 150 140 130 120 110 100 90 80 70 60 50 40 30 20 10 0
f1 (ppm)

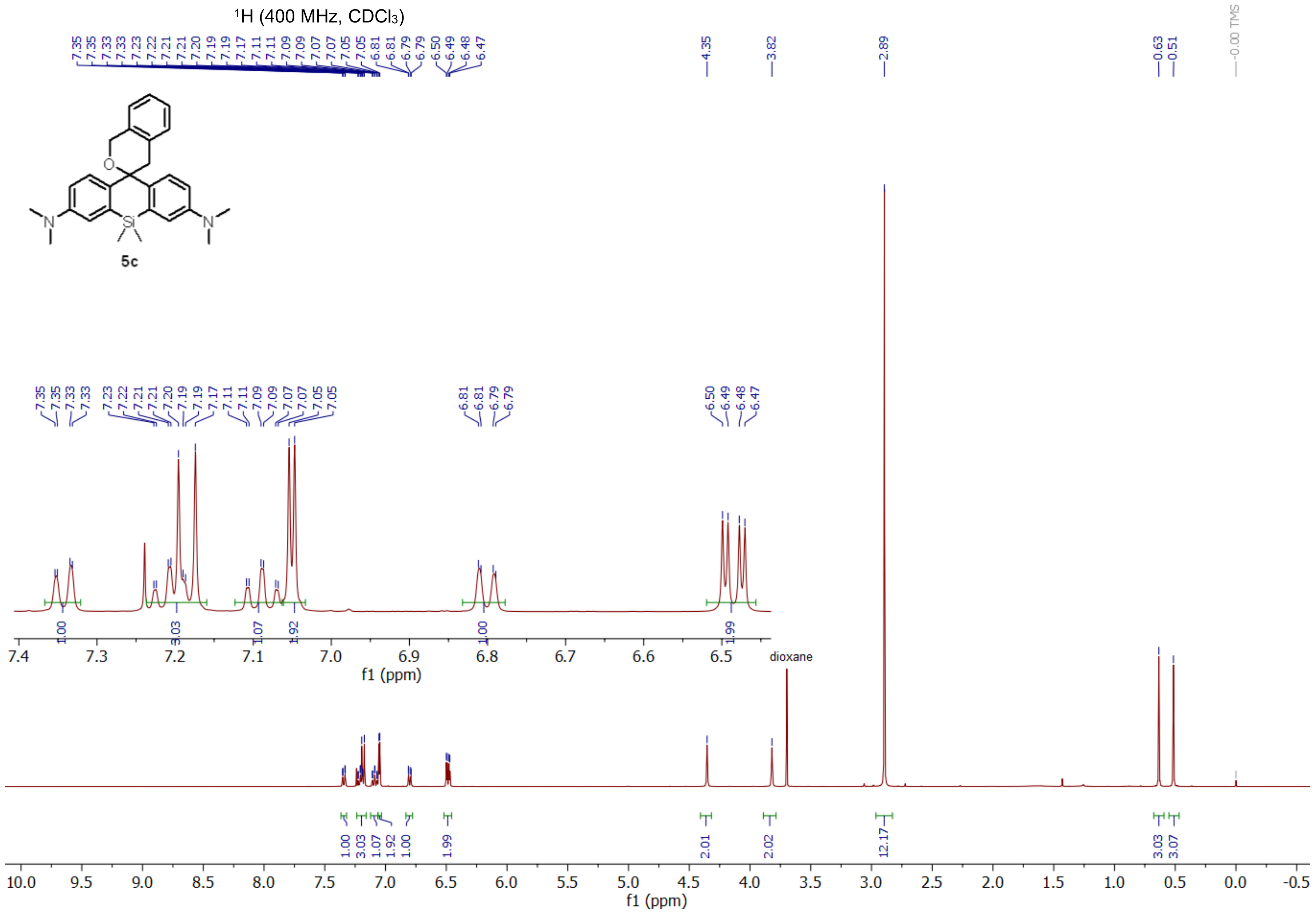

1H (400 MHz, CDCl3)
5c
dioxane
f1 (ppm)

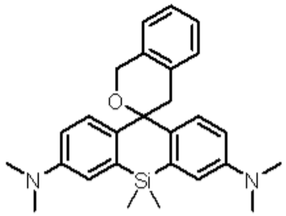

5c

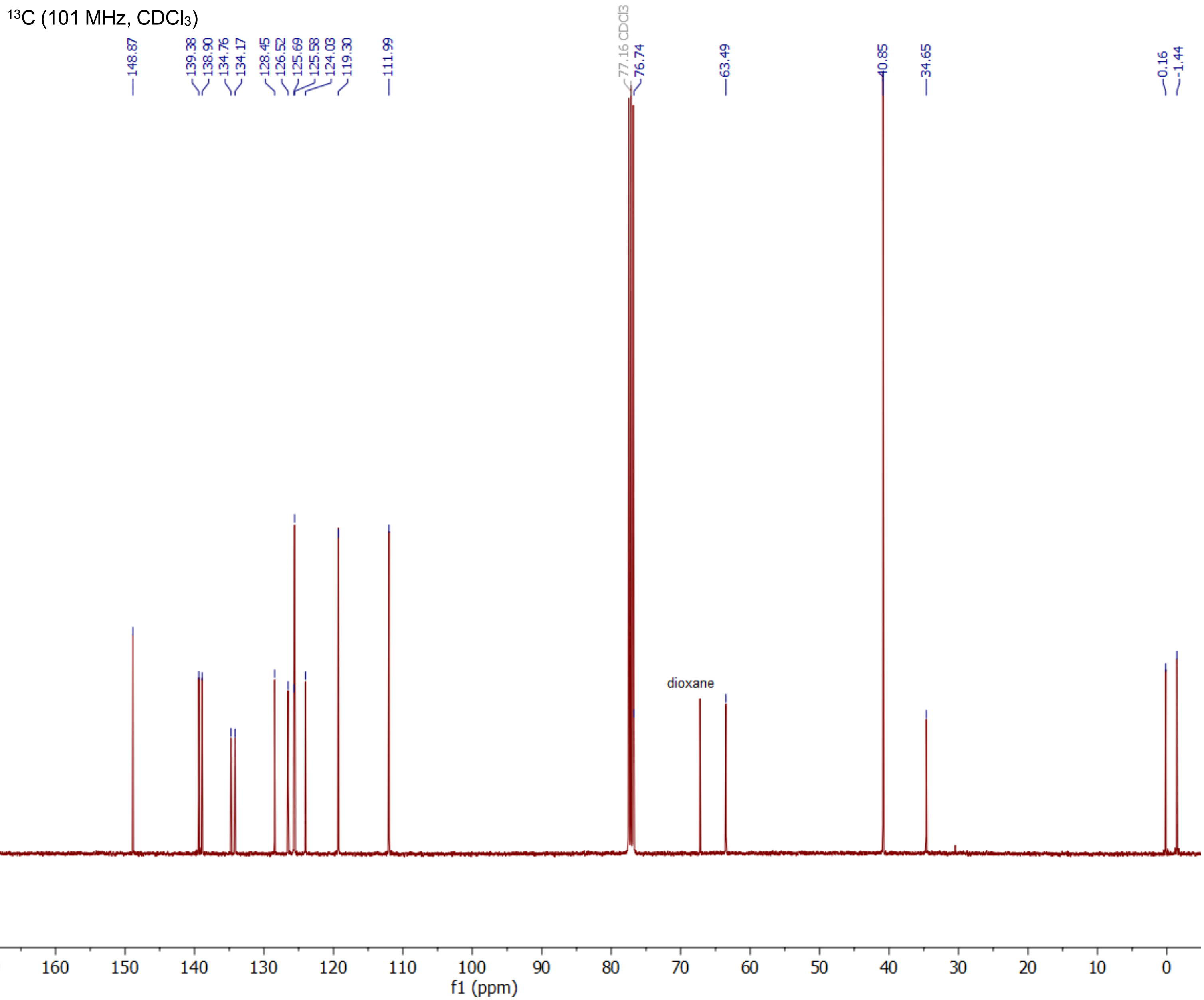

13C (101 MHz, CDCl3)
148.87
139.38
138.90
134.76
134.17
128.45
126.52
125.69
125.58
124.03
119.30
111.99
77.16 CDCl3
76.74
63.49
40.85
34.65
0.16
-1.44
dioxane
200
190
180
170
160
150
140
130
120
110
100
90
80
70
60
50
40
30
20
10
0
f1 (ppm)

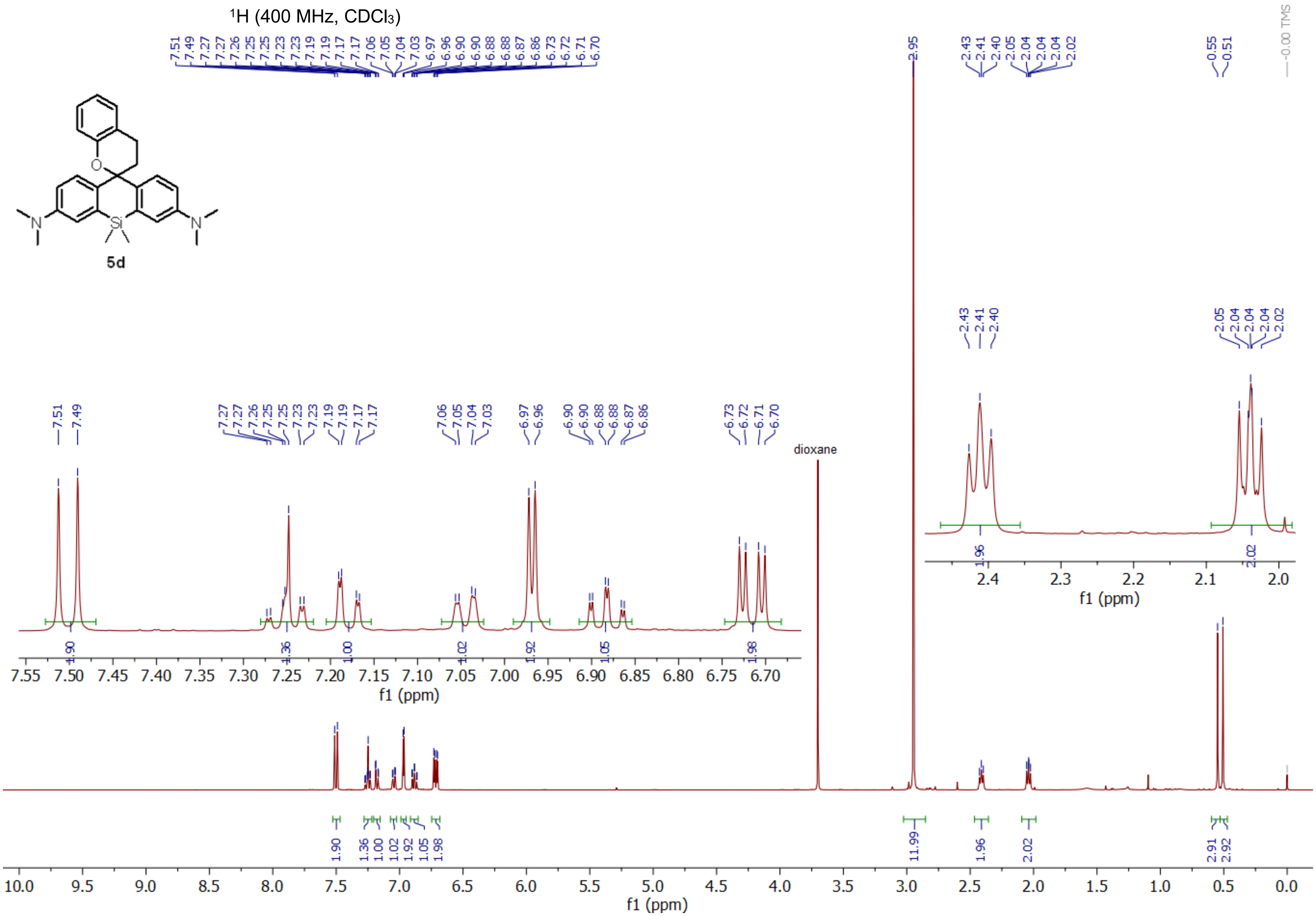

^{1}H (400 MHz, CDCl3)
5d
dioxane
0.00 TMS
f1 (ppm)

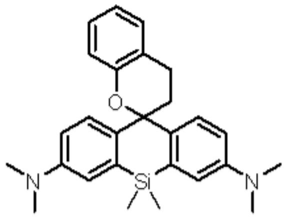

5d

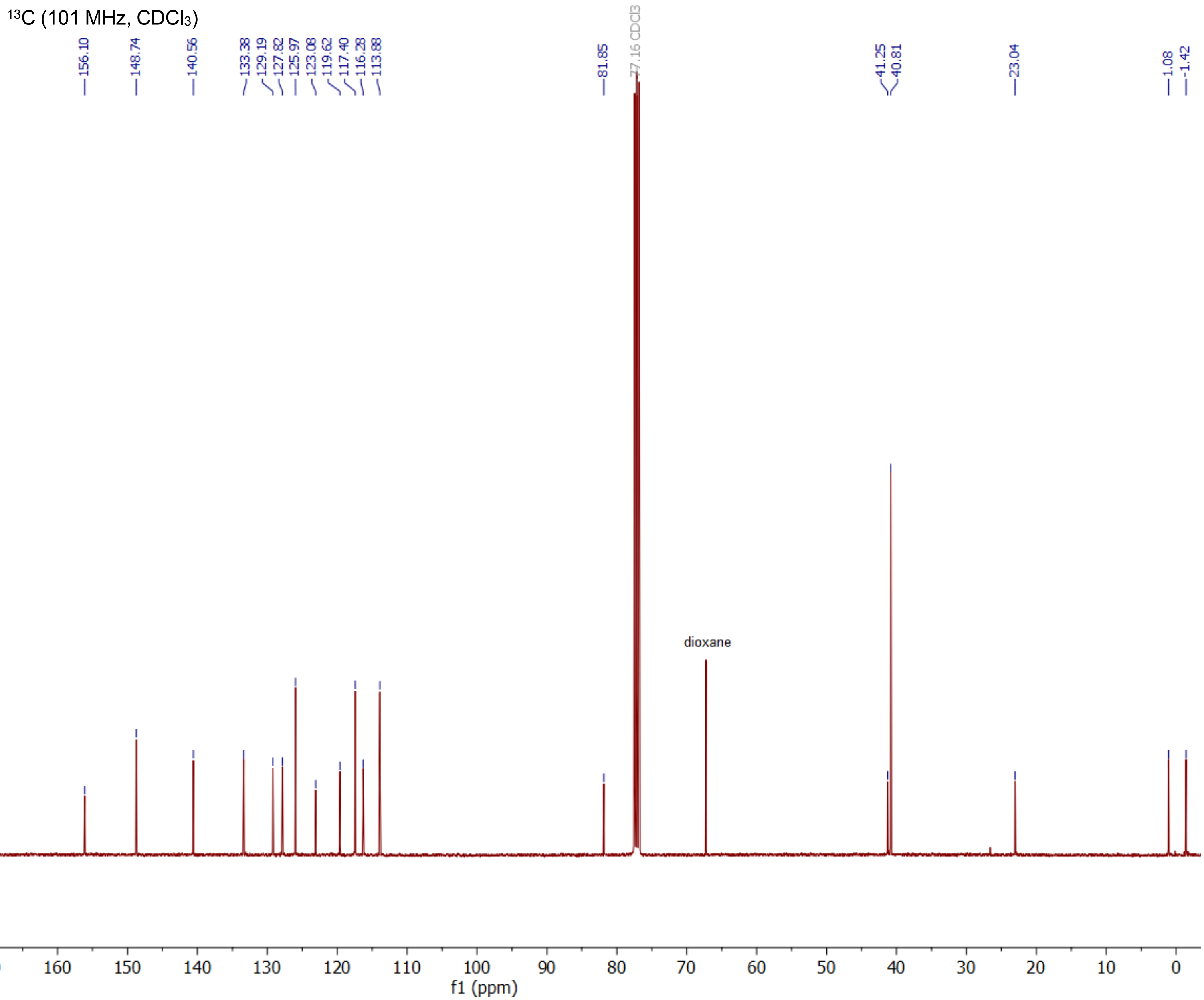

13C (101 MHz, CDCl3)
156.10
148.74
140.56
133.38
129.19
127.82
125.97
123.08
119.62
117.40
116.28
113.88
81.85
77.16 CDCl3
41.25
40.81
23.04
1.08
-1.42
dioxane
200
190
180
170
160
150
140
130
120
110
100
90
80
70
60
50
40
30
20
10
0
f1 (ppm)

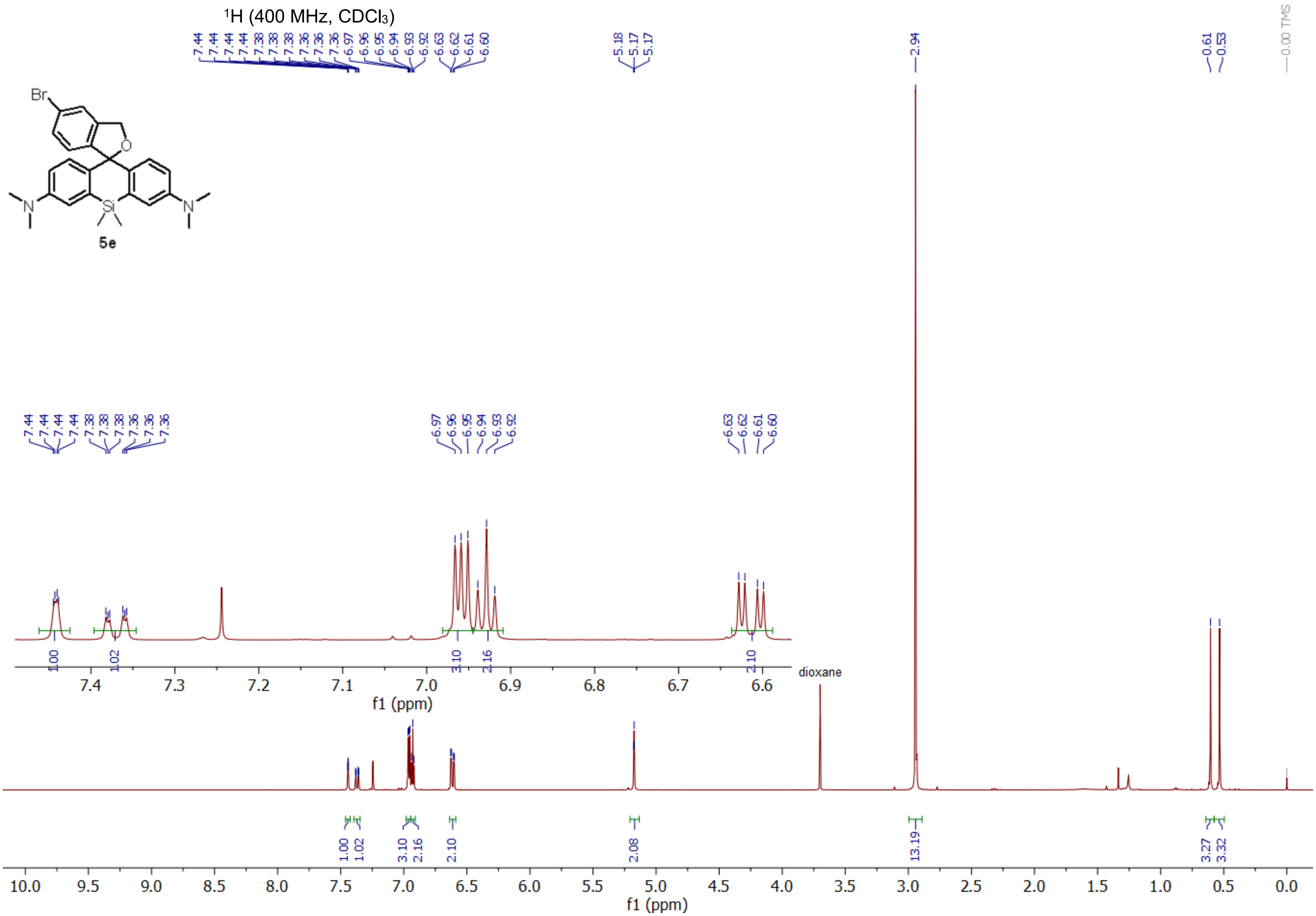

^{1}H (400 MHz, CDCl3)
Br
O
N
Si
N
5e
dioxane
0.00 TMS
f1 (ppm)

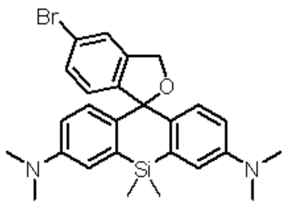

5e

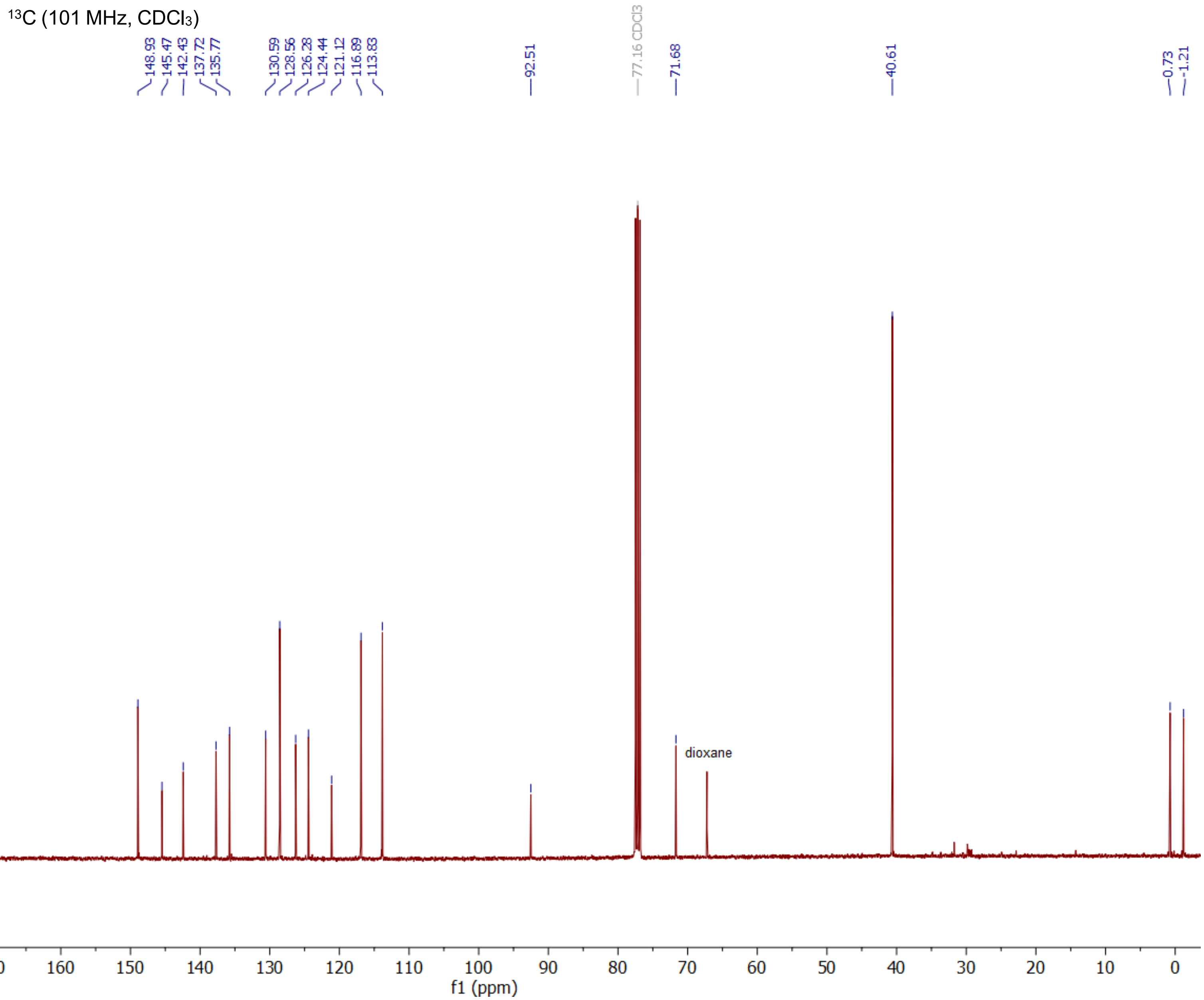

13C (101 MHz, CDCl3)
148.93
145.47
142.43
137.72
135.77
130.59
128.56
126.28
124.44
121.12
116.89
113.83
92.51
77.16 CDCl3
71.68
40.61
0.73
-1.21
dioxane
200 190 180 170 160 150 140 130 120 110 100 90 80 70 60 50 40 30 20 10 0
f1 (ppm)

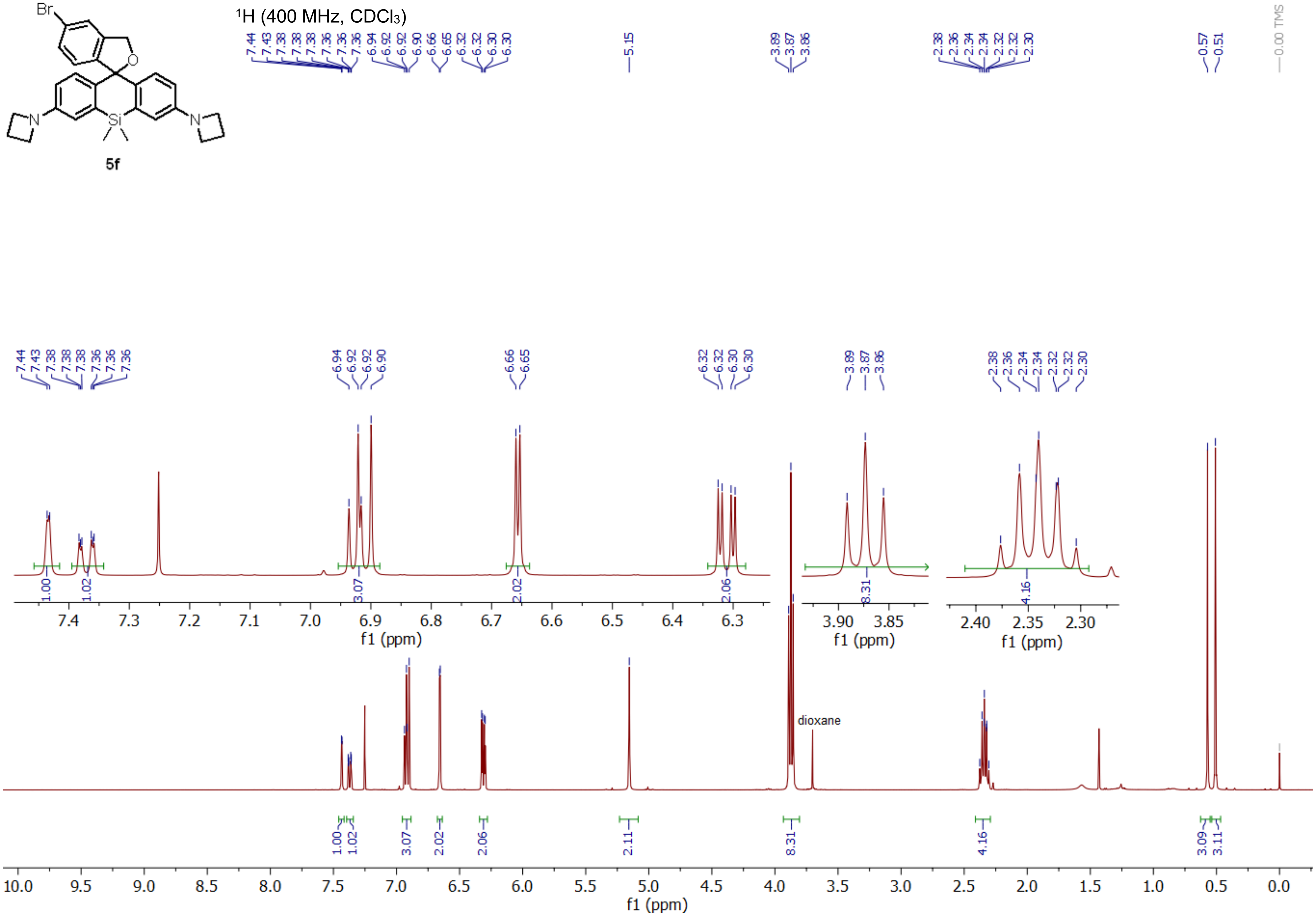

Br
O
N
Si
N
5f
1H (400 MHz, CDCl3)
7.44 7.43 7.38 7.38 7.38 7.38 7.36 7.36 7.36 6.94 6.92 6.92 6.90 6.66 6.65 6.32 6.32 6.30 6.30
5.15
3.89 3.87 3.86
2.38 2.36 2.34 2.34 2.32 2.32 2.30
0.57 0.51
0.00 TMS
dioxane
1.00 1.02 3.07 2.02 2.06 2.11 8.31 4.16 3.09 3.11
f1 (ppm)

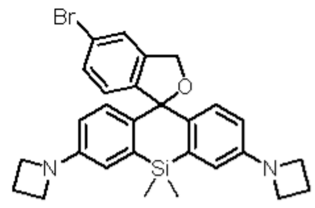
Br
O
N
Si
N

5f

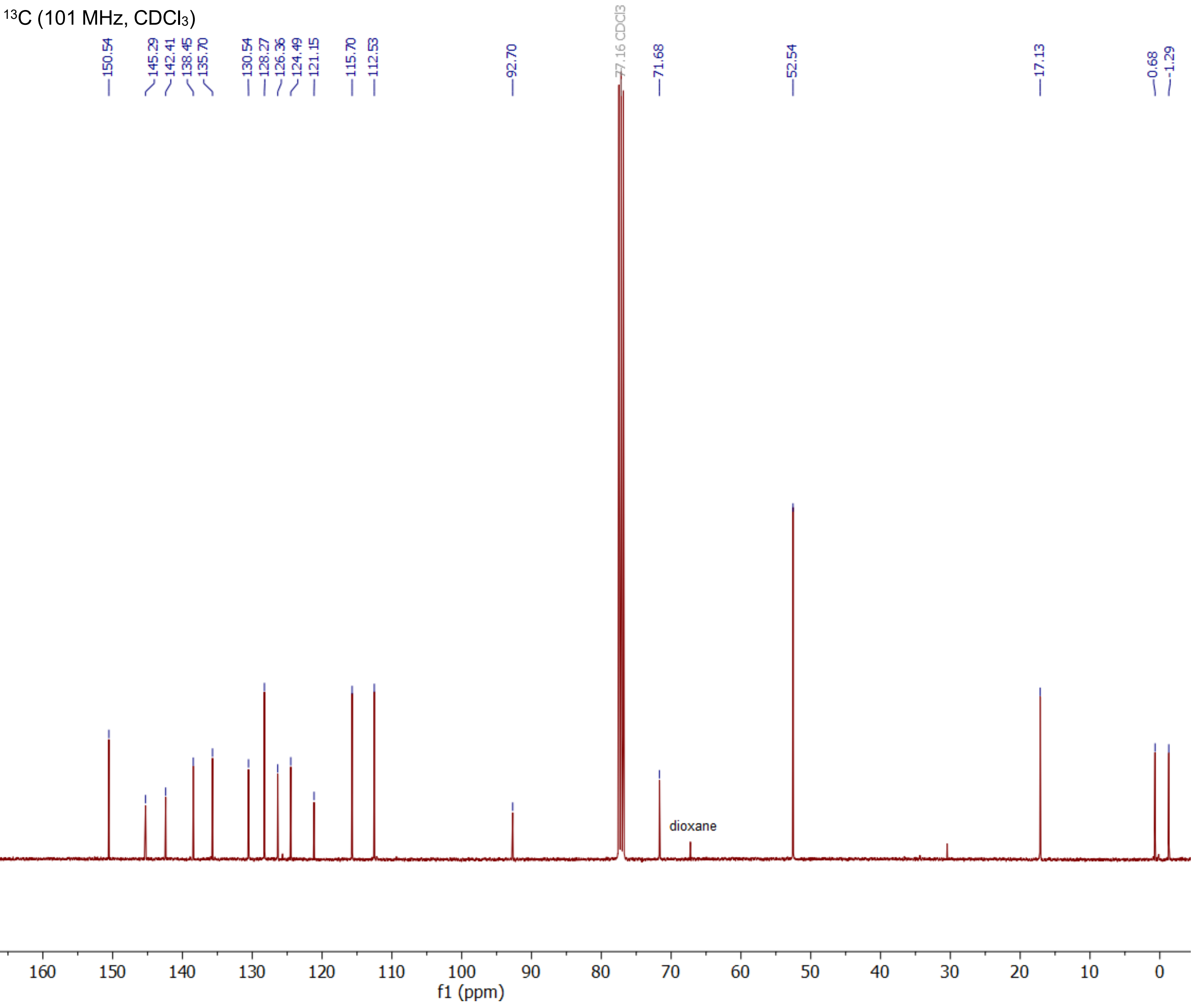
^{13}C (101 MHz, CDCl$_3$)
150.54
145.29
142.41
138.45
135.70
130.54
128.27
126.36
124.49
121.15
115.70
112.53
92.70
77.16 CDCl3
71.68
52.54
17.13
0.68
-1.29
dioxane
200
190
180
170
160
150
140
130
120
110
100
90
80
70
60
50
40
30
20
10
0
f1 (ppm)

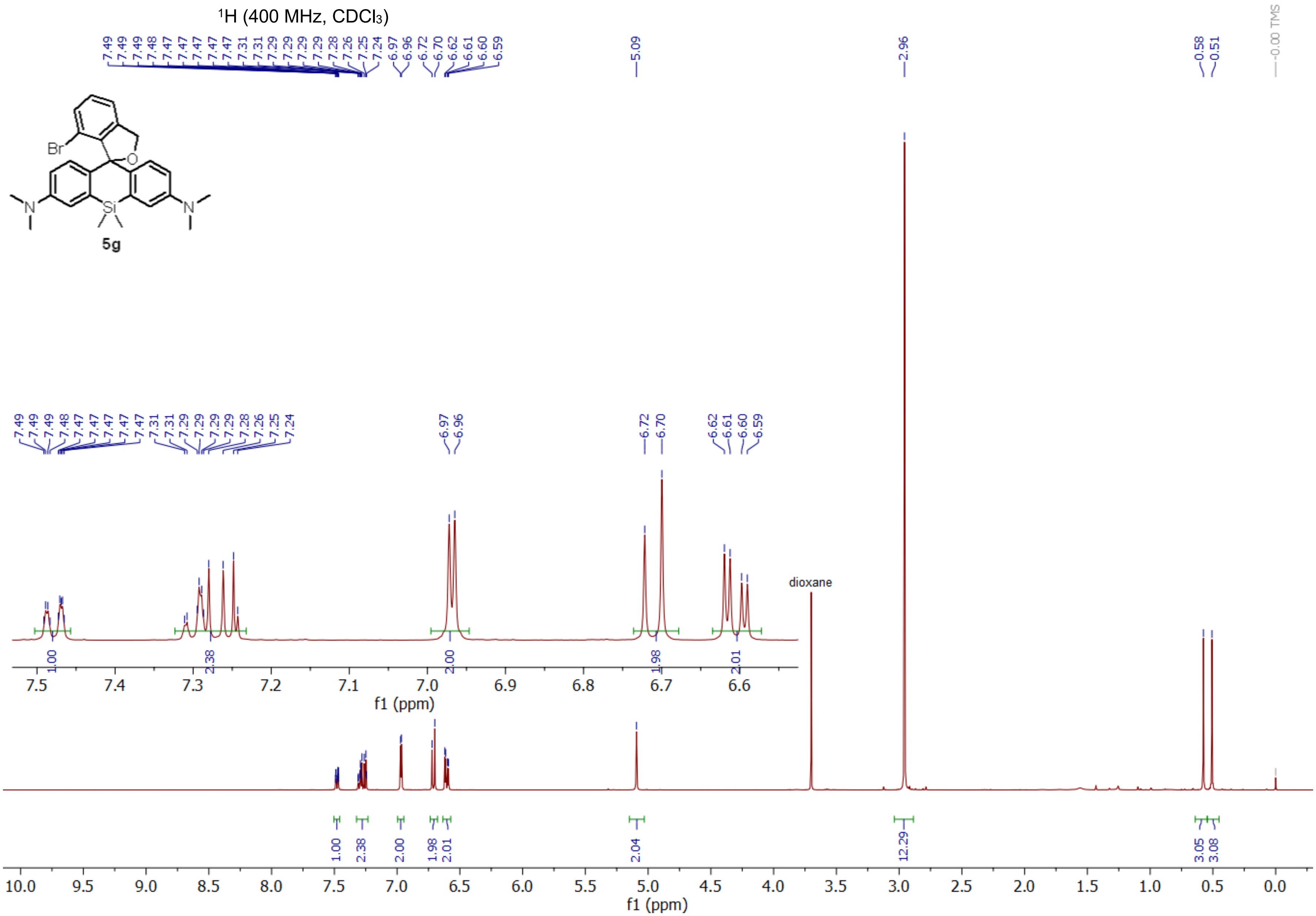

^{1}H (400 MHz, CDCl3)
7.49 7.49 7.49 7.48 7.47 7.47 7.47 7.47 7.47 7.31 7.31 7.29 7.29 7.29 7.29 7.28 7.26 7.25 7.24 6.97 6.96 6.72 6.70 6.62 6.61 6.60 6.59
5.09
2.96
0.58 0.51
0.00 TMS
Br
O
N
Si
N
5g
dioxane
1.00 2.38 2.00 1.98 2.01
f1 (ppm)
1.00 2.38 2.00 1.98 2.01 2.04 12.29 3.05 3.08
f1 (ppm)

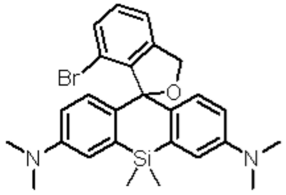

5g

^{13}C (101 MHz, $CDCl_3$)

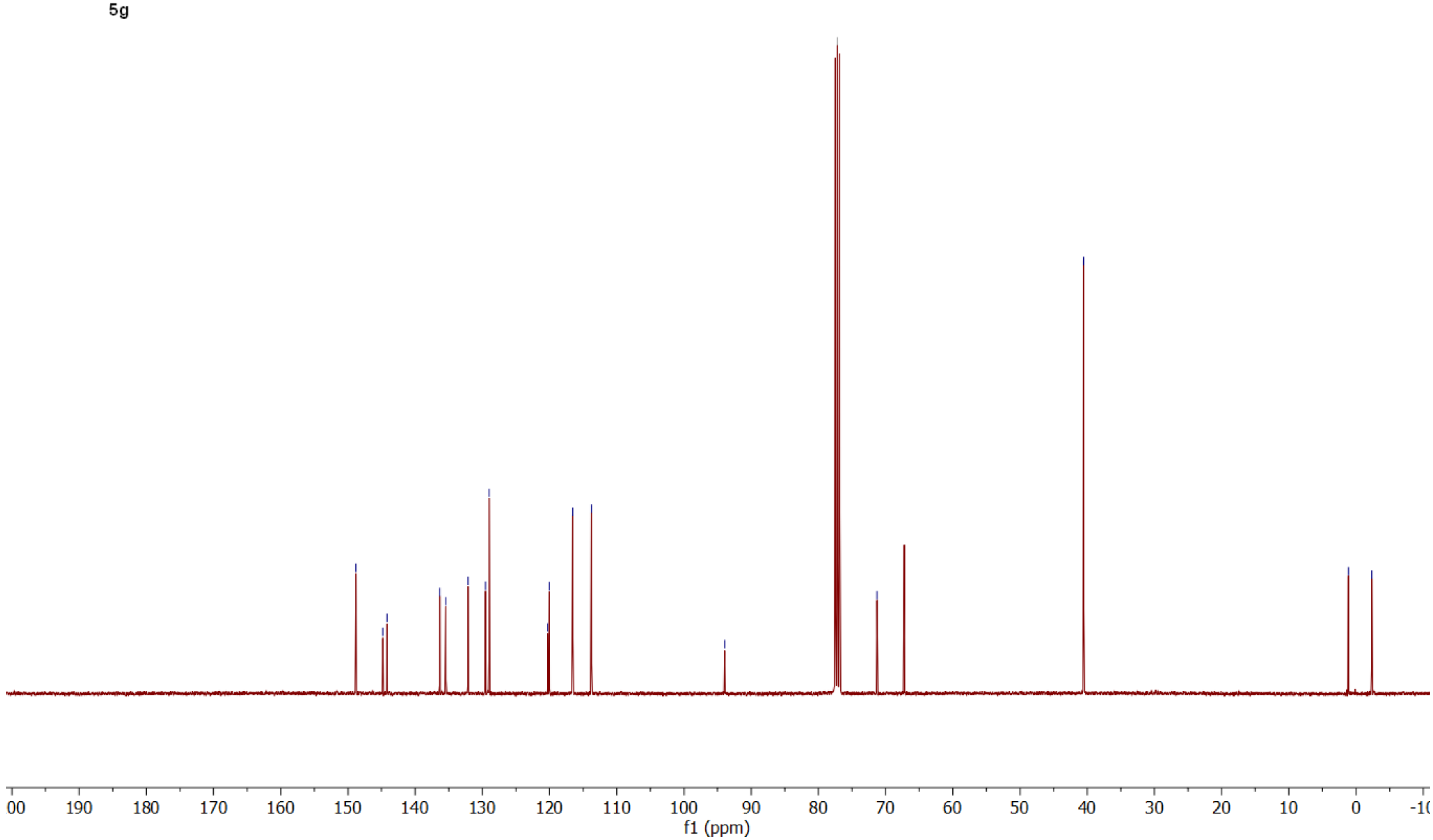

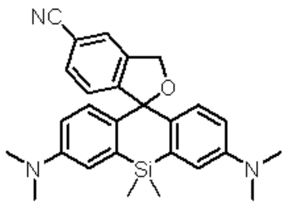

5h

^{1}H (400 MHz, $CDCl_3$)

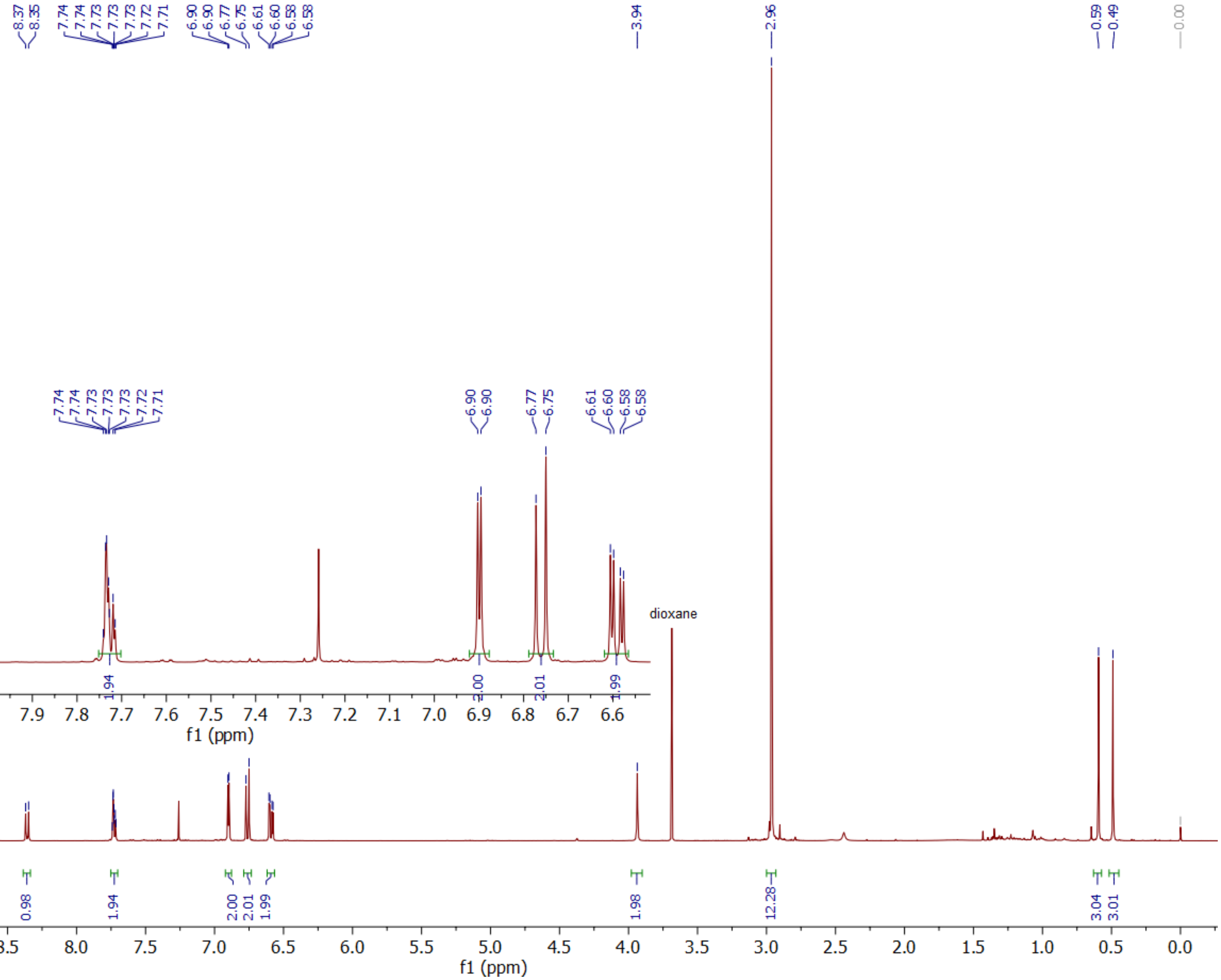

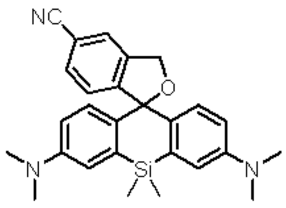

5h

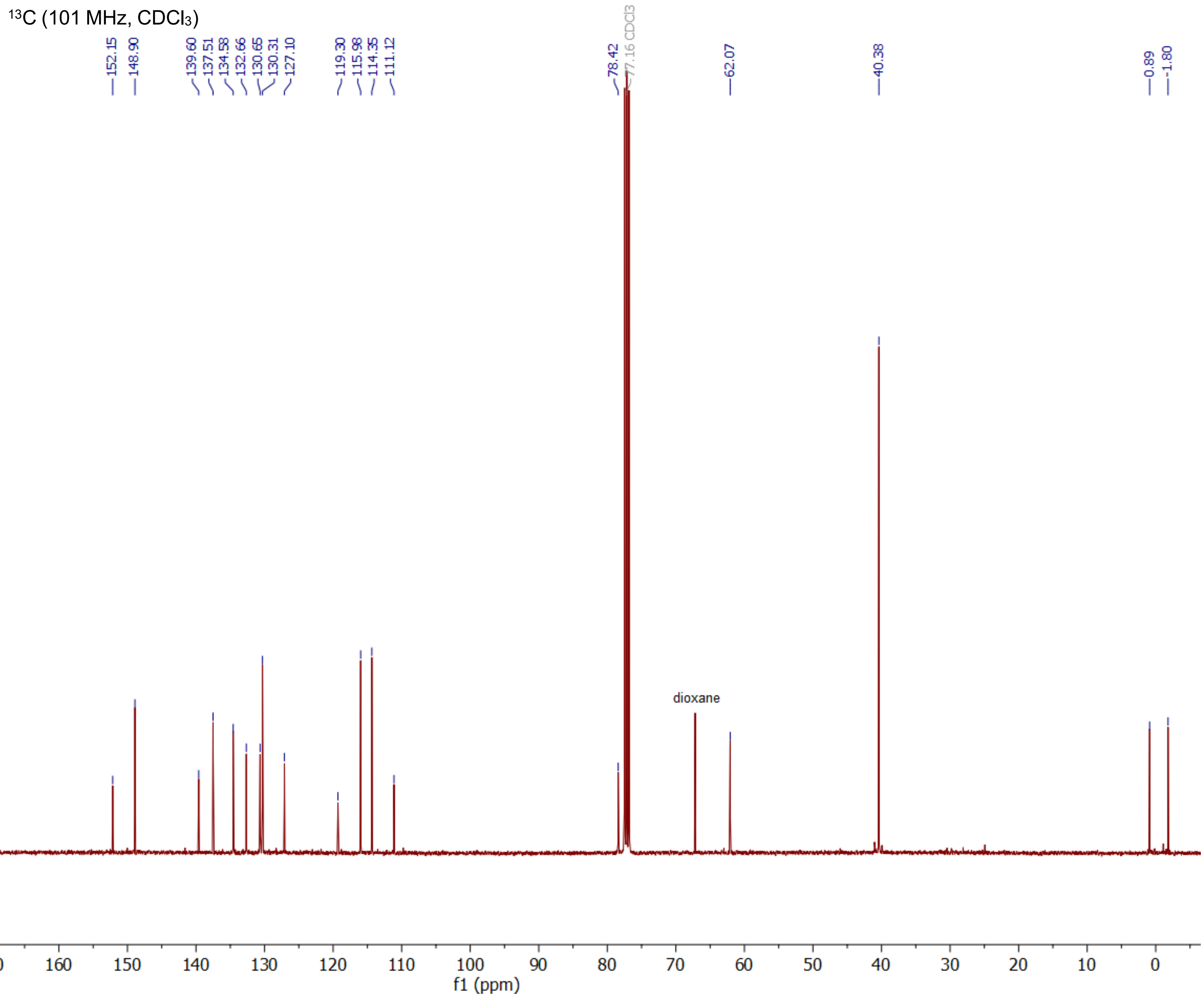

^{13}C (101 MHz, CDCl$_3$)
152.15
148.90
139.60
137.51
134.58
132.66
130.65
130.31
127.10
119.30
115.98
114.35
111.12
78.42
77.16 CDCl3
62.07
40.38
0.89
-1.80
dioxane
200
190
180
170
160
150
140
130
120
110
100
90
80
70
60
50
40
30
20
10
0
f1 (ppm)

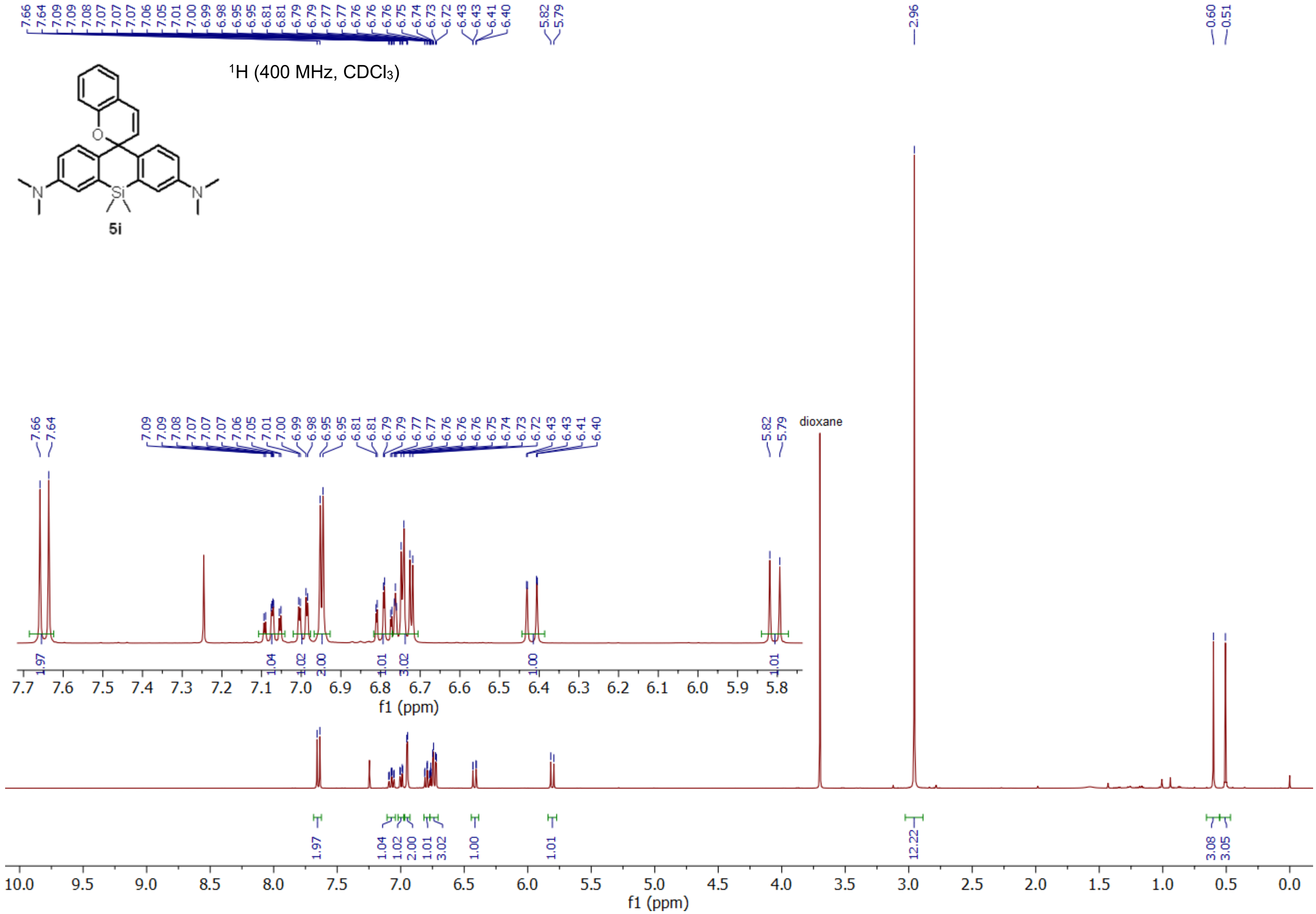

1H (400 MHz, CDCl3)
5i
dioxane
7.66 7.64 7.09 7.09 7.08 7.07 7.07 7.07 7.06 7.05 7.01 7.00 6.99 6.98 6.95 6.95 6.81 6.81 6.79 6.79 6.77 6.77 6.76 6.76 6.76 6.75 6.74 6.73 6.72 6.43 6.43 6.41 6.40 5.82 5.79 2.96 0.60 0.51
1.97 1.04 1.02 2.00 1.01 3.02 1.00 1.01 12.22 3.08 3.05
f1 (ppm)

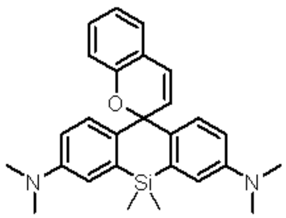

5i

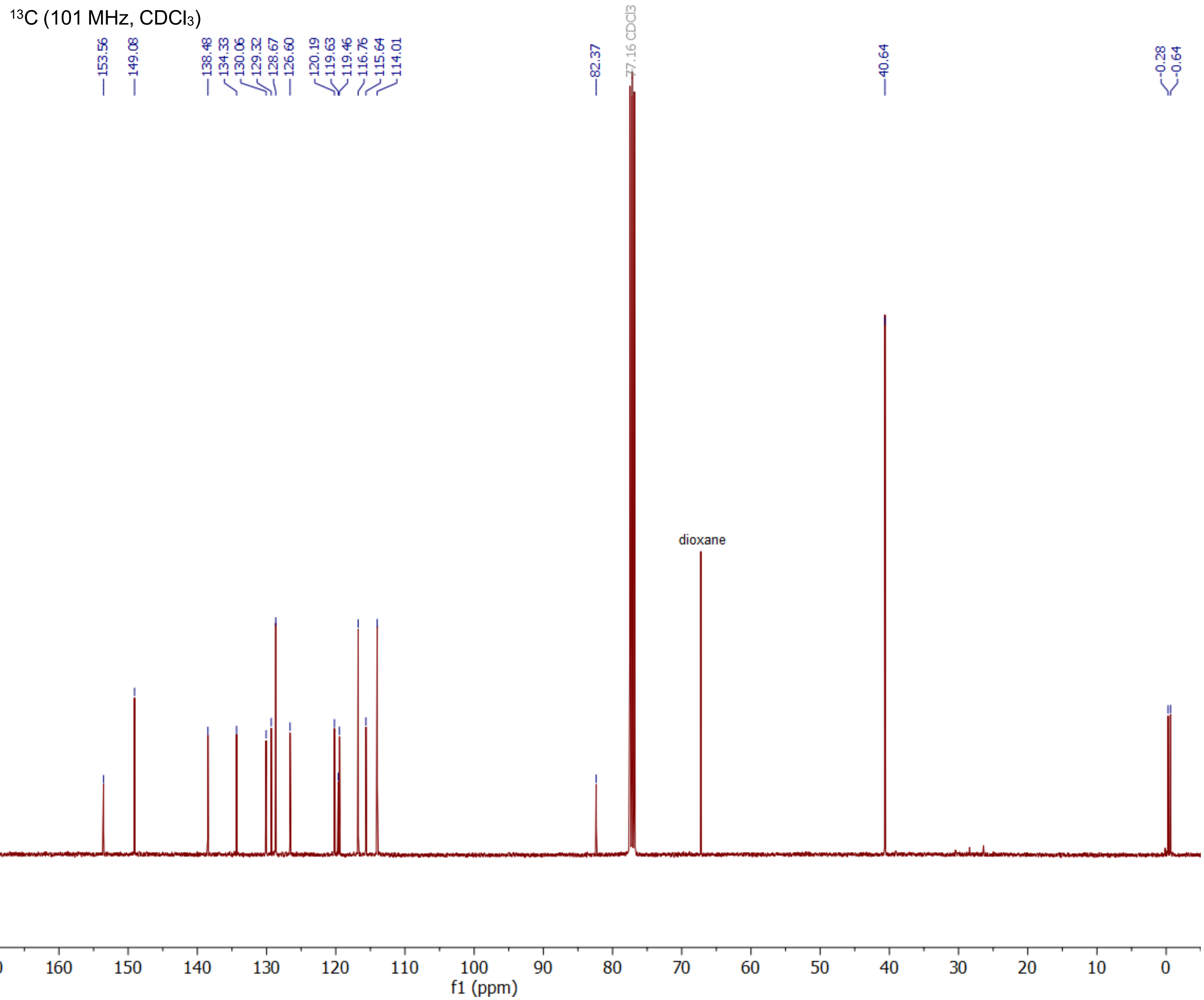

^{13}C (101 MHz, CDCl$_3$)
153.56
149.08
138.48
134.33
130.06
129.32
128.67
126.60
120.19
119.63
119.46
116.76
115.64
114.01
82.37
77.16 CDCl3
40.64
-0.28
-0.64
dioxane
f1 (ppm)

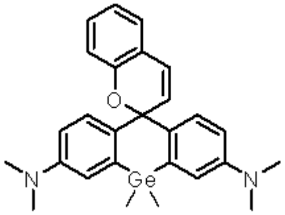

5j

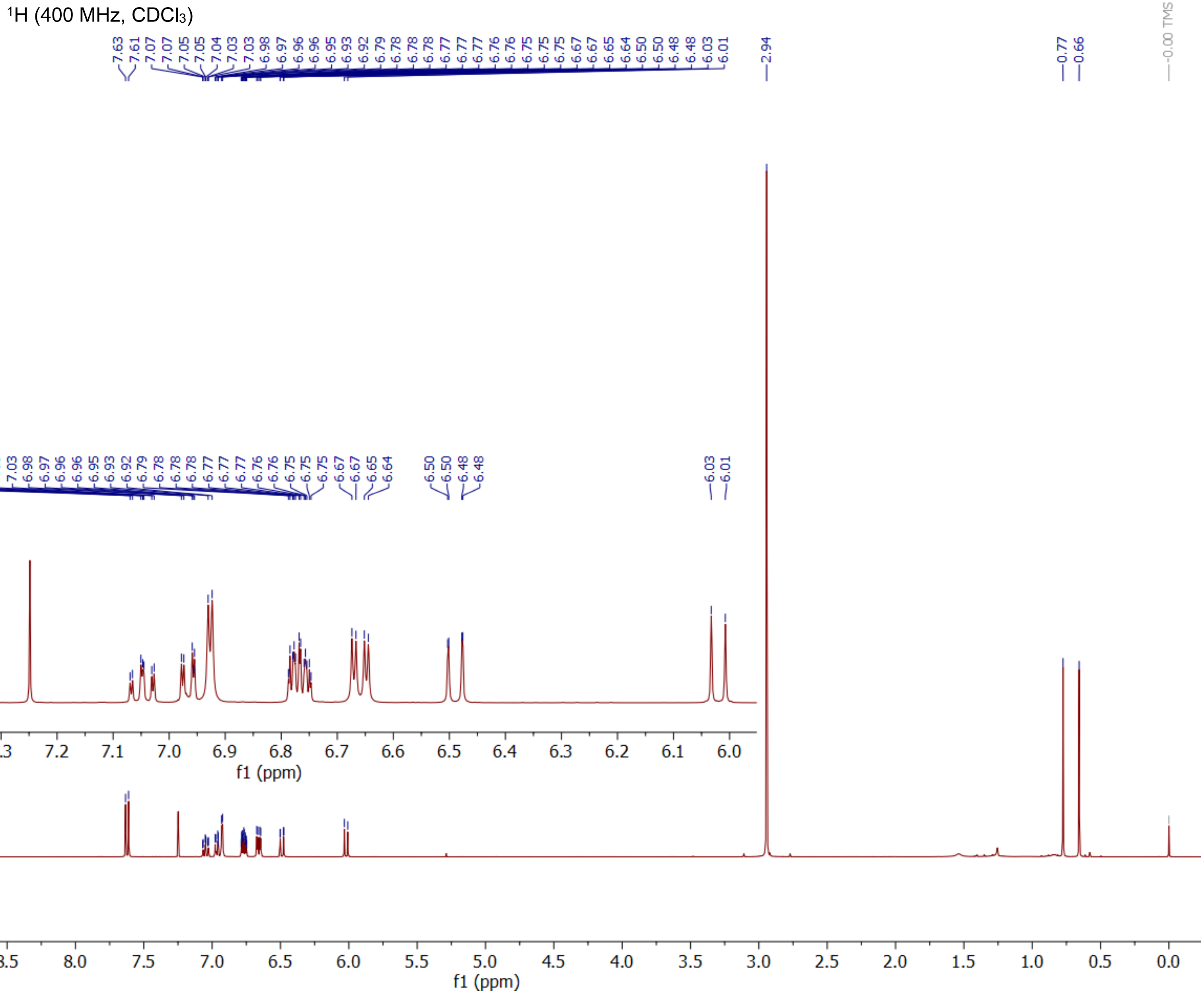

^{1}H (400 MHz, CDCl3)
7.63
7.61
7.07
7.07
7.05
7.05
7.04
7.03
7.03
6.98
6.97
6.96
6.96
6.95
6.93
6.92
6.79
6.78
6.78
6.78
6.77
6.77
6.77
6.76
6.76
6.75
6.75
6.75
6.67
6.67
6.65
6.64
6.50
6.50
6.48
6.48
6.03
6.01
2.94
0.77
0.66
0.00 TMS
f1 (ppm)
f1 (ppm)

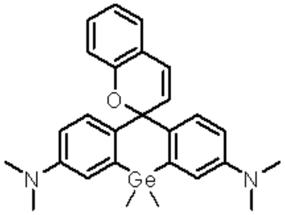
Ge
N
N
O

5j

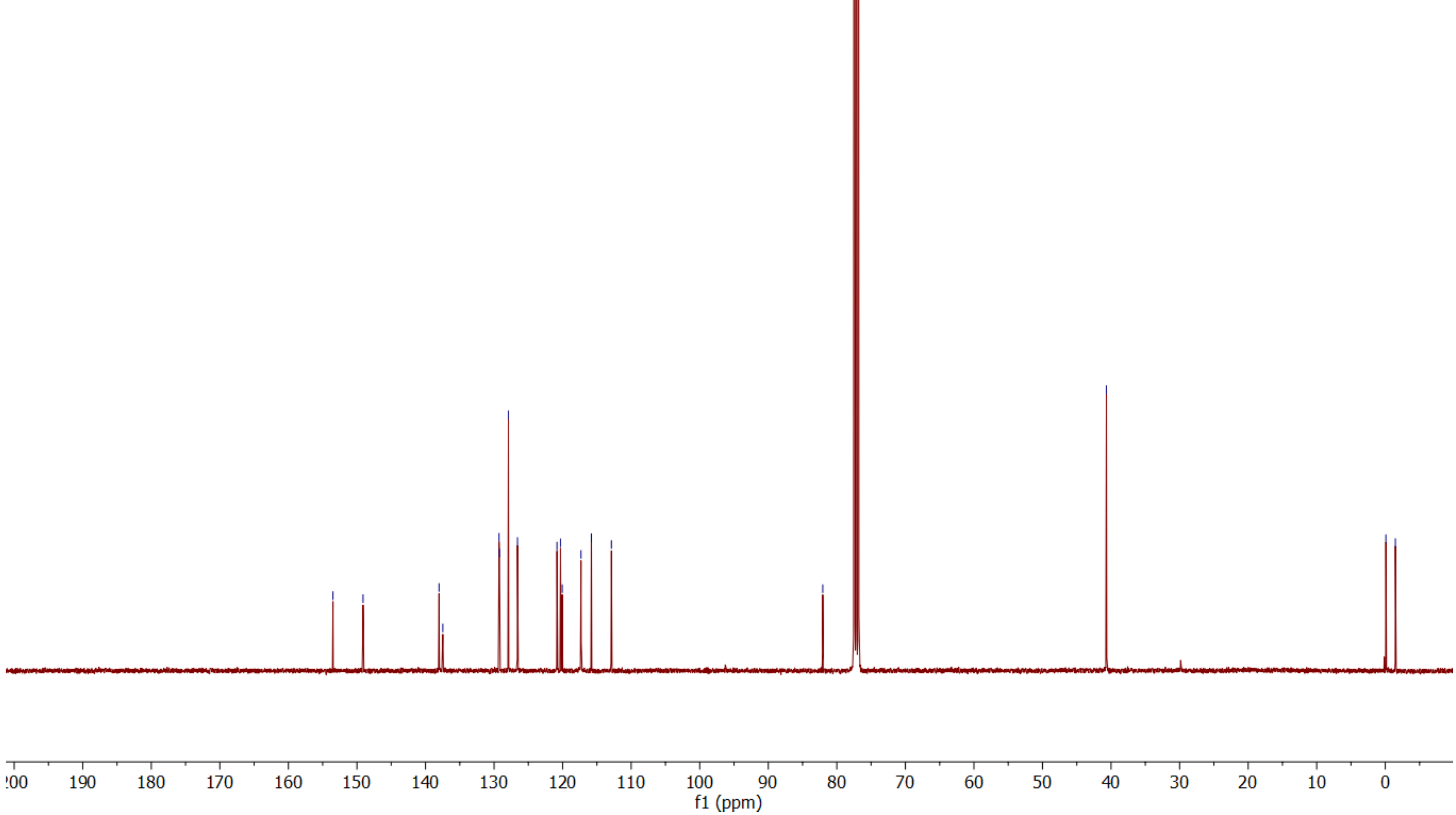
13C (101 MHz, CDCl3)
153.50
149.11
138.02
137.47
129.27
129.23
127.90
126.58
120.82
120.31
120.06
117.32
115.80
112.88
82.05
40.67
-0.09
-1.47
190
180
170
160
150
140
130
120
110
100
90
80
70
60
50
40
30
20
10
0
f1 (ppm)

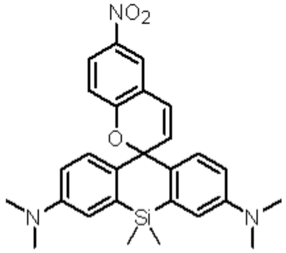

5k

^{1}H (400 MHz, $CDCl_3$)

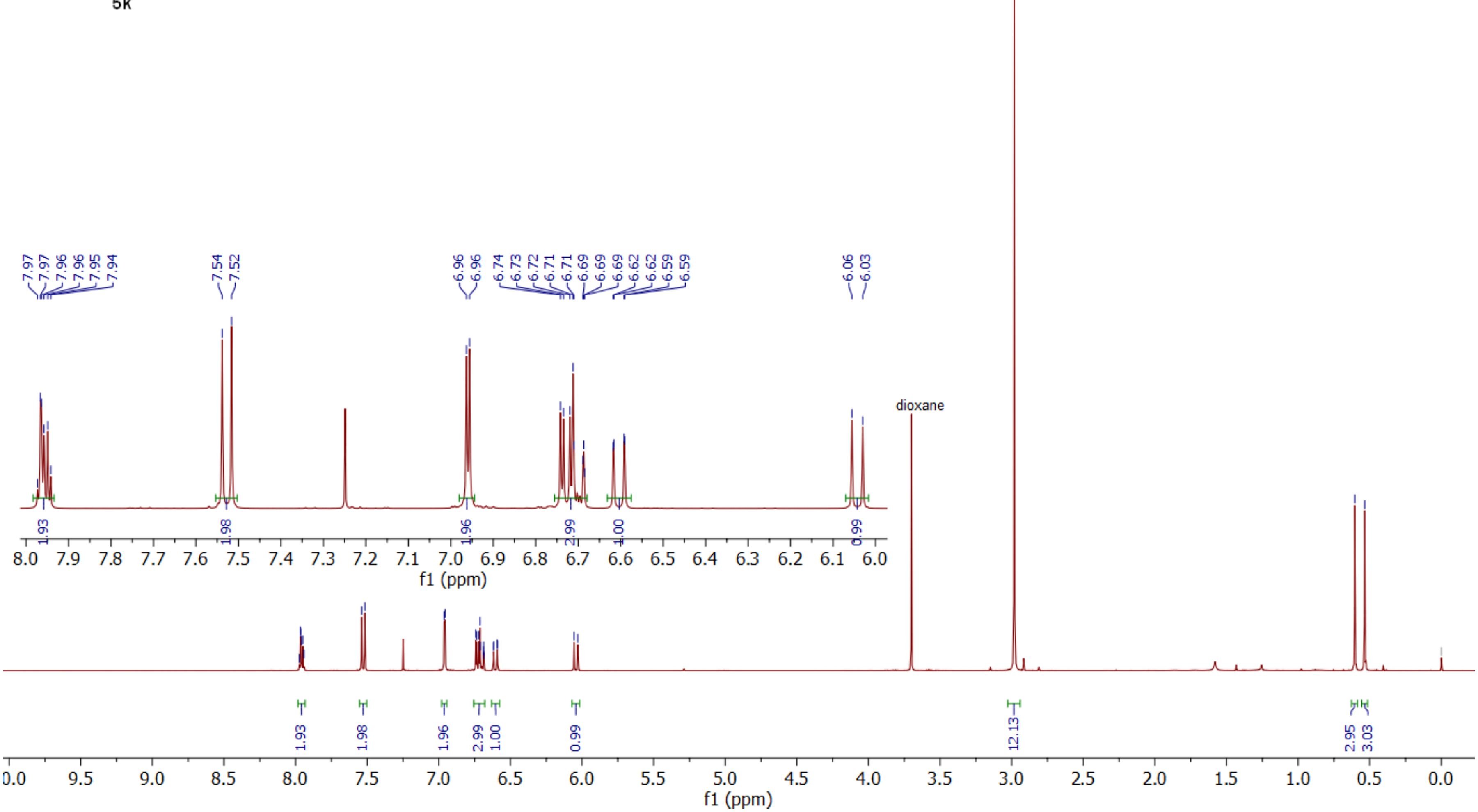

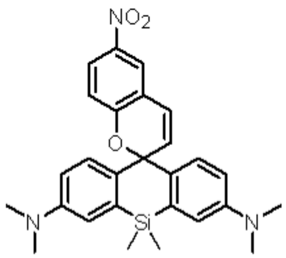
NO2
O
N
N
Si

5k

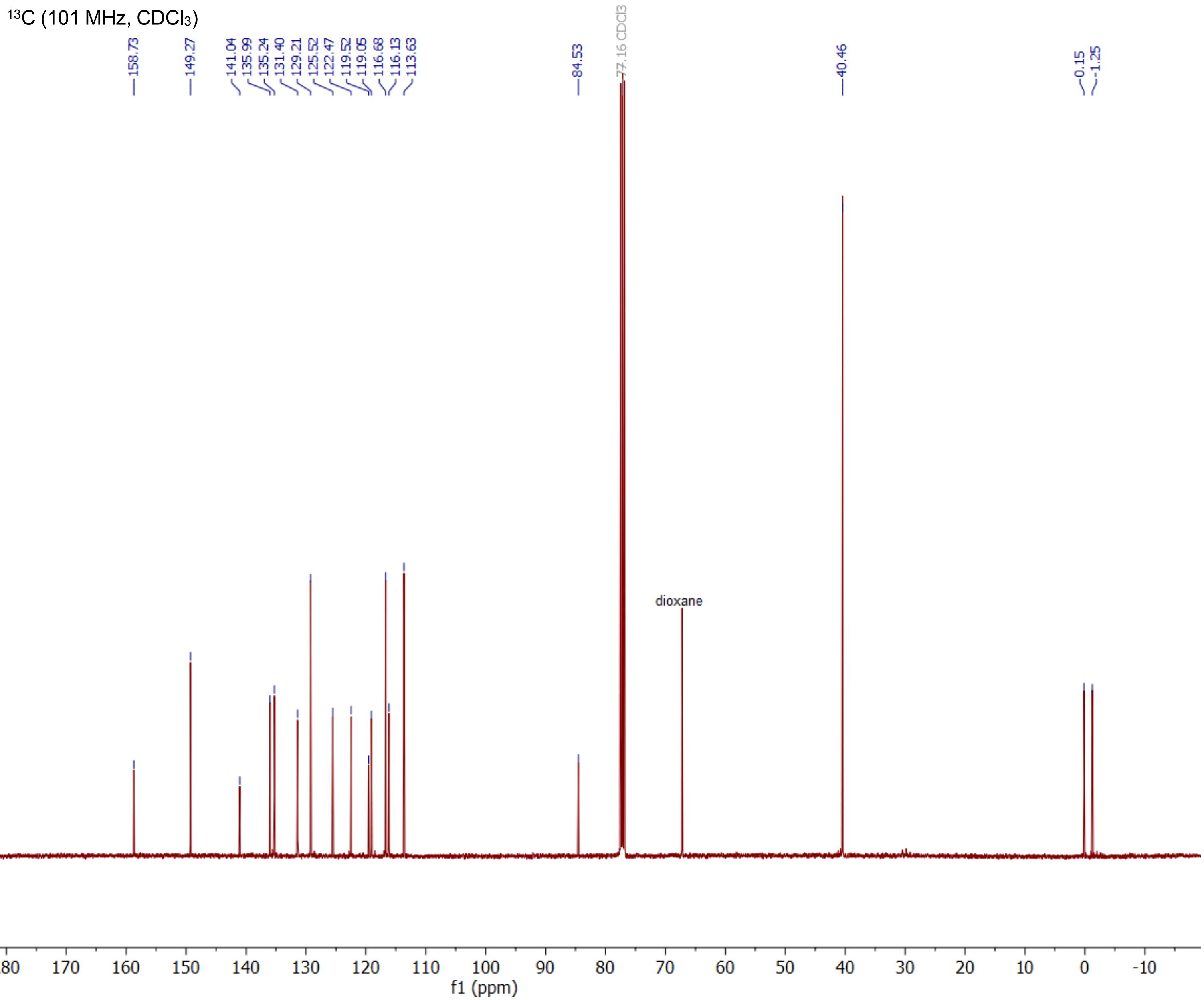
13C (101 MHz, CDCl3)
158.73
149.27
141.04
135.99
135.24
131.40
129.21
125.52
122.47
119.52
119.05
116.68
116.13
113.63
84.53
77.16 CDCl3
40.46
0.15
-1.25
dioxane
210 200 190 180 170 160 150 140 130 120 110 100 90 80 70 60 50 40 30 20 10 0 -10
f1 (ppm)

¹H (400 MHz, CDCl₃)

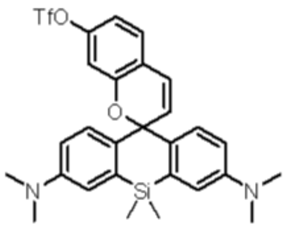

5l

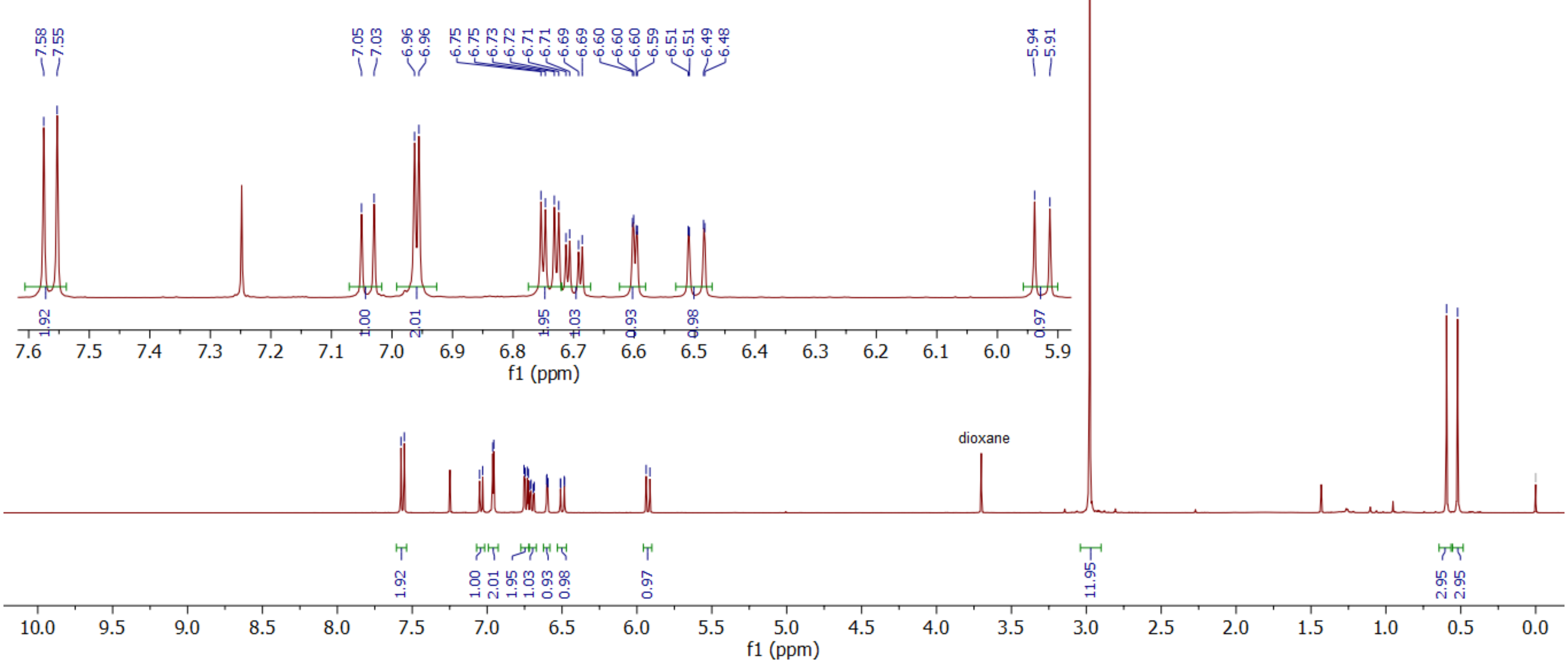

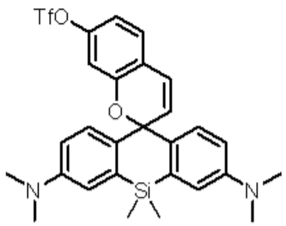

5l

^{19}F (376 MHz, $CDCl_3$)

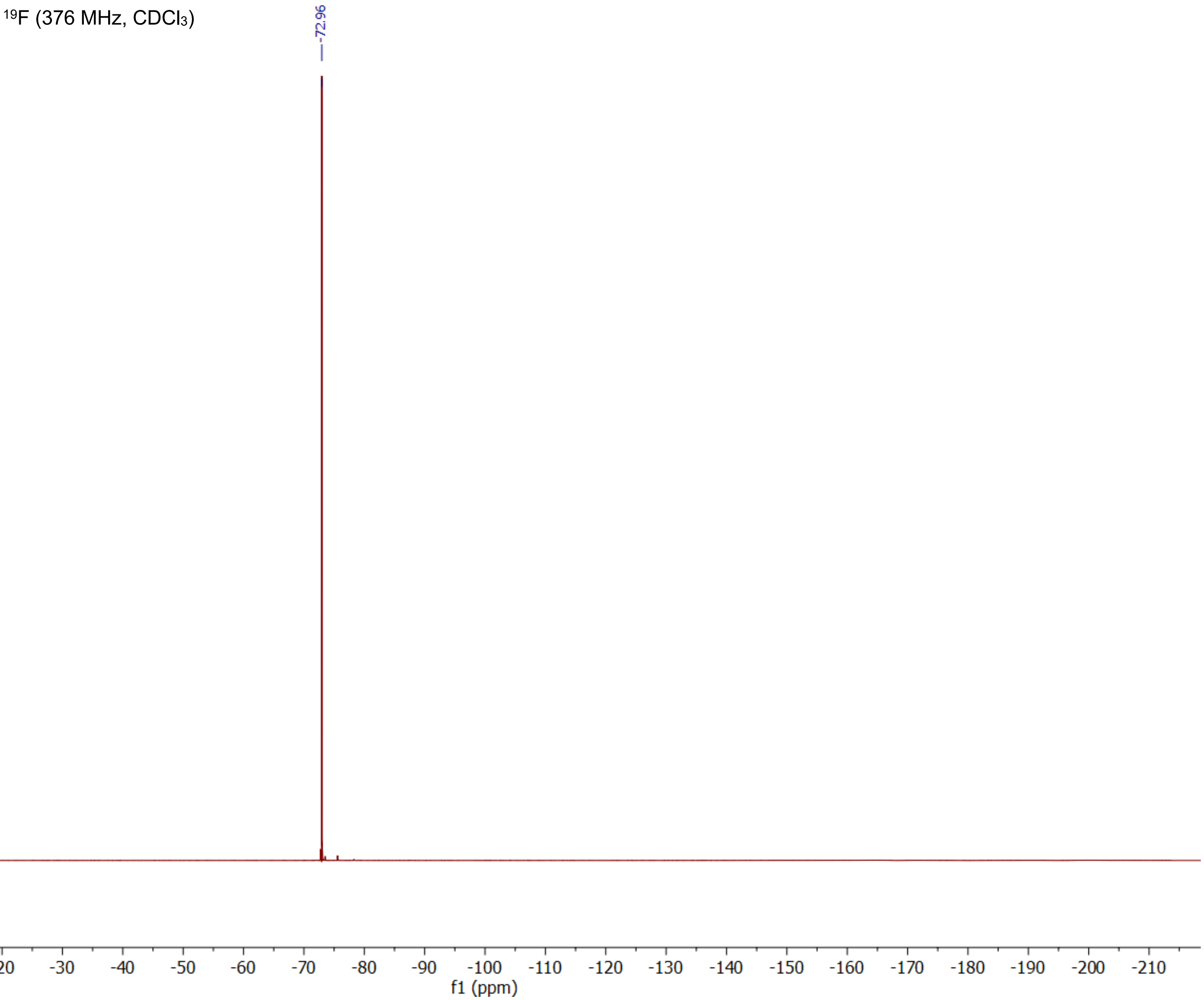

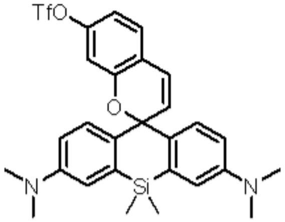

5l

^{13}C (101 MHz, $CDCl_3$)

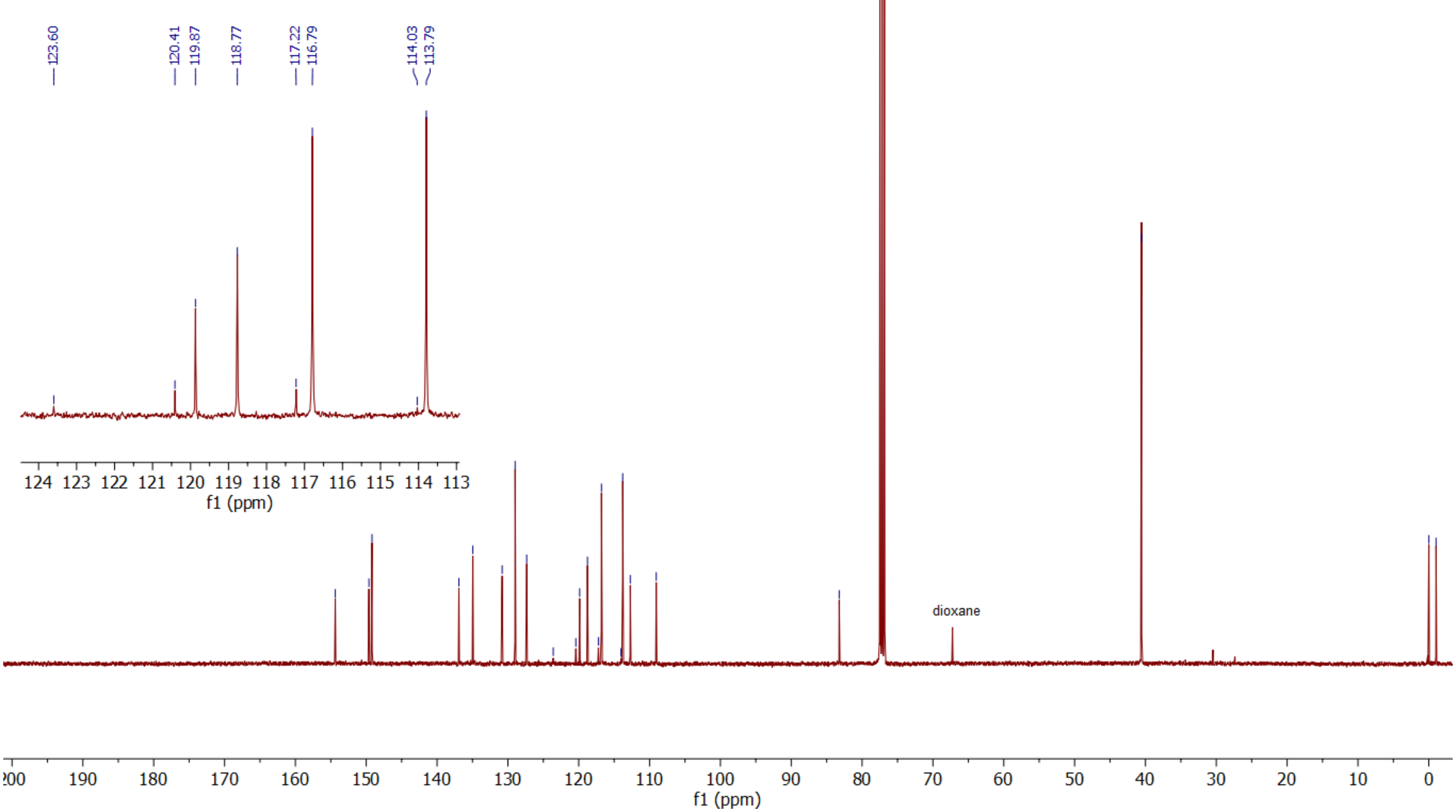

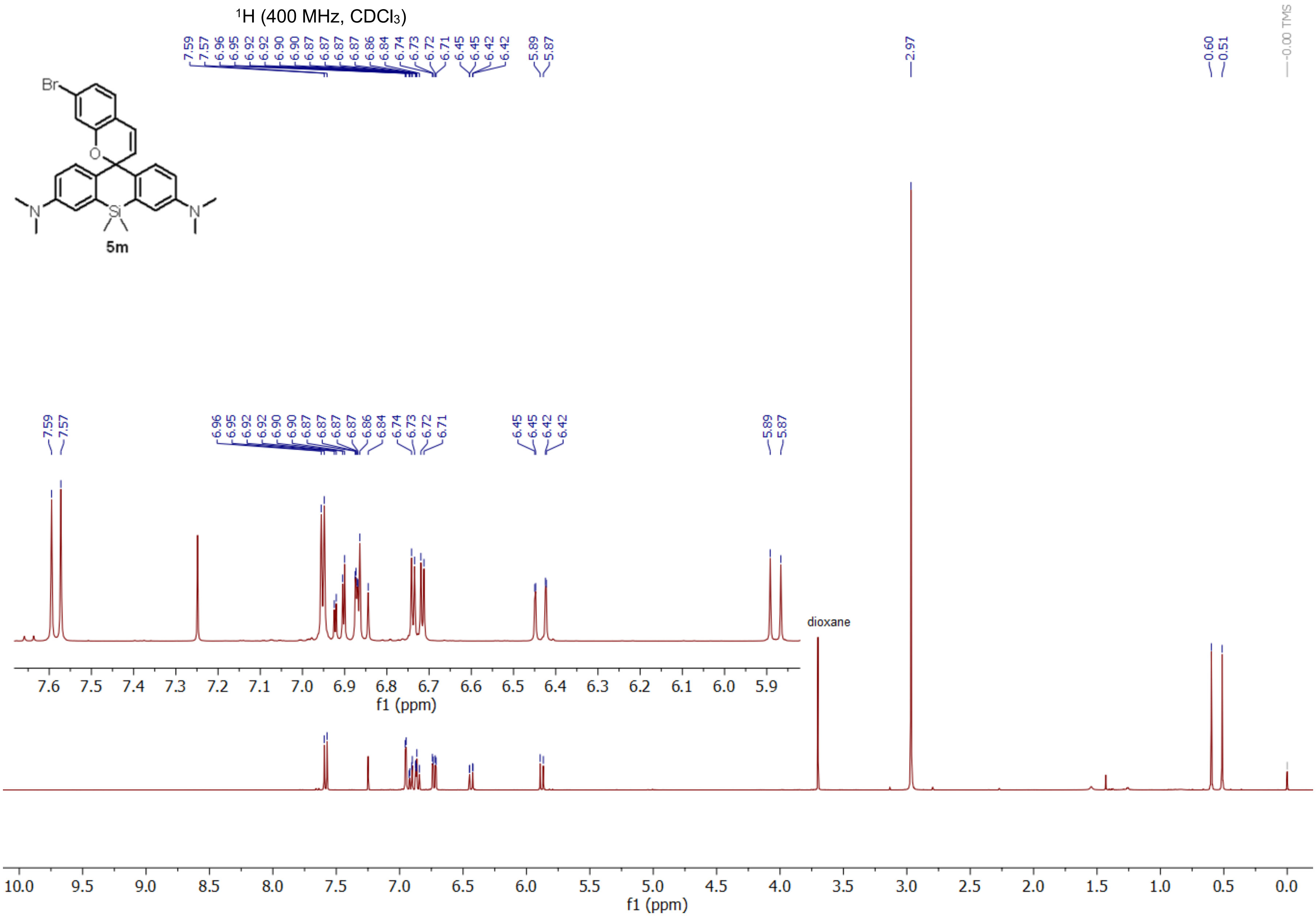

^{1}H (400 MHz, CDCl$_3$)
5m
dioxane
f1 (ppm)
-0.00 TMS

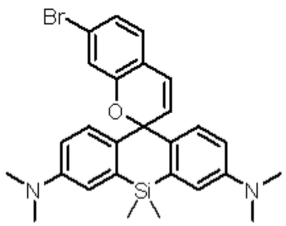

5m

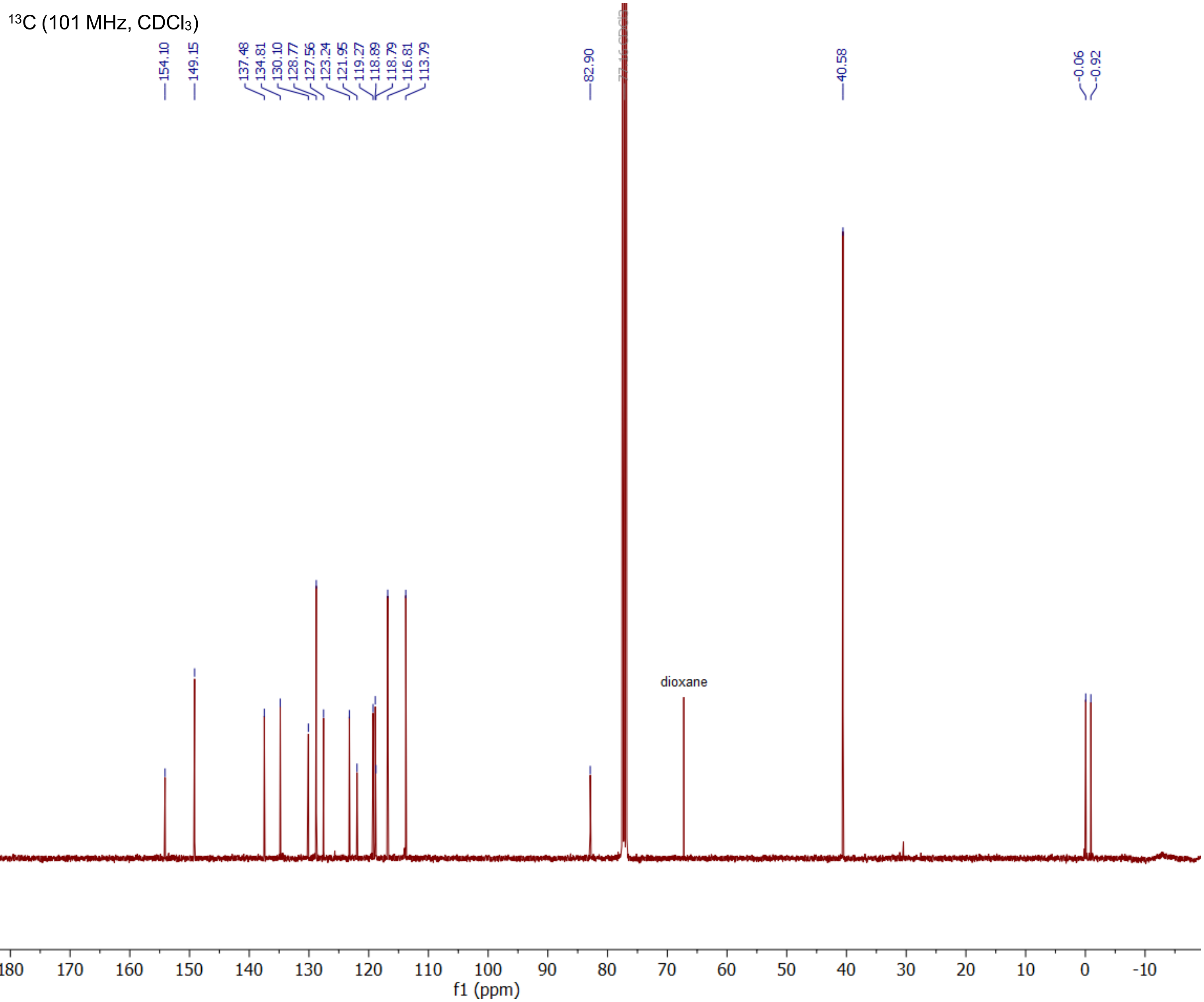

^{13}C (101 MHz, $CDCl_3$)
154.10
149.15
137.48
134.81
130.10
128.77
127.56
123.24
121.95
119.27
118.89
118.79
116.81
113.79
82.90
40.58
-0.06
-0.92
dioxane
f1 (ppm)

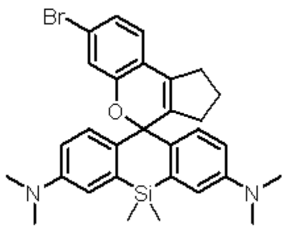

5n

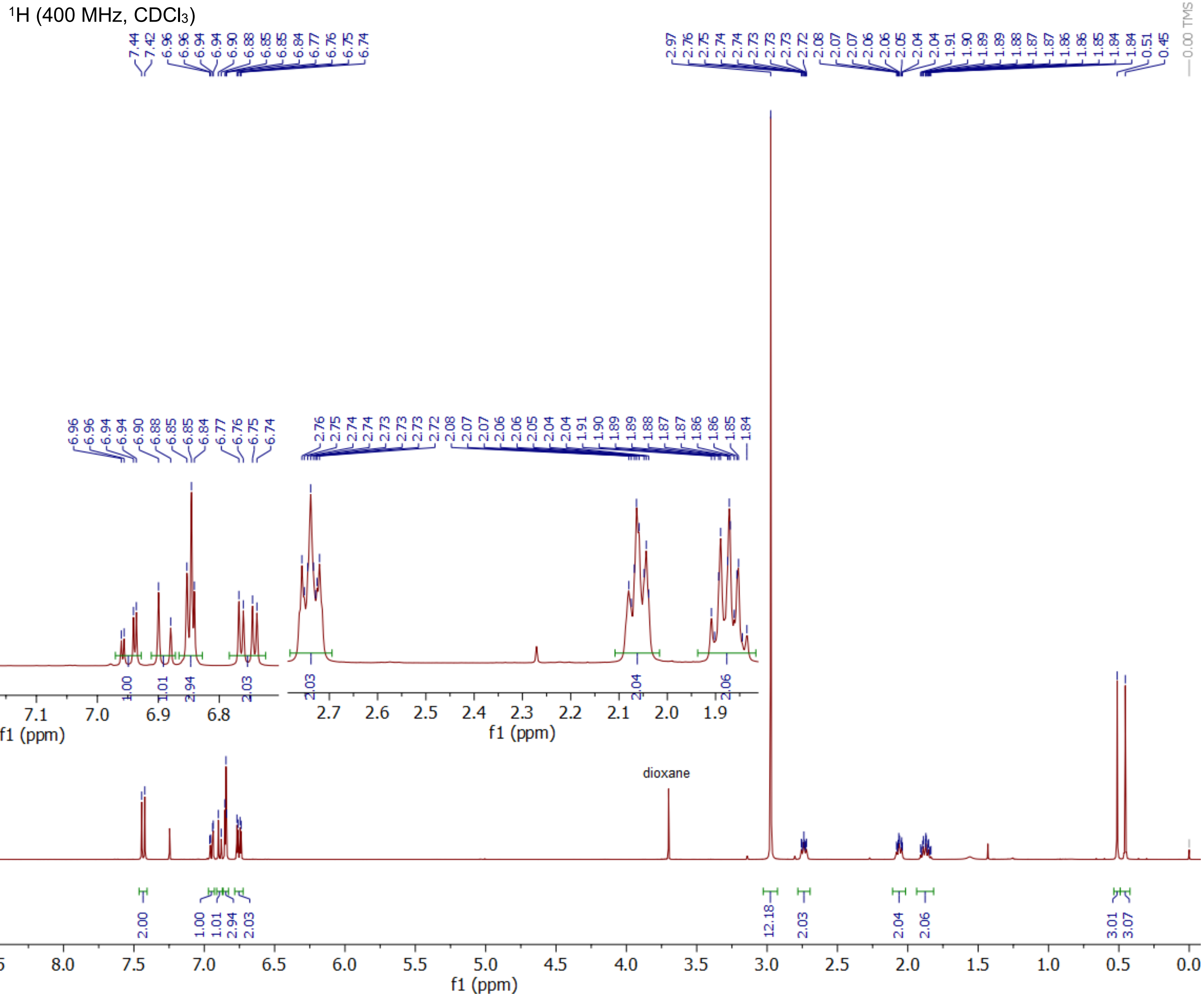

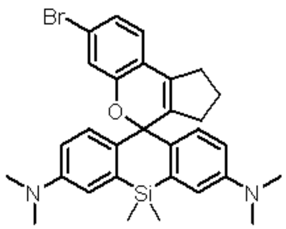

5n

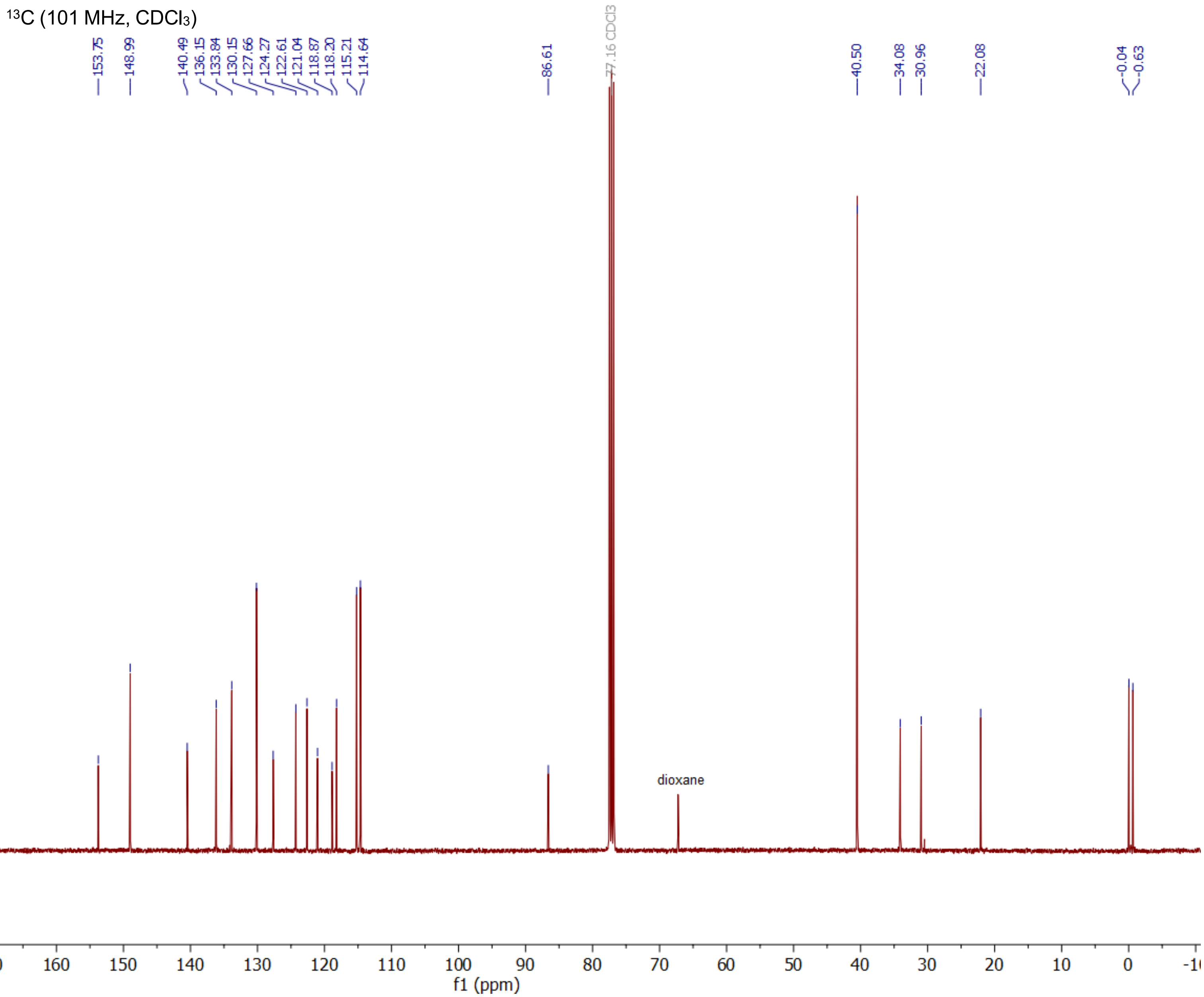

13C (101 MHz, CDCl3)
153.75
148.99
140.49
136.15
133.84
130.15
127.66
124.27
122.61
121.04
118.87
118.20
115.21
114.64
86.61
77.16 CDCl3
40.50
34.08
30.96
22.08
-0.04
-0.63
dioxane
200
190
180
170
160
150
140
130
120
110
100
90
80
70
60
50
40
30
20
10
0
f1 (ppm)

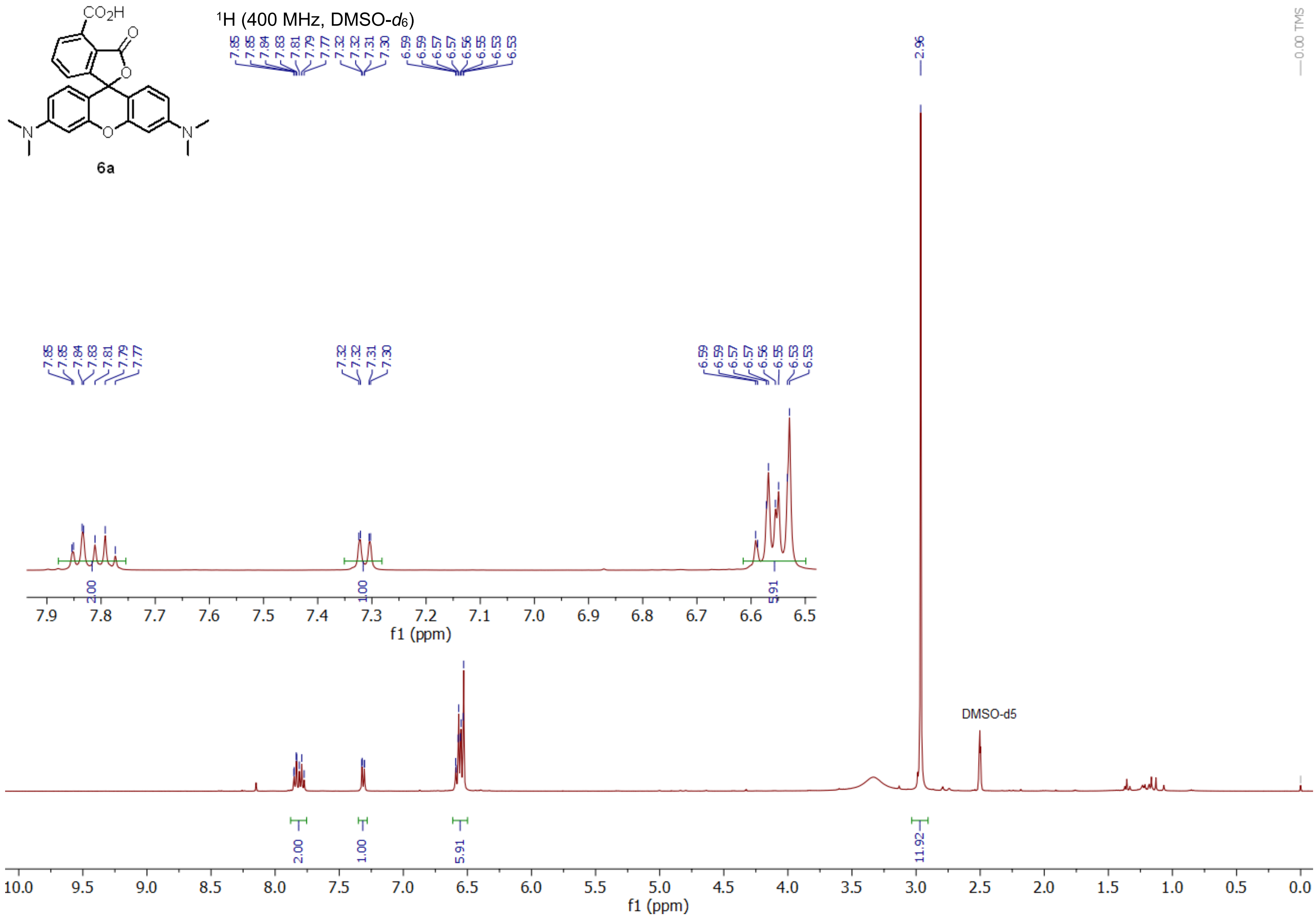

CO2H
6a
1H (400 MHz, DMSO-d6)
7.85 7.85 7.84 7.83 7.81 7.79 7.77 7.32 7.32 7.31 7.30 6.59 6.59 6.57 6.57 6.56 6.55 6.53 6.53
2.96
0.00 TMS
2.00
1.00
5.91
f1 (ppm)
DMSO-d5
11.92

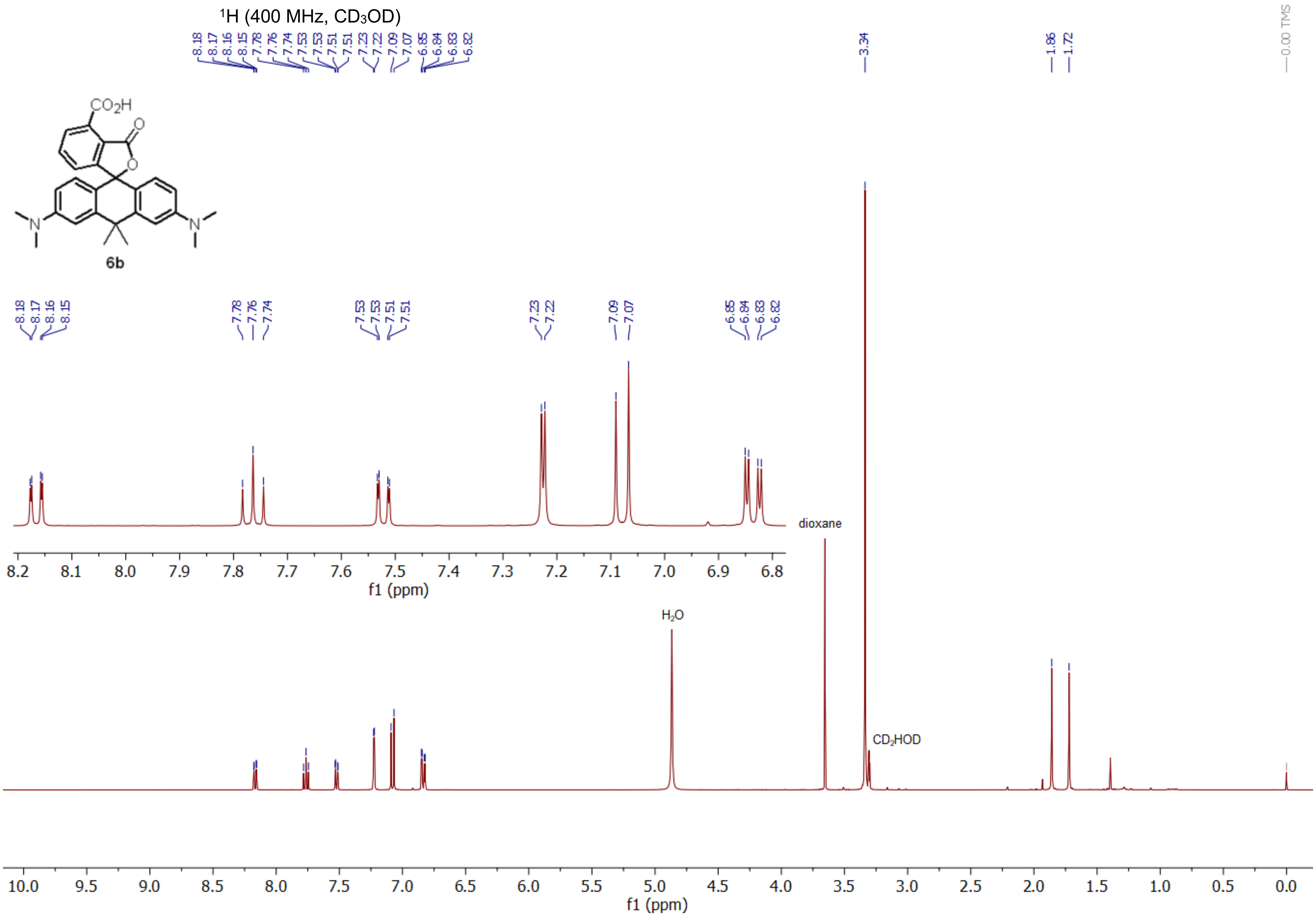

^{1}H (400 MHz, CD3OD)
8.18
8.17
8.16
8.15
7.78
7.76
7.74
7.53
7.53
7.51
7.51
7.23
7.22
7.09
7.07
6.85
6.84
6.83
6.82
3.34
1.86
1.72
0.00 TMS
CO2H
O
O
N
N
6b
dioxane
H2O
CD2HOD
f1 (ppm)
f1 (ppm)

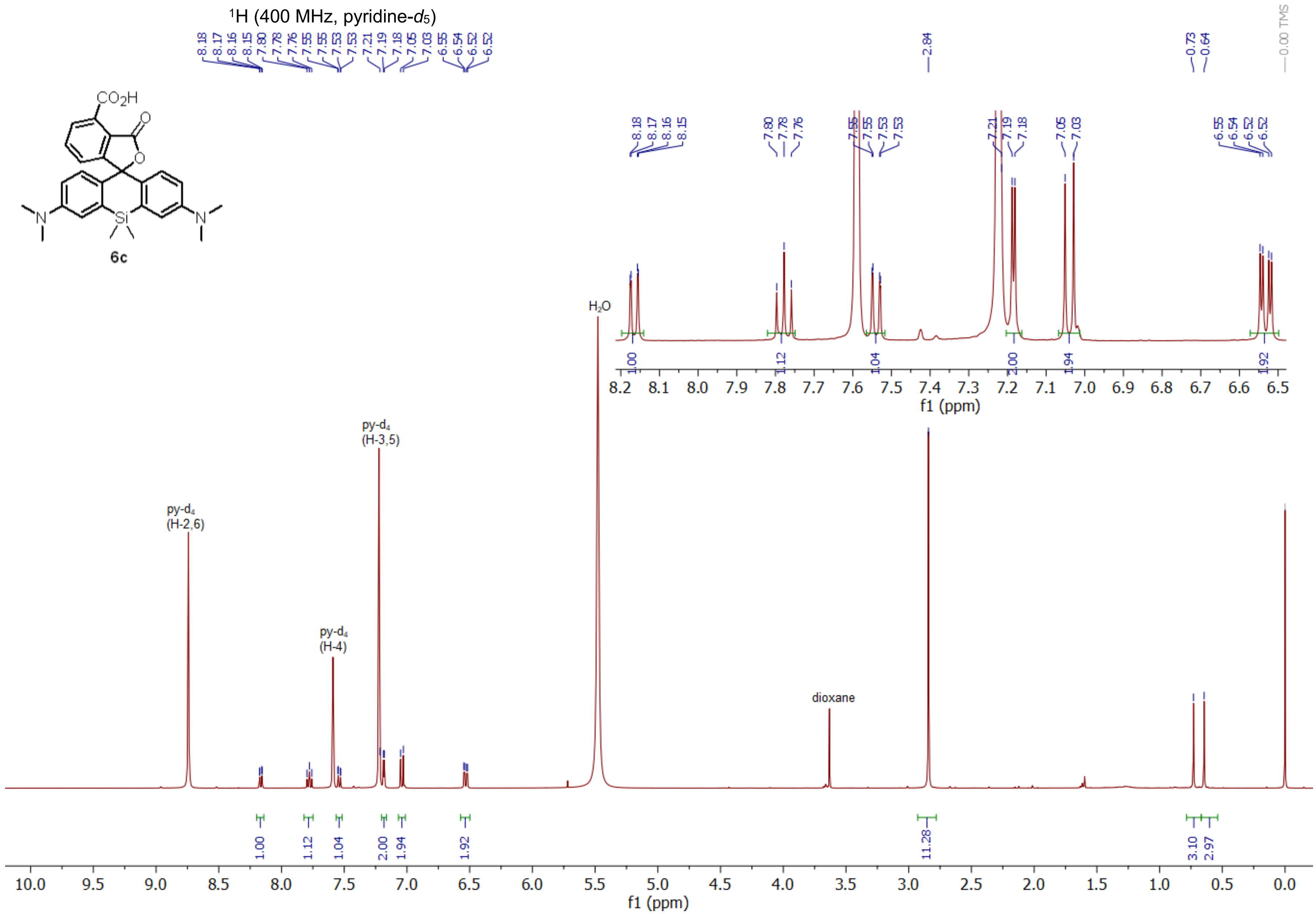

^{1}H (400 MHz, pyridine-d_5)
6c
CO_2H
Si
N
8.18 8.17 8.16 8.15 7.80 7.78 7.76 7.55 7.55 7.53 7.53 7.21 7.19 7.18 7.05 7.03 6.55 6.54 6.52 6.52
2.84
0.73 0.64
0.00 TMS
H_2O
py-d_4 (H-2,6)
py-d_4 (H-4)
py-d_4 (H-3,5)
dioxane
1.00 1.12 1.04 2.00 1.94 1.92 11.28 3.10 2.97
f1 (ppm)

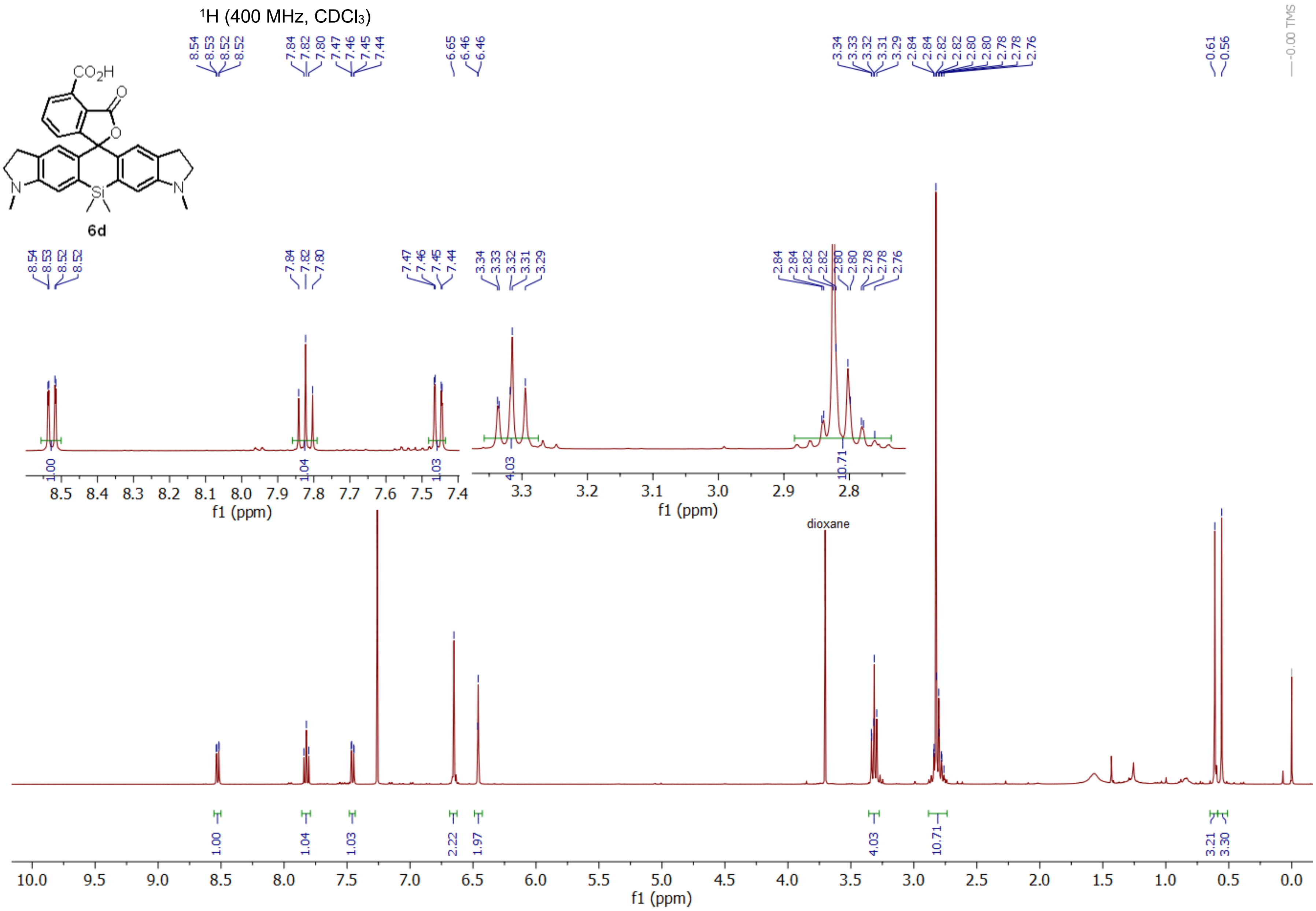

^{1}H (400 MHz, $CDCl_3$)
8.54 8.53 8.52 8.52
7.84 7.82 7.80 7.47 7.46 7.45 7.44
6.65 6.46 6.46
3.34 3.33 3.32 3.31 3.29 2.84 2.84 2.82 2.82 2.80 2.80 2.78 2.78 2.76
0.61 0.56
0.00 TMS
CO_2H
O
O
N
Si
N
6d
dioxane
1.00 1.04 1.03 2.22 1.97 4.03 10.71 3.21 3.30
f1 (ppm)

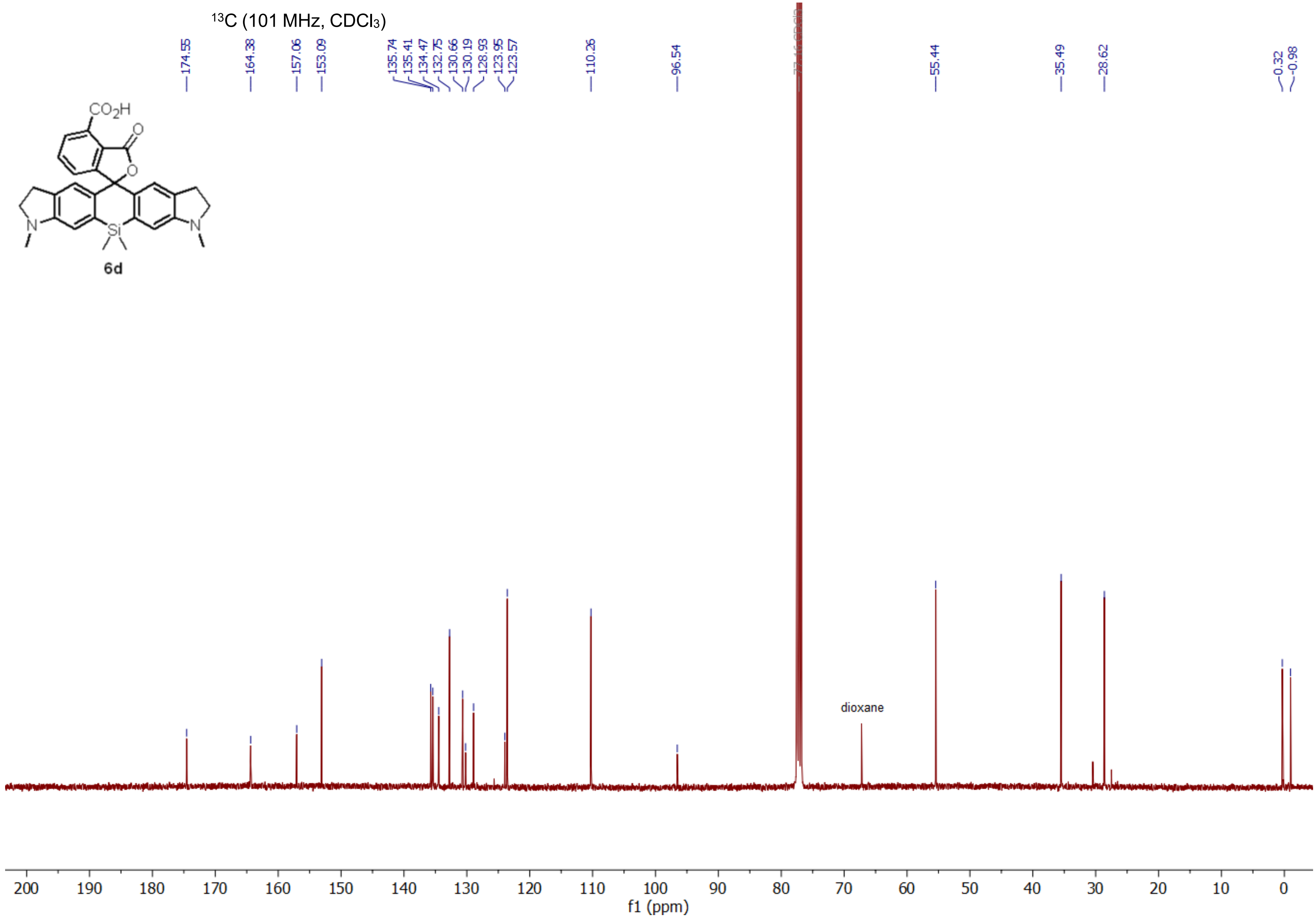

13C (101 MHz, CDCl3)
174.55
164.38
157.06
153.09
135.74
135.41
134.47
132.75
130.66
130.19
128.93
123.95
123.57
110.26
96.54
55.44
35.49
28.62
0.32
-0.98
CO2H
O
O
N
Si
N
6d
dioxane
200 190 180 170 160 150 140 130 120 110 100 90 80 70 60 50 40 30 20 10 0
f1 (ppm)

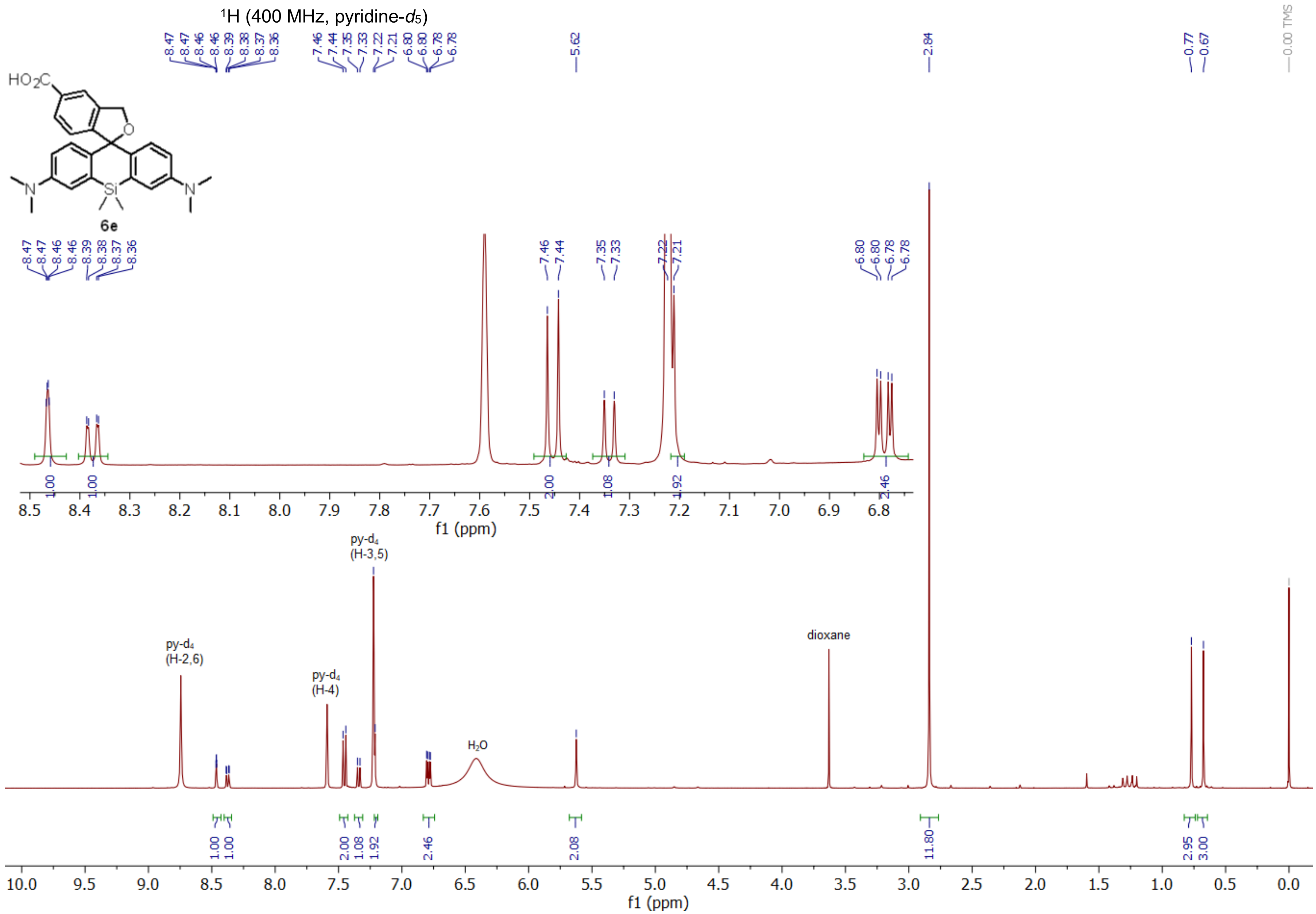

1H (400 MHz, pyridine-d5)
HO2C
O
N
Si
N
6e
py-d4 (H-2,6)
py-d4 (H-4)
py-d4 (H-3,5)
H2O
dioxane
0.00 TMS
f1 (ppm)

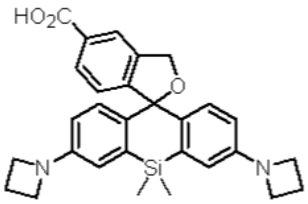

6f

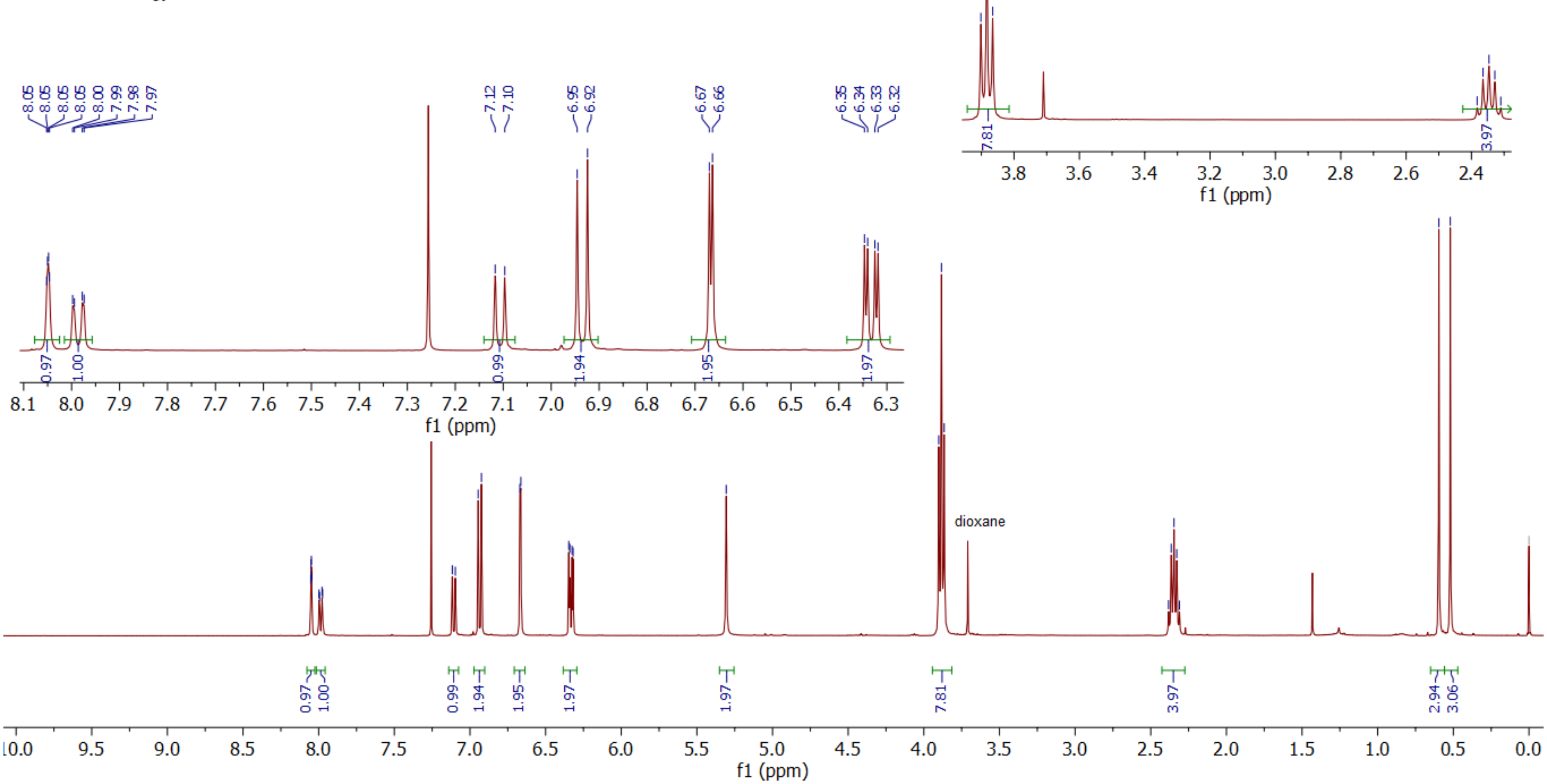

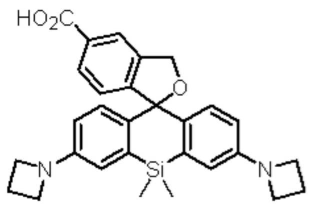
HO2C
O
N
Si
N

6f

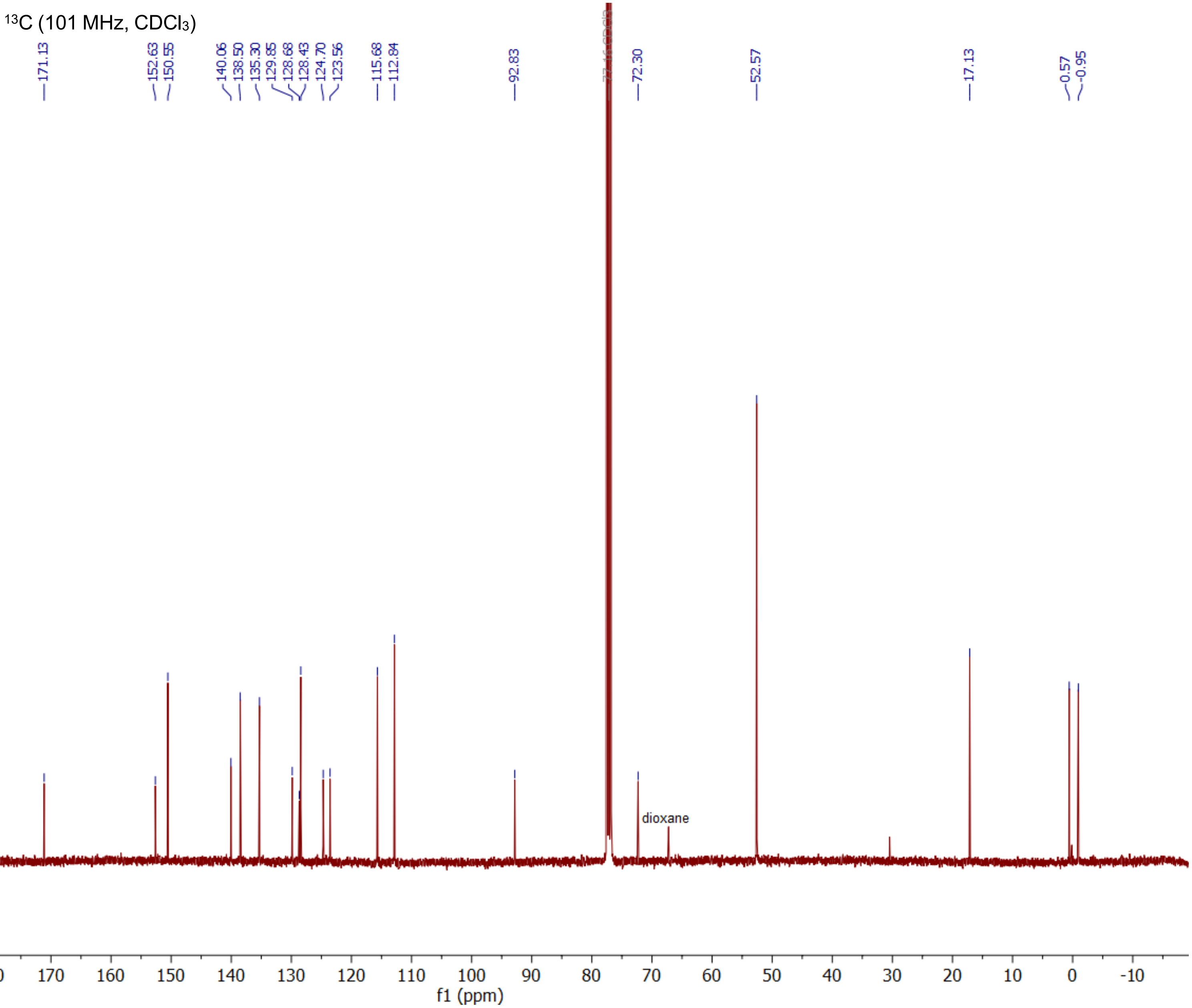
13C (101 MHz, CDCl3)
171.13
152.63
150.55
140.06
138.50
135.30
129.85
128.68
128.43
124.70
123.56
115.68
112.84
92.83
72.30
52.57
17.13
0.57
-0.95
dioxane
210 200 190 180 170 160 150 140 130 120 110 100 90 80 70 60 50 40 30 20 10 0 -10
f1 (ppm)

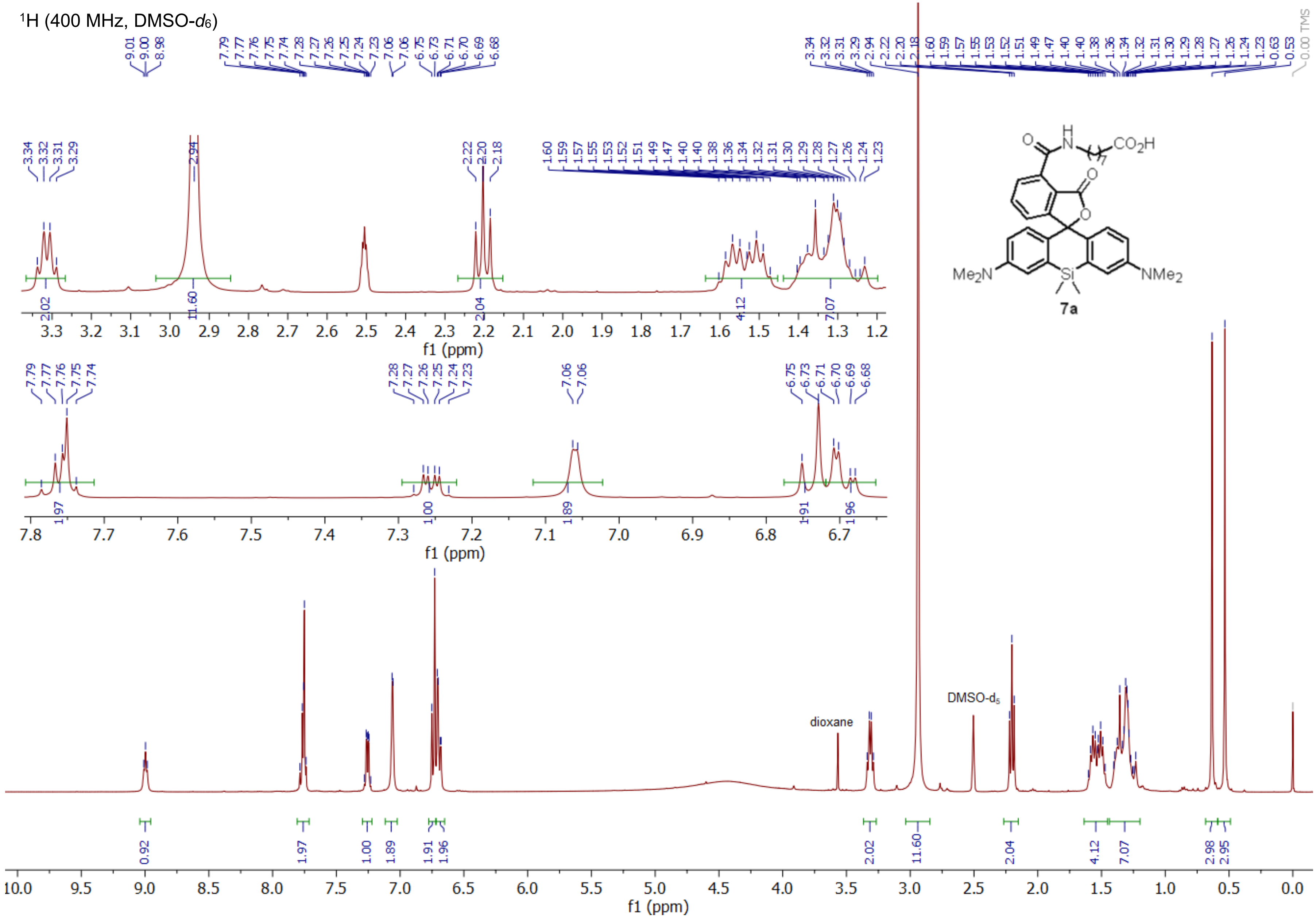

^{1}H (400 MHz, DMSO-d_6)
dioxane
DMSO-d_5
f1 (ppm)
Me$_2$N
NMe$_2$
CO$_2$H
Si
7a

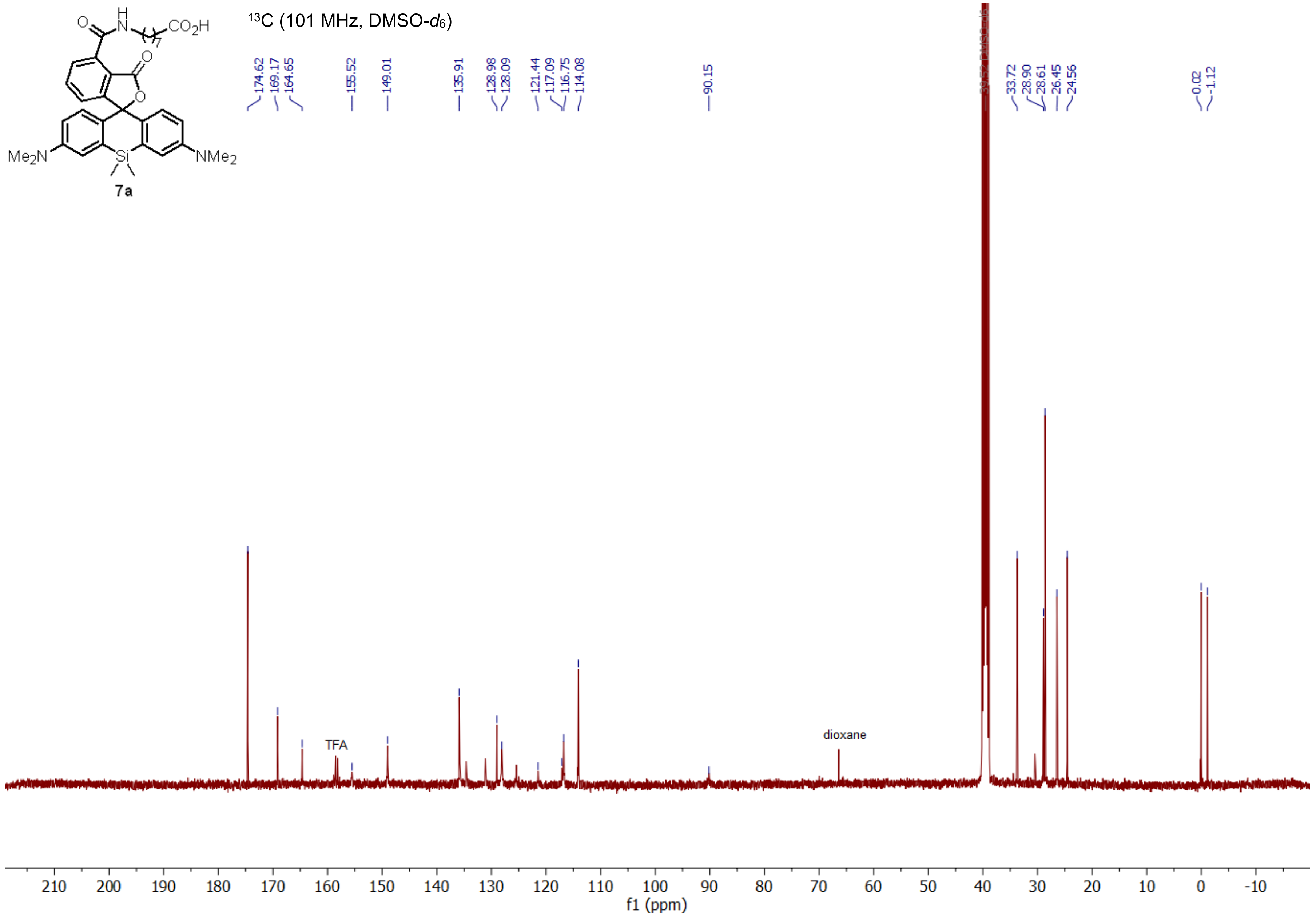

Me2N
NMe2
Si
CO2H
7a
13C (101 MHz, DMSO-d6)
174.62
169.17
164.65
155.52
149.01
135.91
128.98
128.09
121.44
117.09
116.75
114.08
90.15
33.72
28.90
28.61
26.45
24.56
0.02
-1.12
TFA
dioxane
f1 (ppm)

^{1}H (400 MHz, DMSO-d_6)

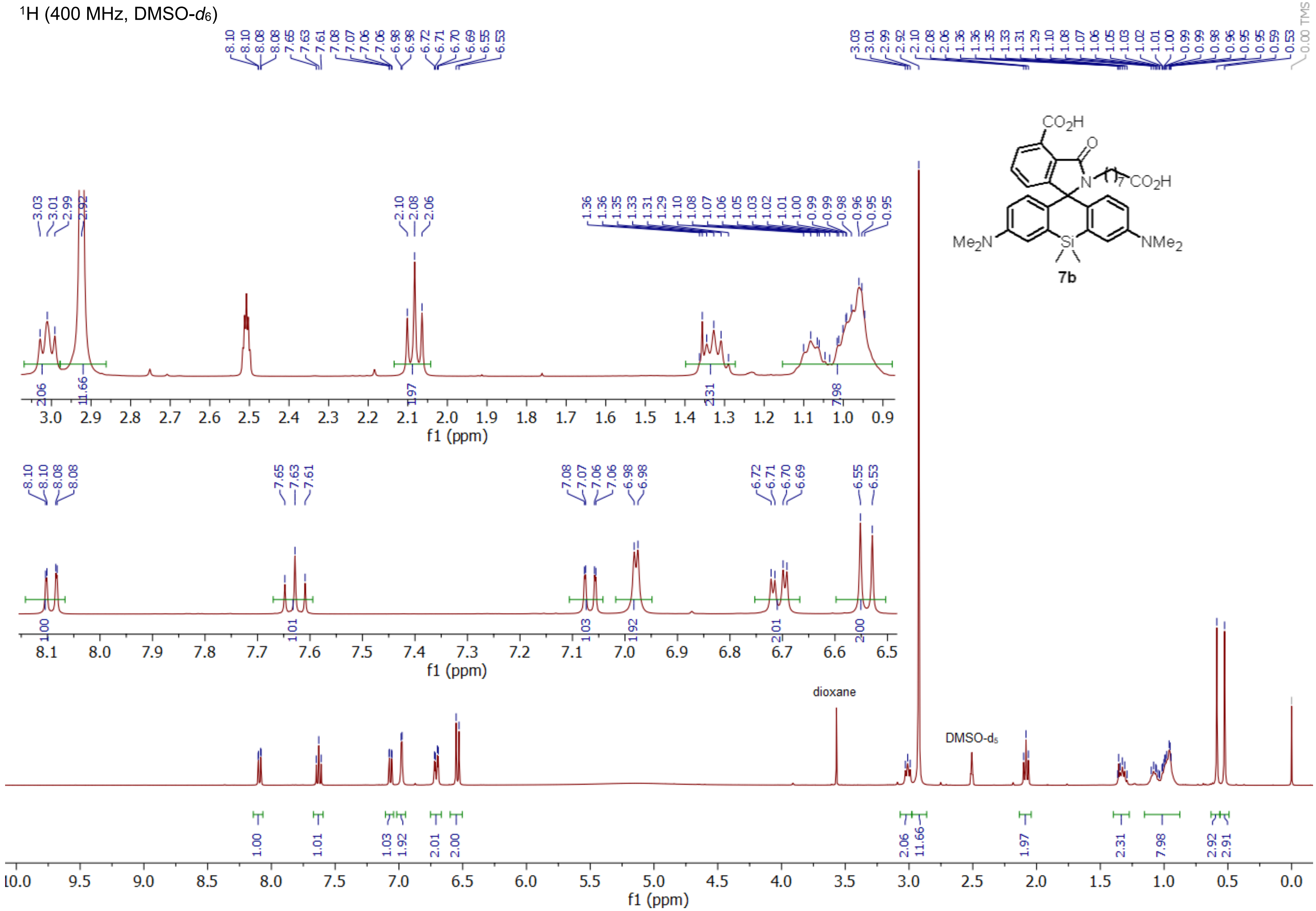

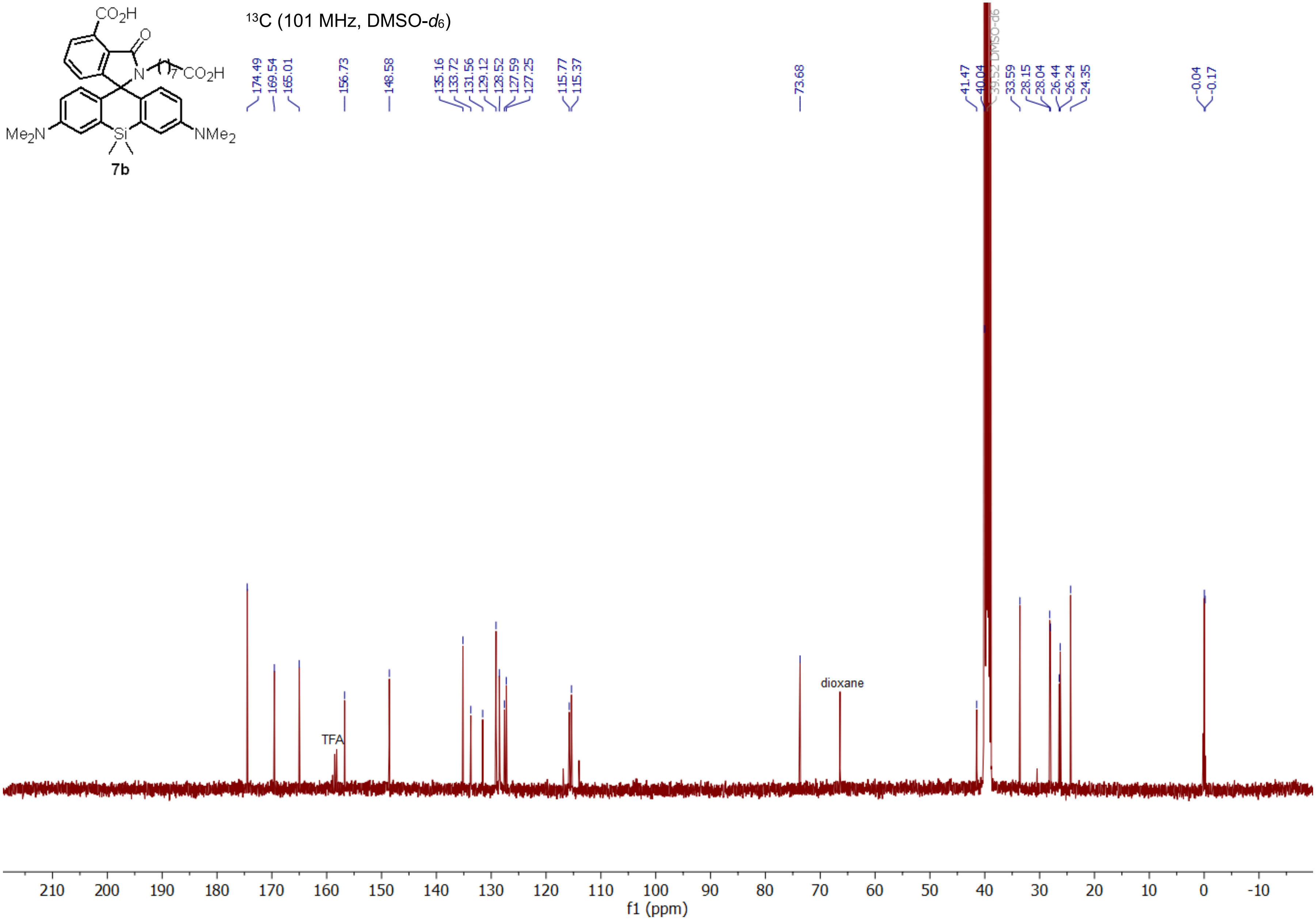

CO2H
O
N
CO2H
7
Me2N
Si
NMe2
13C (101 MHz, DMSO-d6)
174.49
169.54
165.01
156.73
148.58
135.16
133.72
131.56
129.12
128.52
127.59
127.25
115.77
115.37
73.68
41.47
40.04
39.52 DMSO-d6
33.59
28.15
28.04
26.44
26.24
24.35
-0.04
-0.17
TFA
dioxane
f1 (ppm)

7b

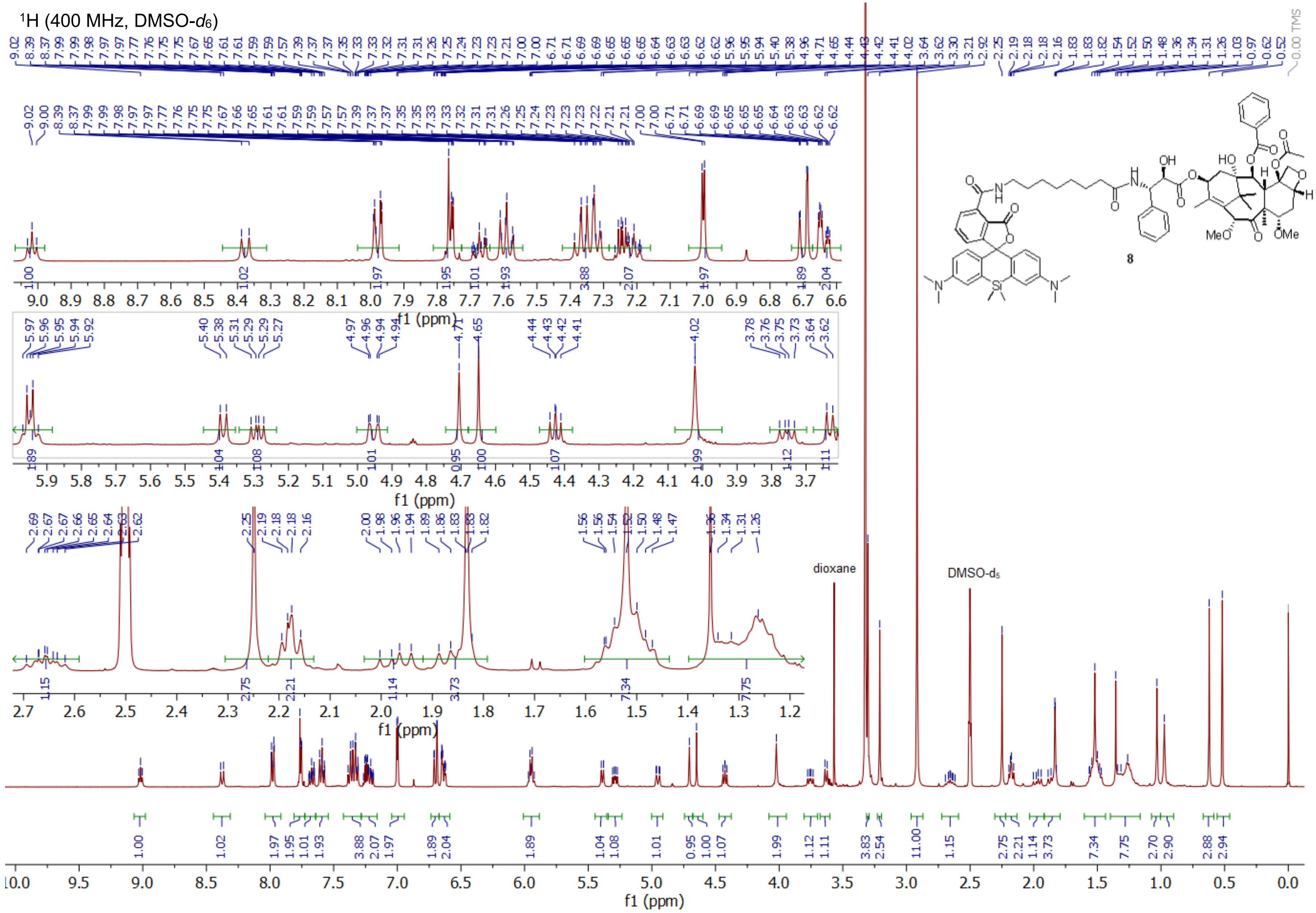

^{1}H (400 MHz, DMSO-d_6)
dioxane
DMSO-d_5
TMS
8
f1 (ppm)

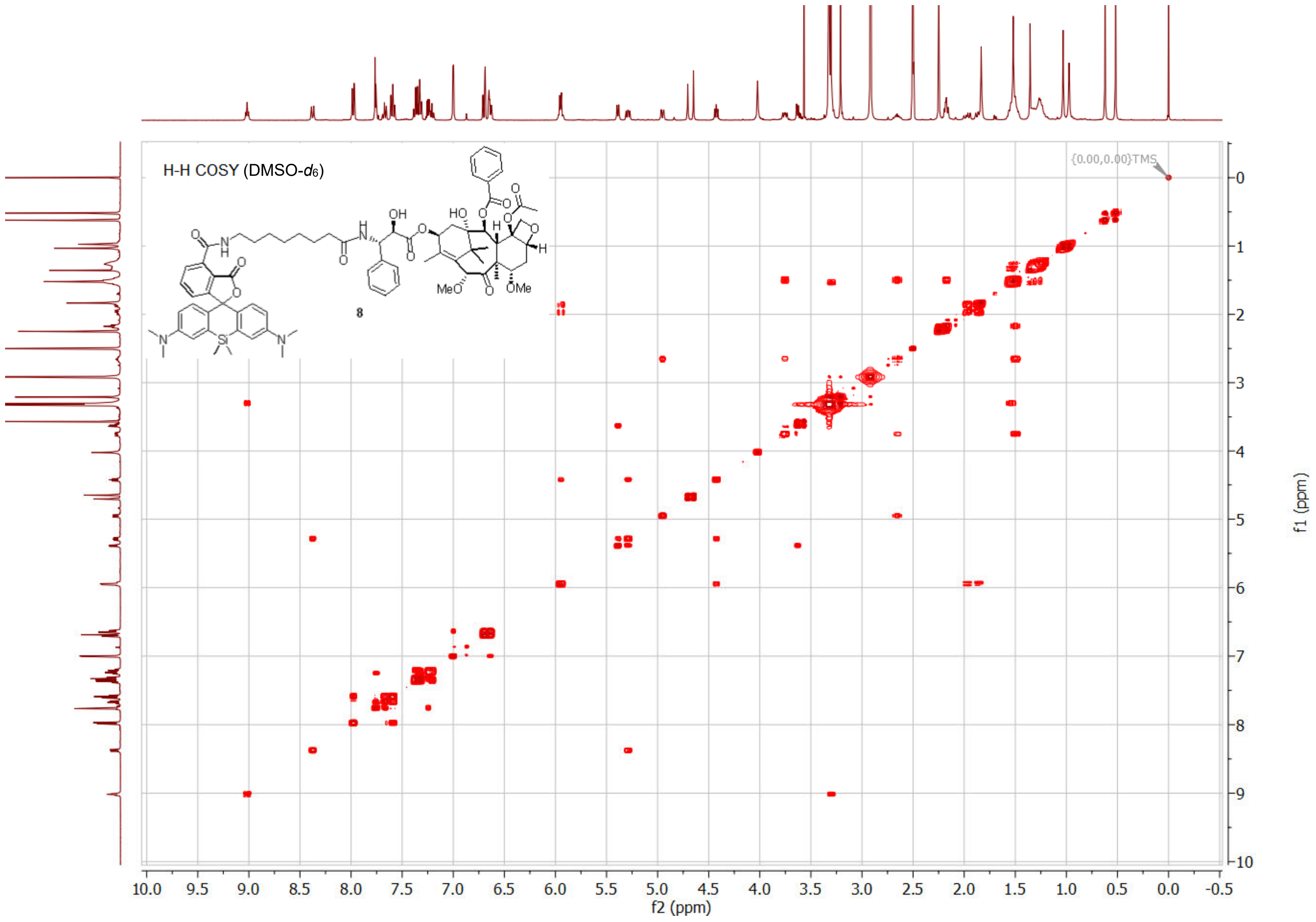
H-H COSY (DMSO-d6)
8
{0.00,0.00}TMS
f2 (ppm)
f1 (ppm)

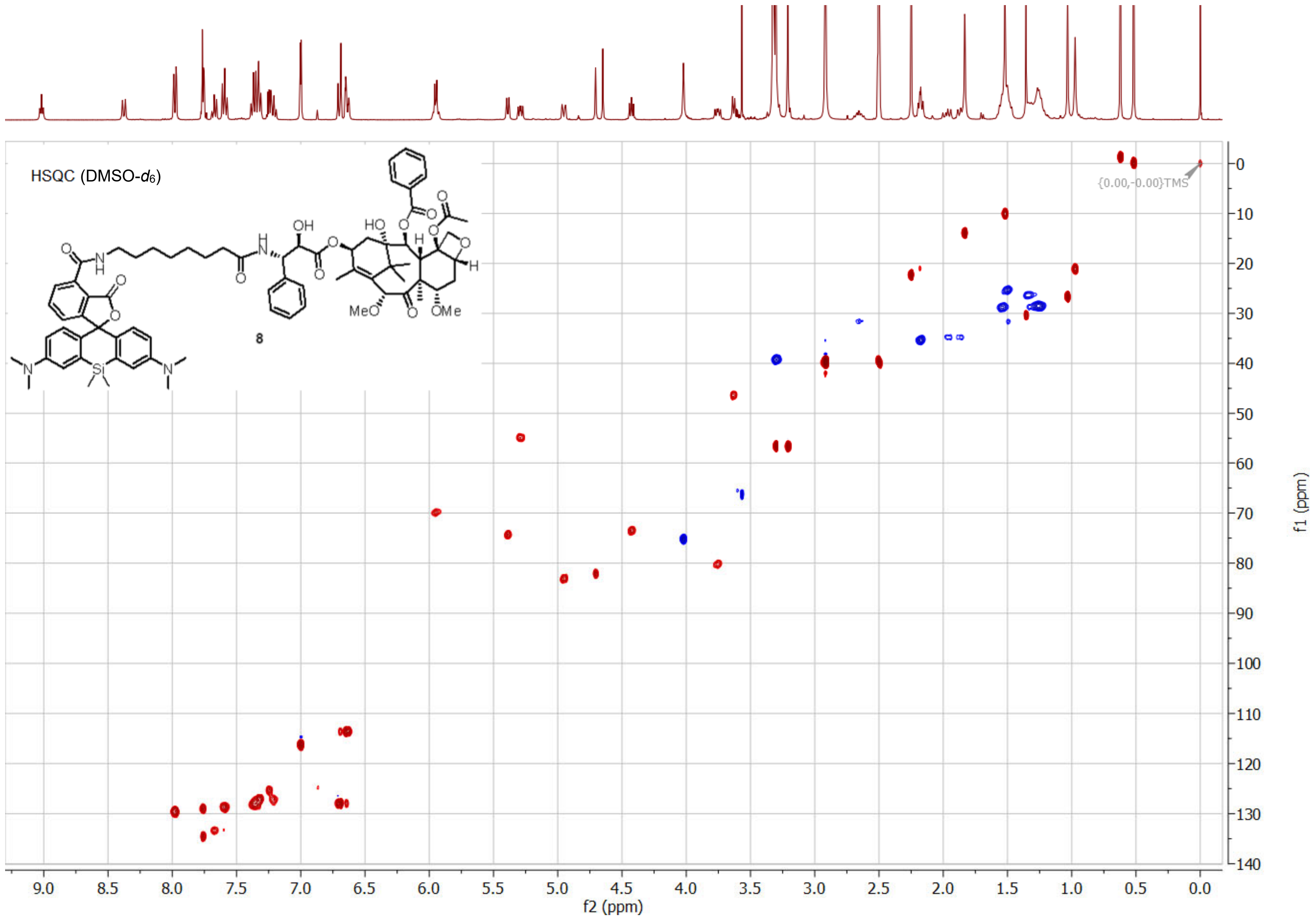
HSQC (DMSO-d6)
8
{0.00,-0.00}TMS
f2 (ppm)
f1 (ppm)
